# Mechanistic Insights on the Selectivity of the Tandem Heck–Ring-Opening of Cyclopropyldiol Derivatives

**DOI:** 10.1021/jacsau.1c00547

**Published:** 2022-03-05

**Authors:** Anthony Cohen, Alexander Kaushansky, Ilan Marek

**Affiliations:** Schulich Faculty of Chemistry, Technion − Israel Institute of Technology, Haifa 3200009, Israel

**Keywords:** diastereocontrol, Heck addition, ring-opening, selectivity, cyclopropane diol, palladium, lactone

## Abstract

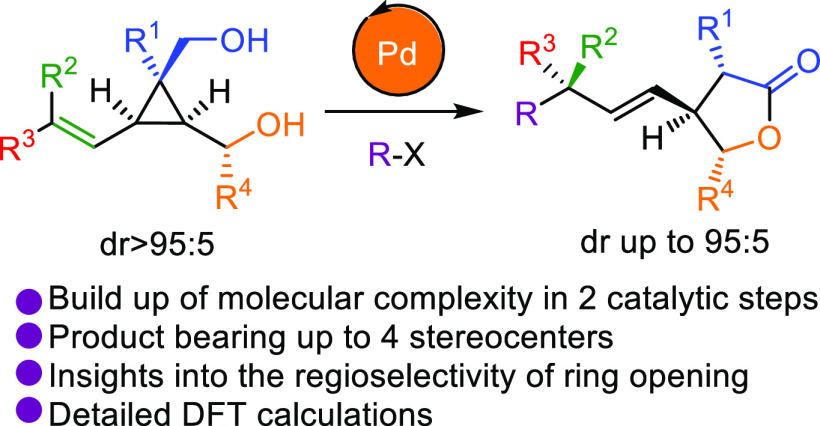

The preparation of a new class of alkenyl cyclopropyl diols, easily available through a copper-catalyzed carbometalation reaction of cyclopropenes, has enabled the study of key mechanistic aspects of the tandem Heck–cyclopropane ring-opening reaction. Utilizing these substrates containing two distinct hydroxyl groups allowed us to examine parameters affecting the reaction outcome and selectivity. The combination of these experimental results with detailed DFT studies shed light on the mechanism governing the regio- and stereoselectivity of the cyclopropane ring-opening. A thorough investigation displayed the dual roles fulfilled by the hydroxyl group during the reaction, which is key to this remarkable transformation. In addition to its mechanistic implication, the reaction granted access to various lactones possessing up to four stereocenters as a single diastereomer, conveniently prepared in only two catalytic steps from easily accessible achiral cyclopropenes.

## Introduction

**Scheme 1:**
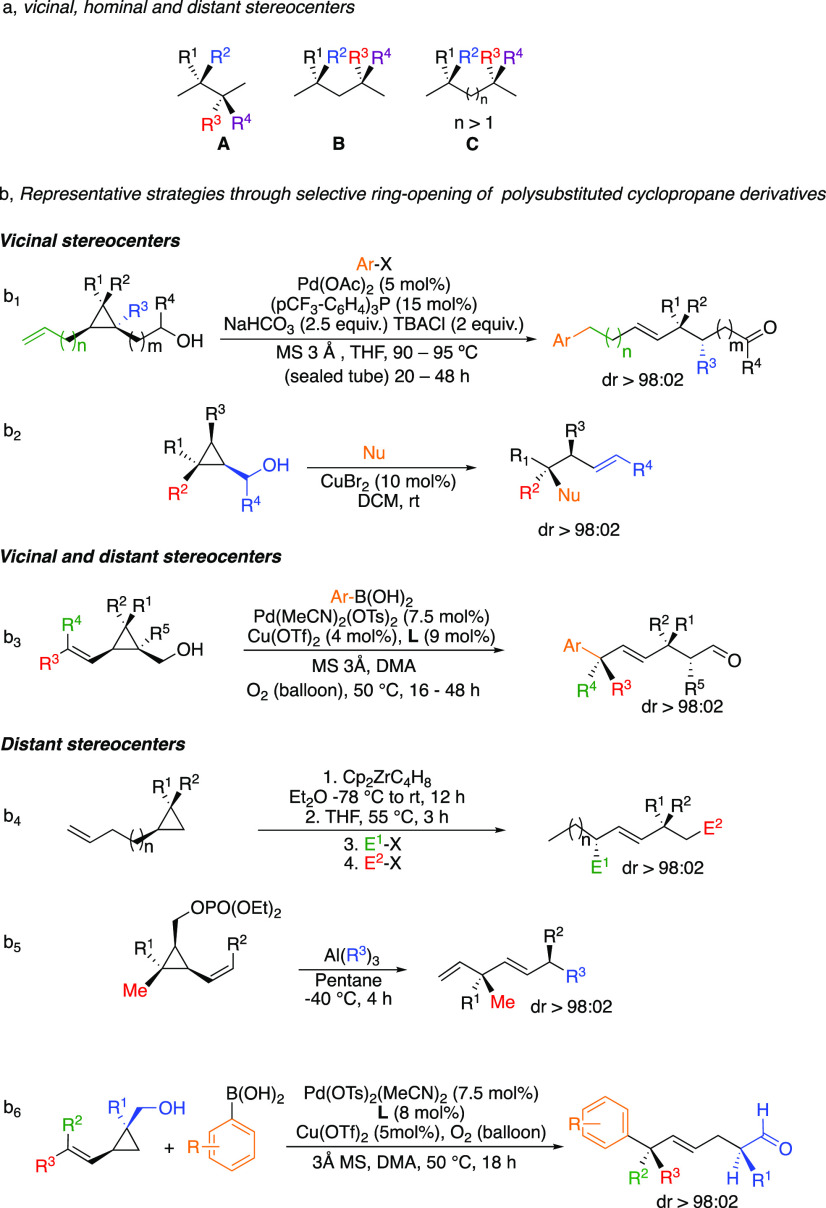
Synthesis of Vicinal and Distant Stereocenters

The rapid and efficient construction of molecular complexity from simple and easily accessible starting materials represents a major goal in modern organic synthesis.^1−6^ The formation of diastereo- and enantiomerically pure vicinal (**A**), hominal (**B**), or distant (**C**) stereocenters in acyclic systems illustrates the pinnacle of these challenges (Scheme 1a). Among all possible strategies to reach these structures,^7−13^ the inherent strain of polysubstituted cyclopropanes could serve as a central platform for selective ring-opening of three-membered rings toward the formation of these desired acyclic motifs.^14,15^ In this context, we have recently reported several modular and stereodivergent strategies to construct congested acyclic molecular frameworks that bear several stereogenic centers at different positions with remarkably high levels of stereocontrol (Scheme 1b).^16−24^ Notably, using these strategies, the enantioselective preparation of the natural product botryococcene and its epimer^25^ as well as the diastereoselective preparation of the side chain of α-tocopherol^26^ could be easily and efficiently achieved in a few catalytic steps from commercially available starting materials. Interestingly, the selectivity of the ring-opening is usually dictated by either the presence of an electron-withdrawing group,^27,28^ a leaving group^16,25,29,30^ that polarizes a specific σ bond through a “push–pull” effect,^15^ by constraints of a bicyclic system^31−33^ (Scheme 1, equations 1b_2_ and 1b_5_), or by the formation of the less substituted and more stable primary alkyl metal intermediate^18,34,35^ (Scheme 1, equation 1b_4_).

However, we have recently revealed that the ring-opening selectivity can also be dictated by an unprecedented transformation of an alcohol into an aldehyde (Scheme 1, eqs 1b_1_, 1b_3_, and 1b_6_) as a driving force.^17,19,21,26^ Evidence for the utility of this transformation in various reactions, notably the Heck^36−38^ relay reaction, was previously reported.^39−45^

**Scheme 2:**
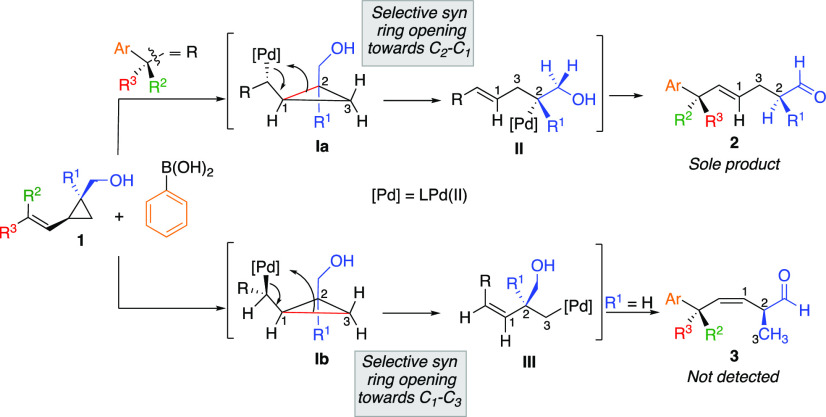
Mechanistic Discrepancies

This selectivity was particularly puzzling^46^ for the example described in eq 1b_6_, as a potential competition could exist between the formation of two products, namely aldehydes **2** and **3** (Scheme 2). The former would result from the ring-opening of the intermediate **Ia** through a postulated *syn*(47) C_1_–C_2_ bond cleavage to lead to the formation of an *E*-configurated secondary (R^1^ = H) or tertiary (R^1^ = CH_3,_ aryl) alkyl-palladium species **II** that would subsequently undergo a β-H elimination and hydride reinsertion to provide **2**.^26^ On the other hand, if the ring-opening would proceed through the cleavage of the C_1_–C_3_ bond (**Ib**) and the β-fragmentation still occurs through a *syn*-process, the thermodynamically more stable primary alkyl palladium intermediate **III** would be formed, and after a sequence of similar β-H elimination (R^1^ = H) and hydride insertion, *Z*-configurated aldehyde **3** should be formed.

Remarkably, the Pd-catalyzed addition of aryl boronic acid to **1** led to the exclusive formation of **2** without any trace of the aldehyde **3**, underlining that the ring-opening preferably proceeds through the cleavage of the C_2_–C_1_ bond, leading to a more substituted organometallic intermediate **II**. Although it became experimentally clear from this study and others^17,19,21,26^ that the presence of an alcohol was controlling the selectivity of the β-carbon fragmentation, the origin of this selective transformation remained elusive.

**Figure 1:**
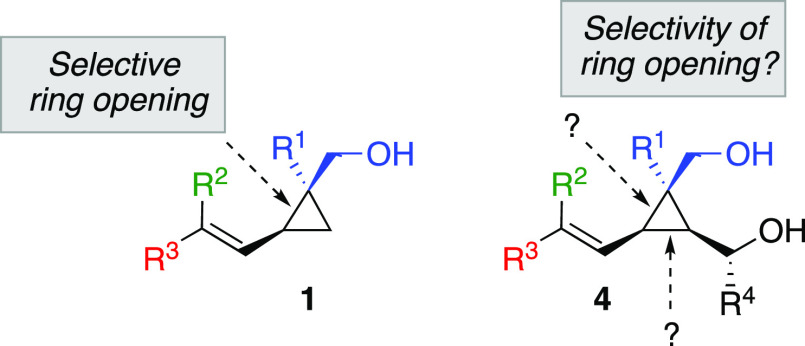
Selectivity for the ring-opening.

This result raised fundamental questions regarding the exact origin of the regioselectivity of the C–C bond cleavage and the role played by the hydroxyl group. To better understand the reaction mechanism, we embarked on a joined effort deciphering on one hand the reaction mechanism by computational analysis and on the other hand to experimentally investigate the parameters controlling the selectivity of the ring-opening. In other words, if an alcohol controls the regioselectivity of the ring-opening of cyclopropyl carbinol **1**, what would be the selectivity when a cyclopropyl diol is concerned? Which of the two alcohol moieties of **4**, with different substitution patterns, would provide the driving force for a selective ring-opening, if any, and why (Figure 1)? Will the overall process still be regio- and diastereoselective?

**Scheme 3:**
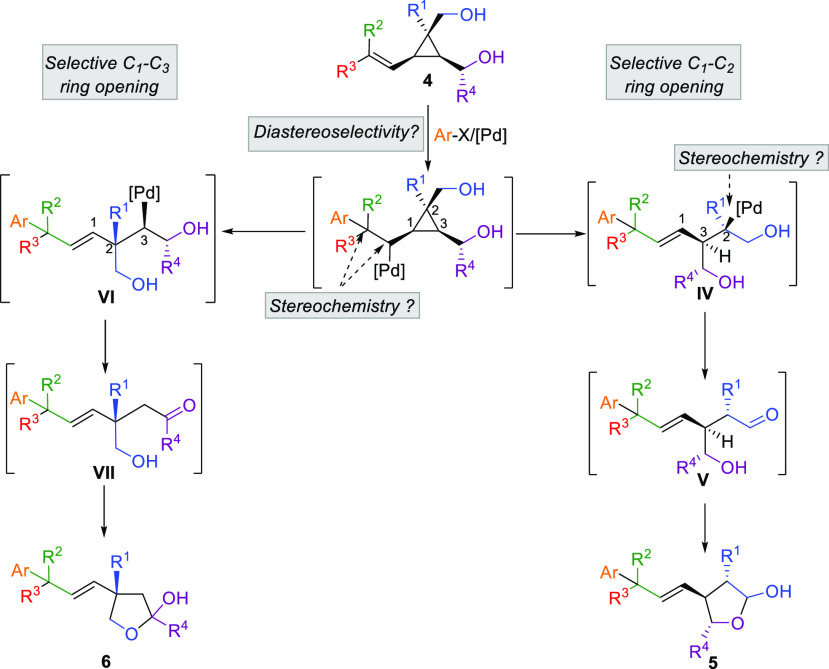
Potential Products for the Pd-Catalyzed Tandem Heck–Ring-Opening Reaction of Cyclopropyl Diol **4**

For instance, what would be the diastereoselectivity of the Heck addition to **4** (Scheme 3), which hydroxyl (if any) would control the diastereofacial addition of the arylpalladium complex to the double bond? In the subsequent step, which carbon–carbon bond is going to be cleaved? Would it be the cleavage of the C_1_–C_2_ bond to provide the more substituted alkyl palladium species **IV** (when R^1^ = alkyl, aryl) and ultimately give **V** by a sequence of β-H elimination and readdition, to form the lactol **5**? Alternatively, would the cleavage of the C_1_–C_3_ bond predominate to provide the formation of **VI** that would, after a similar tandem β-H elimination and readdition sequence, give **VII** and then the lactol **6**?

The success of this challenging single-pot strategy, in which a diastereoselective catalytic reaction initiates a cascade of events, requires a good understanding of each elementary step listed above.

## Results and Discussion

**Scheme 4:**
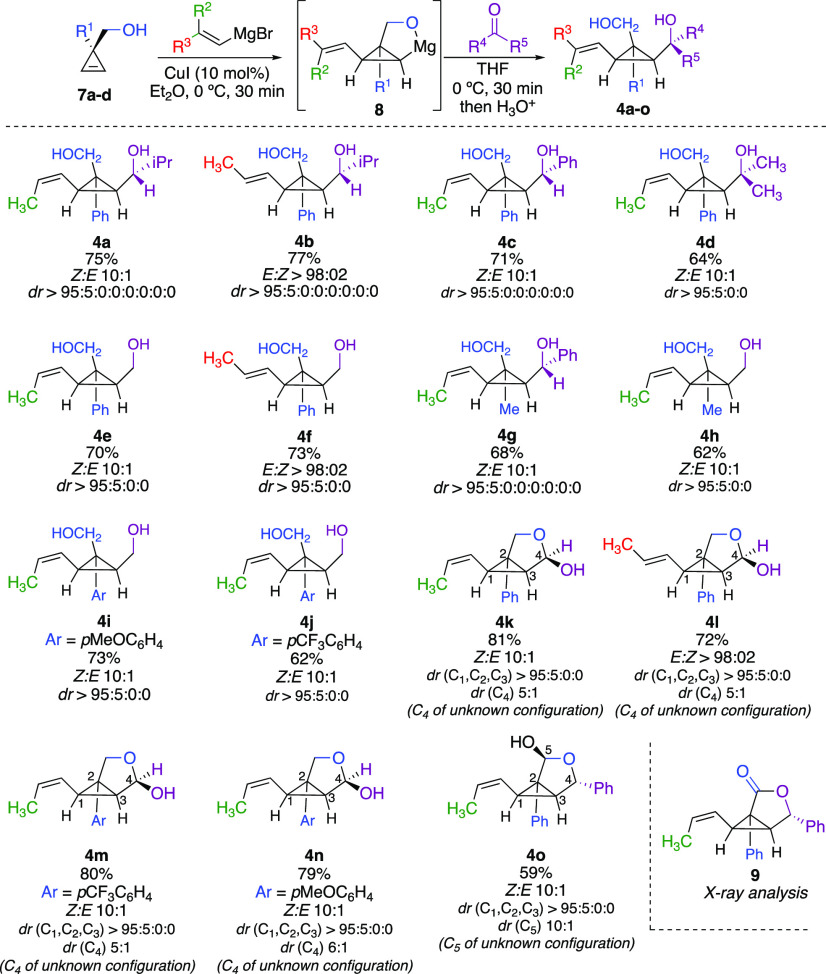
Preparation of *E*- and *Z*-Propenyl Cyclopropyl Diol Derivatives **4a**–**o**

We obviously had first to devise an efficient and practical route to these starting materials, and an extension of our recently reported diastereoselective carbometalation reaction of cyclopropenes could be strategically used as described in Scheme 4.^48−58^ The copper-catalyzed alkenylmagnesiation of easily accessible cyclopropene **7** (easily prepared through Rh-catalyzed decomposition of diazoesters in the presence of terminal alkynes and a subsequent DIBAL reduction, see Supporting Information), provided the *syn*-carbomagnesiated intermediate **8** that subsequently reacted with a large variety of carbonyl derivatives to provide the desired cyclopropyl diols **4** in very good overall yields. To our delight, the addition of aliphatic or aromatic aldehydes to **8** is completely diastereoselective, and **4a**–**c** were formed as a single diastereomer (Scheme 4).^59^ It should be noted that the imperfect *E:Z* ratios of the propenyl chains in **4a**, **4c**–**e**, **4g**–**k**, and **4m**–**4o** stem from the stereochemistry of the respective starting *Z*-propenyl Grignard reagents.^60^

**Scheme 5:**
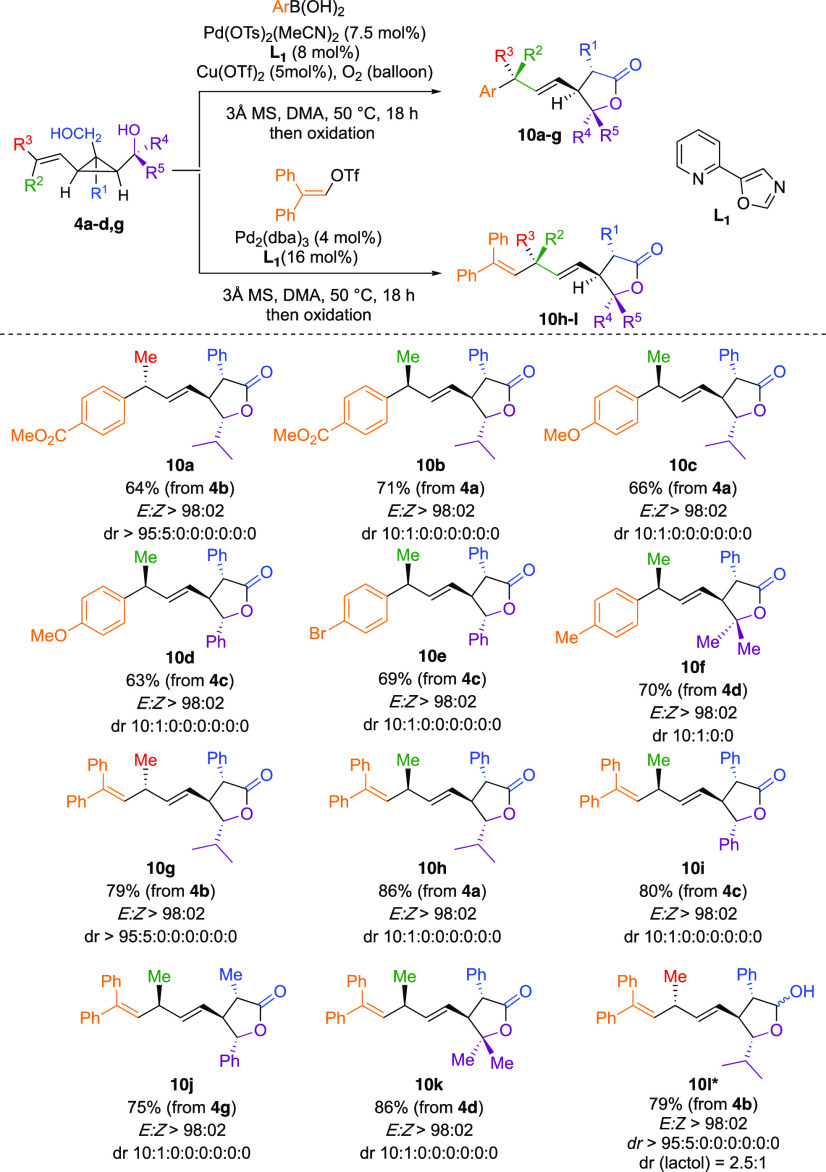
Regio- and Stereospecific Ring-Opening of Alkenyl Cyclopropyl Diols to Lactones Oxidation conditions: 1.5 equiv PIDA, 20 mol % TEMPO, DCM, rt. *Product **10l** was not oxidized.

On the other hand, compounds **4b**, **4f**, and **4l** were prepared by a tandem Cu-catalyzed addition of allylmagnesium bromide to **7**, addition of a carbonyl compound, followed by an isomerization of the terminal double bond into the unique *E*-isomer^61^ (see Supporting Information). Compounds **4i**, **4j**, and **4l** were prepared by NaBH_4_ reduction of the corresponding lactol precursor (see Supporting Information). The relative configuration was determined by X-ray analysis of lactone **9**,^62^ obtained by transformation of **4c** into **9** (see Supporting Information), and all other configurations were assigned by analogy. When DMF was added as electrophilic partner, lactols **4k**–**4n** were obtained as two epimers at C_4_ (dr at C_4_ for **4k**–**4m** of 5:1 and 6:1 for **4n**) of unknown configuration (Scheme 4). For the last example, the lactol **4o** was obtained by a diastereoselective reduction of lactone **9** (dr at C_5_ = 10:1). Having established a straightforward and diastereoselective route to cyclopropyl diol derivatives **4**, we set out to explore the selectivity of the tandem Heck addition–ring-opening reactions (Scheme 5). To easily analyze the formed products, an oxidation reaction of the resulting lactols into lactone was performed in all possible cases.^63^

When our model substrate **4b** was treated under our Pd-catalyzed addition of boronic acid, product **10a** was formed as a single regio- and diastereomer. The stereochemistry of the addition reaction was deduced from our previous research work on the Heck addition on cyclopropyl carbinol.^17,26^ Importantly, the complementary diastereomer was also accessible, with similar diastereoselectivity by simply inverting the stereochemistry of the starting propenyl cyclopropyl diol (**4a** produces **10b**). The transformation is stereospecific as the two starting propenyl cyclopropyl diols **4b** and **4a** are of *E:Z* ratios of >98:02 and 10:90 and provide the two lactone products with identical >98:02 and 90:10 diastereomeric ratios, respectively. Substitution pattern of the secondary alcohol does not impact the reaction outcome, as *iso*propyl, phenyl, and *gem*dimethyl groups gave similar results (see **10a**–**10f**, Scheme 5). Arylation reaction with electron-poor and electron-rich aryl groups (**10a**, **10b** versus **10c**, **10d**) proceeded equally well. By using slightly modified experimental conditions, the alkenylation reaction also proceeded in good overall yields with a complete stereospecificity (Scheme 5, **10g**–**10k**). Compound **10l** is the lactol product of the reaction before oxidation, obtained with a modest diastereomeric ratio of 1:2.5 at the anomeric position. Remarkably, the lactone products featuring up to four stereogenic centers are conveniently prepared in only two catalytic steps from achiral cyclopropenes **7**. In addition to its mechanistic implication, the preparation of densely functionalized stereodefined butyrolactones **10a**–**10k** is synthetically relevant because of the prevalence of this motif in natural products.^64^ Notable compounds possessing stereodefined polysubstituted lactones are (−)-phaseolinic acid,^65^ (−)-blastmycinolactol,^66^ and xanthane sesquiterpenoids, to cite a few.^67^

**Scheme 6:**
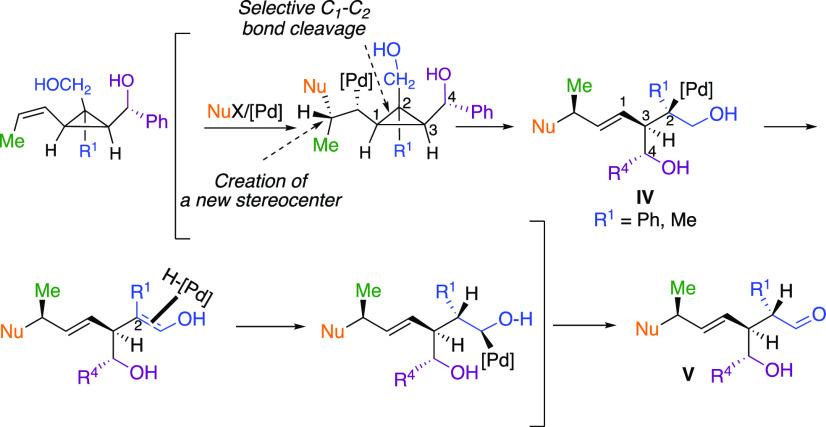
Selectivity of the Ring-Opening

From the two possible ring-opening scenarios originally discussed in Figure 1 and Scheme 3, the addition reaction is completely diastereoselective and undergoes a subsequent selective C_1_–C_2_ bond cleavage toward the formation of **IV**, even if the intermediate is a tertiary alkyl (R^1^ = Me, **10j**, Scheme 6) or aryl palladium species (R^1^ = Ph, **10a**–**10i** and **10k**, Scheme 6). In other words, the reaction proceeds toward the less substituted alcohol (selective cleavage of C_1_–C_2_) whatever is the degree of substitution at the C_2_ cyclopropyl ring. When the β-H elimination occurs from the stereodefined secondary alkyl palladium species **IV**, the formed alkenyl–H[Pd] complex undergoes an addition reaction from the same face, suggesting that the Pd does not disengage during the process and migrates on the same stereoface.

**Scheme 7:**
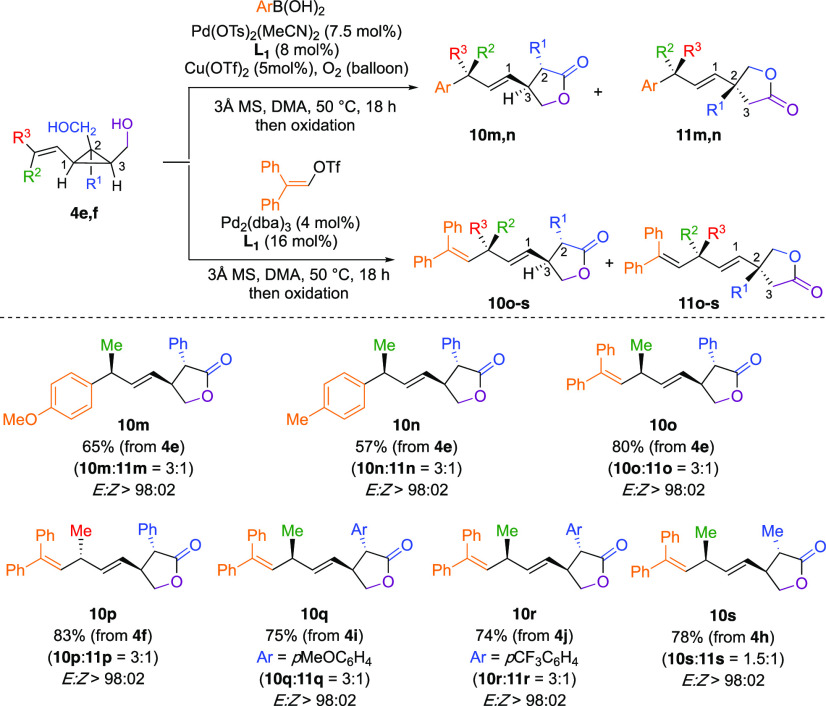
Ring-Opening of Primary Alkenyl Cyclopropyl Diols to Lactones Oxidation conditions: 1.5 equiv PIDA, 20 mol % TEMPO, DCM, rt.

To further gain additional insight on the selectivity of the ring-opening, we decided to investigate the selectivity of the C–C bond cleavage when two primary alcohols are concerned with a different degree of substitution at the cyclopropyl carbon centers (C_2_ tertiary versus C_3_ secondary, Scheme 7). Here again, the formed products are oxidized into lactones for an easier analysis of the NMR spectra and determination of diastereomeric ratios.

When **4e** (R^1^ = Ph) was engaged in the Pd-catalyzed addition of aryl boronic acid, lactones **10m** and **11m** were obtained in a 3:1 ratio, each one as a single diastereomer, suggesting that the C_1_–C_2_ bond cleavage still occurs predominantly toward the formation of the most substituted benzylic carbon–palladium center. However, a significant amount of C_1_–C_3_ ring cleavage was also produced (**11m** as minor product). The same holds for the Pd-catalyzed addition of vinyl triflate to **4e**, as **10o** and **11o** were obtained in a similar ratio (3:1, Scheme 7). The stereochemistry of the propenyl chain has no effect on the selectivity of the ring-opening, as the lactones **10p**:**11p** were obtained in identical ratio as that of **10o**:**11o** originating from *E*- or *Z*-propenyl cyclopropyl diols **4f** and **4e**, respectively. Variation of the electronic effect of the aryl substituent on C_2_ (electron donating or withdrawing substituent) does not affect the selectivity of the reaction (Scheme 7, formation of **10q** and **10r** as major products), indicating that the regioselectivity is not dictated by an electronic effect. Only when the substituent at C_2_ is a methyl group (R^1^ = Me, **10s**, Scheme 7), an almost unselective C–C bond cleavage occurs to provide the two regioisomers. Here again, from the two primary alcohol functionalities, the ring-opening occurs slightly more toward the most substituted cyclopropyl carbon center (C_2_ versus C_3_).

**Scheme 8:**
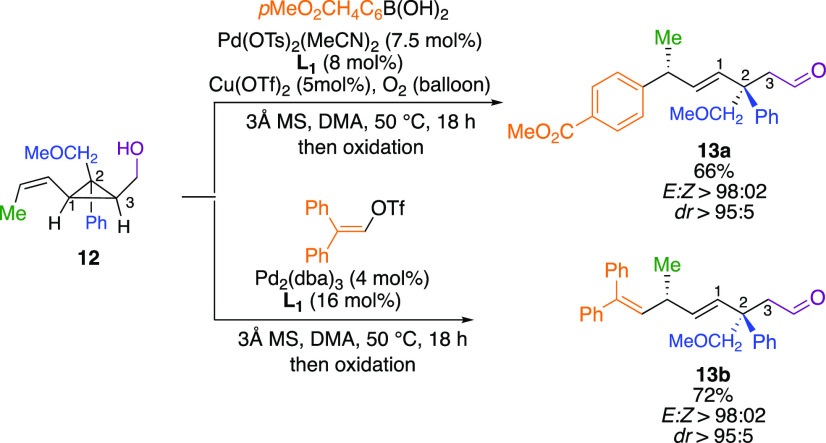
Reverse Selectivity for Mono-Protected Diol

However, if one selectively protects the primary alcohol on the most substituted carbon center as in **12** (Scheme 8), the opposite selectivity for the Heck addition and the following ring-opening reaction occurs—along the cyclopropyl C_3_ carbon center—to produce **13a** and **13b**, respectively, as unique isomers (Scheme 8). This points out that the presence of a free hydroxyl is mandatory to control the diastereoselectivity of the Heck-addition step, which, as we found (see the Supporting Information for computational results), subsequently defines the mode of ring-opening.

**Scheme 9:**
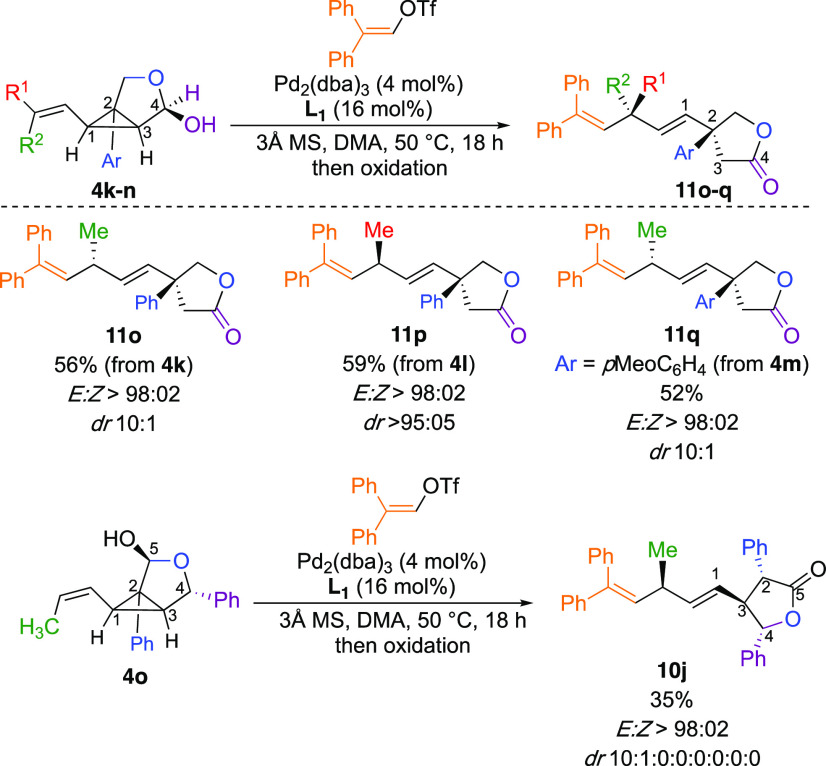
Selective Ring-Opening of Lactols

To further corroborate the primordial role of the free hydroxyl group on the selectivity of the carbon–carbon bond cleavage, we surmised that an alternative and easier approach to *in situ* prepare monoprotected diol analogs would be to use the lactols previously prepared (**4k**–**4n**, Scheme 4). When **4k** (R^1^ = H, R^2^ = Me, Ar = C_6_H_5_, *Z:E* = 10:1) was engaged in our Pd-catalyzed Heck-arylation–selective ring-opening reactions (Scheme 9), the only observed product was **11o** with the same diastereomeric ratio (dr 10:1) as the initial stereochemistry of the starting material **4k**. By permuting the stereochemistry of the initial double bond (**4l**, R^1^ = Me, R^2^ = H, Ar = C_6_H_5_, *E:Z* > 95:5), **11p** was obtained in excellent diastereomeric ratio (dr > 95:5, Scheme 9).

**Figure 2:**
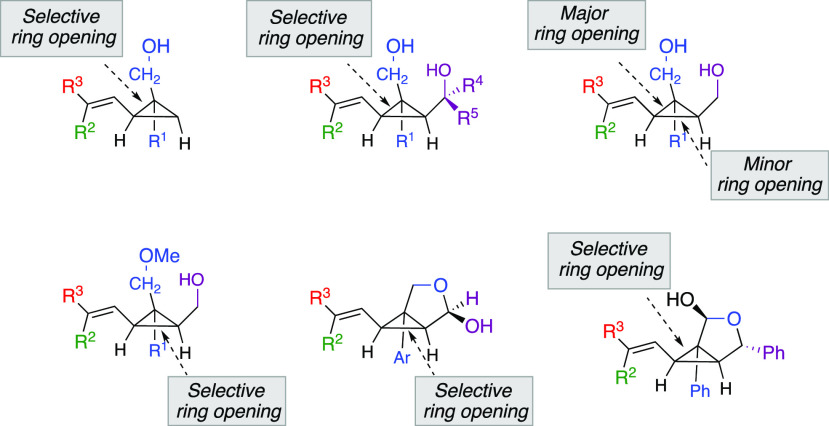
Summary for the selectivity of ring-opening of alkenyl cyclopropyl diol derivatives

Variation of the nature of the aryl substituent at the C_2_ position has no effect on the selectivity of the reaction (Ar = *p*MeOC_6_H_3_, **11q**, Scheme 9). Finally, by changing the position of the hydroxy group of the lactol, it was possible to reverse the regioselectivity of the ring-opening, as **4o** only provides **10j** (Scheme 9). Obviously, the rules governing the ring-opening are of a complex nature, as one could judge by the observed selectivity summarized in Figure 2.

**Scheme 10:**
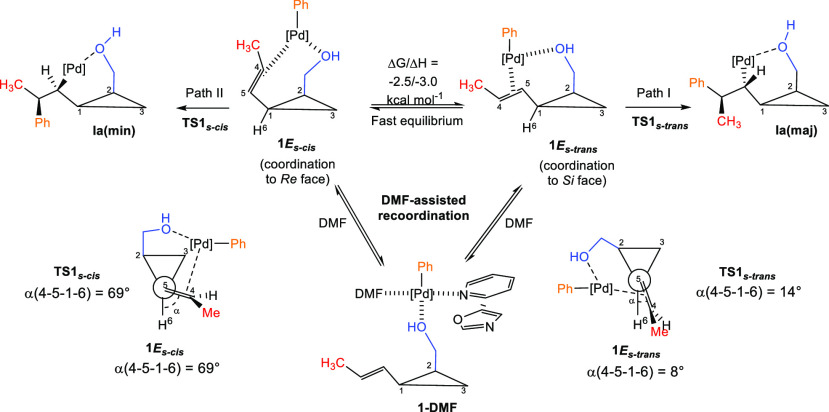
Diastereoselectivity in the Migratory Heck Insertion for the *E*-Isomer **1a**

**Figure 3:**
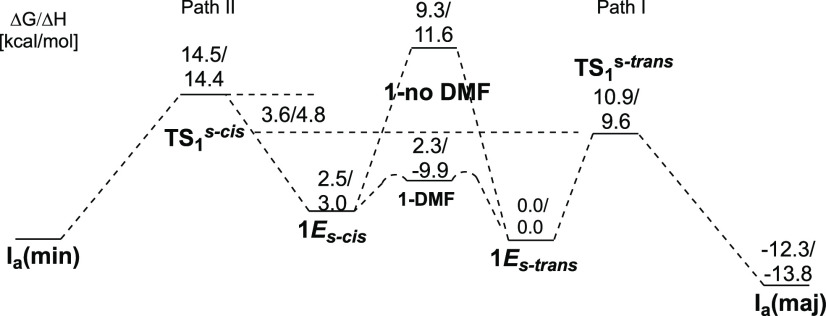
PES for the diastereoselective migratory Heck insertion for the *E*-isomer **1a**.

To shed some light on the selectivity of the carbon–carbon bond cleavage, the reaction mechanism was investigated by density functional theory (DFT) calculations, using Gaussian 09^68^ (see the Supporting Information for all computational details), initially on the simplest alkenyl cyclopropyl carbinol **1** and then on alkenyl cyclopropyl carbinol **4** (Figure 1). The alkenyl cyclopropyl carbinol **1** has several functionalities (cyclopropane σ-bonds, double bond, hydroxyl moiety) and an aryl group that can interact with the Pd center, generating many potential interactions and therefore many combinations. To address this challenge, we used the combined approach of DFT calculation with CREST, the code based on GFNn-xTB, recently developed by Grimme,^69^ to search the lowest energy states to build potential energy surfaces (see the Supporting Information). First, we examined the migratory insertion of the aryl group onto the alkenyl side chain of **1** (Scheme 10 and Figure 3). Depending on the mode of coordination of the double bond to the metal center in **1E**_**s-trans**_ and **1E**_**s-cis**_, the insertion produces **I**_**a**_**(maj)** and **I**_**a**_**(min)**, respectively. The carbopalladation reaction of **1E**_**s-trans**_, leading to the main product **I**_**a**_**(maj)**, is exergonic (Δ*G*/Δ*H* = −12.3/–13.8 kcal/mol) with a relatively low barrier Δ*G*^⧧^/Δ*H*^⧧^ = 10.9/9.6 kcal/mol (Scheme 10 and Figure 3, path I). Calculated free energy of decoordination of the double bond from Pd is 9.3 kcal/mol, and it decreases to 2.3 kcal/mol when assisted by a solvent molecule (DMF as a mimic of DMA), implying a fast equilibrium between **1E**_**s-trans**_ and **1E**_**s-cis**_ via the formation of an intermediate **1-DMF**. Due to this fast equilibrium and virtually irreversible following insertion step, the diastereoselectivity is controlled by relative energies of transition states **TS1**^**s-cis**^ versus **TS1**^**s-trans**^ (Curtin–Hammett principle). We found that the energy difference, Δ*G* (**TS1^s-cis^** – **TS1**^**s-trans**^), is 3.6 kcal/mol, corresponding to **I**_**a**_**(maj)**/**I**_**a**_**(min)** = 400/1 ratio (at 25 °C), which is in line with the experimentally observed diastereoselectivity. DFT studies revealed that stabilizing conjugation between the cyclopropyl and the double bond^70−72^ in **TS1**^**s-trans**^ is more effective than in **TS1**^**s-cis**^, due to the constraints caused by the O–Pd coordination, which is clearly observed in the respective Newman projection (Scheme 10, see Supporting Information for details).

Comparison of the barriers of insertion for the *Z*-isomer of **1a**, **1***Z*_**s-trans**_ and **1***Z*_**s-cis**_, led to very similar results: Δ*G*^⧧^ = 14.4 and 11.9 kcal/mol, respectively.

**Scheme 11:**
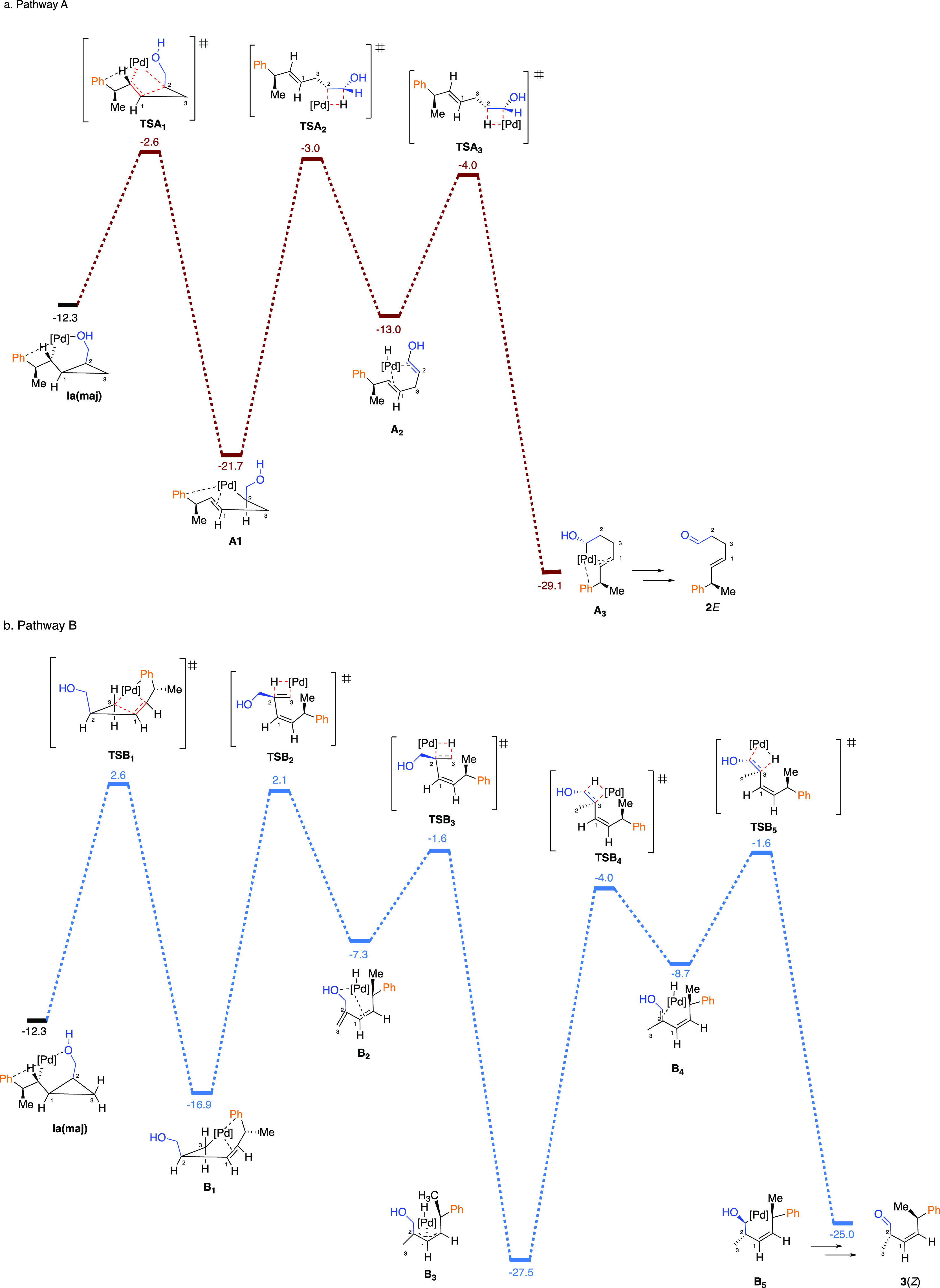
Comparison of the Energy Surfaces for the Two Competing Pathways for Selective Ring-Opening of Cyclopropanes All values of Δ*G* are in kcal/mol.

With a good understanding for the first diastereoselective addition step, we then turned our attention to the origin of the regioselectivity for the ring-opening of the cyclopropyl carbinol **1**. We investigated the potential energy surfaces for the two possible ring-opening pathways, namely, along C_1_–C_2_ and C_1_–C_3_ bonds (Scheme 11). It should be mentioned that Scheme 11 does not represent the complete potential energy surfaces, as few conformational changes between two consecutive transition states are omitted for the sake of clarity (for the whole PES, see the Supporting Information). Thus, starting from the major addition product **I**_**a**_**(maj)**, two pathways, **A** (red) and **B** (blue), were calculated, leading potentially to the two aldehydes **2***E* and **3***Z*, respectively. Both pathways relate to the selectivity of the ring-opening and subsequent β-H elimination and reinsertion steps, resulting in the formation of **A**_**3**_ and **B**_**5**_, precursors of **2***E* and **3***Z*. The last step has already been studied^73,74^ in detail for similar compounds and therefore is not discussed therein. Within both pathways, the energy decreases when the alkyl-palladium approaches the hydroxy-substituted position. This thermodynamic “sink” was already identified for similar reactions.^73,74^ Noteworthy, the PES of path A (initial cleavage along the C_1_–C_2_ bond) is lower than that of path B (initial cleavage along the C_1_–C_3_ bond) throughout the entire process, starting from the first transition state of ring-opening, where Δ*G*(**TSB**_**1**_ – **TSA**_**1**_) = 5.2 kcal/mol.

Assuming kinetic control, the difference of 5.2 kcal/mol explains well the complete selectivity of the C_1_–C_2_ bond cleavage observed experimentally for **1**. Within these studied paths, the highest activation free energies (rate-determining barriers) for both paths are accessible at room temperature Δ*G*^⧧^ (**A**_**1**_ → **TSA**_**2**_) = 18.7 kcal/mol and Δ*G*^⧧^ (**B**_**3**_ → **TSB**_**5**_) = 25.9 kcal/mol. Importantly, the competition between paths **A** and **B** is relevant only for substrates possessing hydrogen-substituted carbon C_2_. Any additional alkyl substitution on that position would require a β-alkyl elimination in **TSB**_**2**_ with an inaccessible barrier in the present experiment of more than 40 kcal/mol.

**Figure 4:**
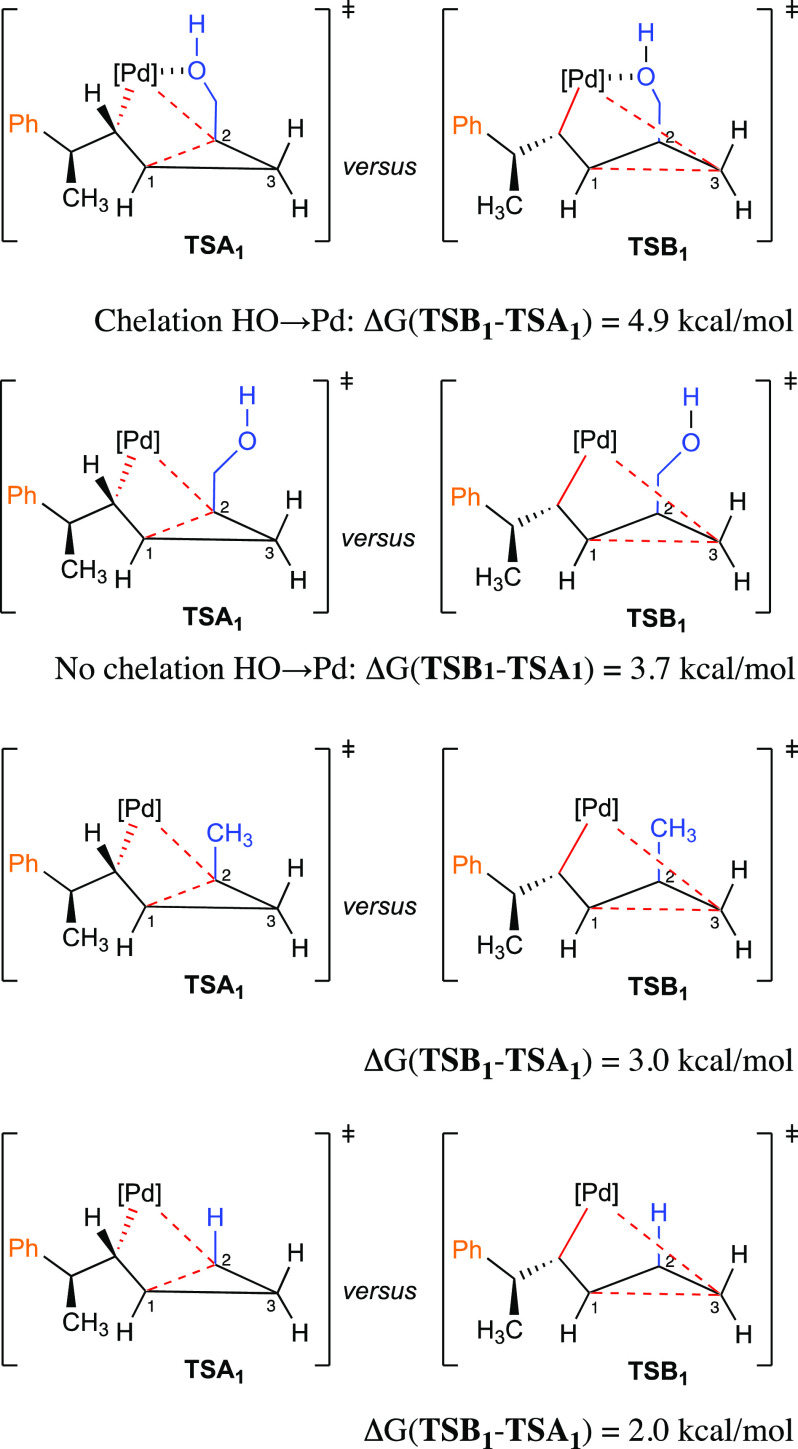
Calculated transition states for cyclopropane ring-opening and their relative energies.

**Scheme 12:**
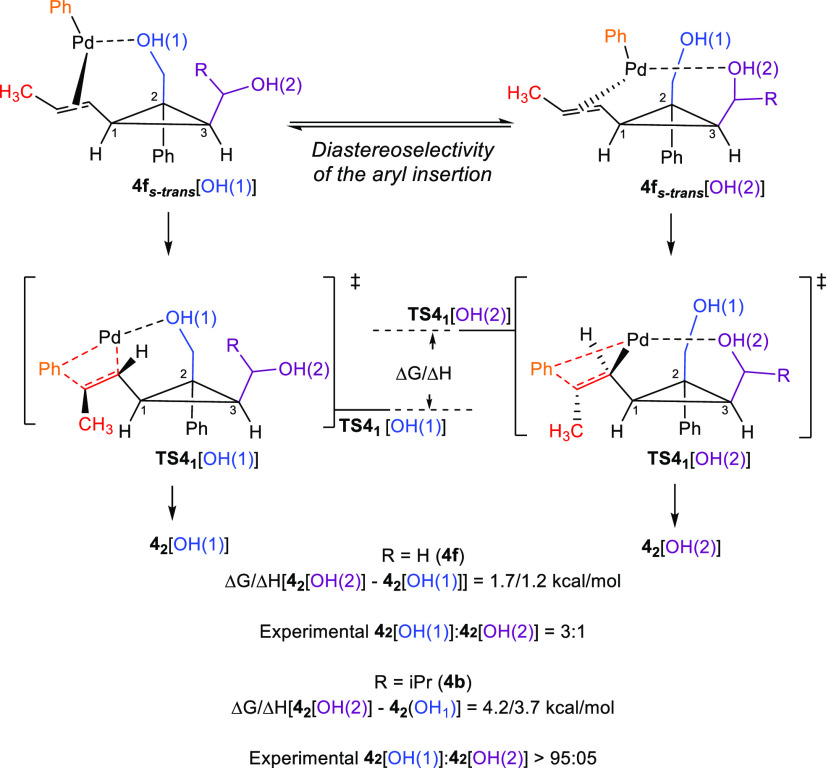
Regioselective Aryl Addition on Cyclopropyl Diols **4f** and **4b**

To further understand the origin of the regioselective ring-opening, we initially focused on the two transition-state structures of the ring-opening steps, namely **TSA**_**1**_ and **TSB**_**1**_. We have calculated and compared the energy differences between **TSB**_**1**_ and **TSA**_**1**_ (Δ*G*(**TSB**_**1**_ – **TSA**_**1**_)) by varying the nature and the effect of the substituent on C_2_ (Figure 4). The calculations were performed using Orca 4 software^75^ and resulted in Δ*G*(**TSB**_**1**_ – **TSA**_**1**_) = 4.9 kcal/mol, which is in a good agreement with the previous value (5.2 kcal/mol) found with Gaussian 09 using similar level of theory. Suppressing the oxygen–palladium coordination in **TSA**_**1**_ produces **unchelated TSA**_**1**_, with 1.2 kcal/mol. The loss of O → Pd stabilization in **TSA**_**1**_ reduces Δ*G*(**TSB**_**1**_ – **TSA**_**1**_) from 4.9 to 3.7 kcal/mol (Figure 4). Next, substitution of OH by H (R = Me on C_2_) in **TSA**_**1**_ destabilizes it further by 0.7 kcal/mol, decreasing the energy difference to 3.0 kcal/mol (Figure 4). The obtained destabilization of 0.7 kcal/mol is due to a higher inductive stabilization of the partial negative charge on the carbon holding CH_2_OH group (C_2_) in **TSA**_**1**_ relative to the inductive effect of Me in **TSA**_**1**_. Finally, when there is no substituent, Δ*G*(**TSB**_**1**_ – **TSA**_**1**_) is decreased further to 2.0 kcal/mol (Figure 4). The calculated 2.0 kcal/mol difference is the relative stability of *E*- versus *Z*-formation in **TSA**_**1**_ and **TSB**_**1**_, respectively. We found that the respective *E* and *Z* intermediates without Pd (where Pd is substituted by a hydrogen in the products of the ring-opening step) differ by 1.9 kcal/mol in favor of the *E*-configurated substrate (see Supporting Information), indicating that the Δ*G* of 2.0 kcal/mol between **TSA**_**1**_ and **TSB**_**1**_ is mainly an intrinsic property of the organic moieties in these complexes. All the results are consistent with the generally higher PES of path B relative to path A, through all subsequent steps, where the coordination Pd–O is out of play, but all other factors remain constant (Scheme 11). The results of this mechanistic computational study for cyclopropyl carbinol **1** are completely consistent with the observed selectivity of insertion and ring-opening reactions. After gaining these valuable insights, we turned our attention to cyclopropyl diol **4f**. Conformational analysis revealed that the conformers with the lowest energies for the insertion reaction to take place {**TS4**_**1**_ [OH(2)] and **TS4**_**1**_ [OH(1)]} have geometries possessing an effective conjugation between the electronic system of the cyclopropyl core with the alkenyl moiety. Therefore, contrary to carbinol **1**, the relative stability of the transition states mainly originates from their steric properties. Based on the Curtin–Hammett principle, the stereoselectivity of aryl insertion step for the diol **4f** (R = H), that is, the ratio of **4**_**2**_ [OH(1)]:**4**_**2**_ [OH(2)], results from the energy difference between their respective transition states Δ*G*{**TS4**_**1**_ [OH(2)] –**TS4**_**1**_ [OH(1)]}, which is 1.7 kcal/mol, being qualitatively in line with experimentally observed ratio of **4**_**2**_ [OH(1)]:**4**_**2**_ [OH(2)] = 3:1 (Schemes 5 and 12). When the substituent is Ph (**10m**–**10r**), the selectivity is higher than Me (**10s**) and could simply be attributed to a better stabilization of the Pd-intermediate (tertiary benzylic versus tertiary nonbenzylic) after the ring-opening.

**Scheme 13:**
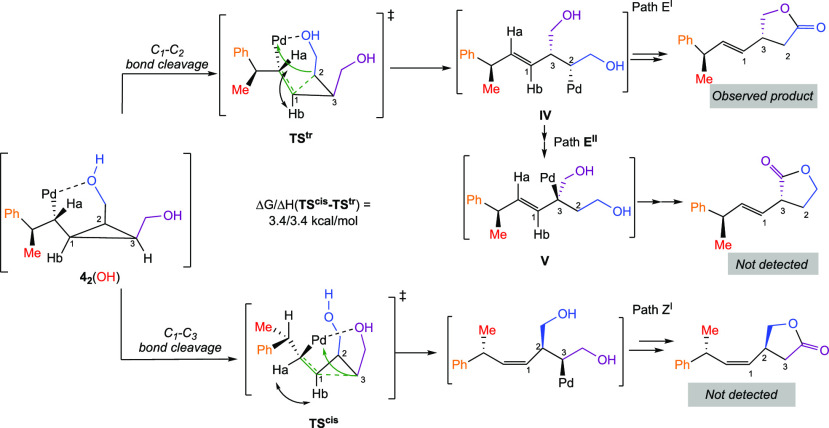
Stereochemistry Dictating the Ring-Opening

When more substituted cyclopropyl diol **4b** was used (R = *i*Pr, Schemes 5 and 12), a higher Δ*G*{**TS4**_**1**_ [OH(2)] –**TS4**_**1**_ [OH(1)]} = 4.2 kcal/mol is obtained, underlining the steric effect of the secondary alcohol on the diastereoselective aryl-addition on the alkenyl side chain of the cyclopropyl diol. The subsequent ring-opening from **4**_**2**_ [OH(1)] can occur along the C_1_–C_2_ or C_2_–C_3_ bond where, contrary to carbinol **1**, the only key difference is the energy required to lead to either the *E*- or *Z*-isomer. The cleavage of C_1_–C_2_ bond (leading to the *E*-isomer) proceeds through a transition state that is 3.4 kcal/mol lower than that of the C_1_–C_3_ bond cleavage (leading to the *Z*-isomer, Scheme 13). This difference, Δ*G*(**TS**^**cis**^ – **TS**^**trans**^) = 3.4 kcal/mol, is in line with the observed selectivity toward the unique formation of the *E*-isomer. It should also be noted that the sequence of β-H elimination and reinsertion on alkenyl-cyclopropyl-diol might lead to additional pathways. For instance, from the intermediate **IV**, the sequence could proceed toward the second hydroxyl (blue) producing intermediate **V** that finally led to the formation of the lactone through pathway **E**^**II**^ (Scheme 13).

## Conclusion

In conclusion, the Heck insertion reaction creates a new stereocenter leading to a configurationally stable carbon–palladium bond that controls the subsequent selectivity of the ring-opening. Due to steric constrains, an *E*-double bond represents the favored pathway, thus dictating the regioselectivity. As computational studies show, the ring-opening is in fact controlled by the diastereoselectivity of the first step, namely the migratory insertion step. In addition to the mechanistic implication, various lactones possessing up to four stereocenters as a single diastereomer were straightforwardly prepared in only two catalytic steps from easily accessible achiral cyclopropenyl carbinols **7**.

## Supporting Information Available

Experimental procedures, characterization data for all new compounds, along with copies of spectra, computational methods and data, and geometries of computed intermediates and transition states (PDF)
