# Supplementary material for: Mechanistic Insights on the Selectivity of the Tandem Heck–Ring-Opening of Cyclopropyldiol Derivatives
Source: JACS Au. 2022 Mar 5;2(3):687–96. doi: 10.1021/jacsau.1c00547 (PMC8970019; doi:10.1021/jacsau.1c00547)
Supplement: Supplementary file 1 — au1c00547_si_001.pdf [file au1c00547_si_001.pdf]

Supporting Information

Mechanistic Insights on the Selectivity of the Tandem Heck – Ring Opening of Cyclopropyldiol Derivatives

*Anthony Cohen, Alexander Kaushansky and Ilan Marek**

Schulich Faculty of Chemistry, Technion—Israel Institute of Technology,

Technion City, Haifa, 32000 (Israel)

*E-mail: chilanm@technion.ac.il

Table of Contents

Experimental procedures 4
General information 4
Procedures and data for new compounds 4
Procedures for the preparation of cyclopropenes 4
Procedure for the vinylmetalation of cyclopropenes (Procedure A) 5
Procedure for the allylmetalation of cyclopropenes (Procedure B) 6
Procedure for the isomerization of allyl cyclopropane (Procedure C) 6
Procedure for the Reduction of lactones to diols (Procedure D) 7
General procedure for the tandem Heck arylation/ vinyl cyclopropyl carbinols ring opening with arylboronic acids (Procedure E) 15
General procedure for the tandem Heck alkenylation/ vinyl cyclopropyl carbinols ring opening with alkenyltriflates (Procedure F) 15
Crystal data and structure refinement of compound 9 26
Computational Supporting Information 28
Computational details 28
High structural degree of freedom – conformational isomers challenge 28
Conformers of the pyridyl(oxazole) ligand 28
Overview of chelating modes of the ligand and alkenyl chain in the complex. 29
Conformational search and analysis 30
Recommendation - do not skip the optimizations! 34
Recommended workflow to obtain the best isomer 36
Calculated reaction mechanisms of carbinol 1 37
Migratory insertion. 37
Path A 41
Path B 43
Ring opening step and variation of the nature and effect of the R group on the reaction barrier.... 45
Reaction mechanisms of diols 46
Migratory insertion 46
Ring opening 46
References 47
NMR spectra of new compounds 49

List of XYZ coordinates (geometries) 121

Experimental procedures

General information

Unless stated otherwise, reactions were conducted in flame-dried glassware under a positive pressure of argon. Ether and THF were dried from Pure-Solv® Purification System (Innovative Technology©). [Pd(OTs)$_2$(MeCN)$_2$] was freshly prepared and stored under a dry atmosphere.[1] All other commercially available reagents were used as received. Thin-layer chromatography (TLC) was conducted with E. Merck silica gel 60 F254 pre-coated plates (0.25 mm) and visualized by exposure to UV light (254 nm) or stained with anisaldehyde, phosphomolybdic acid, or potassium permanganate solutions. Column chromatography was performed using Fluka silica gel 60 Å (40-63mm, 230-400 mesh). NMR spectra were recorded on Bruker spectrometer (AVIII400) and are reported relative to deuterated solvent signals. Chemical shifts are reported in parts per million (ppm) with respect to the residual solvent signal $CDCl_3$ (^{1}H NMR: δ = 7.26; ^{13}C NMR: δ = 77.16). Peak multiplicities are reported as follows: s = singlet, bs = broad singlet, d = doublet, t = triplet, dd = doublet of doublets, td = triplet of doublets, m = multiplet. High-resolution mass spectra (HRMS) were obtained by the mass spectrometry facility at the Technion. Reactions were monitored by gas chromatography spectrometry (GC) using an Agilent Technologies 7820A GC with an Agilent Technologies 19091J-413 (30 m × 0.3 mm) column.

Procedures and data for new compounds

Procedures for the preparation of cyclopropenes

Cyclopropenyl carbinols were prepared through the rhodium catalyzed decomposition of diazo esters in the presence of terminal alkynes, followed by reduction of the ester using DIBAL-H. Cyclopropene 7a[2] and 7b, 7c[3] ,7d[4] and 7e[5] were synthesized according to previously reported protocols. The compounds were isolated and characterized; the experimental results are in good agreement with the literature reports.

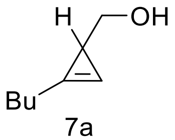

7a

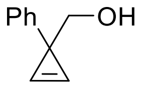

7b

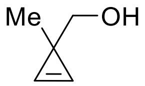

7c

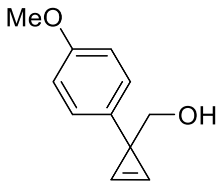

7d

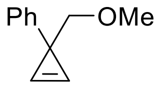

7e

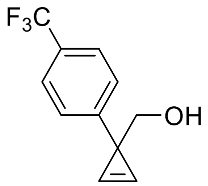

(1-(4-(Trifluoromethyl)phenyl)cycloprop-2-en-1-yl)methanol 7f

The compound was prepared according to a literature protocol.[3]

^{1}H NMR (400 MHz, $CDCl_3$) δ 7.33 (t, *J* = 11.8 Hz, 2H), 7.26 (t, *J* = 7.5 Hz, 2H), 7.19 – 7.13 (m, 1H), 5.71 – 5.56 (m, 1H), 5.31 (t, *J* = 9.5 Hz, 1H), 4.03 – 3.88 (m, 2H), 3.80 (t, *J* = 12.4 Hz, 1H), 3.68 (td, *J* = 11.4, 6.7 Hz, 1H), 2.99 (s, 1H), 2.45 (s, 1H), 2.05 (t, *J* = 8.8 Hz, 1H), 1.90 – 1.81 (m, 1H), 1.67 (d, *J* = 7.0 Hz, 3H). ^{13}C NMR (101 MHz, $CDCl_3$) δ 145.01, 129.36, 129.29, 128.69, 128.61, 128.55, 126.90, 123.41, 63.86, 59.64, 36.99, 30.17, 26.87, 13.49. HRMS m/z: (APCI) $[M-OH]^+$, calculated for $C_{11}H_8F_3$; 197.0573; found 197.0593.

Procedure for the vinylmetalation of cyclopropenes (Procedure A)

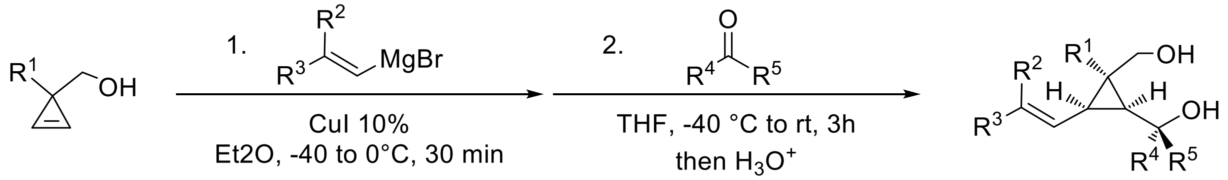

Alkenyl magnesium bromide derivatives were freshly prepared prior to the reaction through the following protocol: A flamed-dried three-neck flask equipped with a condenser under argon atmosphere, was charged with magnesium turnings (1.4 eq.) and a solution of alkenyl bromide (1eq., 1M in THF) was added slowly to the flask while vigorously stirring. The solution was refluxed for 1 hour. The concentration of the Grignard reagent was determined by standard titration with I_2 in THF and ranged between 0.5 and 0.6M. To a flamed-dried three-neck flask equipped with a thermometer under argon atmosphere were added copper iodide (10 mol %, 95 mg), the suitable cyclopropenyl methanol compound (5 mmol) and diethyl ether (0.1 M, 50 ml). The suspension was stirred vigorously and cooled to -40 °C using an acetone/dry ice cooling bath. The alkenyl magnesium bromide solution (22 ml, 0.5M in THF, 2.2 eq.) was added dropwise while maintaining the temperature below -30 °C. The cooling bath was then removed, and the solution was let to warm up to 0 °C. Upon completion of the reaction as monitored by TLC (usually after 30 min), a cooling bath was placed and the reaction mixture was cooled to -40 °C. A solution of the suitable carbonyl compound (20 ml, 0.5 M in THF, 2 eq.) was then added and the solution was let to warm up to 0 °C in an ice bath over 3 hours. Following the complete conversion of the cyclopropene (conversion monitored by TLC and/or GC), the reaction was hydrolyzed with a saturated solution of ammonium chloride and the aqueous phase was extracted with Et_2O three times. The combined organic phases were dried over anhydrous $MgSO_4$ and concentrated under reduced pressure. The resulting crude mixture was purified by column chromatography.

Procedure for the allylmetalation of cyclopropenes (Procedure B)

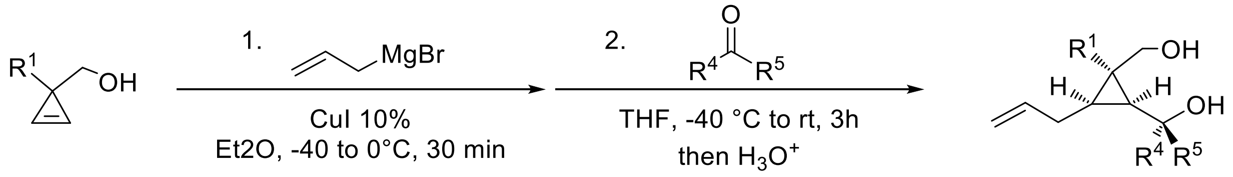

To a flamed-dried three-neck flask equipped with a thermometer under argon atmosphere were added copper iodide (10 mol %, 95 mg), the suitable cyclopropenyl methanol compound (5 mmol) and diethyl ether (0.1 M, 50 ml). The suspension was stirred vigorously and cooled to -40 °C using an acetone/dry ice cooling bath. Allyl magnesium bromide solution (11 ml, 1M in Et_2O, 2.2 eq.) was added dropwise while maintaining the temperature below -30 °C. The cooling bath was then removed, and the solution was let to warm to 0 °C. Upon completion of the reaction as monitored by TLC (usually after 30 min), a cooling bath was placed and the reaction mixture was cooled to -40 °C. A solution of the suitable carbonyl compound (20 ml, 0.1 M in THF, 2 eq.) was then added and the solution was let to warm up to 0 °C in an ice bath over 3 hours. Following the complete conversion of the cyclopropene (conversion monitored by TLC and/or GC), the reaction was hydrolyzed with a saturated solution of ammonium chloride and the aqueous phase was extracted with Et_2O three times. The combined organic phases were dried over anhydrous $MgSO_4$ and concentrated under reduced pressure. The resulting crude mixture was purified by column chromatography.

Procedure for the isomerization of allyl cyclopropane (Procedure C)

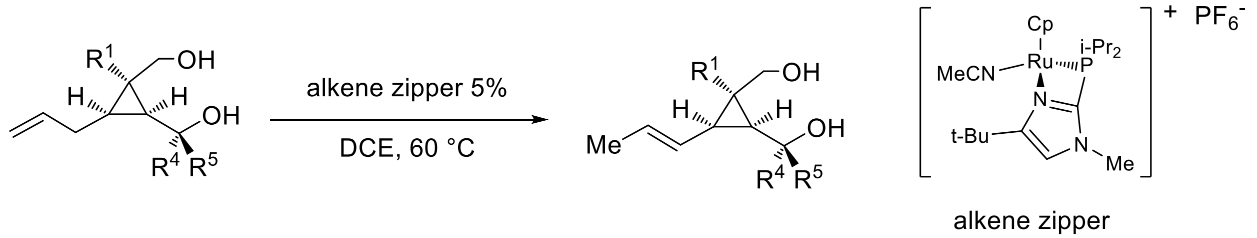

In a flamed-dried one-neck flask under argon atmosphere, the alkene zipper[6] (5 mol%, CAUTION: the catalyst is air and moisture sensitive and thus should be handled in an inert atmosphere) was dissolved in a solution of the corresponding allyl cyclopropyl diol (1 mmol, 1 equiv.) in dry 1,2-dichloroethane (10 mL/mmol substrate) at 50°C and monitored by TLC. Upon completion of the isomerization reaction (c.a. 24 h), the solvent was evaporated, and the reaction mixture was purified through a column chromatography to afford the pure desired product.

Procedure for the Reduction of lactones to diols (Procedure D)

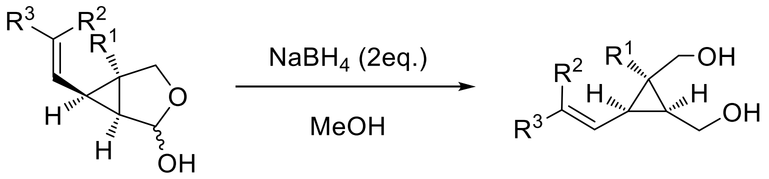

The cyclopropyl lactol (1 mmol) was dissolved in methanol (10 ml, 0.1M) in a one neck flask equipped with a magnetic stirrer. The solution was cooled to 0 °C and sodium borohydride (76 mg, 2 eq.) was added in one portion. Upon completion (as monitored by TLC), the reaction was hydrolyzed with 10 ml of HCl 1M and diluted with Et_2O. The aqueous phase was extracted with Et_2O three times, and the combined organic phases were dried over anhydrous $MgSO_4$ and concentrated under reduced pressure. The resulting crude mixture was purified by column chromatography.

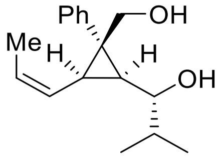

(R*)-1-((1S*,2R*,3R*)-2-(Hydroxymethyl)-2-phenyl-3-((Z)-prop-1-en-1-yl)cyclopropyl)-2-methylpropan-1-ol (4a)

Prepared from **7b** and freshly distilled isobutyraldehyde (2 eq.) as carbonyl electrophile by procedure A.

^{1}H NMR (400 MHz, $CDCl_3$) δ 7.42 (dt, *J* = 3.0, 1.8 Hz, 2H), 7.36 – 7.29 (m, 2H), 7.23 (ddd, *J* = 5.7, 4.3, 2.5 Hz, 1H), 5.67 (dqd, *J* = 10.7, 6.8, 1.0 Hz, 1H), 5.47 – 5.31 (m, 1H), 3.99 (d, *J* = 11.9 Hz, 1H), 3.88 (d, *J* = 12.0 Hz, 1H), 3.82 (t, *J* = 6.7 Hz, 1H), 3.56 (dd, *J* = 10.5, 6.3 Hz, 1H), 2.16 (t, *J* = 9.4 Hz, 1H), 1.81 (dt, *J* = 13.5, 6.7 Hz, 1H), 1.74 (dd, *J* = 6.9, 1.7 Hz, 3H), 1.03 (d, *J* = 6.7 Hz, 3H), 0.98 (d, *J* = 6.9 Hz, 3H). ^{13}C NMR (101 MHz, $CDCl_3$) δ 145.27, 129.37, 128.52, 127.46, 126.79, 124.32, 73.45, 64.20, 36.41, 34.60, 33.55, 27.62, 18.72, 18.27, 13.26. HRMS m/z: (APCI) $[M-OH]^+$, calculated for $C_{17}H_{23}O$; 243.1749; found 243.1750.

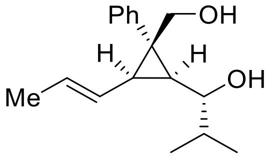

(R*)-1-((1S*,2R*,3R*)-2-(Hydroxymethyl)-2-phenyl-3-((E)-prop-1-en-1-yl)cyclopropyl)-2-methylpropan-1-ol (4b) containing 10% of unreacted starting material (SI-1)

Prepared from SI-1 by procedure C.

^{1}H NMR (400 MHz, $CDCl_3$) δ 7.38 (dt, *J* = 3.1, 1.8 Hz, 2H), 7.33 – 7.29 (m, 2H), 7.22 (ddd, *J* = 7.3, 3.9, 1.3 Hz, 1H), 5.74 – 5.60 (m, 1H), 5.46 – 5.32 (m, 1H), 3.99 (d, *J* = 11.9 Hz, 1H), 3.88 (d, *J* = 11.9 Hz, 1H), 3.53 (dd, *J* = 10.5, 6.4 Hz, 1H), 1.95 (t, *J* = 9.4 Hz, 1H), 1.82 (dq, *J* = 13.4, 6.7 Hz, 1H), 1.74 (dd, *J* = 6.5, 1.5 Hz, 3H), 1.62 (t, *J* = 9.8 Hz, 1H), 1.02 (d, *J* = 6.7 Hz, 3H), 0.96 (d, *J* = 6.9 Hz, 3H). ^{13}C NMR (101 MHz, $CDCl_3$) δ 145.42, 129.31, 128.44, 128.42, 126.67, 125.47, 73.67, 64.21, 35.94, 34.75, 33.59, 32.57, 18.84, 18.53, 18.35. HRMS m/z: (APCI) [M-OH]$^+$, calculated for $C_{17}H_{23}O$; 243.1743; found 243.1758.

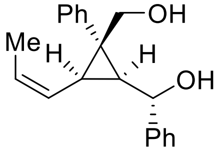

(*S)-((1*S**,2R*,3R)-2-(Hydroxymethyl)-2-phenyl-3-((Z)-prop-1-en-1-yl)cyclopropyl)(phenyl)methanol (4c)**

Prepared from **7b** and freshly distilled benzaldehyde (2 eq.) as carbonyl electrophile by procedure A.

^{1}H NMR (400 MHz, $CDCl_3$) δ 7.51 – 7.44 (m, 4H), 7.38 – 7.34 (m, 4H), 7.32 – 7.24 (m, 3H), 5.83 – 5.70 (m, 1H), 5.60 – 5.49 (m, 1H), 4.83 (d, *J* = 10.5 Hz, 1H), 4.18 (s, 1H), 4.02 (q, *J* = 12.0 Hz, 2H), 3.22 (s, 1H), 2.17 (t, *J* = 9.1 Hz, 1H), 2.02 – 1.95 (m, 1H), 1.79 (dd, *J* = 6.4, 1.3 Hz, 1H), 1.69 (dd, *J* = 6.9, 1.6 Hz, 3H). ^{13}C NMR (101 MHz, $CDCl_3$) δ 144.83, 143.17, 129.16, 128.58, 128.48, 128.38, 127.43, 126.77, 125.87, 123.58, 70.61, 64.07, 37.23, 36.71, 27.32, 13.37. HRMS m/z: (APCI) [M-OH]$^+$, calculated for $C_{20}H_{21}O$; 277.1592; found 277.1597.

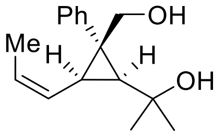

2-((1*S,2R*,3R*)-2-(Hydroxymethyl)-2-phenyl-3-((Z)-prop-1-en-1-yl)cyclopropyl)propan-2-ol (4d)**

Prepared from **7b** and freshly distilled acetone (5 eq.) as carbonyl electrophile by procedure A.

^{1}H NMR (400 MHz, $CDCl_3$) δ 7.31 (d, *J* = 7.7 Hz, 2H), 7.25 (t, *J* = 7.5 Hz, 2H), 7.16 (dd, *J* = 13.4, 6.0 Hz, 1H), 5.87 (t, *J* = 10.1 Hz, 1H), 5.63 (dq, *J* = 13.5, 6.8 Hz, 1H), 4.15 (s, 2H), 2.25 (s, 1H), 2.12 (t, *J* = 9.5 Hz, 1H), 1.86 (s, 1H), 1.71 (d, *J* = 6.8 Hz, 3H), 1.51 (s, 3H), 1.36 (d, *J* = 9.6 Hz, 1H), 1.31 (s, 3H). ^{13}C NMR (101 MHz, $CDCl_3$) δ 147.15, 129.10, 128.68, 126.74, 126.71, 125.56, 70.92, 63.89, 39.94, 38.07, 32.71, 30.60, 26.11, 13.36. HRMS m/z: (APCI) [M-OH]$^+$, calculated for $C_{16}H_{21}O$; 229.1592; found 229.1587.

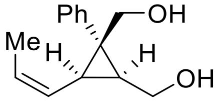

((1R*,2S*,3R*)-1-Phenyl-3-((Z)-prop-1-en-1-yl)cyclopropane-1,2-diyl)dimethanol (4e)

Prepared from **7b** and dry DMF (5 eq.) as carbonyl electrophile by procedure A followed by reduction with procedure D.

^{1}H NMR (400 MHz, $CDCl_3$) δ 7.33 (t, *J* = 11.8 Hz, 2H), 7.26 (t, *J* = 7.5 Hz, 2H), 7.19 – 7.13 (m, 1H), 5.71 – 5.56 (m, 1H), 5.31 (t, *J* = 9.5 Hz, 1H), 4.03 – 3.88 (m, 2H), 3.80 (t, *J* = 12.4 Hz, 1H), 3.68 (td, *J* = 11.4, 6.7 Hz, 1H), 2.99 (s, 1H), 2.45 (s, 1H), 2.05 (t, *J* = 8.8 Hz, 1H), 1.90 – 1.81 (m, 1H), 1.67 (d, *J* = 7.0 Hz, 3H). ^{13}C NMR (101 MHz, $CDCl_3$) δ 145.01, 129.36, 129.29, 128.69, 128.61, 128.55, 126.90, 123.41, 63.86, 59.64, 36.99, 30.17, 26.87, 13.49. HRMS m/z: (APCI) $[M\text{-}OH]^+$, calculated for $C_{14}H_{17}O$; 201.1279; found 201.1272.

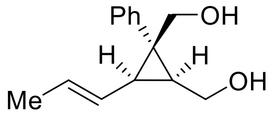

((1R*,2S*,3R*)-1-Phenyl-3-((E)-prop-1-en-1-yl)cyclopropane-1,2-diyl)dimethanol (4f)

Prepared from SI-2 by procedure C followed by reduction with procedure D.

^{1}H NMR (400 MHz, $CDCl_3$) δ 7.31 (d, *J* = 7.6 Hz, 2H), 7.25 (t, *J* = 7.5 Hz, 2H), 7.17 (dd, *J* = 12.0, 4.4 Hz, 1H), 5.64 (dq, *J* = 13.0, 6.4 Hz, 1H), 5.34 (dd, *J* = 15.0, 8.8 Hz, 1H), 4.02 – 3.90 (m, 2H), 3.84 (t, *J* = 13.2 Hz, 1H), 3.70 (t, *J* = 11.5 Hz, 1H), 2.97 (s, 1H), 2.41 (s, 1H), 1.91 (t, *J* = 9.0 Hz, 1H), 1.85 – 1.76 (m, 1H), 1.65 (d, *J* = 6.4 Hz, 4H). ^{13}C NMR (101 MHz, $CDCl_3$) δ 144.96, 129.37, 129.34, 128.62, 126.91, 124.44, 64.01, 59.81, 36.87, 31.61, 30.38, 18.40. HRMS m/z: (APCI) $[M\text{-}OH]^+$, calculated for $C_{14}H_{17}O$; 201.1279; found 201.1255.

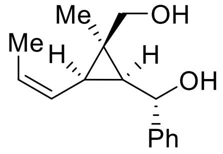

(S*)-((1S*,2R*,3R*)-2-(Hydroxymethyl)-2-methyl-3-((Z)-prop-1-en-1-yl)cyclopropyl)(phenyl)methanol (4g)

Prepared from **7c** and freshly distilled benzaldehyde (2 eq.) as carbonyl electrophile by procedure A.

^{1}H NMR (400 MHz, CDCl3) δ 7.34 (d, J = 7.2 Hz, 2H), 7.27 (dd, J = 10.0, 4.7 Hz, 2H), 7.21 – 7.17 (m, 1H), 5.56 (dqd, J = 13.5, 6.8, 1.3 Hz, 1H), 5.33 (ddd, J = 10.6, 8.8, 1.7 Hz, 1H), 4.61 (d, J = 10.5 Hz, 1H), 3.76 (dd, J = 23.8, 11.7 Hz, 2H), 1.63 (dd, J = 10.8, 6.4 Hz, 1H), 1.52 (dd, J = 6.8, 1.5 Hz, 3H). ^{13}C NMR (101 MHz, CDCl3) δ 143.74, 128.48, 128.03, 127.52, 125.79, 124.18, 71.58, 64.20, 37.48, 27.61, 27.12, 24.53, 13.35. HRMS m/z: (APCI) $[M\text{-}H_3]^+$, calculated for $C_{15}H_{17}O2$; 229.1229; found 229.1202.

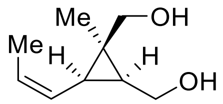

((1R*,2S*,3R*)-1-Methyl-3-((Z)-prop-1-en-1-yl)cyclopropane-1,2-diyl)dimethanol (4h)

Prepared from **7c** and dry DMF (5 eq.) as carbonyl electrophile by procedure A followed by reduction with procedure D.

^{1}H NMR (400 MHz, $CDCl_3$) δ 5.58 – 5.44 (m, 1H), 5.23 – 5.11 (m, 1H), 3.88 (dd, *J* = 11.8, 6.0 Hz, 1H), 3.70 – 3.55 (m, 2H), 3.51 (dd, *J* = 19.0, 7.7 Hz, 1H), 2.99 (s, 2H), 1.62 – 1.58 (m, 3H), 1.56 (d, *J* = 8.6 Hz, 1H), 1.28 – 1.22 (m, 1H), 1.21 (s, 3H). ^{13}C NMR (101 MHz, $CDCl_3$) δ 128.01, 124.03, 77.48, 77.16, 76.84, 63.75, 60.13, 30.87, 27.05, 26.64, 24.49, 13.41. HRMS m/z: (APCI) [M-OH]$^+$, calculated for $C_9H_{15}O$; 139.1123; found 139.1121.

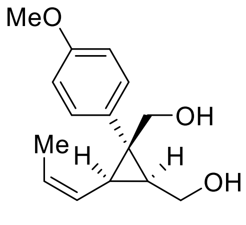

((1R*,2S*,3R*)-1-(4-Methoxyphenyl)-3-((Z)-prop-1-en-1-yl)cyclopropane-1,2-diyl)dimethanol (4i)

Prepared from **7d** and dry DMF (5 eq.) as carbonyl electrophile by procedure A followed by reduction with procedure D.

^{1}H NMR (400 MHz, $CDCl_3$) δ 7.36 – 7.31 (m, 2H), 6.89 – 6.83 (m, 2H), 5.75 – 5.63 (m, 1H), 5.43 – 5.30 (m, 1H), 4.05 – 3.91 (m, 2H), 3.82 (s, 1H), 3.79 (s, 3H), 3.70 (t, *J* = 11.5 Hz, 1H), 3.28 (s, 1H), 2.75 (s, 1H), 2.06 (t, *J* = 8.7 Hz, 1H), 1.92 – 1.81 (m, 1H), 1.74 (dd, *J* = 6.8, 1.3 Hz, 3H). ^{13}C NMR (101 MHz, $CDCl_3$) δ 158.50, 137.19, 130.49, 128.51, 123.52, 113.96, 64.05, 59.67, 55.40, 36.30, 30.22, 26.87, 13.46. HRMS m/z: (APCI) [M-H]$^+$, calculated for $C_{15}H_{19}O_3$; 247.1329; found 247.1341.

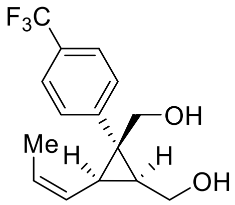

((1R*,2S*,3R*)-3-((Z)-Prop-1-en-1-yl)-1-(4-(trifluoromethyl)phenyl)cyclopropane-1,2-diyl)dimethanol (4j)

Prepared from **7f** and dry DMF (5 eq.) as carbonyl electrophile by procedure A followed by reduction with procedure D.

^{1}H NMR (400 MHz, $CDCl_3$) δ 7.56 (d, *J* = 8.2 Hz, 2H), 7.50 (d, *J* = 8.1 Hz, 2H), 5.72 (dqd, *J* = 10.6, 6.8, 1.5 Hz, 1H), 5.34 (ddq, *J* = 11.7, 8.2, 1.7 Hz, 1H), 4.06 – 3.92 (m, 2H), 3.84 (d, *J* = 12.1 Hz, 1H), 3.72 – 3.65 (m, 1H), 3.62 (s, 1H), 3.36 (s, 1H), 2.11 (dd, *J* = 12.9, 4.4 Hz, 1H), 1.92 – 1.81 (m, 1H), 1.72 (dd, *J* = 6.9,

1.2 Hz, 3H). ^{13}C NMR (101 MHz, $CDCl_3$) δ 149.21, 149.20, 129.49, 129.35, 129.11, 128.79, 125.49, 125.45, 125.42, 125.38, 122.70, 63.24, 59.39, 36.48, 30.29, 27.09, 13.49. HRMS m/z: (APCI) [M-OH]$^+$, calculated for $C_{15}H_{16}F_3O$; 269.1148; found 269.1160.

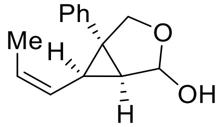

(1S*,5R*,6R*)-5-Phenyl-6-((Z)-prop-1-en-1-yl)-3-oxabicyclo[3.1.0]hexan-2-ol (4k)

Prepared from **7c** and dry DMF (5 eq.) as carbonyl electrophile by procedure A. dr (lactol 4:1)

^1H NMR (400 MHz, $CDCl_3$) δ 7.26 – 7.19 (m, 2H), 7.17 – 7.11 (m, 3H), 5.63 (qdd, *J* = 10.8, 5.8, 2.4 Hz, 1H), 5.39 – 5.32 (m, 1H), 5.30 (s, 1H), 4.25 (dd, *J* = 8.4, 4.5 Hz, 1H), 4.01 (d, *J* = 8.4 Hz, 1H), 3.79 – 3.64 (m, 1H), 2.14 (d, *J* = 8.5 Hz, 1H), 2.04 (t, *J* = 8.3 Hz, 1H), 1.63 (dd, *J* = 6.8, 1.7 Hz, 3H). ^{13}C NMR (101 MHz, $CDCl_3$) δ 139.84, 128.65, 128.23, 127.64, 126.77, 122.62, 98.52, 70.34, 37.35, 35.43, 26.25, 13.49. HRMS m/z: (APCI) [M-OH]$^+$, calculated for $C_{14}H_{15}O$; 199.1117; found 199.1134.

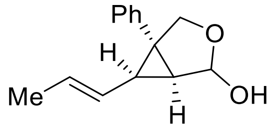

(1S*,5R*,6R*)-5-Phenyl-6-((E)-prop-1-en-1-yl)-3-oxabicyclo[3.1.0]hexan-2-ol

Prepared from SI-2 by procedure C.

^1H NMR (400 MHz, $CDCl_3$) δ 7.35 – 7.27 (m, 2H), 7.26 – 7.15 (m, 3H), 5.81 – 5.68 (m, 1H), 5.53 (ddq, *J* = 15.2, 8.9, 1.5 Hz, 1H), 5.43 (d, *J* = 4.2 Hz, 1H), 4.35 (d, *J* = 8.4 Hz, 1H), 4.17 (d, *J* = 8.4 Hz, 1H), 3.01 (d, *J* = 4.6 Hz, 1H), 2.18 (d, *J* = 8.5 Hz, 1H), 1.99 (t, *J* = 8.7 Hz, 1H), 1.73 (dd, *J* = 6.4, 1.5 Hz, 3H). ^{13}C NMR (101 MHz, $CDCl_3$) δ 139.88, 129.46, 128.66, 127.63, 126.77, 123.91, 98.79, 70.72, 37.44, 35.98, 31.37, 18.43. HRMS m/z: (APCI) [M-OH]$^+$, calculated for $C_{14}H_{15}O$; 199.1117; found 199.1133.

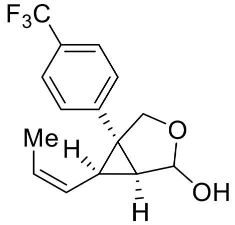

(1S*,5R*,6R*)-6-((Z)-Prop-1-en-1-yl)-5-(4-(trifluoromethyl)phenyl)-3-oxabicyclo[3.1.0]hexan-2-ol (4m)

Prepared from **7f** and dry DMF (5 eq.) as carbonyl electrophile by procedure A. dr (lactol 6:1)

^{1}H NMR (400 MHz, $CDCl_3$) δ 7.57 (d, *J* = 8.1 Hz, 2H), 7.32 (d, *J* = 8.0 Hz, 2H), 5.73 (dqd, *J* = 10.9, 6.8, 1.4 Hz, 1H), 5.48 – 5.31 (m, 2H), 4.32 (d, *J* = 8.4 Hz, 1H), 4.11 (d, *J* = 8.4 Hz, 1H), 3.79 (s, 1H), 2.28 (d, *J* = 8.6 Hz, 1H), 2.13 (t, *J* = 8.2 Hz, 1H), 1.70 (dd, *J* = 6.8, 1.4 Hz, 3H). ^{13}C NMR (101 MHz, $CDCl_3$) δ 144.10, 131.24, 128.90, 128.85, 127.76, 125.70, 125.66, 125.62, 125.59, 122.02, 98.37, 69.77, 37.05, 35.80, 26.93, 13.51. HRMS m/z: (APCI) [M+H]$^+$, calculated for $C_{15}H_{16}F_3O_2$; 285.1097; found 285.1076.

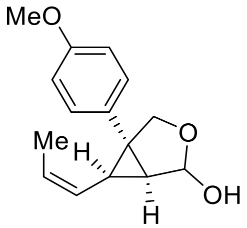

(1S*,5R*,6R*)-5-(4-Methoxyphenyl)-6-((Z)-prop-1-en-1-yl)-3-oxabicyclo[3.1.0]hexan-2-ol (4n)

Prepared from 7d and dry DMF (5 eq.) as carbonyl electrophile by procedure A. <u>dr (lactol 4:1)</u>

^{1}H NMR (400 MHz, $CDCl_3$) δ 7.23 – 7.17 (m, 2H), 6.88 – 6.83 (m, 2H), 5.70 (dqd, *J* = 10.8, 6.8, 1.3 Hz, 1H), 5.48 – 5.40 (m, 1H), 5.38 (s, 1H), 4.26 (d, *J* = 8.4 Hz, 1H), 4.05 (d, *J* = 8.4 Hz, 1H), 3.79 (s, 3H), 2.15 (d, *J* = 8.4 Hz, 1H), 2.09 (dd, *J* = 9.8, 7.7 Hz, 1H), 1.73 (dd, *J* = 6.8, 1.7 Hz, 3H). ^{13}C NMR (101 MHz, $CDCl_3$) δ 158.47, 131.75, 130.01, 128.97, 127.94, 122.76, 114.05, 114.02, 98.53, 70.71, 55.35, 36.81, 35.29, 25.58, 13.43. HRMS m/z: (APCI) [M+Na]$^+$, calculated for $C_{15}H_{18}NaO_3$; 269.1148; found 269.1151.

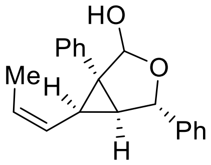

(1R*,4S*,5S*,6R*)-1,4-Diphenyl-6-((Z)-prop-1-en-1-yl)-3-oxabicyclo[3.1.0]hexan-2-ol (4o)

Compound **9** was dissolved in diethyl ether (0.1 M) in a flame dried one neck flask under argon atmosphere. The stirred mixture was cooled to -78 °C and DIBAL-H (1.2 eq.) was added. The mixture was let to gradually warm up to -20 °C and monitored by TLC. Upon completion, the reaction was carefully hydrolyzed with an aqueous solution of HCl (1M) and stirred for 1 hour. The aqueous phase was extracted with Et_2O three times. The combined organic phases were dried over anhydrous $MgSO_4$ and concentrated under reduced pressure. The resulting crude mixture was purified by column chromatography. (dr lactol = 2:1)

^{1}H NMR (400 MHz, $CDCl_3$) δ 7.61 (dd, *J* = 5.0, 3.4 Hz, 2H), 7.37 – 7.31 (m, 4H), 7.31 – 7.27 (m, 4H), 7.24 – 7.18 (m, 2H), 6.05 – 5.85 (m, 1H), 5.83 – 5.73 (m, 1H), 5.56 – 5.47 (m, 1H), 5.00 (s, 1H), 3.12 (d, *J* = 5.1 Hz, 1H), 2.47 (d, *J* = 8.1 Hz, 1H), 2.08 (t, *J* = 8.3 Hz, 1H), 1.73 (dd, *J* = 6.9, 1.7 Hz, 3H). ^{13}C NMR (101 MHz, $CDCl_3$) δ 142.87, 142.62, 138.67, 138.48, 129.90, 129.29, 129.18, 128.83, 128.76, 128.69, 128.63, 128.47, 128.42, 127.79, 127.73, 127.30, 127.08, 127.01, 126.97, 125.74, 123.87, 122.28,

104.03, 99.52, 81.59, 79.02, 44.56, 42.55, 35.84, 34.40, 27.97, 26.89, 13.90, 13.62. HRMS m/z: (APCI) [M-H]+, calculated for $C_{20}H_{19}O2$; 291.1380; found 291.1398.

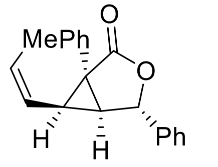

(1R*,4S*,5S*,6R*)-1,4-Diphenyl-6-((Z)-prop-1-en-1-yl)-3-oxabicyclo[3.1.0]hexan-2-one (9)

Compound **4c** (1 mmol) was dissolved in DCM in a one-neck flask and TEMPO (20 mol%, 0.2 mmol, 31 mg) was added in one portion. PIDA (4 eq., 4 mmol, 1288 mg) was then added, and the reaction was stirred until completion, as monitored by TLC. The solvent was evaporated and the product was purified by column chromatography to afford compound **9** as white solid in 92% yield.[7]

^{1}H NMR (400 MHz, $CDCl_3$) δ 7.47 – 7.29 (m, 10H), 5.98 (dqd, *J* = 10.7, 6.9, 1.4 Hz, 1H), 5.46 (ddq, *J* = 11.6, 8.0, 1.7 Hz, 1H), 5.32 (s, 1H), 2.88 – 2.75 (m, 1H), 2.61 (t, *J* = 8.0 Hz, 1H), 1.83 (dd, *J* = 6.9, 1.5 Hz, 3H). ^{13}C NMR (101 MHz, $CDCl_3$) δ 173.82, 139.56, 134.52, 132.09, 129.19, 129.02, 128.86, 128.24, 127.95, 125.91, 120.78, 38.96, 37.35, 29.55, 13.99. HRMS m/z: (APCI) [M+H]+, calculated for $C_{20}H_{19}O_2$; 291.1380; found 291.1400.

CCDC Deposition Number 2023855

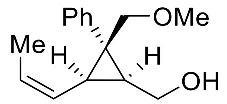

((1S*,2R*,3R*)-2-(Methoxymethyl)-2-phenyl-3-((Z)-prop-1-en-1-yl)cyclopropyl)methanol (12)

Prepared from **7e** and dry DMF (5 eq.) by procedure A.

^{1}H NMR (400 MHz, $CDCl_3$) δ 7.33 (d, *J* = 7.7 Hz, 2H), 7.24 (t, *J* = 7.5 Hz, 2H), 7.16 (dd, *J* = 12.9, 5.6 Hz, 1H), 5.64 (dq, *J* = 13.6, 6.8 Hz, 1H), 5.33 (t, *J* = 9.3 Hz, 1H), 3.90 (td, *J* = 11.6, 5.3 Hz, 1H), 3.73 (d, *J* = 10.3 Hz, 1H), 3.66 – 3.55 (m, 2H), 3.27 – 3.13 (m, 4H), 2.05 (t, *J* = 8.6 Hz, 1H), 1.96 – 1.81 (m, 1H), 1.67 (d, *J* = 6.8 Hz, 3H). ^{13}C NMR (101 MHz, $CDCl_3$) δ 145.68, 129.00, 128.82, 128.40, 126.66, 123.59, 74.23, 59.84, 59.12, 34.90, 31.10, 27.07, 13.57. HRMS m/z: (APCI) [M+H]+, calculated for $C_{15}H_{21}O_2$; 233.1536; found 233.1565.

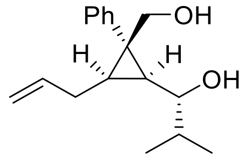

(R*)-1-((1S*,2R*,3R*)-3-Allyl-2-(hydroxymethyl)-2-phenylcyclopropyl)-2-methylpropan-1-ol (SI-1)

Prepared from 7b and freshly distilled isobutyraldehyde (2 eq.) as carbonyl electrophile by procedure B.

^{1}H NMR (400 MHz, $CDCl_3$) δ 7.38 (dd, *J* = 8.1, 1.1 Hz, 2H), 7.31 (dd, *J* = 10.2, 4.8 Hz, 2H), 7.24 – 7.19 (m, 1H), 5.92 (ddt, *J* = 16.8, 10.2, 6.6 Hz, 1H), 5.11 (ddd, *J* = 13.6, 11.4, 1.4 Hz, 2H), 4.05 (d, *J* = 11.9 Hz, 1H), 3.82 (d, *J* = 11.9 Hz, 1H), 3.48 (dd, *J* = 10.4, 6.2 Hz, 1H), 2.47 – 2.36 (m, 1H), 2.21 – 2.11 (m, 1H), 1.83 (dt, *J* = 13.3, 6.7 Hz, 1H), 1.51 (t, *J* = 9.8 Hz, 1H), 1.41 – 1.32 (m, 1H), 1.04 (d, *J* = 6.7 Hz, 3H), 1.01 (d, *J* = 6.9 Hz, 3H). ^{13}C NMR (101 MHz, $CDCl_3$) δ 145.68, 137.62, 129.46, 128.49, 126.65, 115.70, 73.83, 63.99, 34.67, 33.86, 31.73, 29.87, 29.20, 19.05, 18.31. HRMS m/z: (APCI) $[M-OH]^+$, calculated for $C_{17}H_{23}O$; 243.1743; found 243.1771.

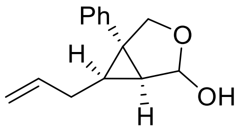

(1S*,5R*,6R*)-6-Allyl-5-phenyl-3-oxabicyclo[3.1.0]hexan-2-ol (SI-2) <u>dr (lactol 5:1)</u>

Prepared from **7b** and dry DMF (5 eq.) as carbonyl electrophile by procedure B.

^{1}H NMR (400 MHz, $CDCl_3$) δ 7.33 – 7.29 (m, 2H), 7.23 (dd, *J* = 6.5, 1.8 Hz, 3H), 5.94 (ddt, *J* = 16.6, 10.2, 6.3 Hz, 1H), 5.40 (s, 1H), 5.12 (ddd, *J* = 17.1, 3.5, 1.7 Hz, 1H), 5.03 (ddd, *J* = 10.2, 3.2, 1.4 Hz, 1H), 4.35 (dd, *J* = 8.5, 0.4 Hz, 1H), 4.12 (d, *J* = 8.6 Hz, 1H), 2.39 – 2.31 (m, 2H), 2.06 (d, *J* = 8.7 Hz, 1H), 1.46 – 1.39 (m, 1H). ^{13}C NMR (101 MHz, $CDCl_3$) δ 140.43, 137.93, 128.62, 127.82, 126.69, 115.05, 98.41, 70.65, 35.67, 34.41, 27.35, 26.31. HRMS m/z: (APCI) $[M-OH]^+$, calculated for $C_{14}H_{15}O$; 199.1117; found 199.1135.

General procedure for the tandem Heck arylation/ vinyl cyclopropyl carbinols ring opening with arylboronic acids (Procedure E)

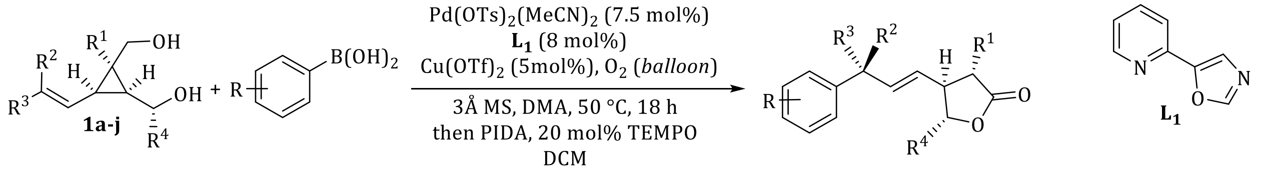

In a dry one-neck flask under O_2 (balloon, 1 atm), 5-(2-pyridyl)-1,3-oxazole (8 mol%), $[Pd(MeCN)_2(OTs)_2]$ (7.5 mol%), Cu(OTf)2 (5 mol%), molecular sieves 3 Å (100% w/w substrate) and vinyl cyclopropyl diol (1 equiv., 0.2 mmol) were dissolved into DMA (8 mL/mmol substrate). The corresponding arylboronic acid (3 equiv.) was then rapidly poured into the reactional mixture which was set on stirring at 50 °C for 18 h. After completion of the reaction, the reactional mixture was diluted with brine and EtOAc. The phases were separated, and the aqueous phase was successively washed three times with EtOAc. The combined organic phases were then dried over anhydrous $MgSO_4$ and concentrated under reduced pressure. Column chromatography afforded the lactol which were subsequently oxidized: The lactol product was dissolved in DCM in a one-neck flask and TEMPO (20 mol%) was added in one portion. PIDA (1.5 eq.) was then added, and the reaction was stirred until completion, as monitored by TLC. The solvent was evaporated, and the product was purified by column chromatography.

General procedure for the tandem Heck alkenylation/ vinyl cyclopropyl carbinols ring opening with alkenyltriflates (Procedure F)

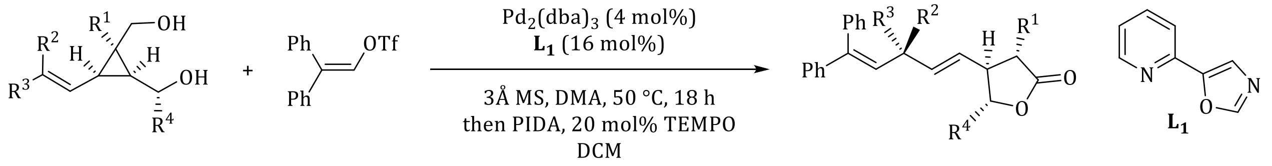

In a dry one-neck flask under Ar (balloon, 1 atm), 5-(2-pyridyl)-1,3-oxazole (16 mol%), Pd_2dba_3 (4 mol%) molecular sieves 3 Å (100% w/w substrate) and vinyl cyclopropyl carbinol (1 eq., 0.2 mmol) were dissolved into DMA (8 mL/mmol substrate). The corresponding alkenyl triflate[8] (2 eq.) was then added into the reactional mixture which was set on stirring at 50 °C for 18 h. After completion of the reaction, the reactional mixture was diluted with brine and EtOAc. The phases were separated, and the aqueous phase was successively washed three times with EtOAc. The combined organic phases were then dried over anhydrous $MgSO_4$ and concentrated under reduced pressure. Column chromatography afforded the lactol which were subsequently oxidized: The lactol product was dissolved in DCM in a one-neck flask and TEMPO (20 mol%) was added in one portion. PIDA (1.5 eq.) was then added, and the reaction was stirred until completion, as monitored by TLC. The solvent was evaporated, and the product was purified by column chromatography.

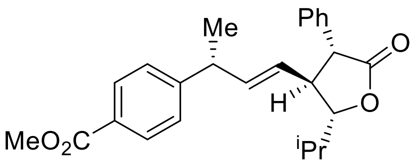

Methyl 4-((S*,E)-4-((2R*,3S*,4R*)-2-isopropyl-5-oxo-4-phenyltetrahydrofuran-3-yl)but-3-en-2-yl)benzoate (10a)

Prepared through procedure E from compound **4b**.

^{1}H NMR (400 MHz, $CDCl_3$) δ 7.91 (d, *J* = 8.3 Hz, 2H), 7.39 – 7.29 (m, 3H), 7.21 – 7.14 (m, 2H), 7.10 (d, *J* = 8.3 Hz, 2H), 5.53 (dd, *J* = 15.4, 6.2 Hz, 1H), 5.45 – 5.34 (m, 1H), 4.08 (dd, *J* = 9.7, 5.1 Hz, 1H), 3.90 (s, 3H), 3.61 (d, *J* = 11.8 Hz, 1H), 3.47 (p, *J* = 7.1 Hz, 1H), 2.92 (dt, *J* = 11.7, 9.2 Hz, 1H), 1.97 (qd, *J* = 12.2, 6.8 Hz, 1H), 1.29 (d, *J* = 7.0 Hz, 3H), 1.02 (dd, *J* = 6.8, 2.3 Hz, 6H). ^{13}C NMR (101 MHz, $CDCl_3$) δ 175.87, 167.11, 150.41, 139.25, 135.42, 129.92, 128.91, 128.86, 128.38, 127.87, 127.25, 126.61, 86.94, 54.53, 52.99, 42.08, 31.32, 21.00, 19.22, 17.60. HRMS m/z: (APCI) $[M+H]^+$, calculated for $C_{25}H_{29}O_4$; 393.2060; found 393.2060.

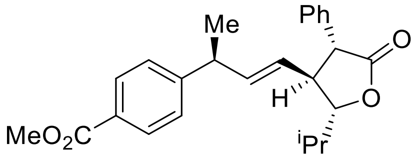

Methyl 4-((S*,E)-4-((2R*,3S*,4R*)-2-isopropyl-5-oxo-4-phenyltetrahydrofuran-3-yl)but-3-en-2-yl)benzoate (10b)

Prepared through procedure E from compound **4a**.

^{1}H NMR (400 MHz, $CDCl_3$) δ 7.90 (d, *J* = 8.3 Hz, 2H), 7.26 (dd, *J* = 5.3, 3.4 Hz, 3H), 7.16 – 7.02 (m, 4H), 5.48 (dd, *J* = 15.4, 6.7 Hz, 1H), 5.42 – 5.32 (m, 1H), 4.06 (dd, *J* = 9.6, 5.2 Hz, 1H), 3.88 (s, 3H), 3.55 (d, *J* = 11.8 Hz, 1H), 3.44 (p, *J* = 6.9 Hz, 1H), 2.88 (ddd, *J* = 11.7, 9.5, 8.5 Hz, 1H), 2.02 – 1.91 (m, 1H), 1.27 (d, *J* = 7.0 Hz, 3H), 1.02 (dd, *J* = 6.8, 1.1 Hz, 6H). ^{13}C NMR (101 MHz, $CDCl_3$) δ 175.83, 167.05, 150.44, 139.17, 135.36, 129.82, 128.80, 128.75, 128.26, 127.71, 127.15, 126.77, 86.88, 54.34, 52.86, 52.11, 42.04, 31.36, 20.85, 19.13, 17.62. HRMS m/z: (APCI) $[M+H]^+$, calculated for $C_{25}H_{29}O_4$; 393.2060; found 393.2060.

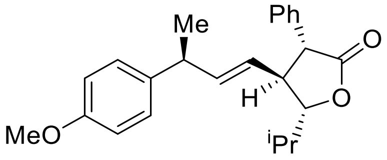

(3R*,4S*,5R*)-5-Isopropyl-4-((S*,E)-3-(4-methoxyphenyl)but-1-en-1-yl)-3-phenyldihydrofuran-2(3H)-one (10c)

Prepared through procedure E from compound **4a**.

^{1}H NMR (400 MHz, $CDCl_3$) δ 7.32 – 7.20 (m, 3H), 7.06 (dd, J = 7.9, 1.5 Hz, 2H), 6.96 – 6.84 (m, 2H), 6.77 – 6.70 (m, 2H), 5.44 (dd, J = 15.4, 6.7 Hz, 1H), 5.28 (ddd, J = 15.4, 8.4, 1.1 Hz, 1H), 4.01 (dd, J = 9.6, 5.1 Hz, 1H), 3.72 (s, 3H), 3.51 (d, J = 11.7 Hz, 1H), 3.34 – 3.26 (m, 1H), 2.90 – 2.77 (m, 1H), 1.93 (dq, J = 12.0, 6.8 Hz, 1H), 0.99 (dd, J = 6.8, 4.5 Hz, 6H). ^{13}C NMR (101 MHz, $CDCl_3$) δ 176.08, 158.10, 140.56, 137.19, 135.57, 128.84, 128.12, 127.70, 125.78, 113.88, 87.11, 55.40, 54.45, 52.80, 41.17, 31.36, 21.19, 19.28, 17.63. HRMS m/z: (APCI) $[M+H]^+$, calculated for $C_{24}H_{29}O_3$; 365.2111; found 365.2126.

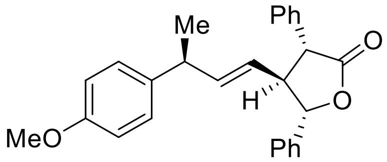

(3R*,4S*,5S*)-4-((S*,E)-3-(4-Methoxyphenyl)but-1-en-1-yl)-3,5-diphenyldihydrofuran-2(3H)-one (10d)

Prepared through procedure E from compound **4c**.

^{1}H NMR (400 MHz, CDCl3) δ 7.43 – 7.27 (m, 4H), 7.25 – 7.20 (m, 1H), 6.96 – 6.89 (m, 2H), 6.81 – 6.74 (m, 2H), 5.45 – 5.40 (m, 1H), 5.18 (dd, J = 9.9, 4.1 Hz, 1H), 3.81 (s, 1H), 3.78 (s, 2H), 3.41 – 3.30 (m, 1H), 3.14 – 3.01 (m, 1H), 2.35 (t, J = 7.5 Hz, 1H), 1.24 (s, 2H). ^{13}C NMR (101 MHz, CDCl3) δ 175.55, 158.01, 141.83, 137.12, 136.96, 134.88, 128.83, 128.76, 128.70, 128.61, 128.06, 127.80, 126.10, 123.41, 113.75, 83.56, 57.78, 55.31, 53.86, 40.99, 21.11. HRMS m/z: (APCI) [M+H]+, calculated for $C_{27}H_{27}O3$; 399.1960; found 399.1955.

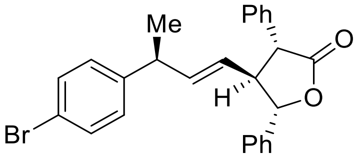

(3R*,4S*,5S*)-4-((S*,E)-3-(4-Bromophenyl)but-1-en-1-yl)-3,5-diphenyldihydrofuran-2(3H)-one (10e)

Prepared through procedure E from compound **4c**.

^{1}H NMR (400 MHz, $CDCl_3$) δ 7.44 – 7.29 (m, 10H), 7.25 – 7.18 (m, 2H), 6.88 – 6.82 (m, 2H), 5.52 – 5.35 (m, 2H), 5.23 – 5.14 (m, 1H), 3.84 – 3.76 (m, 1H), 3.35 (p, J = 6.8 Hz, 1H), 3.15 – 3.02 (m, 1H), 1.23 (d, J = 7.0 Hz, 3H). ^{13}C NMR (101 MHz, $CDCl_3$) δ 175.47, 143.98, 140.94, 137.09, 134.87, 132.00, 131.54, 128.98, 128.95, 128.78, 128.75, 127.97, 127.80, 127.03, 126.21, 124.27, 120.11, 83.57, 77.48, 77.36, 77.16, 76.84, 57.97, 53.96, 41.39, 20.98. HRMS m/z: (APCI) $[M+H]^+$, calculated for $C_{26}H_{24}BrO_2$; 447.0954; found 447.0943.

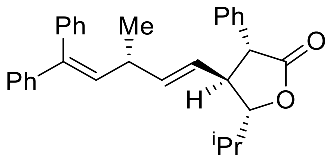

(3R*,4S*,5R*)-5-Isopropyl-4-((R*,E)-3-methyl-5,5-diphenylpenta-1,4-dien-1-yl)-3-phenyldihydrofuran-2(3H)-one (10g)

Prepared through procedure F from compound **4b**.

^{1}H NMR (400 MHz, $CDCl_3$) δ 7.31 – 7.22 (m, 6H), 7.19 (ddd, *J* = 9.1, 5.0, 3.4 Hz, 4H), 7.13 – 7.06 (m, 4H), 7.03 (dd, *J* = 6.5, 3.0 Hz, 2H), 5.71 (d, *J* = 9.9 Hz, 1H), 5.26 (qd, *J* = 15.5, 6.7 Hz, 2H), 3.99 (dd, *J* = 9.7, 5.0 Hz, 1H), 3.49 (d, *J* = 11.7 Hz, 1H), 2.93 – 2.73 (m, 2H), 1.91 (dq, *J* = 13.5, 6.8 Hz, 1H), 1.00 – 0.93 (m, 9H). ^{13}C NMR (101 MHz, $CDCl_3$) δ 175.97, 142.30, 141.26, 139.95, 139.85, 135.52, 132.16, 129.56, 128.78, 128.22, 128.18, 127.66, 127.33, 127.22, 127.20, 125.43, 86.95, 54.48, 52.91, 36.75, 31.15, 29.74, 21.06, 19.26, 17.48. HRMS m/z: (APCI) [M+H]$^+$, calculated for $C_{31}H_{33}O_2$; 437.2475; found 437.2484.

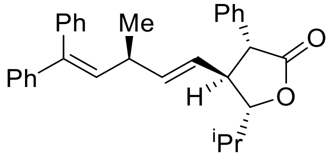

(3R*,4S*,5R*)-5-Isopropyl-4-((S*,E)-3-methyl-5,5-diphenylpenta-1,4-dien-1-yl)-3-phenyldihydrofuran-2(3H)-one (10h)

Prepared through procedure F from compound **4a**.

^{1}H NMR (400 MHz, $CDCl_3$) δ 7.28 – 7.14 (m, 9H), 7.10 (dd, *J* = 11.2, 5.7 Hz, 5H), 7.04 (d, *J* = 7.0 Hz, 2H), 5.69 (d, *J* = 9.9 Hz, 1H), 5.29 (dd, *J* = 15.4, 5.6 Hz, 1H), 5.20 (dd, *J* = 15.5, 8.1 Hz, 1H), 3.96 (dd, *J* = 9.5, 4.9 Hz, 1H), 3.48 (d, *J* = 11.6 Hz, 1H), 2.97 – 2.70 (m, 2H), 1.90 (dd, *J* = 12.3, 6.3 Hz, 1H), 0.96 (dd, *J* = 11.0, 4.6 Hz, 9H). ^{13}C NMR (101 MHz, $CDCl_3$) δ 175.46, 141.77, 140.56, 139.50, 139.15, 135.03, 131.79, 129.07, 128.27, 128.21, 127.76, 127.73, 127.68, 127.15, 126.83, 126.79, 126.70, 125.05, 86.47, 53.83, 52.11, 36.32, 30.73, 20.51, 18.69, 17.06. HRMS m/z: (APCI) [M+H]$^+$, calculated for $C_{31}H_{33}O_2$; 437.2475; found 437.2496.

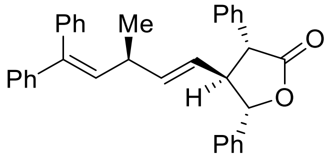

(3R*,4S*,5S*)-4-((S*,E)-3-Methyl-5,5-diphenylpenta-1,4-dien-1-yl)-3,5-diphenyldihydrofuran-2(3H)-one (10i)

Prepared through procedure F from compound **4c**.

^{1}H NMR (400 MHz, CDCl3) δ 7.28 (d, J = 7.3 Hz, 6H), 7.26 – 7.19 (m, 5H), 7.15 (dt, J = 6.2, 5.1 Hz, 5H), 7.09 (dd, J = 6.6, 5.1 Hz, 2H), 7.00 (dd, J = 6.8, 2.3 Hz, 2H), 5.66 (dd, J = 9.9, 3.8 Hz, 1H), 5.27 (dd, J = 15.5, 8.0 Hz, 1H), 5.16 (dd, J = 15.4, 5.7 Hz, 1H), 5.03 (d, J = 9.8 Hz, 1H), 3.66 (d, J = 12.1 Hz, 1H), 2.93 (dt, J = 11.7, 9.6 Hz, 1H), 2.84 (dt, J = 9.8, 6.4 Hz, 1H), 0.90 (d, J = 6.8 Hz, 3H). ^{13}C NMR (101 MHz, $CDCl_3$) δ 175.13, 141.83, 140.75, 140.73, 139.51, 136.71, 134.51, 131.74, 129.13, 128.39, 128.31, 128.24, 128.17, 127.81, 127.75, 127.38, 126.88, 126.80, 126.78, 125.66, 122.75, 77.38, 77.06, 76.74, 57.35, 53.37, 36.35, 20.53. HRMS m/z: (APCI) $[M+H]^+$, calculated for $C_{34}H_{31}O_2$; 471.2319; found 471.2327.

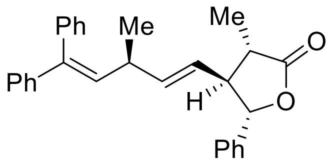

(3S*,4S*,5S*)-3-Methyl-4-((S*,E)-3-methyl-5,5-diphenylpenta-1,4-dien-1-yl)-5-phenyldihydrofuran-2(3H)-one (10j)

Prepared through procedure F from compound **4g**.

^{1}H NMR (400 MHz, $CDCl_3$) δ 7.19 (ddt, *J* = 12.4, 8.0, 5.3 Hz, 10H), 7.07 (dd, *J* = 7.4, 1.6 Hz, 2H), 5.76 (d, *J* = 9.8 Hz, 1H), 5.37 (dd, *J* = 15.4, 5.7 Hz, 1H), 5.21 (dd, *J* = 15.5, 7.4 Hz, 1H), 4.90 (d, *J* = 9.3 Hz, 1H), 3.02 – 2.84 (m, 1H), 2.56 – 2.35 (m, 2H), 1.18 (d, *J* = 6.5 Hz, 3H), 1.00 (d, *J* = 6.8 Hz, 3H). ^{13}C NMR (101 MHz, $CDCl_3$) δ 177.87, 142.09, 141.22, 140.55, 139.83, 137.30, 132.03, 129.43, 128.40, 128.35, 128.12, 128.06, 127.14, 127.10, 127.08, 125.79, 123.83, 83.53, 57.09, 42.00, 36.63, 27.62, 21.00, 17.96, 12.76. HRMS m/z: (APCI) $[M+H]^+$, calculated for $C_{29}H_{29}O_2$; 409.2168; found 409.2171.

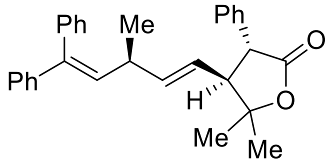

(3R*,4S*)-5,5-Dimethyl-4-((S*,E)-3-methyl-5,5-diphenylpenta-1,4-dien-1-yl)-3-phenyldihydrofuran-2(3H)-one (10k)

Prepared through procedure F from compound **4d**.

^{1}H NMR (400 MHz, $CDCl_3$) δ 7.31 – 7.23 (m, 5H), 7.18 (qd, *J* = 6.3, 3.7 Hz, 4H), 7.14 – 7.08 (m, 4H), 7.03 (dd, *J* = 7.5, 1.7 Hz, 2H), 5.69 (d, *J* = 10.0 Hz, 1H), 5.40 (dd, *J* = 15.4, 6.1 Hz, 1H), 5.21 (ddd, *J* = 15.3, 8.6, 1.0 Hz, 1H), 3.65 (d, *J* = 12.5 Hz, 1H), 2.96 – 2.84 (m, 1H), 2.79 (dt, *J* = 17.3, 8.7 Hz, 1H), 1.41 (s, 3H), 1.26 (s, 3H), 0.97 (d, *J* = 6.8 Hz, 3H). ^{13}C NMR (101 MHz, $CDCl_3$) δ 175.33, 142.11, 140.92, 140.60, 139.83, 135.39, 132.16, 129.42, 128.55, 128.44, 128.08, 128.00, 127.43, 127.12, 127.03, 127.01, 122.86, 84.36, 58.27, 51.79, 36.72, 27.03, 22.51, 20.97. HRMS m/z: (APCI) $[M+H]^+$, calculated for $C_{30}H_{31}O_2$; 423.2319; found 423.2330.

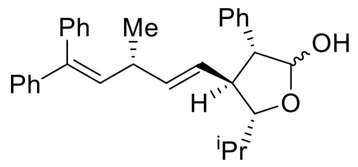

(3R*,4S*,5R*)-5-isopropyl-4-((R,E)-3-methyl-5,5-diphenylpenta-1,4-dien-1-yl)-3-phenyltetrahydrofuran-2-ol (10l)

Prepared through procedure F from compound **4b**, omitting the oxidation step.

^{1}H NMR (400 MHz, $CDCl_3$) δ 7.27 – 7.06 (m, 14H), 7.05 – 7.02 (m, 1H), 5.70 (d, J = 10.0 Hz, 1H), 5.42 – 5.33 (m, 1H), 5.23 (dd, J = 5.3, 2.9 Hz, 1H), 3.84 (dd, J = 9.5, 4.9 Hz, 1H), 2.94 (dd, J = 9.4, 4.1 Hz, 1H), 2.87 – 2.71 (m, 1H), 2.60 – 2.50 (m, 1H), 1.80 (ddd, J = 11.9, 9.8, 5.6 Hz, 1H), 0.97 – 0.87 (m, 9H). ^{13}C NMR (101 MHz, $CDCl_3$) δ 142.66, 140.76, 140.40, 140.22, 137.57, 133.09, 129.75, 129.68, 129.62, 128.67, 128.32, 128.22, 128.20, 128.18, 128.15, 127.96, 127.63, 127.49, 127.46, 127.12, 127.07, 127.05, 126.86, 103.60, 87.47, 61.13, 55.40, 36.84, 31.07, 21.26, 19.75, 18.07. HRMS m/z: (APCI) $[M+H]^+$, calculated for $C_{31}H_{35}O_2$; 439.2637; found 439.2643.

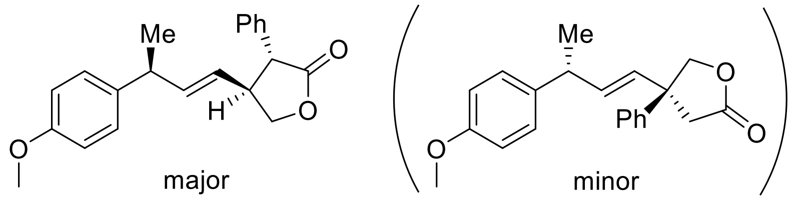

(3R*,4S*)-4-((S*,E)-3-(4-Methoxyphenyl)but-1-en-1-yl)-3-phenyldihydrofuran-2(3H)-one (10m)

Prepared through procedure E from compound **4e**. Major : minor = 3:1

^{1}H NMR (400 MHz, CDCl3) δ 7.39 – 7.29 (m, 3H), 7.18 (dd, J = 10.4, 4.0 Hz, 2H), 7.07 – 6.97 (m, 2H), 6.86 – 6.78 (m, 2H), 5.67 – 5.58 (m, 1H), 5.36 (ddd, J = 15.4, 7.9, 1.1 Hz, 1H), 4.49 (dd, J = 17.5, 9.4 Hz, 1H), 4.06 (dd, J = 13.1, 6.2 Hz, 1H), 3.79 (s, 3H), 3.51 (d, J = 11.5 Hz, 1H), 3.42 – 3.34 (m, 1H), 3.23 (ddd, J = 18.8, 10.7, 7.9 Hz, 1H), 2.92 (q, J = 16.7 Hz, 1H), 1.29 (s, 3H). ^{13}C NMR (101 MHz, CDCl3) δ 176.56, 158.07, 140.60, 136.98, 135.11, 128.83, 128.65, 128.06, 126.15, 124.39, 113.84, 77.38, 77.06, 76.75, 55.32, 52.44, 48.84, 41.10, 29.75, 21.11. HRMS m/z: (APCI) $[M+H]^+$, calculated for $C_{21}H_{23}O_3$; 323.1647; found 323.1646.

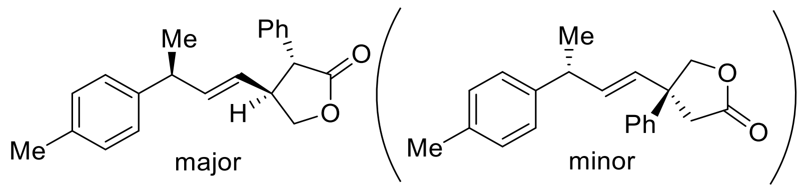

(3R*,4S*)-3-Phenyl-4-((S*,E)-3-(p-tolyl)but-1-en-1-yl)dihydrofuran-2(3H)-one (10n)

Prepared through procedure E from compound **4e**. Major : minor=3:1

^{1}H NMR (400 MHz, $CDCl_3$) δ 7.40 – 7.27 (m, 3H), 7.18 (d, *J* = 6.9 Hz, 2H), 7.09 (d, *J* = 7.7 Hz, 2H), 6.98 (d, *J* = 7.9 Hz, 2H), 5.70 – 5.59 (m, 1H), 5.38 (dd, *J* = 15.4, 7.9 Hz, 1H), 4.49 (q, *J* = 8.5 Hz, 1H), 4.05 (t, *J* = 9.6 Hz, 1H), 3.55 – 3.49 (m, 1H), 3.45 – 3.34 (m, 1H), 3.23 (ddd, *J* = 18.7, 10.6, 7.9 Hz, 1H), 2.32 (s, 3H), 1.29 (d, *J* = 7.1 Hz, 3H). ^{13}C NMR (101 MHz, $CDCl_3$) δ 176.57, 141.90, 140.42, 135.87, 135.12, 129.16, 129.02, 128.83, 128.65, 127.73, 127.01, 124.49, 70.48, 52.42, 48.82, 41.52, 21.06, 21.01. HRMS m/z: (APCI) $[M+H]^+$, calculated for $C_{21}H_{23}O_2$; 307.1698; found 307.1703.

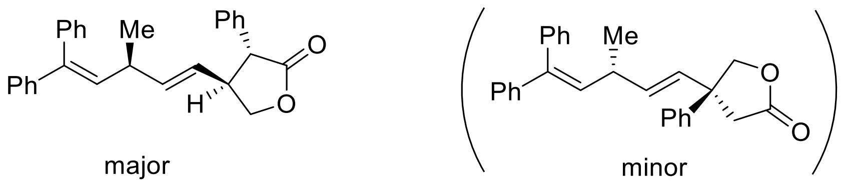

(3R*,4S*)-4-((S*,E)-3-Methyl-5,5-diphenylpenta-1,4-dien-1-yl)-3-phenyldihydrofuran-2(3H)-one (10o)

Prepared through procedure F from compound **4e**. Major : minor=3:1

^{1}H NMR (400 MHz, $CDCl_3$) δ 7.33 – 7.21 (m, 6H), 7.19 – 7.08 (m, 7H), 7.03 (t, *J* = 6.8 Hz, 2H), 5.71 (d, *J* = 9.9 Hz, 1H), 5.44 – 5.38 (m, 1H), 5.22 (dd, *J* = 15.4, 7.7 Hz, 1H), 4.39 (dd, *J* = 8.4, 4.9 Hz, 1H), 3.94 (t, *J* = 9.6 Hz, 1H), 3.42 (d, *J* = 11.4 Hz, 1H), 3.23 – 3.06 (m, 1H), 2.99 – 2.87 (m, 1H), 0.97 (t, *J* = 5.9 Hz, 3H). ^{13}C NMR (101 MHz, $CDCl_3$) δ 176.78, 175.85, 142.46, 142.32, 142.22, 141.57, 141.28, 140.16, 140.10, 139.85, 137.79, 135.38, 134.97, 132.51, 132.32, 131.60, 130.61, 129.81, 129.29, 129.10, 128.94, 128.88, 128.56, 128.51, 128.44, 128.02, 127.81, 127.75, 127.50, 127.47, 126.42, 124.52, 80.09, 79.77, 79.45, 76.68, 70.89, 52.85, 52.73, 50.09, 49.37, 48.97, 40.72, 36.96, 36.91, 21.43, 21.35, 21.28. HRMS m/z: (APCI) $[M+H]^+$, calculated for $C_{28}H_{27}O_2$; 395.2006; found 395.2026.

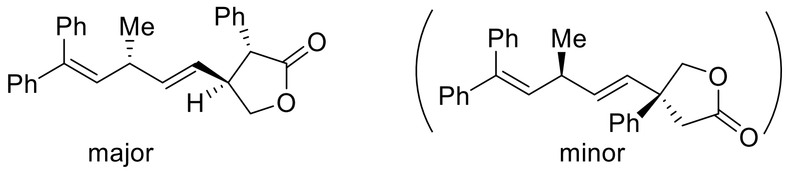

(3R*,4S*)-4-((R*,E)-3-Methyl-5,5-diphenylpenta-1,4-dien-1-yl)-3-phenyldihydrofuran-2(3H)-one (10p)

Prepared through procedure F from compound **4f**. Major : minor=3:1

^{1}H NMR (400 MHz, $CDCl_3$) δ 7.31 – 7.26 (m, 3H), 7.23 (s, 5H), 7.19 – 7.10 (m, 9H), 7.03 (d, *J* = 4.4 Hz, 3H), 5.73 (d, *J* = 9.7 Hz, 1H), 5.39 (dd, *J* = 15.3, 5.4 Hz, 1H), 5.22 (dd, *J* = 15.3, 7.9 Hz, 1H), 4.38 (dd, *J* = 12.4, 8.4 Hz, 1H), 3.93 (t, *J* = 9.5 Hz, 1H), 3.40 (d, *J* = 11.5 Hz, 1H), 3.21 – 3.04 (m, 1H), 3.00 – 2.84 (m, 1H), 0.96 (d, *J* = 6.5 Hz, 3H). ^{13}C NMR (101 MHz, $CDCl_3$) δ 175.59, 174.71, 141.22, 141.12, 141.02, 140.33, 138.96, 138.91, 136.54, 134.15, 133.75, 131.09, 130.40, 129.34, 128.61, 128.56, 128.05, 127.87, 127.71, 127.30, 127.27, 127.22, 127.20, 126.79, 126.51, 126.31, 126.27, 126.24, 126.19,

125.18, 123.24, 75.46, 69.55, 51.58, 48.90, 48.18, 39.32, 35.76, 35.70, 20.18, 20.10. HRMS m/z: (APCI) $[M+H]^+$, calculated for $C_{28}H_{27}O_2$; 395.2006; found 395.2010.

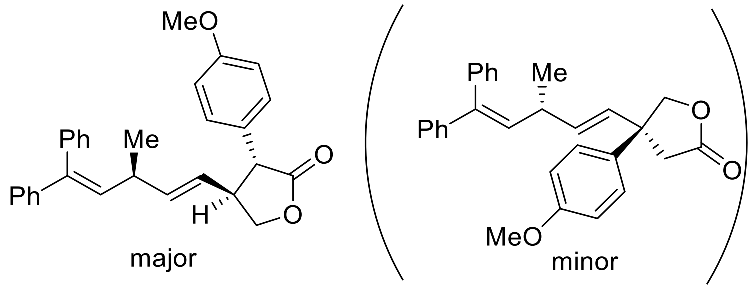

(3R*,4S*)-3-(4-Methoxyphenyl)-4-((S*,E)-3-methyl-5,5-diphenylpenta-1,4-dien-1-yl)dihydrofuran-2(3H)-one (10q)

Prepared through procedure F from compound **4e**. Major : minor=3:1

^{1}H NMR (400 MHz, $CDCl_3$) δ 7.30 – 7.22 (m, 4H), 7.14 (qdd, *J* = 7.6, 3.5, 1.7 Hz, 8H), 7.06 – 7.03 (m, 4H), 7.00 (d, *J* = 8.8 Hz, 1H), 6.81 (dd, *J* = 8.8, 2.5 Hz, 3H), 5.71 (d, *J* = 10.0 Hz, 1H), 5.38 (ddd, *J* = 15.8, 10.4, 5.6 Hz, 1H), 5.20 (ddd, *J* = 15.5, 7.9, 1.0 Hz, 1H), 4.37 (dt, *J* = 13.2, 9.5 Hz, 2H), 3.91 (dd, *J* = 10.1, 9.3 Hz, 1H), 3.70 (s, 3H), 3.36 (d, *J* = 11.6 Hz, 1H), 3.16 – 3.01 (m, 1H), 2.98 – 2.85 (m, 1H), 0.98 (d, *J* = 6.8 Hz, 3H). ^{13}C NMR (101 MHz, $CDCl_3$) δ 176.74, 175.63, 158.95, 158.61, 142.12, 141.99, 141.15, 140.87, 139.82, 139.76, 139.42, 134.50, 133.80, 132.20, 132.03, 131.36, 129.58, 129.52, 129.44, 128.13, 128.02, 127.14, 127.12, 127.07, 127.04, 126.96, 124.19, 114.15, 70.25, 55.16, 51.53, 48.66, 36.59, 20.83. HRMS m/z: (APCI) $[M+H]^+$, calculated for $C_{29}H_{29}O_3$; 425.2111; found 425.2120.

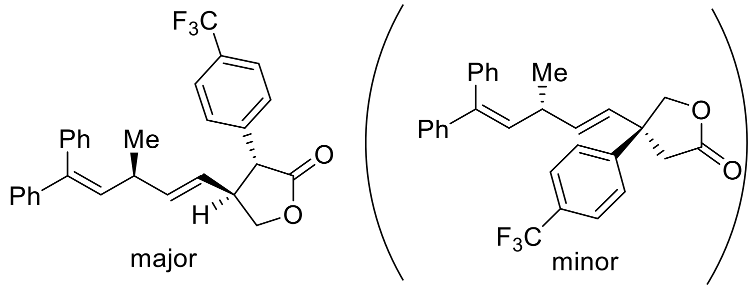

(3R*,4S*)-4-((S*,E)-3-methyl-5,5-diphenylpenta-1,4-dien-1-yl)-3-(4-(trifluoromethyl)phenyl) dihydrofuran-2(3H)-one (10r)

Prepared through procedure F from compound **4e**. Major : minor=3:1

^{1}H NMR (400 MHz, $CDCl_3$) δ 7.59 – 7.49 (m, 3H), 7.26 (t, *J* = 7.9 Hz, 6H), 7.14 (ddd, *J* = 21.6, 12.1, 5.9 Hz, 8H), 7.06 – 7.00 (m, 3H), 5.69 (d, *J* = 10.0 Hz, 1H), 5.42 – 5.36 (m, 1H), 5.20 (dd, *J* = 15.3, 7.9 Hz, 1H), 4.50 – 4.35 (m, 2H), 3.95 (t, *J* = 9.7 Hz, 1H), 3.48 (d, *J* = 11.7 Hz, 1H), 3.20 – 3.04 (m, 1H), 2.92 (dt, *J* = 9.5, 6.7 Hz, 1H), 0.99 (d, *J* = 6.8 Hz, 3H). ^{13}C NMR (101 MHz, $CDCl_3$) δ 175.74, 174.97, 142.21, 141.28,

140.46, 139.97, 139.14, 135.81, 132.08, 131.74, 130.54, 129.58, 129.16, 128.38, 128.27, 127.37, 127.35, 127.30, 127.25, 126.75, 126.09, 126.05, 125.88, 125.84, 123.63, 75.97, 70.59, 52.19, 50.04, 48.90, 40.37, 36.77, 21.15, 20.95. HRMS m/z: (APCI) $[M+H]^+$, calculated for $C_{29}H_{26}F_3O_2$; 463.1879; found 463.1899.

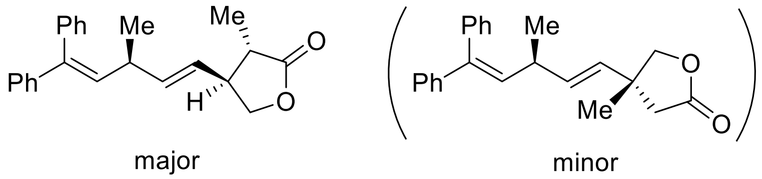

(3S*,4S*)-3-Methyl-4-((S*,E)-3-methyl-5,5-diphenylpenta-1,4-dien-1-yl)dihydrofuran-2(3H)-one (10s)

Prepared through procedure F from compound **4e**. Major: minor=1.5:1

^{1}H NMR (400 MHz, $CDCl_3$) δ 7.41 – 7.31 (m, 3H), 7.25 – 7.21 (m, 3H), 7.19 – 7.13 (m, 2H), 5.87 (d, *J* = 9.9 Hz, 1H), 5.64 (dd, *J* = 15.4, 6.0 Hz, 1H), 5.20 (ddd, *J* = 15.4, 8.2, 1.4 Hz, 1H), 4.33 (dd, *J* = 8.9, 8.0 Hz, 1H), 3.83 (dd, *J* = 10.4, 9.1 Hz, 1H), 3.11 – 2.96 (m, 1H), 2.70 (tt, *J* = 10.8, 8.1 Hz, 1H), 2.28 (dq, *J* = 11.5, 7.0 Hz, 1H), 1.21 (d, *J* = 7.0 Hz, 3H), 1.12 (d, *J* = 6.8 Hz, 3H). ^{13}C NMR (101 MHz, $CDCl_3$) δ 179.18, 142.33, 141.34, 140.09, 139.42, 132.42, 129.70, 128.40, 128.30, 127.33, 127.31, 125.00, 70.54, 48.06, 40.65, 36.78, 21.19, 13.01. HRMS m/z: (APCI) $[M-OH]^+$, calculated for $C_{23}H_{25}O_2$; 333.1855; found 333.1854.

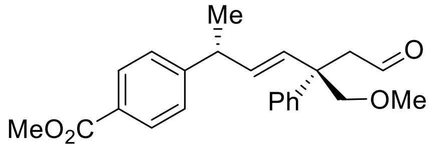

Methyl 4-((2R*,5R*,E)-5-(Methoxymethyl)-7-oxo-5-phenylhept-3-en-2-yl)benzoate (13a)

Prepared through procedure E from compound **12**.

^{1}H NMR (400 MHz, $CDCl_3$) δ 9.52 (s, 1H), 7.92 (d, *J* = 7.9 Hz, 1H), 7.30 – 7.19 (m, 4H), 5.79 – 5.51 (m, 2H), 3.85 (s, 2H), 3.60 (d, *J* = 4.9 Hz, 1H), 3.55 – 3.49 (m, 1H), 3.26 (d, *J* = 8.2 Hz, 2H), 2.88 (dd, *J* = 15.9, 1.9 Hz, 1H), 2.74 (dd, *J* = 15.8, 2.2 Hz, 1H), 1.33 (d, *J* = 6.6 Hz, 2H). ^{13}C NMR (101 MHz, $CDCl_3$) δ 202.48, 167.19, 151.07, 142.96, 135.11, 133.24, 129.98, 129.92, 128.63, 128.25, 127.37, 127.30, 127.13, 126.99, 78.79, 59.20, 52.17, 52.11, 50.23, 47.25, 42.58, 36.72, 24.78, 21.20. HRMS m/z: (APCI) $[M+H]^+$, calculated for $C_{23}H_{27}O_4$; 367.1904; found 367.1897.

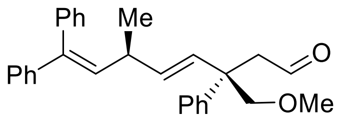

(3R*,6S*,E)-3-(Methoxymethyl)-6-methyl-3,8,8-triphenylocta-4,7-dienal (13b)

Prepared through procedure F from compound **12**.

^{1}H NMR (400 MHz, $CDCl_3$) δ 9.49 (s, 1H), 7.25 (t, *J* = 7.5 Hz, 7H), 7.16 (dd, *J* = 13.9, 5.6 Hz, 6H), 7.09 (d, *J* = 7.5 Hz, 2H), 5.83 (d, *J* = 9.9 Hz, 1H), 5.46 (dt, *J* = 15.9, 10.9 Hz, 2H), 3.57 (dd, *J* = 19.9, 9.3 Hz, 2H), 3.26 (d, *J* = 11.8 Hz, 3H), 3.07 – 2.93 (m, 1H), 2.90 – 2.77 (m, 1H), 2.69 (dd, *J* = 15.8, 2.2 Hz, 1H), 1.05 (d, *J* = 6.7 Hz, 3H). ^{13}C NMR (101 MHz, $CDCl_3$) δ 202.66, 143.12, 142.35, 140.94, 140.08, 135.24, 132.75, 132.12, 129.80, 129.71, 128.51, 128.25, 128.16, 128.12, 127.31, 127.28, 127.14, 127.11, 127.01, 126.85, 78.89, 59.15, 50.14, 47.12, 37.22, 21.35. HRMS m/z: (APCI) $[M+H]^+$, calculated for $C_{29}H_{31}O_2$; 411.2319; found 411.2343.

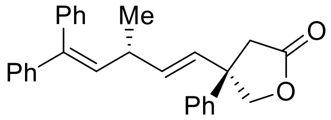

(R*)-4-((S*,E)-3-Methyl-5,5-diphenylpenta-1,4-dien-1-yl)-4-phenyldihydrofuran-2(3H)-one (14a)

Prepared through procedure F from compound **4K**.

^{1}H NMR (400 MHz, $CDCl_3$) δ 7.41 – 7.28 (m, 7H), 7.24 – 7.14 (m, 6H), 7.13 – 7.07 (m, 2H), 5.85 (d, *J* = 9.8 Hz, 1H), 5.55 (d, *J* = 15.8 Hz, 1H), 5.47 (dd, *J* = 15.7, 5.7 Hz, 1H), 4.55 (d, *J* = 8.9 Hz, 1H), 4.46 (dd, *J* = 8.9, 2.0 Hz, 1H), 3.06 – 2.97 (m, 1H), 2.90 (q, *J* = 16.6 Hz, 2H), 1.09 (d, *J* = 6.8 Hz, 3H). ^{13}C NMR (101 MHz, $CDCl_3$) δ 175.74, 142.23, 142.14, 141.50, 140.01, 134.91, 132.20, 131.47, 129.70, 129.12, 128.38, 128.27, 127.58, 127.33, 127.30, 127.27, 126.25, 50.02, 40.49, 36.86, 32.08, 29.85, 29.51, 22.84, 21.26, 14.27. HRMS m/z: (APCI) $[M+H]^+$, calculated for $C_{28}H_{27}O_2$; 395.2006; found 395.2020.

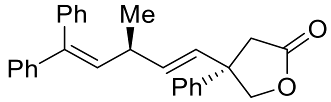

(R*)-4-((R*,E)-3-Methyl-5,5-diphenylpenta-1,4-dien-1-yl)-4-phenyldihydrofuran-2(3H)-one (14b)

Prepared through procedure F from compound **4I**.

^{1}H NMR (400 MHz, $CDCl_3$) δ 7.32 – 7.21 (m, 8H), 7.12 (dddd, *J* = 12.0, 10.6, 6.6, 3.1 Hz, 8H), 7.04 – 7.00 (m, 2H), 5.78 (d, *J* = 9.8 Hz, 1H), 5.48 (d, *J* = 16.1 Hz, 1H), 5.40 (dd, *J* = 15.8, 5.7 Hz, 1H), 4.47 (d, *J* = 8.9 Hz, 1H), 4.38 (d, *J* = 8.9 Hz, 1H), 2.99 – 2.89 (m, 1H), 2.83 (q, *J* = 16.7 Hz, 2H), 1.02 (d, *J* = 6.8 Hz, 3H). ^{13}C NMR (101 MHz, $CDCl_3$) δ 175.78, 142.24, 142.14, 141.49, 140.01, 134.88, 132.21, 131.51, 129.71, 129.12, 128.38, 128.27, 127.58, 127.33, 127.29, 127.26, 126.25, 50.01, 40.38, 36.86, 29.85, 21.24. HRMS m/z: (APCI) $[M+H]^+$, calculated for $C_{28}H_{27}O_2$; 395.2006; found 395.2014.

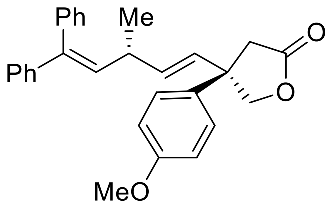

(R*)-4-(4-Methoxyphenyl)-4-((*S,E)-3-methyl-5,5-diphenylpenta-1,4-dien-1-yl)dihydrofuran-2(3H)-one (14c)**

Prepared through procedure F from compound **4n**.

^{1}H NMR (400 MHz, $CDCl_3$) δ 7.32 (dd, *J* = 8.0, 1.9 Hz, 3H), 7.24 – 7.18 (m, 6H), 7.13 – 7.06 (m, 4H), 6.92 – 6.87 (m, 2H), 5.85 (d, *J* = 9.8 Hz, 1H), 5.58 – 5.50 (m, 1H), 5.45 (dd, *J* = 15.7, 5.8 Hz, 1H), 4.50 (d, *J* = 8.8 Hz, 1H), 4.42 (d, *J* = 8.9 Hz, 1H), 3.81 (d, *J* = 1.6 Hz, 3H), 3.01 (dt, *J* = 9.8, 6.7 Hz, 1H), 2.86 (q, *J* = 16.6 Hz, 2H), 1.09 (d, *J* = 6.8 Hz, 3H). ^{13}C NMR (101 MHz, $CDCl_3$) δ 175.79, 158.77, 142.14, 141.31, 139.91, 134.67, 133.95, 132.17, 131.50, 129.60, 128.27, 128.16, 127.28, 127.22, 127.18, 114.29, 55.36, 49.36, 40.65, 36.72, 29.74, 21.17, 14.16. HRMS m/z: (APCI) $[M+H]^+$, calculated for $C_{29}H_{29}O_3$; 425.2111; found 425.2138.

Crystal data and structure refinement of compound 9

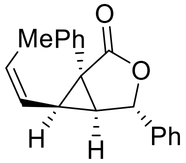

The compound was crystalized from $CHCl_3$. Crystallographic data are summarized in Table S1. Additionally, complete data have been deposited with the Cambridge Crystallographic Data Centre under the number C CCDC Deposition Number 2023855 (9). Copies of the data can be obtained free of charge from www.ccdc.cam.ac.uk/data_request/cif.

**Table S1 Crystal data and structure refinement for 9**	
**Identification code**	Marek164b
**Empirical formula**	$C_{20}H_{18}O_2$
**Formula weight**	290.34
**Temperature/K**	200.15
**Crystal system**	monoclinic
**Space group**	$P2_1/c$
**a/Å**	10.1343(9)
**b/Å**	13.5725(13)
**c/Å**	12.2672(12)
**α/°**	90
**β/°**	112.958(2)
**γ/°**	90
**Volume/Å^3**	1553.7(3)
**Z**	4
**ρ_{calc}g/cm^3**	1.241
**μ/mm^{-1}**	0.079
**F(000)**	616.0
**Crystal size/mm^3**	0.21 × 0.12 × 0.12
**Radiation**	MoKα (λ = 0.71073)
**2Θ range for data collection/°**	4.364 to 50.224
**Index ranges**	$-12 \le h \le 10, -16 \le k \le 16, -14 \le l \le 14$
**Reflections collected**	10694
**Independent reflections**	2751 [R_{int} = 0.0457, R_{sigma} = 0.0473]
**Data/restraints/parameters**	2751/0/200
**Goodness-of-fit on F^2**	1.003
**Final R indexes [I>=2σ (I)]**	R_1 = 0.0444, wR_2 = 0.0963
**Final R indexes [all data]**	R_1 = 0.0887, wR_2 = 0.1161
**Largest diff. peak/hole / e Å^{-3}**	0.16/-0.23

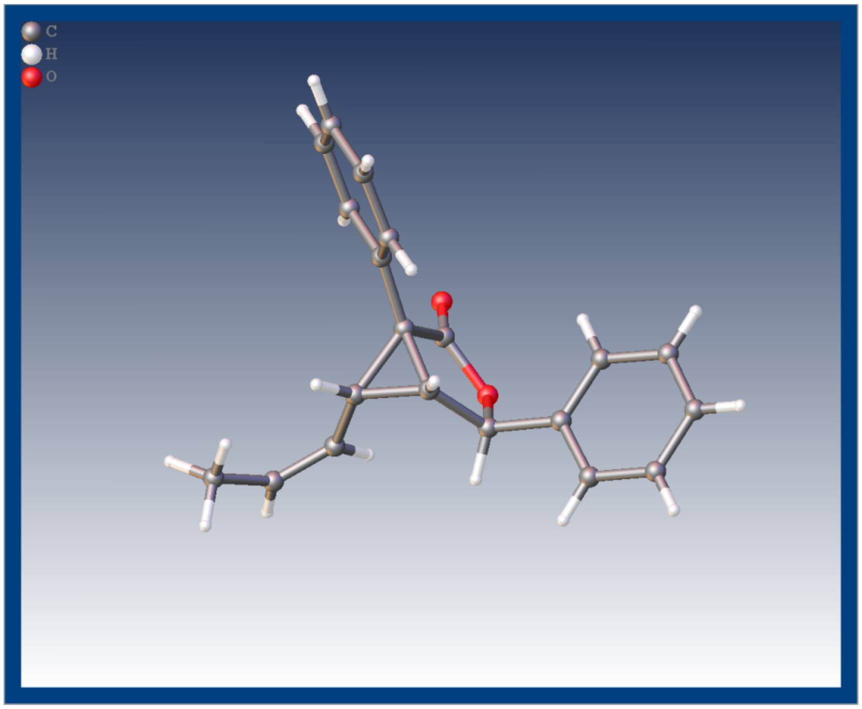

crystal structure acquired for compound 9.

Computational Supporting Information

Computational details

The calculations were performed using Orca 4.2.1[9] and Gaussian 09 Rev. D.01[10]. For geometry optimizations and frequency calculations functional BP86[11–13]-D3(bj)[14] with def2-SVP[15] basis were used, denoted as M2 method. For single point calculations ωB97XD with def2-TZVP[15] were used, denoted as M1 method. For more details see Table S in section ”Conformational search and analysis”. All calculations (optimizations, frequency calculations and single point energies) were performed in DMF using SMD[16] solvation model. PB86 functional in ORCA was treated with the RI-J approximation, whereas hybrid, ωB97XD was treated with the chain-of-sphere approximation[17] to evaluate exchange integrals (RIJCOSX[17]). The optimized minima and transition states were verified by harmonic vibrational analysis to have no and one proper imaginary frequency, respectively. All main transition-state structures (see ”Conformational search and analysis”) were confirmed to connect corresponding reactants and products by intrinsic reaction coordinate (IRC) calculations.[18,19] The quoted energies electronic single point energies by M1 with inclusion of zero-point, enthalpy, and entropic corrections determined from vibrational frequencies calculated at M2 method (M1//M2).

High structural degree of freedom – conformational isomers challenge

In this section we discuss the origin of the especially high structural degree of freedom of the studied molecules making the theoretical study very challenging and then describe the approach we used to overcome the challenges.

Conformers of the pyridyl(oxazole) ligand

Calculated barrier energy (ΔG) for rotation around C-C bond (eq.(S1) is 4.9 kcal/mol, implying fast equilibrium between anti and syn conformers, whereas the energy of syn conformer is 0.3 kcal/mol lower.

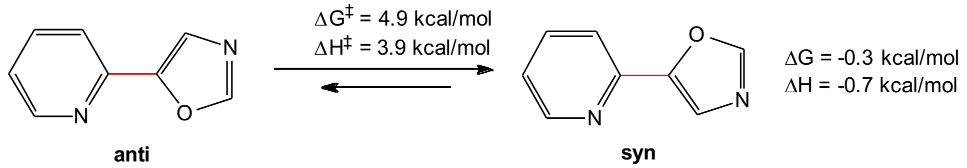

(S1)

The calculated barrier for the same rotation in the ligand coordinated to Pd complex even little bit lower, 4.0 kcal/mol (eq. (S2).

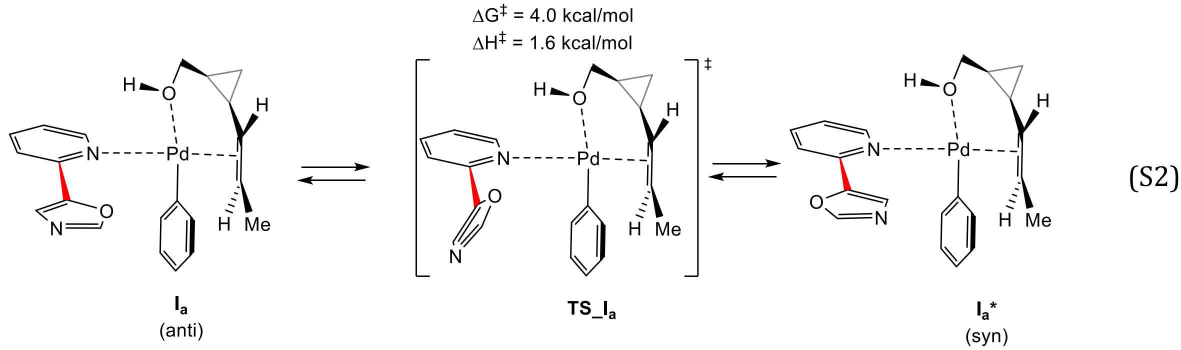

(S2)

Overview of chelating modes of the ligand and alkenyl chain in the complex.

Different modes of coordination of the ligand to Pd complex are presented in Figure S1. Structures **Mod1-Mod4** possess similar energies and are common for the transformation discussed in this manuscript, while **Mod5** is rarer, because of its higher energy. It was found that the ligand can be in mono-chelating (**Mod3**, **Mod4**) and di-chelating modes. The organic moiety (within Pd-complex) can interact with electrophilic metal center (Figure S1, down) via: 1) lone pair of oxygen atom (of hydroxyl group); 2) π interaction with double bonds; 3) π system of aromatic substituent and 4) via agostic interaction of the C-H bons both of hybridizations, sp^2 and sp^3. There were also found π-π stacking interaction in some cases (see an example **A5**).

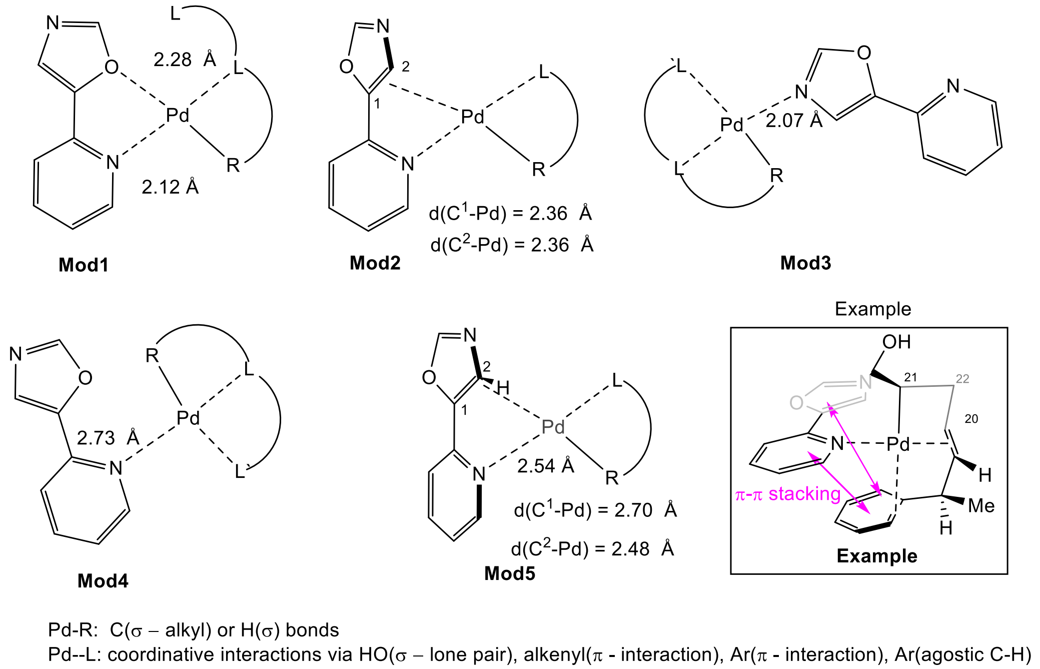

Figure S1. Different modes of coordination (taken from isomers of **A2** and **A3**).

As we demonstrated above - the studied Pd-complex can adopt a variety of conformations (in some cases more than 40 within the energy window of only 3 kcal/mol). This complicates the study of the reaction pathways by the necessity of a thorough conformational search to evaluate the lowest energy intermediates and transition states. The next section describes the method that we used for the conformational search and analysis.

Conformational search and analysis

For conformational search we used CREST[20], program developed by Grimme et al., that makes use of the fast semi-empirical GFNn-xTB[20,21]methods to generate the ensemble of isomers. Further, CREST calculations were followed by DFT calculations. The approach we used here is the sequence of 4 phases of calculations, beginning from a relatively large number of generated conformers (40-130) using fast computations with low levels of theory in early phases of calculations and finishing with a few selected, 6-10 most stable conformers using higher level of theory in later phases, from which the one having the lowest free energy is finally chosen for building respective potential energy surfaces. Cutting down the number of isomers proceeding through the phases was controlled by the electronic energy windows (EEWs) or free energy window (FEW) as illustrated in Figure S2. A set of different methods was used (M0-M3', see Table S below) for different phases, the choice of levels of theory, the necessity of the multi-phase approach and other parameter (energy windows, parameters for CREST program) are discussed shortly at the end of the section.

Here is a short description of all phases (Scheme S1) of calculation for intermediates **I** and transition states **TS** (for **TS** the protocol is slightly corrected see below):

1) Phase 1. Starting structure was obtained based on chemical intuition of the structure and further optimization by M2 method. Feeding the starting structure to CREST with M0 set of parameters (Table S) results in an ensemble of isomers with a varying number of population (in the current study from 20 to 130), depending on the degree of freedom of the studied compound. Further, the lowest energy isomers within the electronic energy window EEW1 = 10 kcal/mol are selected for the following phase.
2) Phase 2. Geometries of the obtained isomers were partially optimized[1] (by M2 method) and their single point energies were calculated (by M1 method). The best isomers were selected for further phase using energy window EEW1 = 3 kcal/mol.
3) In phase 3 full geometry optimizations of the selected isomers are done (by M3 method) followed by frequency calculations by the same method in order to validate the ground state (by the absence of imaginary frequencies for intermediate or one for transition state) and to obtain thermal corrections. Following calculation of single point energies (by M1 method) resulted in the final set of energies (electronic, enthalpy and free energy) by hybrid method M1//M3.
4) In phase 3' we used the same level of theory as in phase 3, without using RI approximations, which accelerate calculations significantly (by 10-15 times!), for 3-6 lowest energy isomers. This method denoted as M3`//M1`. Unlike in phases 2 and 3 where Orca4 was used, here we

[1] As we found later, there are still significant displacements going from partly optimized to fully optimized ensembles, therefore full optimization is strongly recommended (see discussion at the end of the section).

used Gaussian 09. Noteworthy, the differences in relative energies (<0.5 kcal/mol) and respective potential energy surfaces were negligible, indicating that significantly more expensive calculations of phase 3` could be avoided due to the reliable results obtained using accelerating RI approximations.

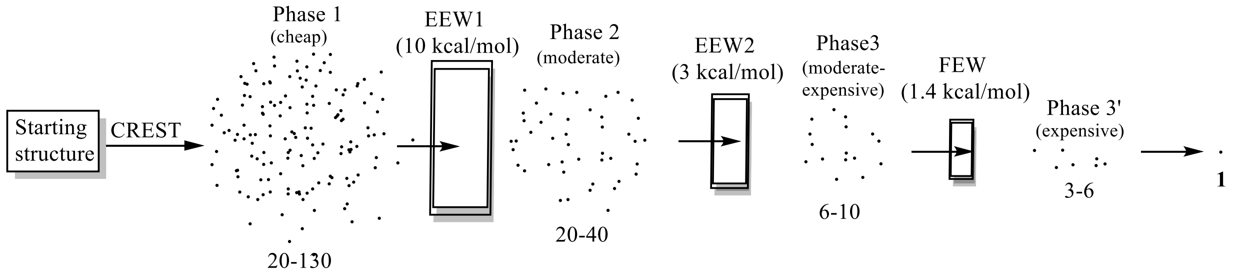

2

Figure S2. Illustration of the workflow presented in Scheme S1 - proceeding through the phases with shrinking energy window filters and converging to the lowest energy isomer **1**.

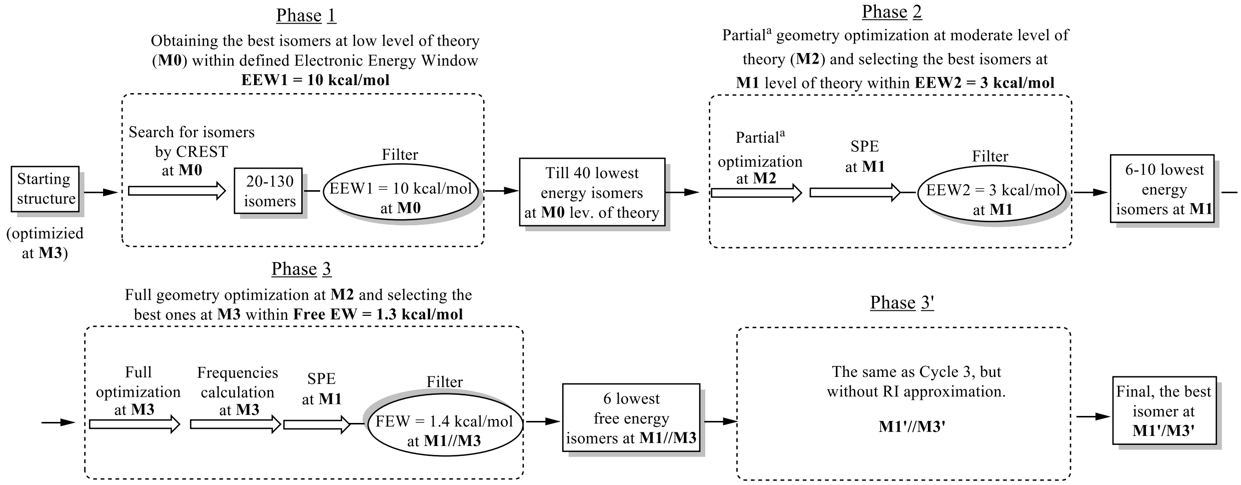

Scheme S1. Workflow for conformational search and analysis generating the lowest energy isomer.

More challenging is the search of possible isomers for transition states. We used the same workflow (Scheme S1) with the only modified phase 2 in which the geometry optimizations were performed with frozen parameter or a couple of parameters that are most affected during the reaction. For example, in reaction of insertion (Scheme S2), bonds Pd2-C3, C1-C2 are forming and bonds Pd2-C1, Pd-π(C2=C3), π(C2-C3) are breaking, with the most affected distances between Pd1-C2 and C1-C2. Running CREST with frozen C1-C2 distance[3] led an ensemble of mostly relevant isomers. Freezing

[3] The distances are obtained from optimized predicted structure, from which the workflow started.

both Pd1-C2 and C1-C2 lead to ensemble of isomers with less irrelevant structures, although nearly *twice smaller* one with a *loss of important (low energy) isomers* obtained by the previous procedure. Generally, less constrain leads to a broader ensemble, which nevertheless contains more irrelevant transition states. While, more constrained search results in ensembles with much less irrelevant structures, although with much less relevant isomers. In the current study all transition state ensembles were found with freezing one, and in rare cases two parameters[3] (bonds or angles). Finally, the best isomer obtained from phase 3' was treated by IRC method with following optimizations and single point calculations to obtain respective reactant and product (Figure S3).

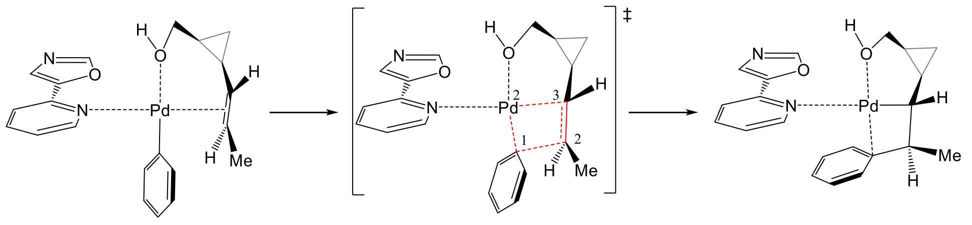

TS1_R TS1$^{s\text{-}trans}$ TS1_P

Scheme S2. Step of insertion reaction.

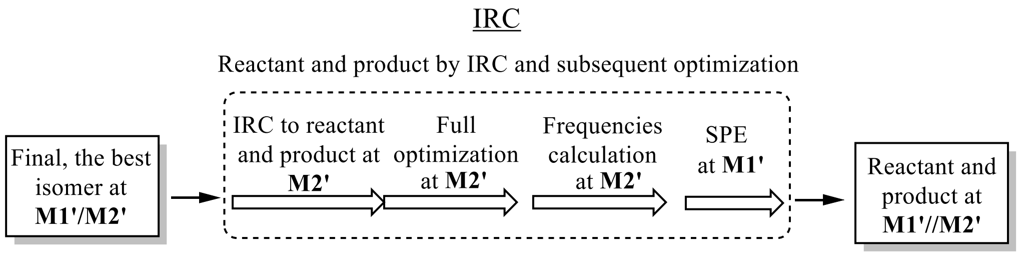

Figure S3. IRC and the following DFT calculations for evaluation of the respective reactant and product.

					Grids			
Name	Software	Functional	Basis Set	Solvent	for Metal	for others	Approx.	other
M1	Orca 4.2	wb97xd3	def2-tzvp	SMD(DMF)	7/5	3/4	COSJX	
M2	Orca 4.2	BP86-D3bj	def2-svp	SMD(DMF)	6/6	3/4	RIJ	LooseOpt
M3	Orca 4.2	BP86-D3bj	def2-svp	SMD(DMF)	6/6	3/4	RIJ	
M3`	Gaussian 09	BP86-D3bj	def2-tzvp, SDD	SMD(DMF)	Default	Default		
M1'	Gaussian 09	wb97xd	def2-tzvp, SDD	SMD(DMF)	Default	Default		
M3	Orca 4.2	BP86-D3bj	def2-svp	SMD(DMF)	6/6	3/4	RIJ	
M0	CREST	CREST, GFNn-xTB, parameters: Ewin = 60 kcal/mol**Error! Bookmark not defined.**, the rest are default.						

Table S2. Methods and computational levels of theory.

Why the multi-phase is necessary?

Despite the very high efficiency of generation of large ensembles of isomers by CREST, its semi-empiric GFNn-xTB method is not accurate enough relative to DFT and relative energies and positions of isomers within ensembles obtained by CREST and DFT can differ significantly. Figure S4 demonstrates 38 isomers obtained and optimized by CREST (red) and the same ensemble optimized by DFT (blue); for both methods isomers indexed by their energies relative to the lowest one. Important, index of the same isomer, obtained by CREST can be changed significantly after treating by DFT. Figure S4 illustrates three examples of such displacements: 1-->8, 7-->1 and 14-->2, where the first index is an initial, obtained by CREST, and the second one is final, obtained by DFT. Noteworthy, due to 7-->1 displacement, if only six first isomers were chosen from the initial ensemble by CREST for further study, the best isomer with initial index 7 (and the final 1) would be lost. In addition, if the number of chosen isomers would be less than 14, the second best isomer would be lost (due to 14-->2 displacement). To solve the issue, we suggest in our approach to choose isomers of CREST ensembles within the broad energy window of 10[4] kcal/mol (20-40 isomers, depending on the degree of freedom of compound) for preliminary optimization in phase 2 with "LooseOpt" keyword, as implemented in Orca4. Further choice among partly optimized isomers with new indexes is much safer, as indexes are nearly final.[1] Therefore, for the bottleneck phase 3 (due to its expensive frequency calculations) can safely be chosen ca. 6 isomers. We choose isomers using free energy window of 1.4 kcal/mol (6-10 conformers).

[4] Comparison of energy distribution within ensembles by CREST and by DFT (for different intermediates) revealed that the energy gap of 10 kcal/mol in CREST-ensembles corresponds to the 2.5-6 kcal/mol in DFT-ensemble (in the case presented by Figure S4 it is ca. 6 kcal/mol). Final window of 2.5 kcal/mol is more than enough for high level of confidence that the important isomers were not lost. Moreover, the final 2.5 kcal/mol is even enough for using Boltzmann weighted averages (future plans), since 2.5 kcal/mol corresponds to the equilibrium of 1:58.

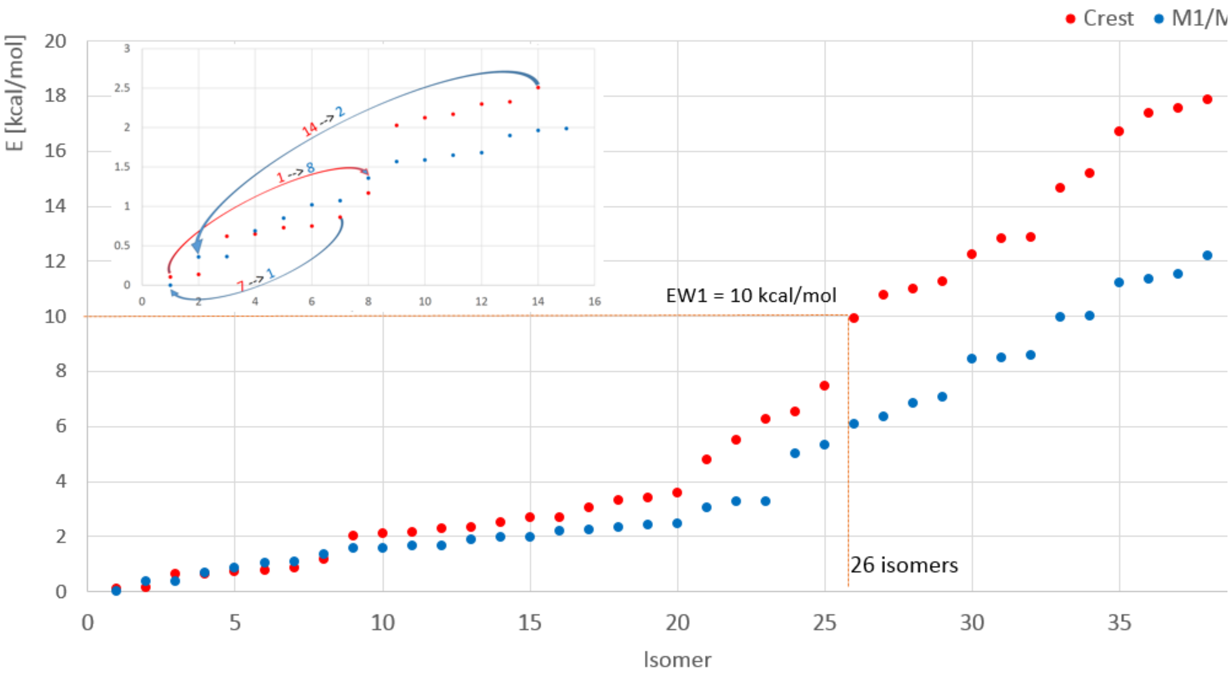

Figure S4. Isomers of **A1**, ordered by their relative energies obtained by CREST (red) and by DFT optimizations (blue); and examples of changed indexes (arrows up field) going from CREST to DFT results.

Recommendation - do not skip the optimizations!

Figure S5 demonstrates a typical geometry optimization graph which indicates that on 17th step the geometry is almost converged (within 0.05 kcal/mol), suggesting that after 17th step there should be no significant index displacements within an ensemble of isomers. This cause temptation to skimp twice on optimizations of an initially large ensemble of structures by restricting the number of optimization steps or, rather, by defining looser parameters of convergence criteria, as "LooseOpt" keyword we used. Nevertheless, more thorough analysis performed later revealed that there were geometry optimizations with slow convergence. In such cases optimizations with loose convergence criteria were finished significantly far away from the final structure (Figure S6), leading to underestimation of the final energy of the isomer by 0.7 kcal/mol or even more. Typical converging behavior of the whole ensemble to its final state is demonstrated in Figure S7. To validate our results, obtained by our "skimp approach", all key structures were fully optimized to obtain final states of the ensembles. Fortunately, there were no displacements of the type n$^{[\text{partly optimized ensemble}]}$ --> 1$^{[\text{fully optimized ensemble}]}$ (n>1), so the best conformers were not changed and the final PESs were not affected. <u>Nevertheless, we conclude that **full** optimization for the initial ensemble is strongly recommended before selecting the best isomers for further treatment.</u>

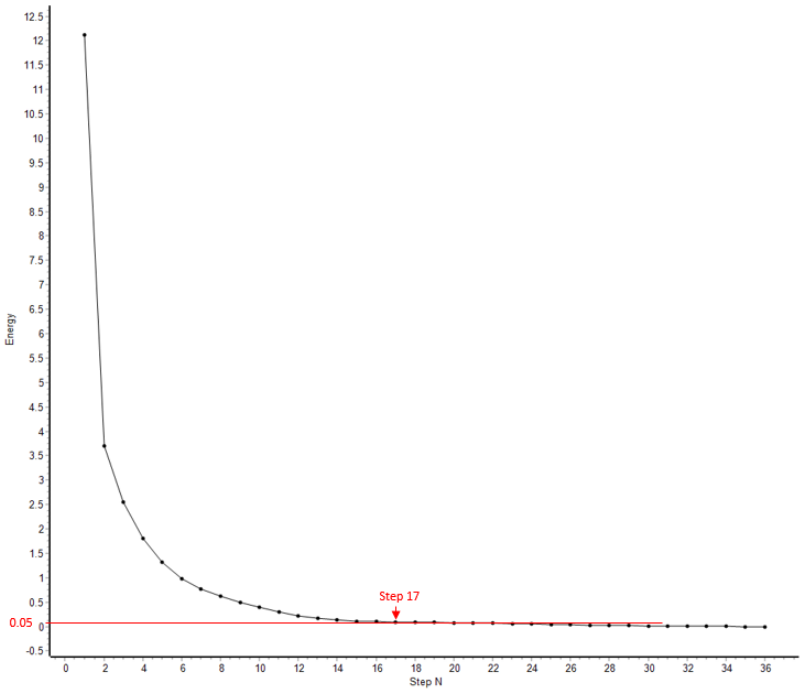

Figure S5. Typical optimization of geometry.

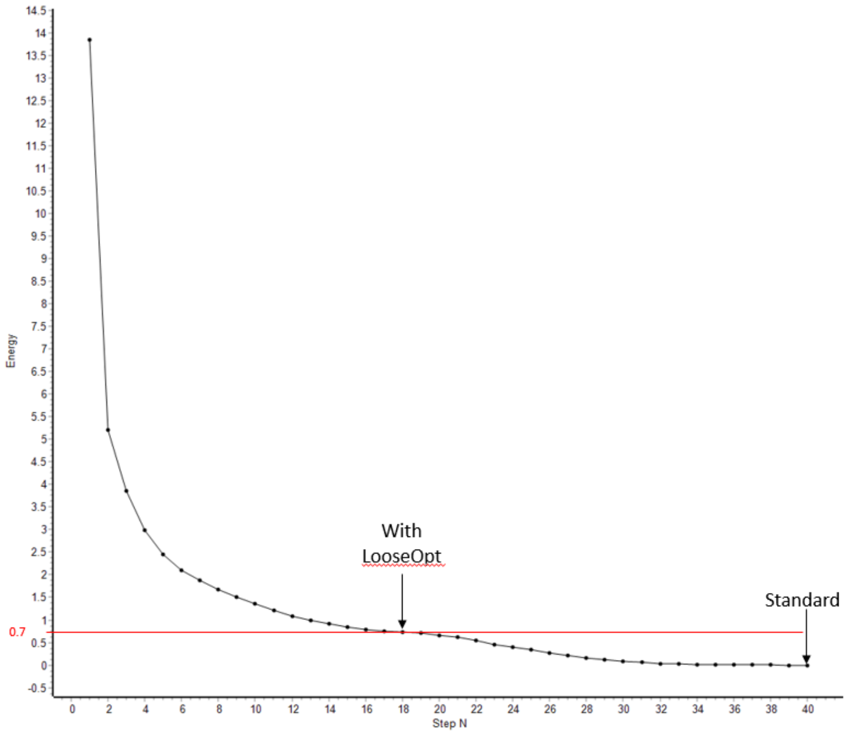

Figure S6. Slowly converging optimization.

It can be concluded from the Figure S7 that convergence of the ensemble to its final state (final order of isomers arranged by their energies) proceed slowly: displacements by more than 15 positions within the ensemble (blue graph) occurs till step 21; and the rest of displacements: by more than 10, 5 and 1 positions occur till the steps 28, 39 and 40 respectively.

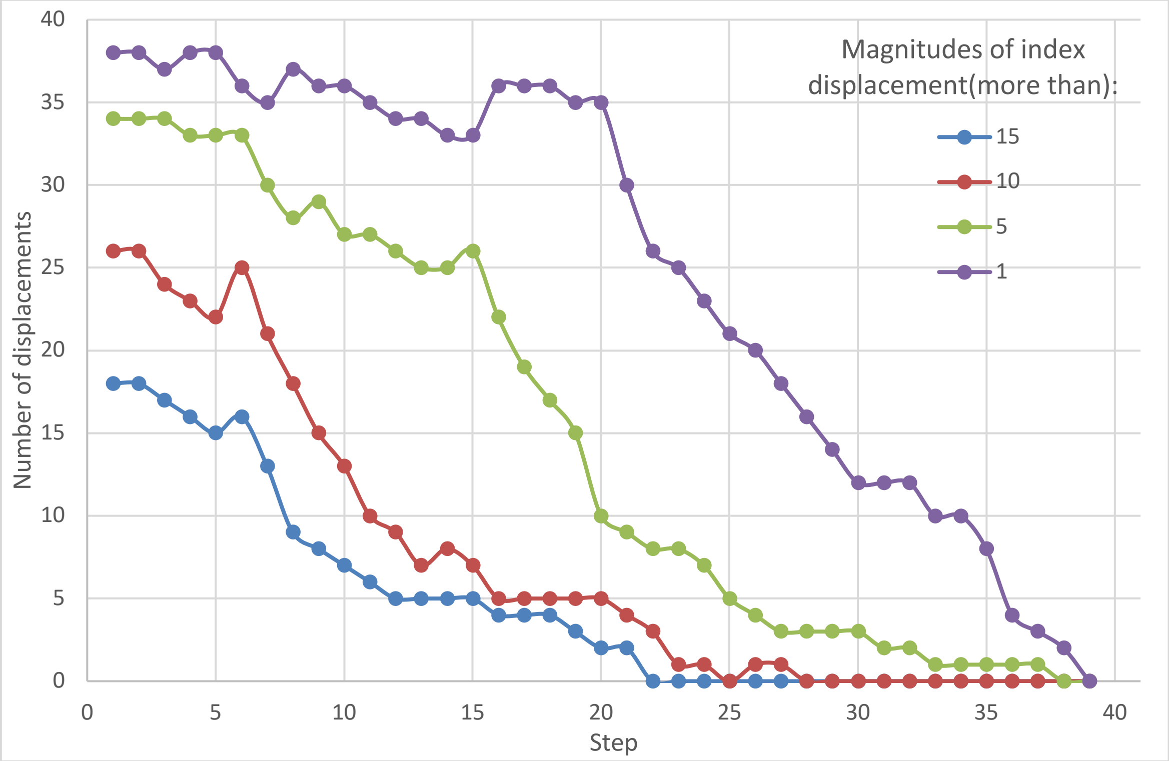

Figure S7. Number of displacements (of various magnitudes) within the ensemble as a function of optimization step. The ensemble contains 62 isomers for compound **A3** obtained by CREST. For example: orange graph is the number of displacements (number of structures that changed their index relative to the final) by more than 10 positions within the ensemble.

Recommended workflow to obtain the best isomer

Based on the experience of the current study and some analysis partly presented above we can suggest simplified and robust workflow for the search of the best (lowest energy) isomer (Scheme S3), where the most expensive part of full optimization of large ensemble of isomers (phase 2), which proceeds for 20-24 hours on 16 CPUs at the M2 level of theory (ca. 40 isomers).

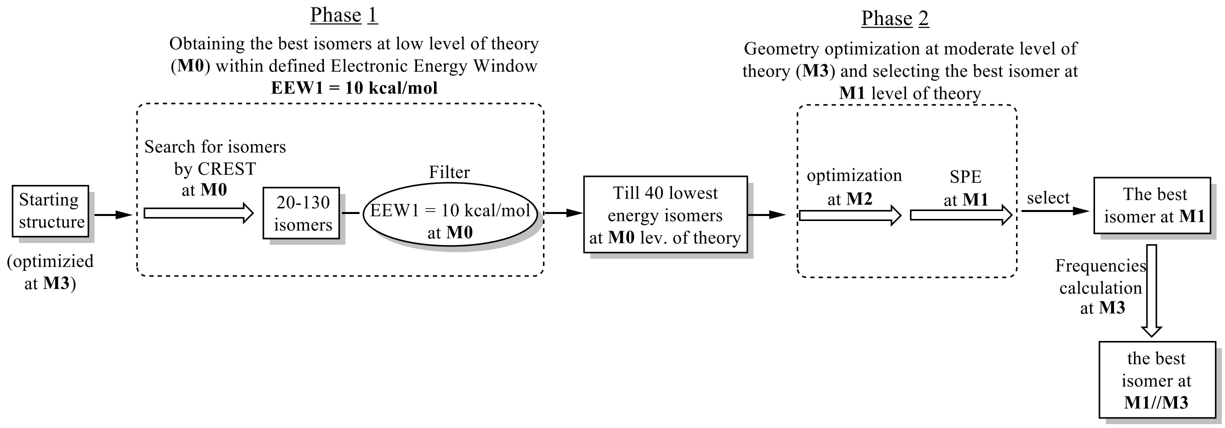

Scheme S3. Recommended workflow for the search of the best isomer.

Calculated reaction mechanisms of carbinol 1

Migratory insertion.

Synopsis. Stabilizing conjugation between cyclopropyl and double bond in **TS1s-trans** (coordination from face 1 of the double bond) is more effective than in **TS1s-cis** (coordination from face 2), making the former lower in energy and leading to the selective migratory insertion via **TS1s-trans** onto face 1. Structural constraints in **TS1s-cis** are caused by HO-Pd coordination when Pd coordinated from face 2 of the double bond.

To understand the origin of the difference in energy E(**TS1s-cis** - **TS1s-trans**) = 2.2 kcal/mol, we studied their geometries and the geometries of their respective preceding intermediates **1s-cis** and **1s-trans**. We found that these transition states, being early, resemble their respective intermediates, having the energy difference between them of ΔG = 2.5 kcal/mol in favour of **1s-trans**, which is similar to the inherited energy difference between their respective transition states (2.2 kcal/mol). The key structural difference within both pairs in their dihedral angles 4-5-1-6: 69° vs 8° for **1s-cis** and **1s-trans**, and 69° vs 14° for **TS1s-cis** and **TS1s-trans** respectively (Scheme S4, Newman projections). Further, we studied intermediates **1s-cis** and **1s-trans**, assuming that the comparison between them reflects the differences between **TS1s-cis** and **TS1s-trans**.

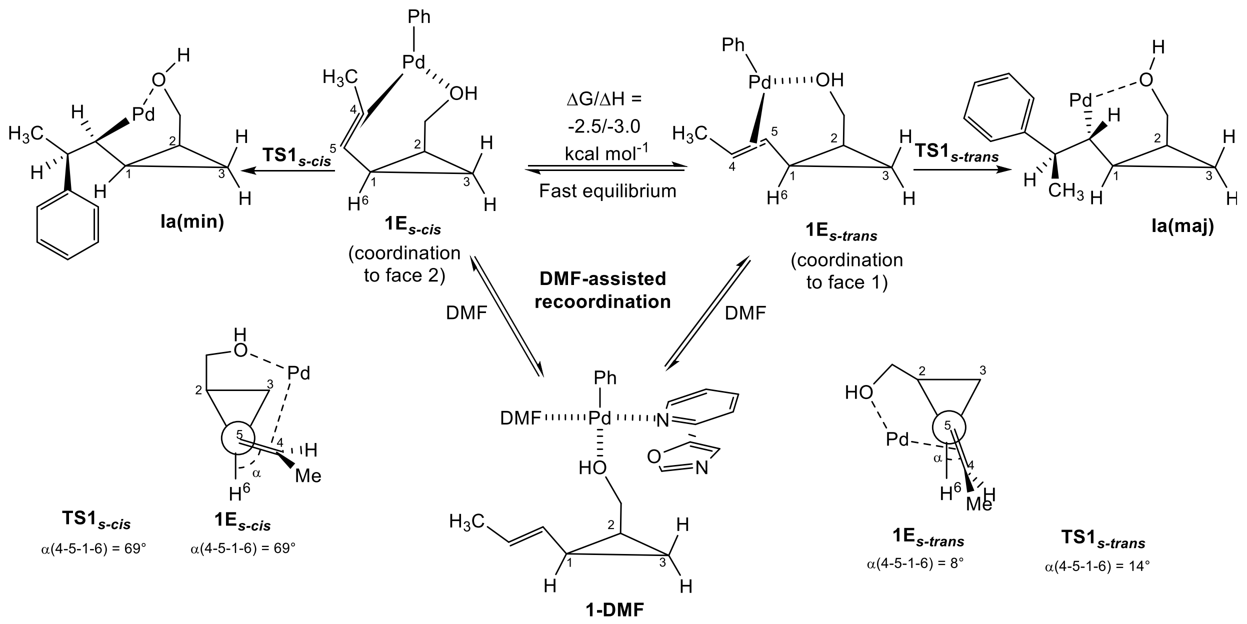

Scheme S4. Insertion step from two faces and Newman projection of respective reactants.

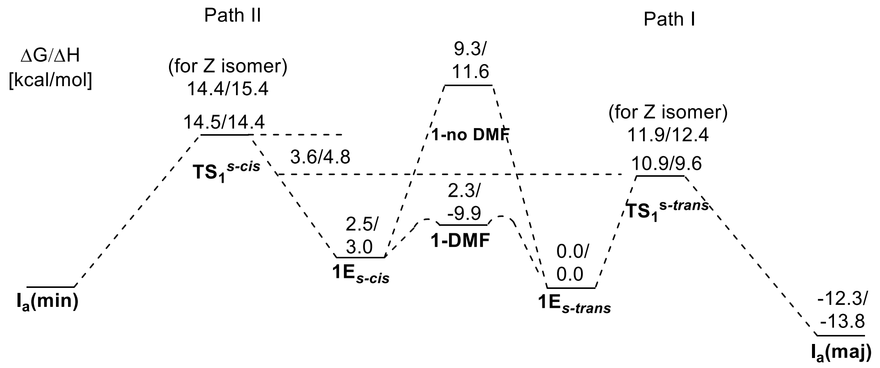

Figure S8. Potential energy surface of insertion step from two faces

Previously it was experimentally found and supported by calculations that conjugation between cyclopropyl and the π system of the double bond most effective at bisected antiperiplanar conformation (φ = 0°) (Figure S9, a) and less effective at synclinal with φ = 65° (Figure S9, b).[22–24] Our calculations for the parent system **1'** and for the fragment of real cyclopropyl unit **1** (without [Pd] unit) are in line with previous studies[22–24] indicating the validity of theoretical method (see potential energy surface on Figure S10). For the parent system **1'** the energy difference between two conformers with the angles corresponding to those in $\mathbf{1E_{s\text{-}cis}}$ and $\mathbf{1E_{s\text{-}trans}}$ is 2.7 kcal/mol, while for the real fragment **1** it is even slightly greater, 4.6 kcal/mol, probably, due to the repulsive interaction of vinyl substituent with methanol unit which is absent in **1'**. Moreover, unleashing Pd in $\mathbf{1E_{s\text{-}cis}}$, by substitution of chelating CH_2OH group by hydrogen atom on position C^2 in $\mathbf{1E_{s\text{-}cis}}$ (compound $\mathbf{1E_{s\text{-}cis}'}$) and subsequent geometry relaxation resulted in change of the φ from 69° to 11° in $\mathbf{1E_{s\text{-}trans}'}$ with relaxation energy of 6 kcal/mol (Scheme S5). These findings

indicate that the main reason for destabilization of **1E$_{s\text{-}cis}$** and **TS1s-cis** relative to **1s-trans** and **TS1s-trans**, respectively, is reduced effectiveness of conjugation between cyclopropyl and the double bond in the former pair caused by constraining HO—Pd chelation when Pd coordinated to the face 2.

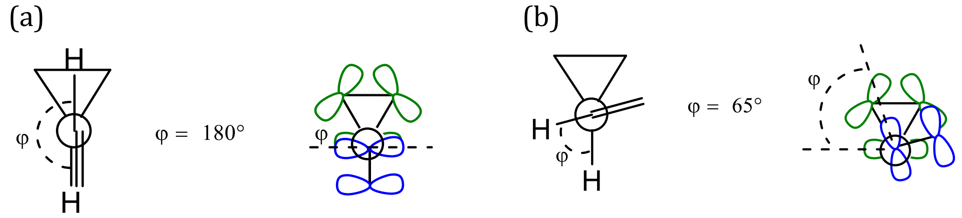

Figure S9. Two conformational minima: (a) antiperiplanar with φ = 0°; and (b) synclinal with φ = 65°

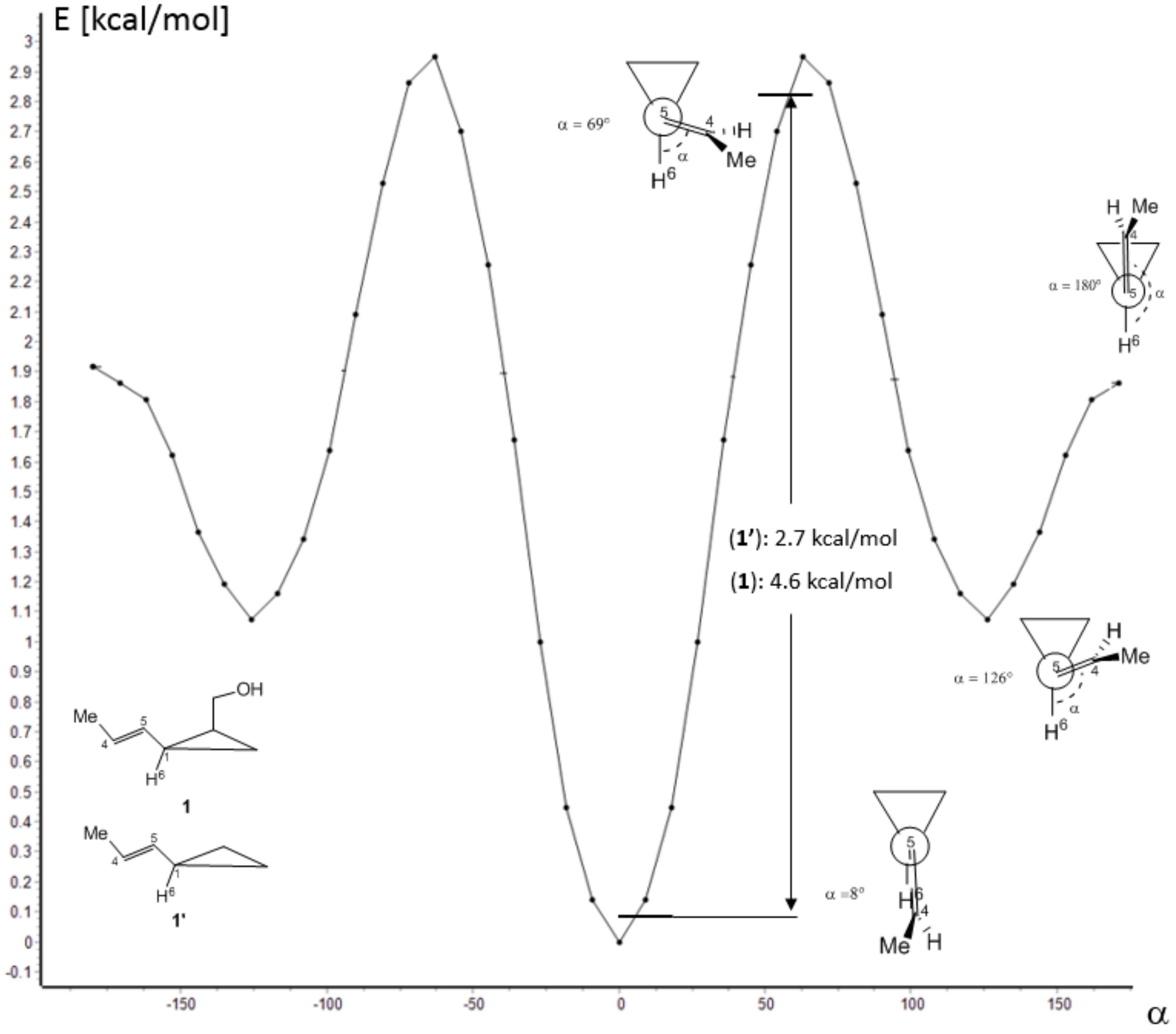

Figure S10. Potential energy surface (electronic energy) of **1'** with respect to the dihedral angle α(4-5-1-6)

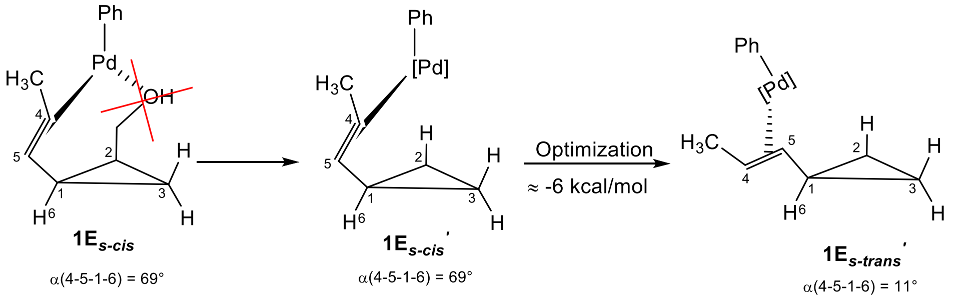

Scheme S5. Structure **1E$_{s\text{-}cis}$'** is obtained from the geometry of **1E$_{s\text{-}cis}$** by substitution of CH_2OH with hydrogen atom (on position C[2]) for the following geometry optimization.

Path A

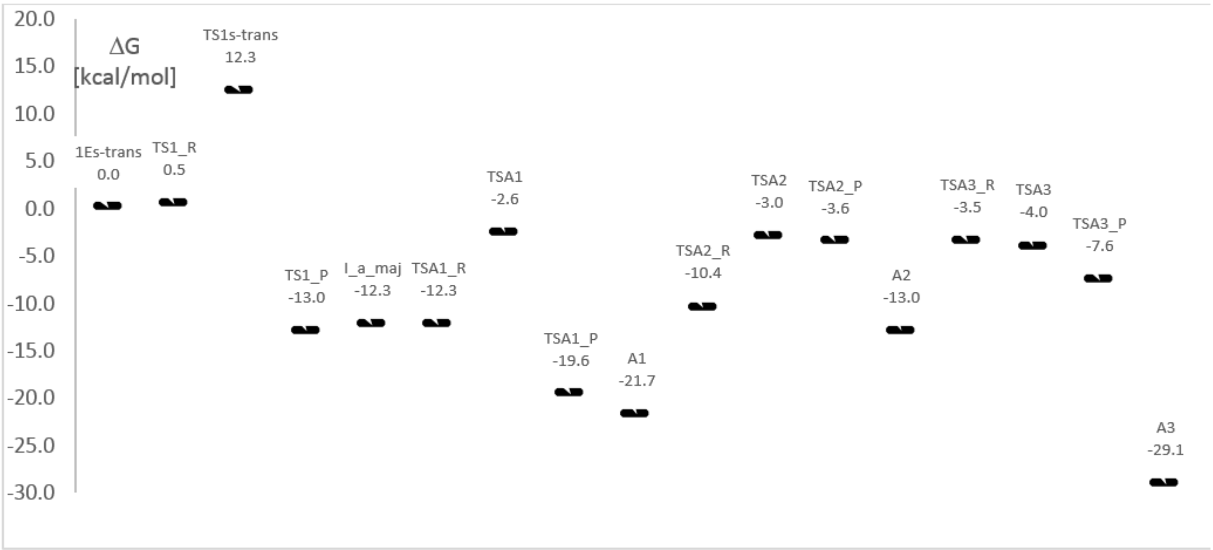

Figure S11. Complete PES of path A

Path A

(complete scheme)

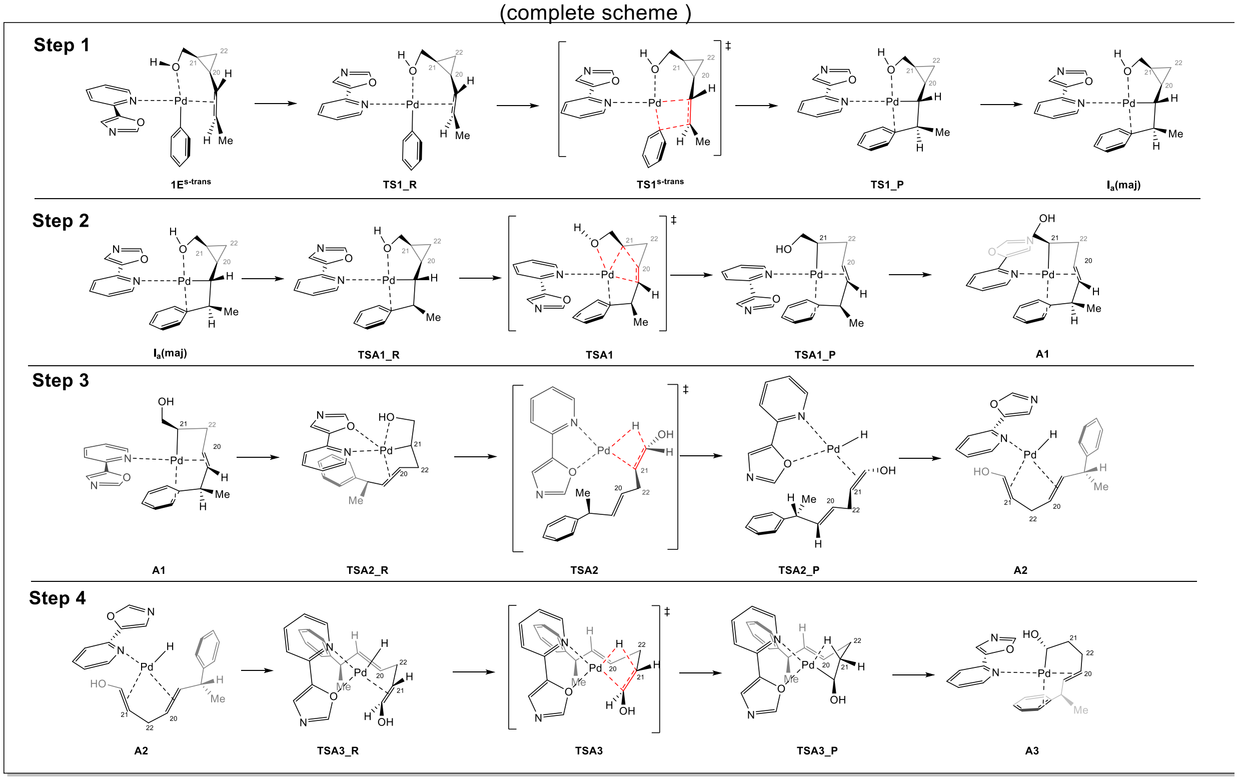

Scheme S6. Structures of all the lowest energy intermediates, transition states with their respective reactants and products of path A.

	$1E^{s-trans}$	TS1_R	$TS1^{s-trans}$	TS1_P	Ia(maj)	TSA1_R	TSA1	TSA1_P	A1	TSA2_R	TSA2	TSA2_P	A2	TSA3_R	TSA3	TSA3_P	A3
ΔE	0.0	-0.6	10.6	-14.2	-14.2	-14.2	-3.6	-19.6	-21.0	-12.2	-1.6	-2.5	-10.9	-3.5	-1.8	-7.4	-31.3
ΔH	0.0	-0.4	9.0	-13.8	-13.8	-13.8	-4.4	-19.5	-20.9	-11.8	-4.1	-3.6	-12.4	-4.5	-4.3	-8.2	-30.6
ΔG	0.0	0.5	12.3	-13.0	-12.3	-12.3	-2.6	-19.6	-21.7	-10.4	-3.0	-3.6	-13.0	-3.5	-4.0	-7.6	-29.1

Table S3. Electronic energies (ΔE), enthalpies (ΔH) and free energies (ΔG) relative to the $\mathbf{1E^{s\text{-}trans}}$

Path B

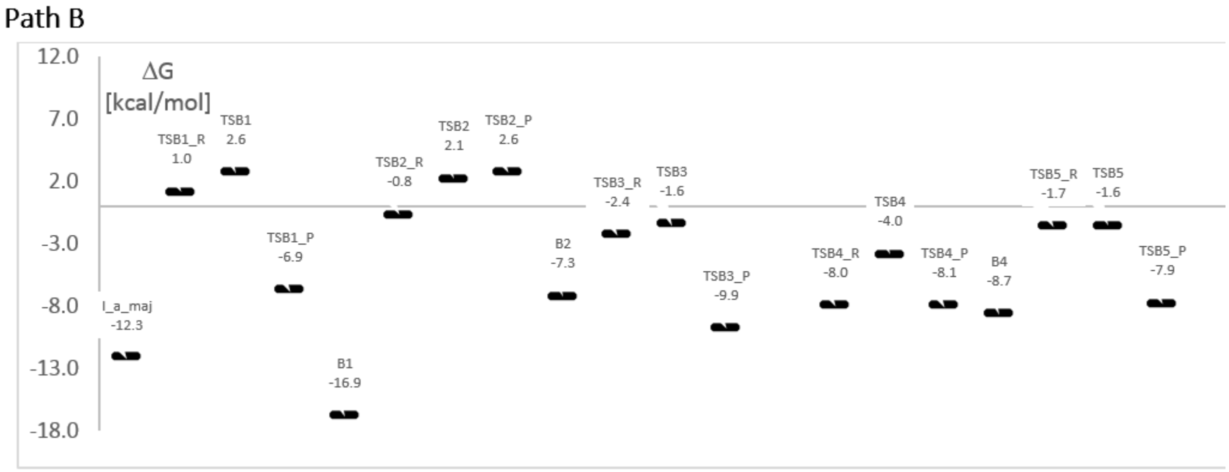

Figure S12. Complete PES of path B

Path B
(complete scheme)

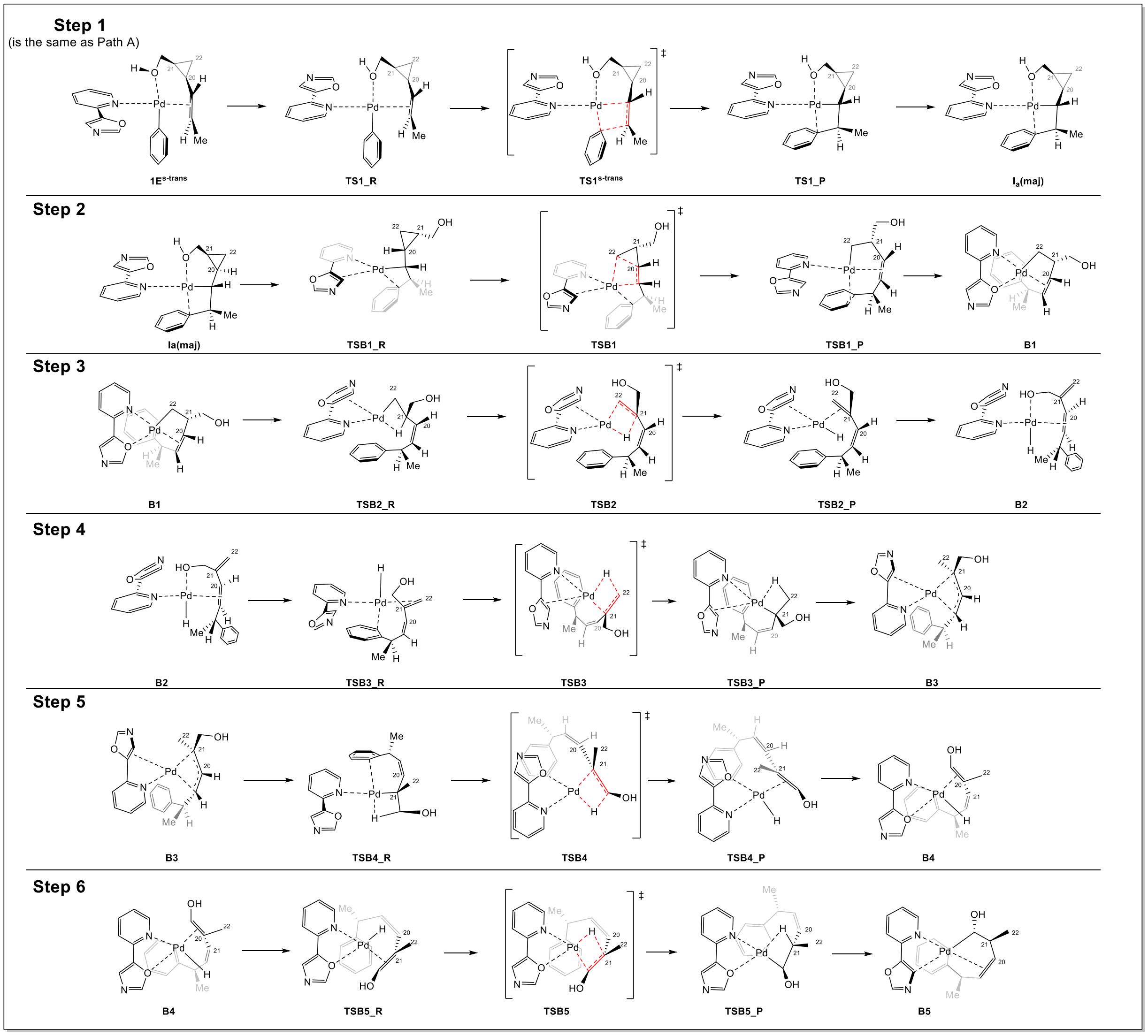

Scheme S7. Structures of all the lowest energy intermediates, transition states with their respective reactants and products of path A.

	Ia(maj)	TSB1_R	TSB1	TSB1_P	B1	TSB2_R	TSB2	TSB2_P	B2	TSB3_R	TSB3	TSB3_P
ΔE	-14.2	0.6	2.0	-9.0	-18.6	0.8	4.5	3.7	-4.6	-2.0	-0.2	-9.2
ΔH	-13.8	0.6	1.3	-8.6	-18.0	-0.9	1.1	1.6	-5.8	-3.3	-2.7	-10.2
ΔG	-12.3	1.0	2.6	-6.9	-16.9	-0.8	2.1	2.6	-7.3	-2.4	-1.6	-9.9

B3	TSB4_R	TSB4	TSB4_P	B4	TSB5_R	TSB5	TSB5_P	B5
-27.9	-5.7	-1.8	-8.0	-8.5	-0.6	-0.3	-7.1	-24.7
-27.8	-7.5	-4.9	-9.3	-9.7	-2.4	-3.2	-8.4	-24.5
-27.5	-8.0	-4.0	-8.1	-8.7	-1.7	-1.6	-7.9	-25.0

Table S4. Electronic energies (ΔE), enthalpies (ΔH) and free energies (ΔG) relative to the $\mathbf{1E}^{s\text{-}trans}$

Ring opening step and variation of the nature and effect of the R group on the reaction barrier

(a)

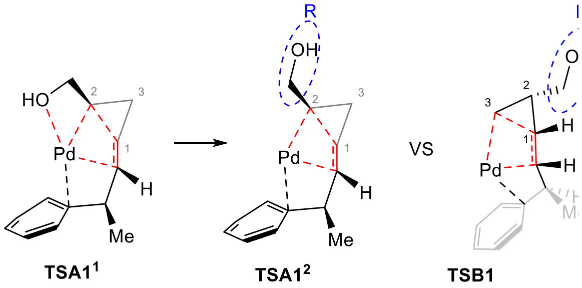

(b)

Entry (i)	R	ΔG(**TSB$_1^i$**- **TSA$_1^i$**) [kcal/mol]
1	-CH_2OH (HO→Pd)	4.9 (5.2)
2	-CH_2OH (no HO→Pd)	3.7
3	-Me	3.0
4	-H	2.0

(c)

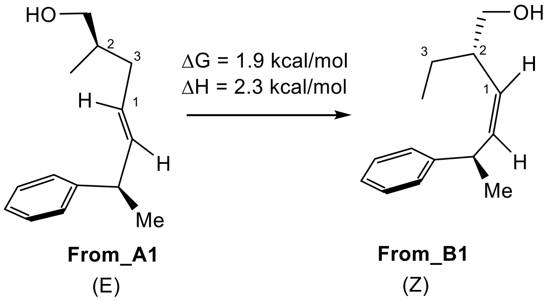

Figure S13. (a) Ring opening structures of the transition states **TSA$_1^1$**, **TSA$_1^2$** (without O-Pd coordination) and **TSB$_1$**; (b) relative energies ΔG(**TSB$_1$**-**TSA$_1^i$**) [kcal/mol] as a function of substituent R; (c) Relative energies of E- and Z-isomers ("dissociated" from Pd complex).

Reaction mechanisms of diols

Migratory insertion

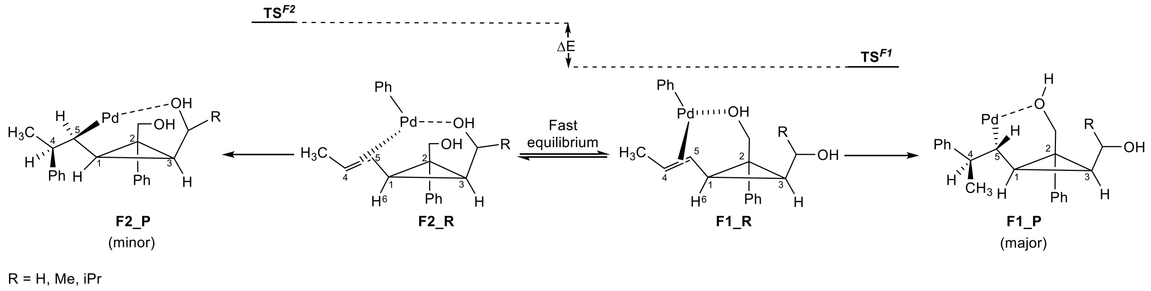

Scheme S8. Migratory insertion on two faces of the double bond

R	H	Me[R]	Me[S]	iPr[R]	iPr[S]
ΔE	1.0	0.9	0.8	3.7	1.4
ΔH	1.2	0.7	1.7	4.0	1.4
ΔG	1.7	2.7	1.5	4.5	2.5

Table S5. Migratory insertion on two faces of the double bond for different substituents R

Ring opening

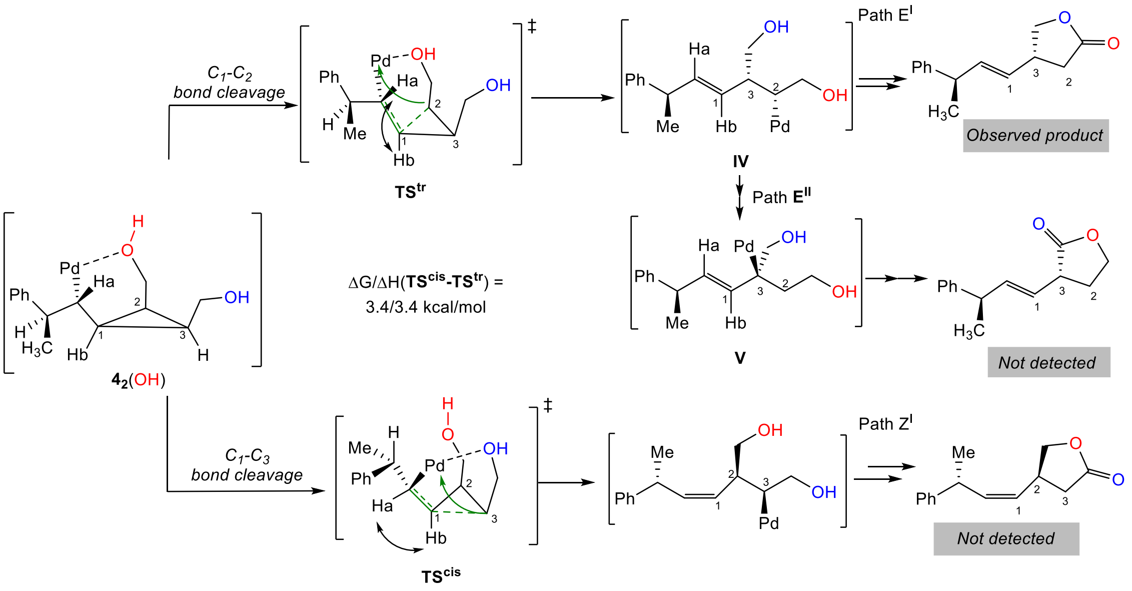

Scheme S9. Ring opening transition states and following final products.

References

(1) Drent, E.; Van Broekhoven, J. A. M.; Doyle, M. J. Efficient Palladium Catalysts for the Copolymerization of Carbon Monoxide with Olefins to Produce Perfectly Alternating Polyketones. *J. Organomet. Chem.* **1991**, *417*, 235–251.

(2) Liao, L. A.; Fox, J. M. A Copper-Catalyzed Method for the Facially Selective Addition of Grignard Reagents to Cyclopropenes. *J. Am. Chem. Soc.* **2002**, *124*, 14322–14323.

(3) Liu, X.; Fox, J. M. Enantioselective, Facially Selective Carbomagnesation of Cyclopropenes. *J. Am. Chem. Soc.* **2006**, *128*, 5600–5601.

(4) Cohen, A.; Chagneau, J.; Marek, I. Stereoselective Preparation of Distant Stereocenters (1,5) within Acyclic Molecules. *ACS Catal.* **2020**, *10*, 7154–7161.

(5) Phan, D. H. T.; Kou, K. G. M.; Dong, V. M. Enantioselective Desymmetrization of Cyclopropenes by Hydroacylation. *J. Am. Chem. Soc.* **2010**, *132*, 16354–16355.

(6) Bruffaerts, J.; Vasseur, A.; Marek, I. Alkene-Zipper Catalyzed Selective and Remote Retro-Ene Reaction of Alkenyl Cyclopropylcarbinol. *Adv. Synth. Catal.* **2018**, *360*, 1389–1396.

(7) Hansen, T. M.; Florence, G. J.; Lugo-Mas, P.; Chen, J.; Abrams, J. N.; Forsyth, C. J. Highly Chemoselective Oxidation of 1,5-Diols to δ-Lactones with TEMPO/BAIB. *Tetrahedron Lett.* **2003**, *44*, 57–59.

(8) Liu, Y.; Kaiser, A. M.; Arndtsen, B. A. Palladium Catalyzed Carbonylative Generation of Potent, Pyridine-Based Acylating Electrophiles for the Functionalization of Arenes to Ketones. *Chem. Sci.* **2020**, *11*, 8610–8616.

(9) Neese, F. The ORCA Program System. *Wiley Interdiscip. Rev. Comput. Mol. Sci.* **2012**, *2*, 73–78.

(10) Frisch, Æ.; Plata, R. E.; Singleton, D. A. Gaussian 09W Reference. *J. Am. Chem. Soc.* **2009**, *137*, 3811–3826.

(11) Perdew, J. P. Erratum: Density-Functional Approximation for the Correlation Energy of the Inhomogeneous Electron Gas. *Phys. Rev. B* **1986**, *34*, 7406.

(12) Perdew, J. P. Correction. *Phys. Rev. B* **1986**, *34*, 7406.

(13) Kassel, L. S. The Limiting High Temperature Rotational Partition Function of Nonrigid Molecules: I. General Theory. II. CH4, C2H6, C3H8, CH(CH3)3, C(CH 3)4 and CH3(CH2)2CH 3. III. Benzene and Its Eleven Methyl Derivatives. *J. Chem. Phys.* **1936**, *4*, 276–282.

(14) Allouche, A. Software News and Updates Gabedit — A Graphical User Interface for Computational Chemistry Softwares. *J. Comput. Chem.* **2012**, *32*, 174–182.

(15) Weigend, F.; Ahlrichs, R. Balanced Basis Sets of Split Valence, Triple Zeta Valence and Quadruple Zeta Valence Quality for H to Rn: Design and Assessment of Accuracy. *Phys. Chem. Chem. Phys.* **2005**, *7*, 3297–3305.

(16) Marenich, A. V; Cramer, C. J.; Truhlar, D. G. Universal Solvation Model Based on Solute

Electron Density and on a Continuum Model of the Solvent Defined by the Bulk Dielectric Constant and Atomic Surface Tensions. *J. Phys. Chem. B* **2009**, *113*, 6378–6396.

(17) Izsák, R.; Neese, F. An Overlap Fitted Chain of Spheres Exchange Method. *J. Chem. Phys.* **2011**, *135*, 14405.

(18) K. Fukui, Formulation of the reaction coordinate, *The Journal of Physical Chemistry* **1970**, *74*, 4161-4163.

(19) K. Fukui, The path of chemical reactions - the IRC approach, *Accounts of Chemical Research* **1981**, *14*, 363-368.

(20) Pracht, P.; Bohle, F.; Grimme, S. Automated Exploration of the Low-Energy Chemical Space with Fast Quantum Chemical Methods. *Phys. Chem. Chem. Phys.* **2020**, *22*, 7169–7192.

(21) Bannwarth, C.; Ehlert, S.; Grimme, S. GFN2-XTB—An Accurate and Broadly Parametrized Self-Consistent Tight-Binding Quantum Chemical Method with Multipole Electrostatics and Density-Dependent Dispersion Contributions. *J. Chem. Theory Comput.* **2019**, *15*, 1652–1671.

(22) A. de Meijere, Bonding Properties of Cyclopropane and Their Chemical Consequences, *Angew. Chem. Int. Ed.* **1979**, *18*, 809-826.

(23) T. Haumann, R. Boese, S. I. Kozhushkov, K. Rauch, A. de Meijere, Structural Aspects of Cyclopropyl Conjugation: Experimental Studies and Ab Initio Calculations, *Liebigs Annalen* **1997**, *1997*, 2047-2053.

(24) Rademacher, P. Photoelectron Spectra of Cyclopropane and Cyclopropene Compounds. *Chem. Rev.* **2003**, *103*, 933–976.

NMR spectra of new compounds

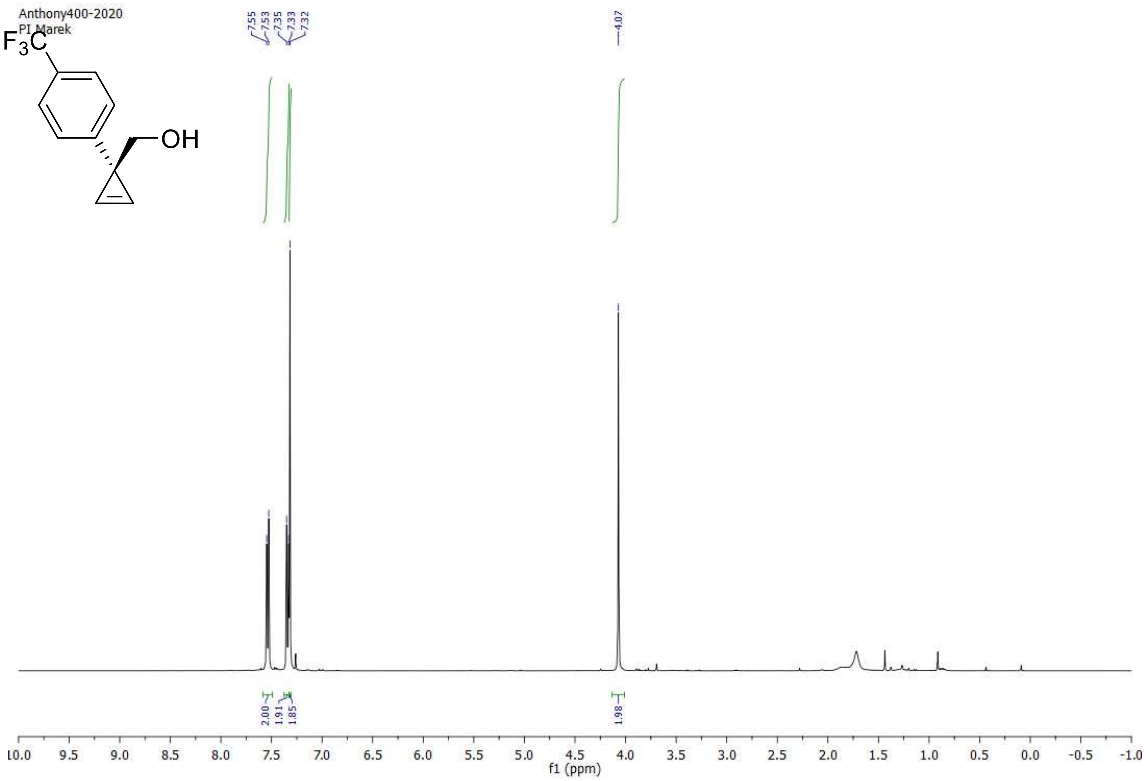

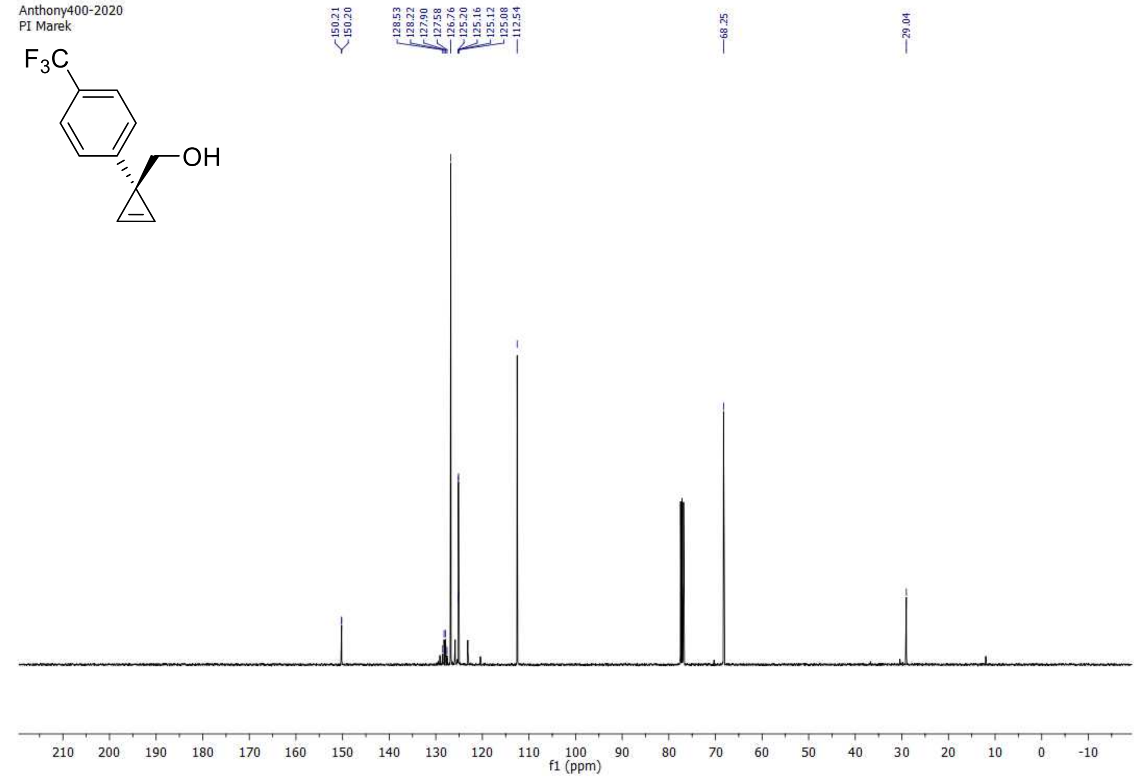

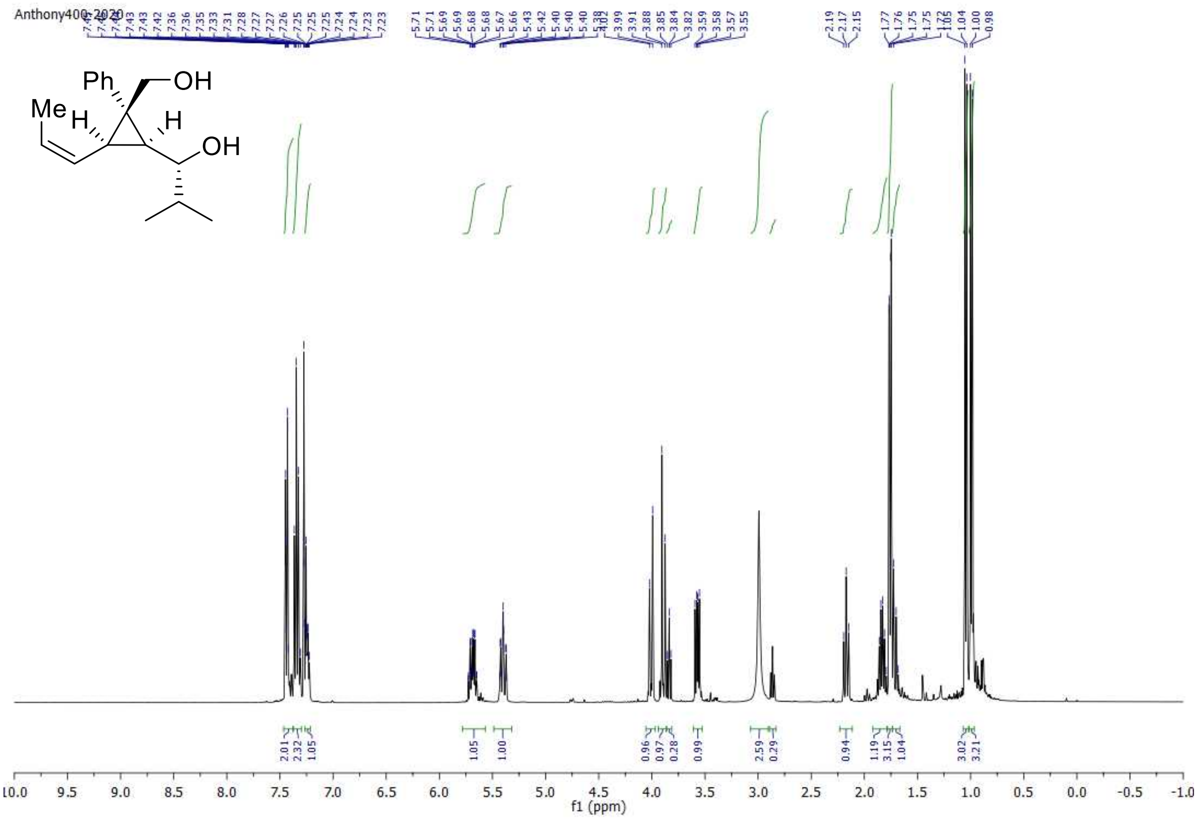

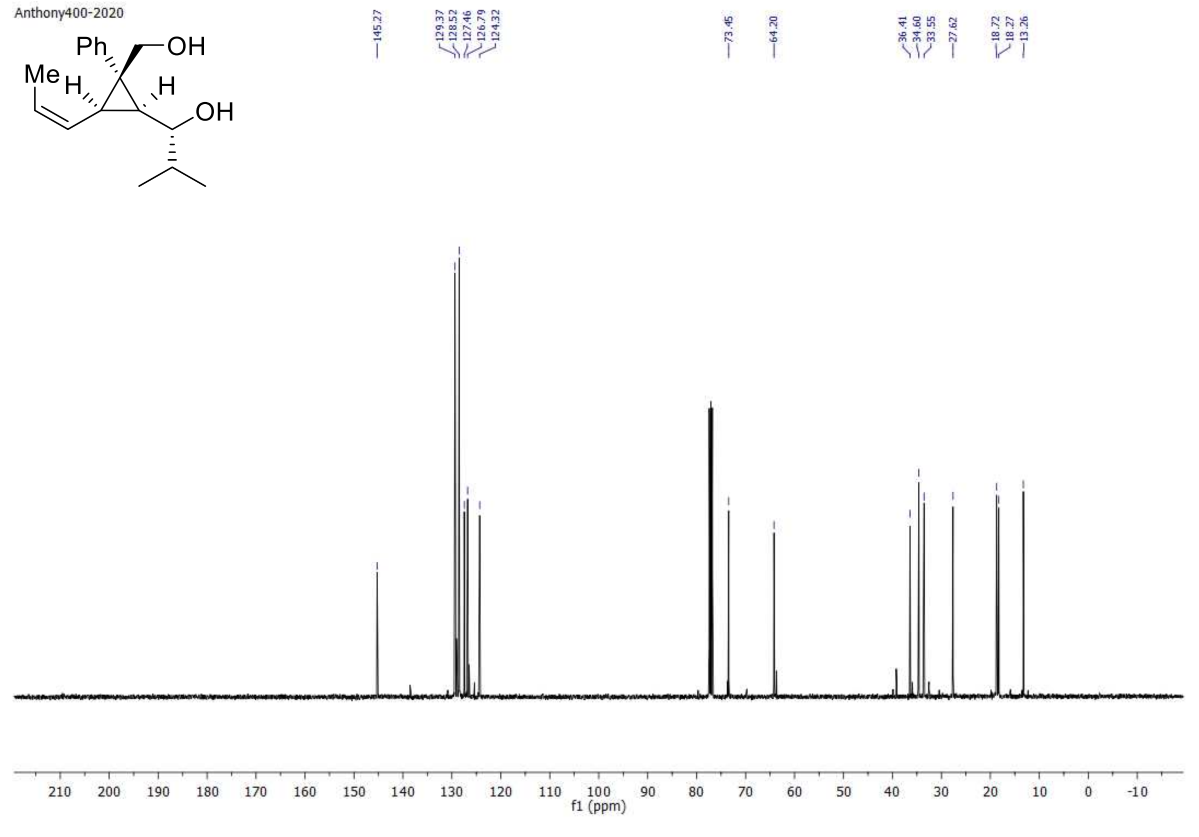

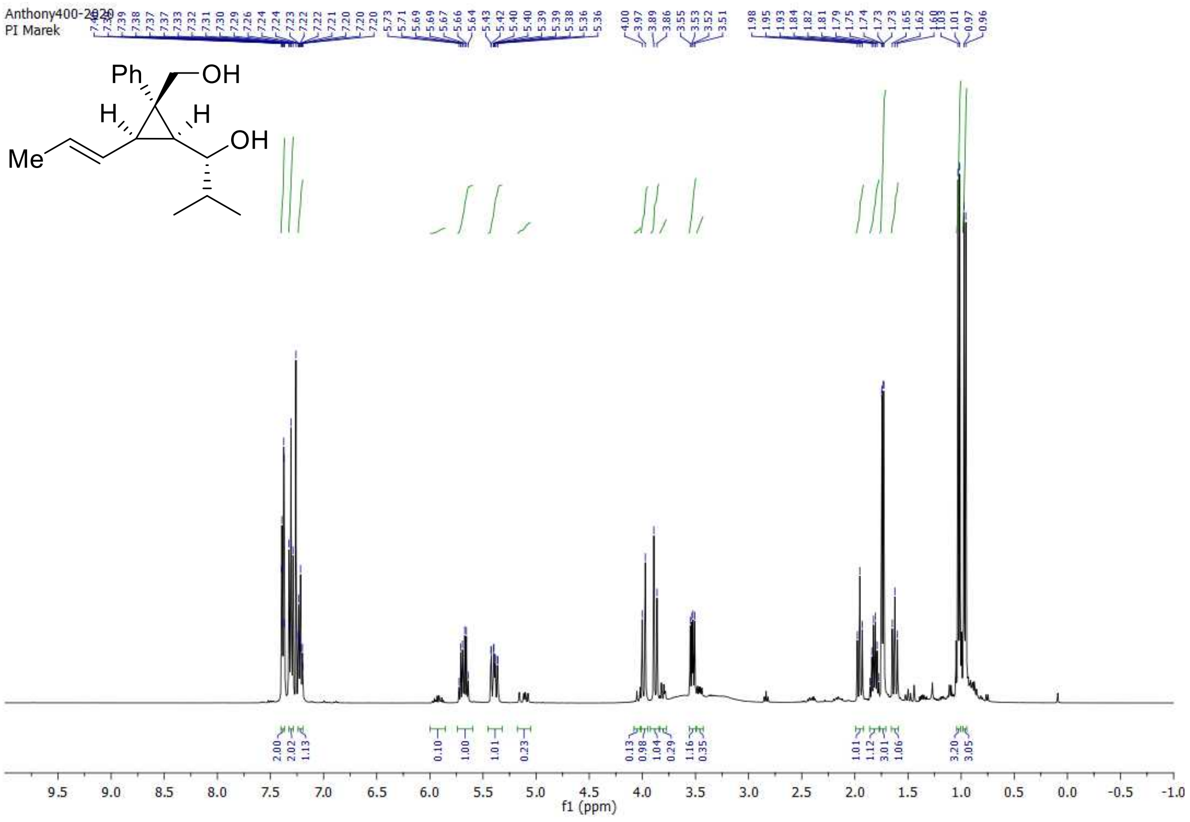

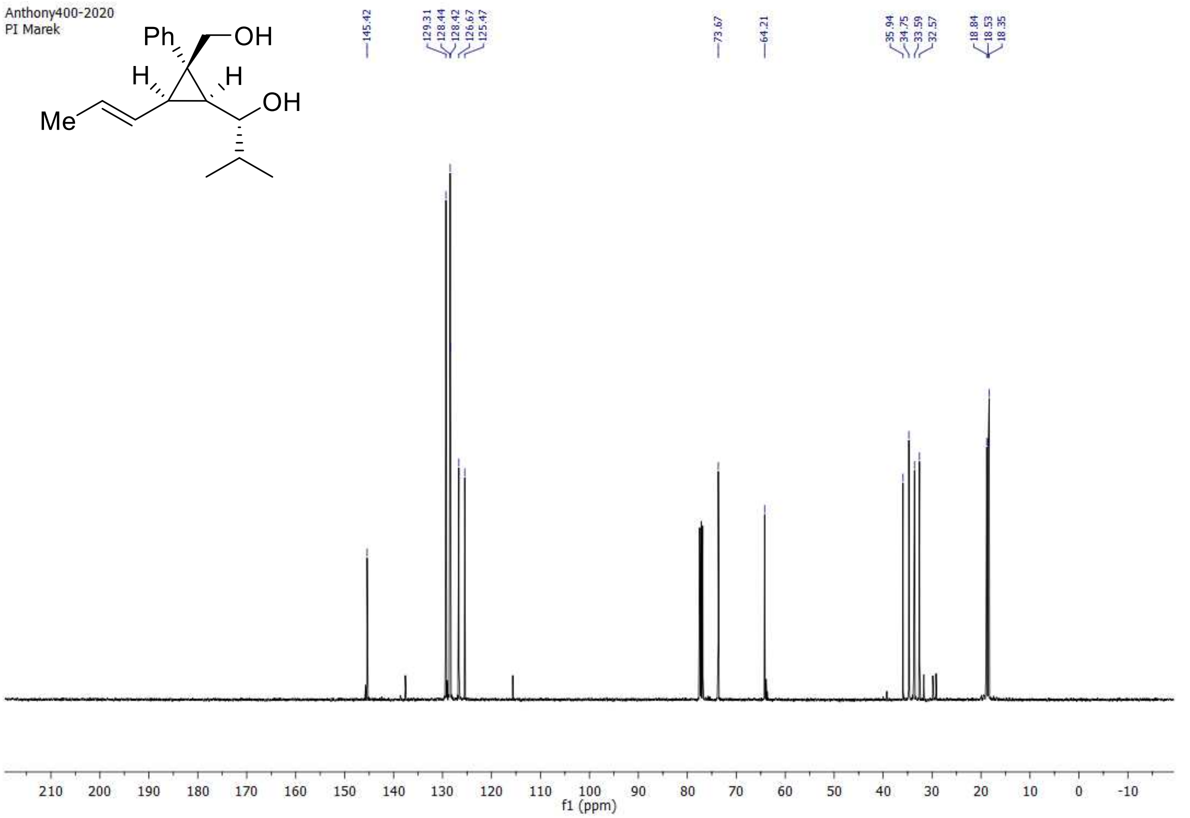

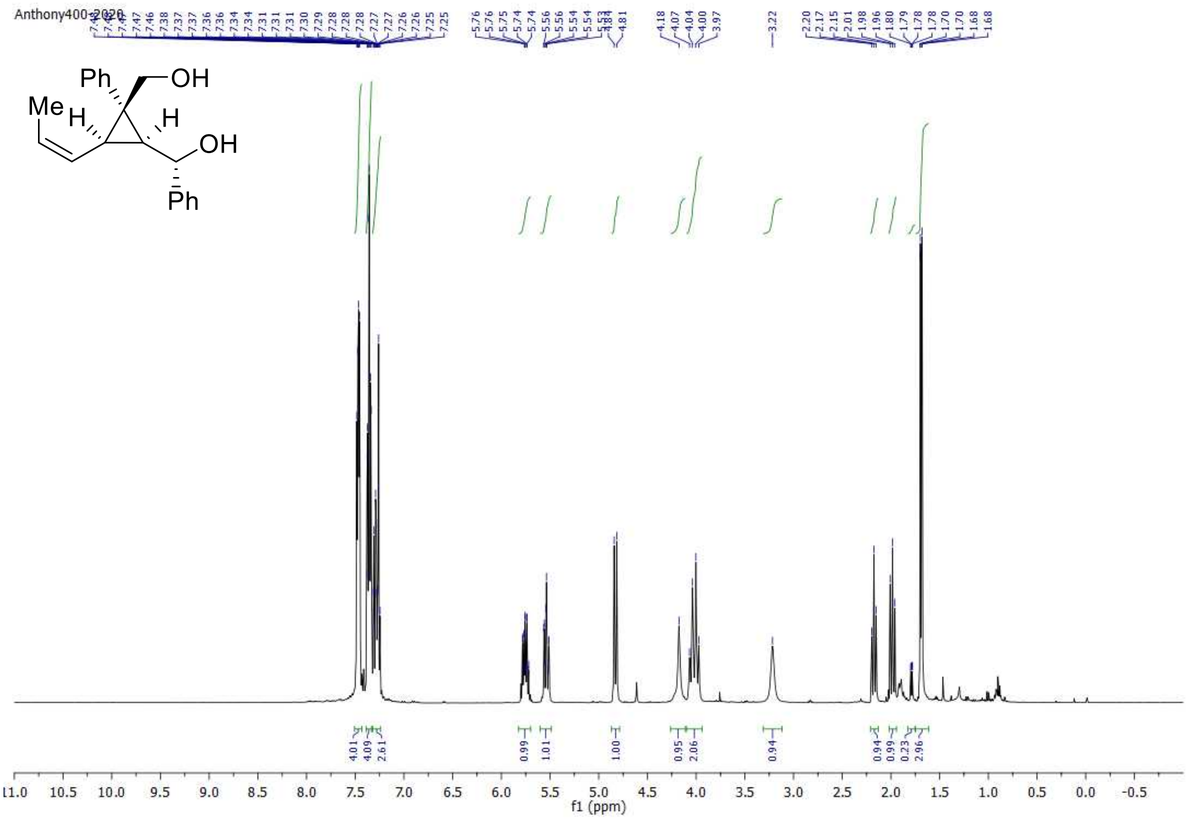
Anthony400-2020
Ph
OH
Me
H
H
OH
Ph
f1 (ppm)

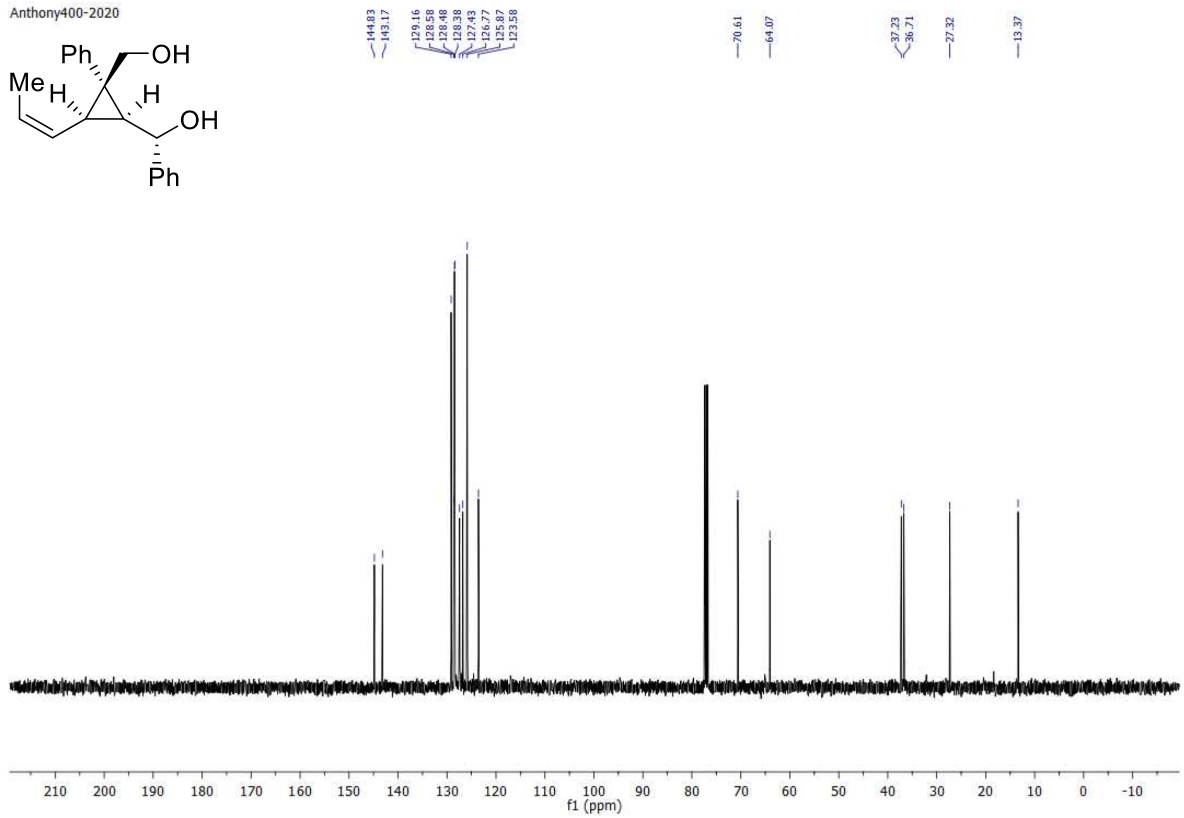
Anthony400-2020
144.83
143.17
129.16
128.58
128.48
128.38
127.43
126.77
125.87
123.58
70.61
64.07
37.23
36.71
27.32
13.37
Ph
OH
Me
H
H
OH
Ph
f1 (ppm)

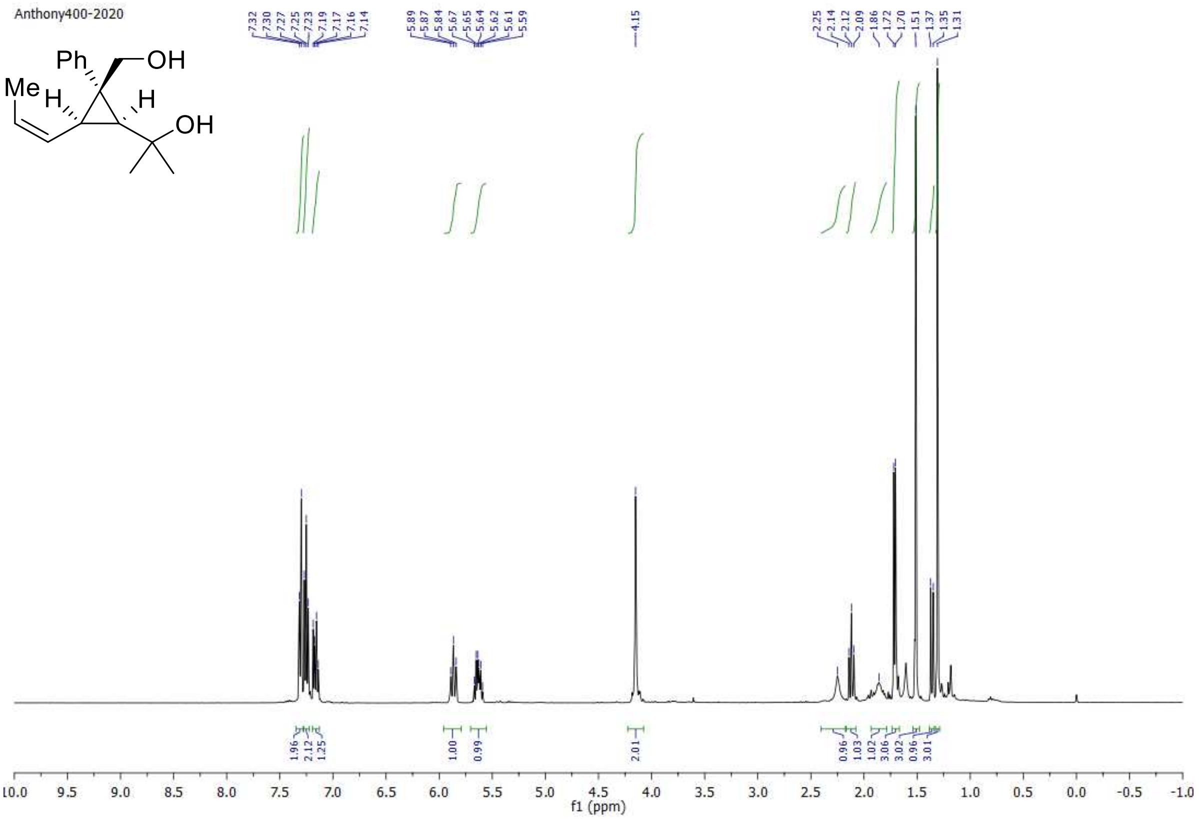

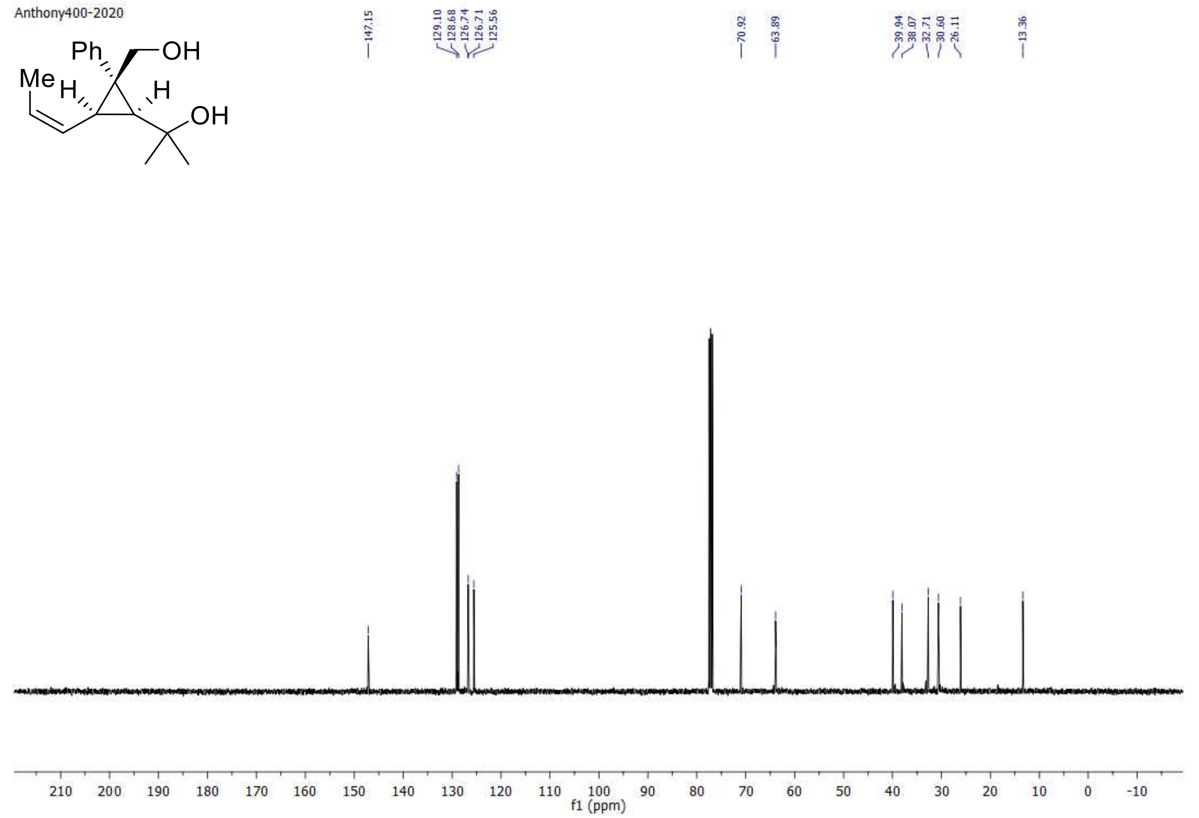

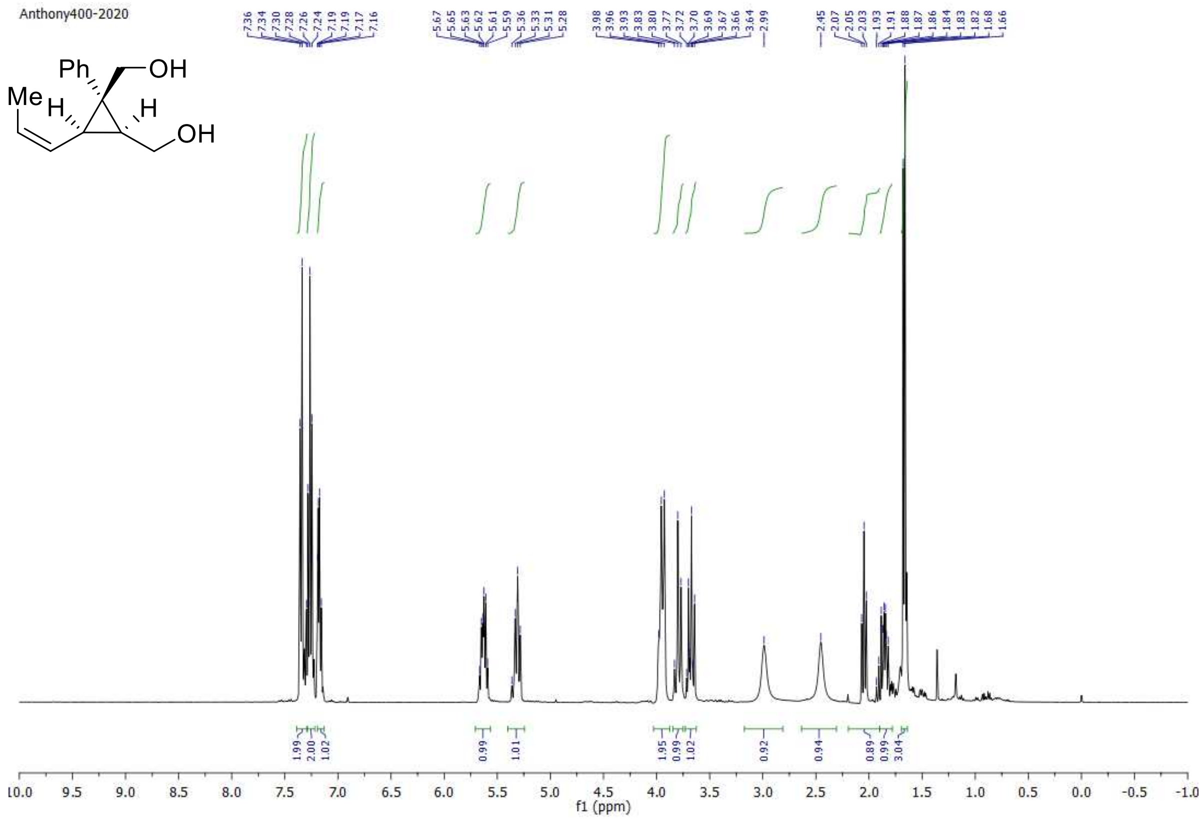

Anthony400-2020
Ph
OH
Me
H
H
OH
7.36 7.34 7.30 7.28 7.26 7.24 7.19 7.19 7.17 7.16
5.67 5.65 5.63 5.62 5.61 5.59 5.36 5.33 5.31 5.28
3.98 3.96 3.93 3.83 3.80 3.77 3.72 3.70 3.69 3.67 3.66 3.64 2.99
2.45 2.07 2.05 2.03 1.93 1.91 1.88 1.87 1.86 1.84 1.83 1.82 1.68 1.66
1.99 2.00 1.02
0.99 1.01
1.95 0.99 1.02
0.92 0.94
0.89 0.99 3.04
f1 (ppm)

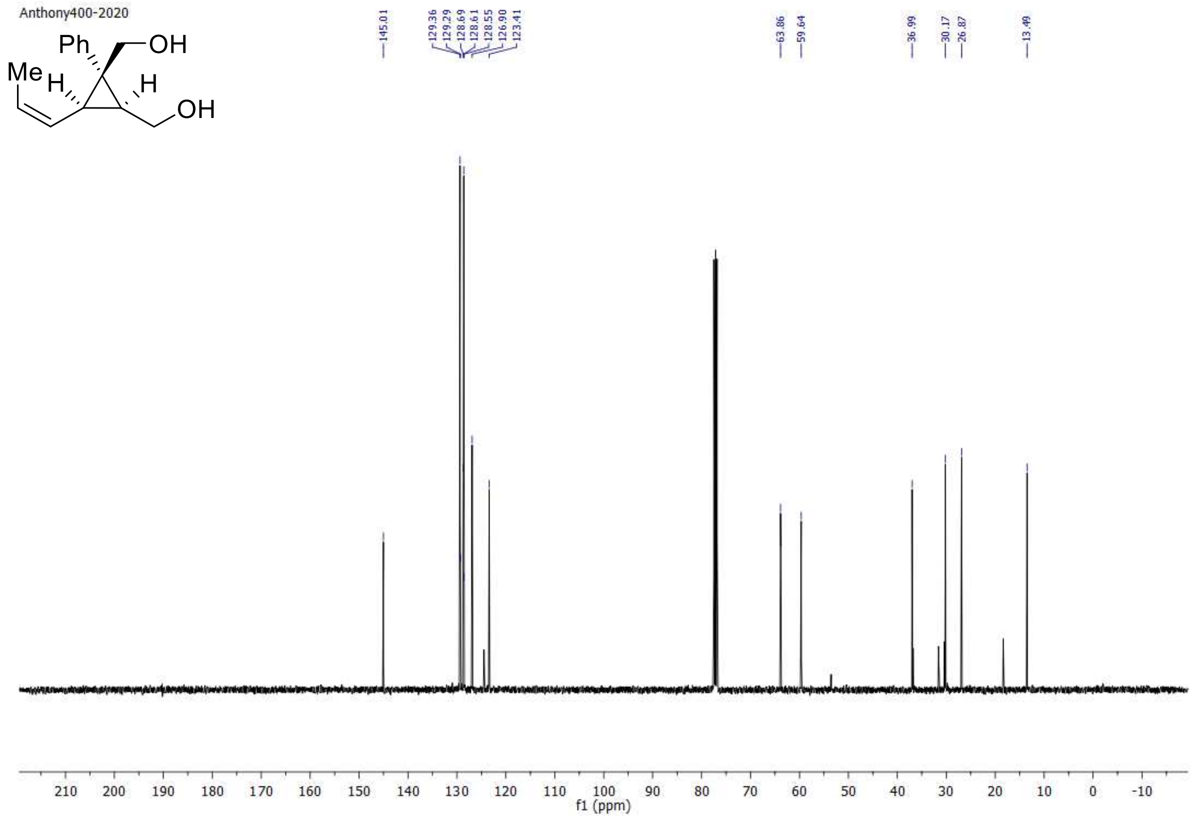

Anthony400-2020
Ph
OH
Me
H
H
OH
145.01
129.36 129.29 128.69 128.61 128.55 126.90 123.41
63.86 59.64
36.99 30.17 26.87
13.49
f1 (ppm)

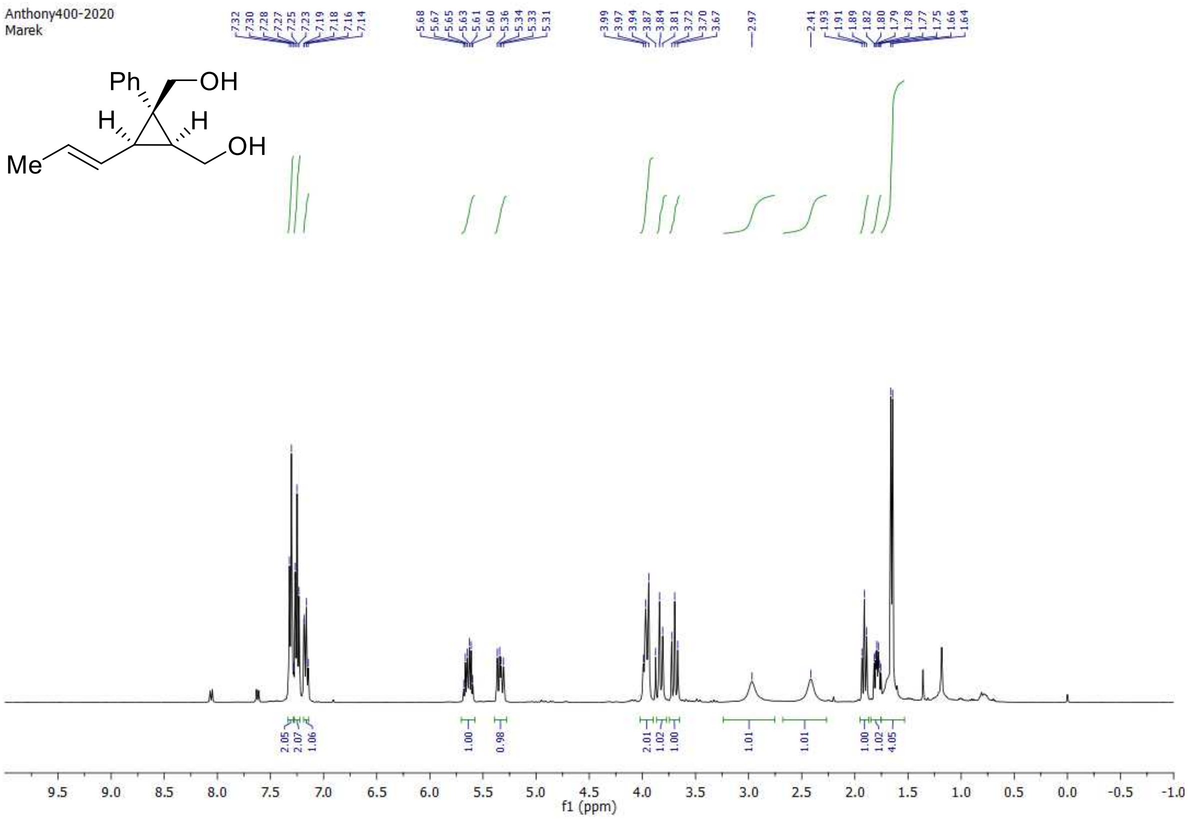
Anthony400-2020
Marek
Ph
OH
H
H
OH
Me
f1 (ppm)

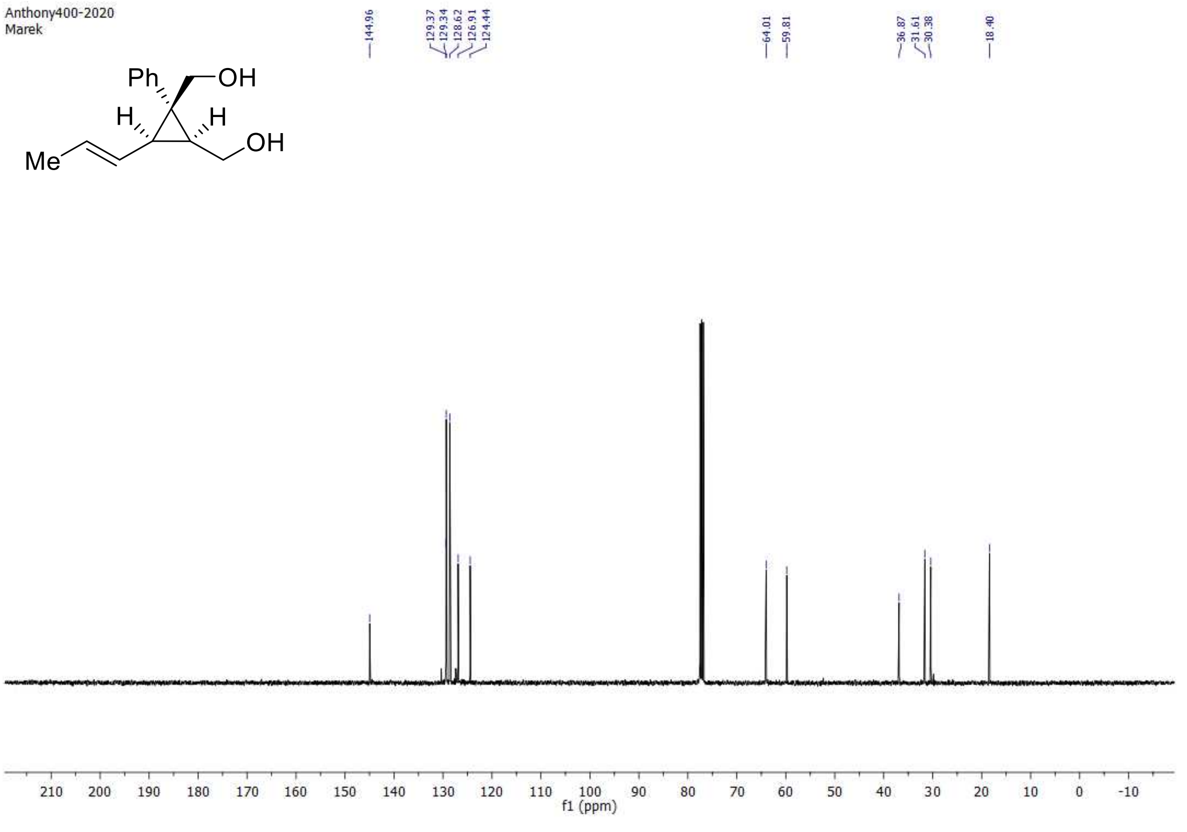
Anthony400-2020
Marek
Ph
OH
H
H
OH
Me
144.96
129.37
129.34
128.62
126.91
124.44
64.01
59.81
36.87
31.61
30.38
18.40
f1 (ppm)

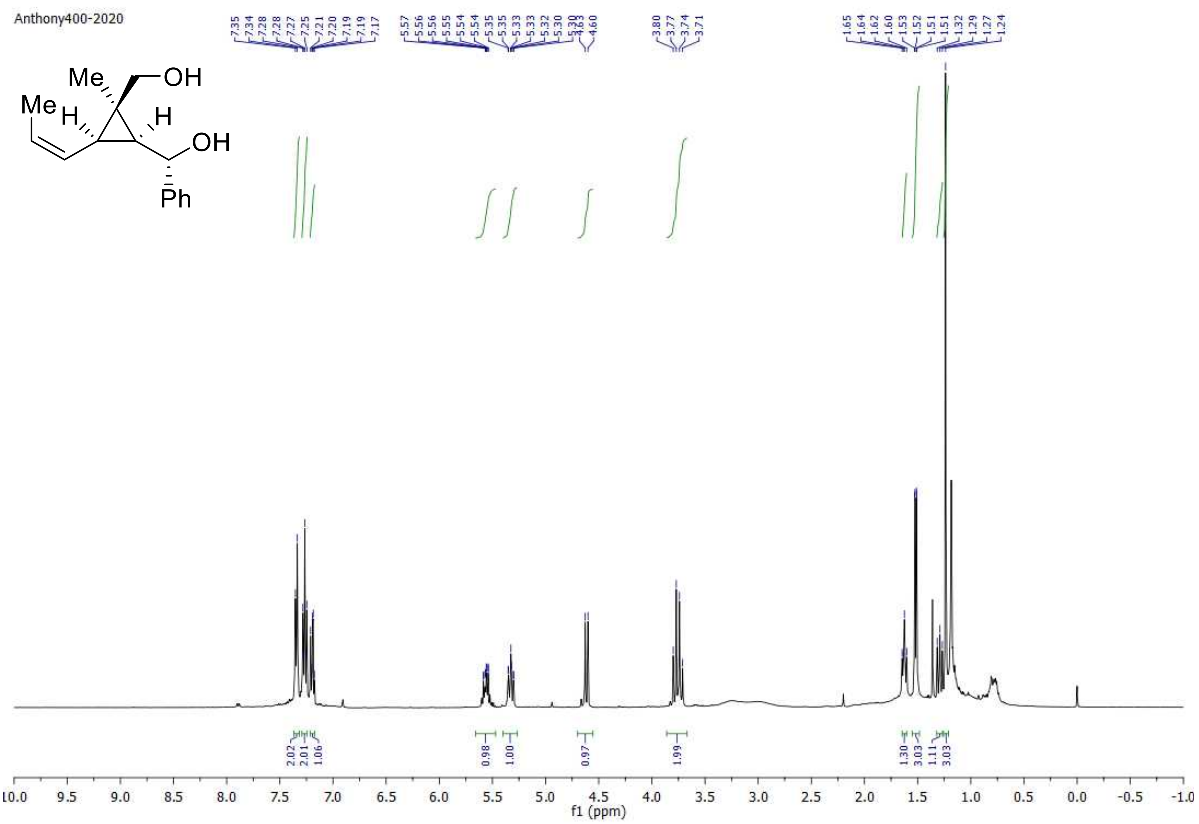
Anthony400-2020
7.35
7.34
7.28
7.27
7.25
7.21
7.20
7.19
7.19
7.17
5.57
5.56
5.56
5.55
5.54
5.54
5.35
5.35
5.33
5.33
5.32
5.30
4.60
3.80
3.77
3.74
3.71
1.65
1.64
1.62
1.60
1.53
1.52
1.51
1.51
1.32
1.29
1.27
1.24
2.02
2.01
1.06
0.98
1.00
0.97
1.99
1.30
3.03
1.11
3.03
f1 (ppm)

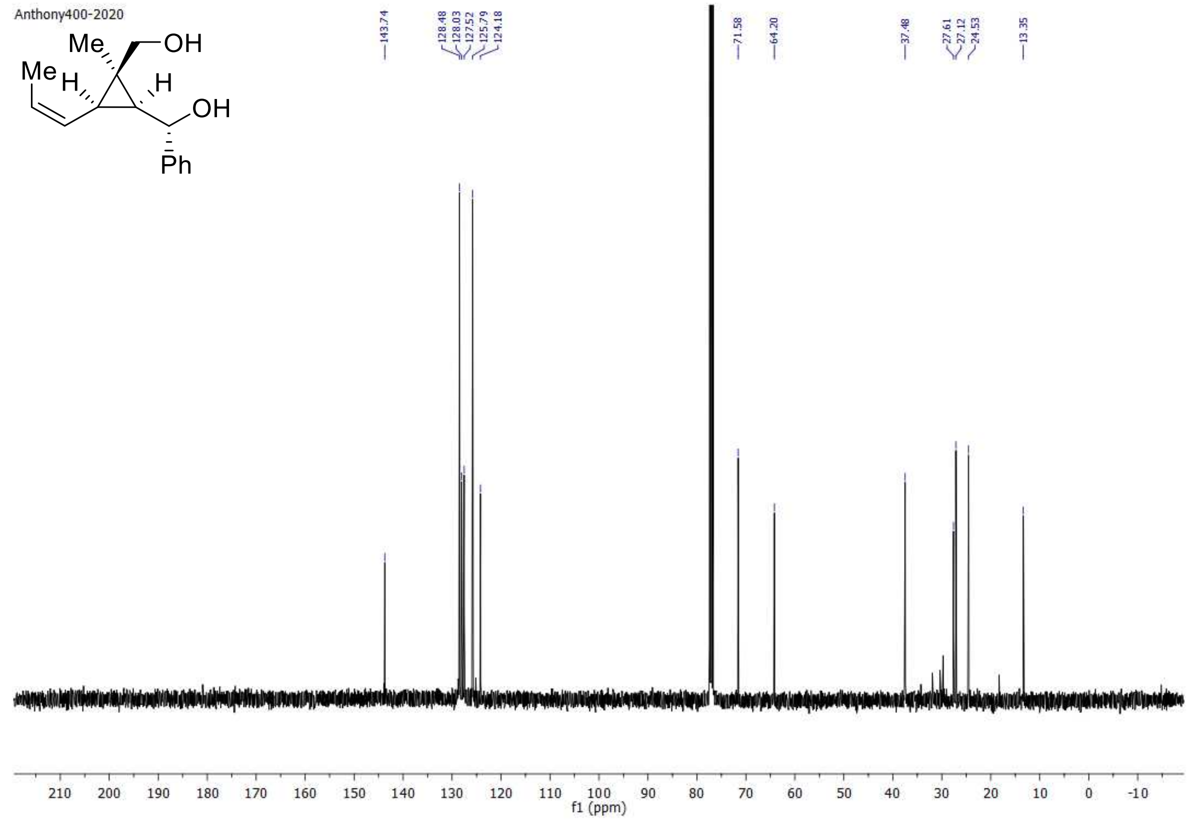
Anthony400-2020
143.74
128.48
128.03
127.52
125.79
124.18
71.58
64.20
37.48
27.61
27.12
24.53
13.35
f1 (ppm)

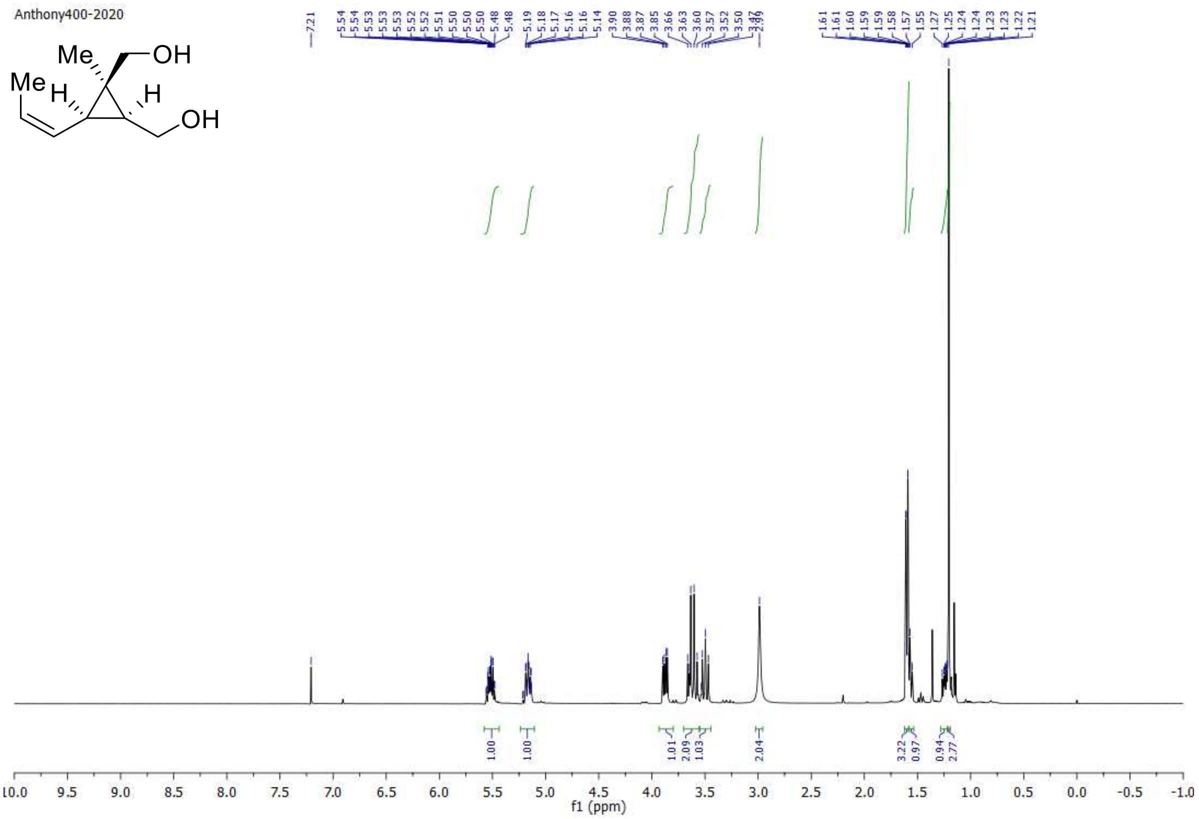
Anthony400-2020
Me
OH
Me
H
H
OH
7.21
5.54 5.54 5.53 5.53 5.53 5.52 5.52 5.51 5.50 5.50 5.48 5.48
5.19 5.18 5.17 5.16 5.16 5.14
3.90 3.88 3.87 3.85 3.66 3.63 3.60 3.57 3.52 3.50 3.47 2.99
1.61 1.61 1.60 1.59 1.59 1.58 1.57 1.55 1.27 1.25 1.24 1.24 1.23 1.23 1.22 1.21
1.00
1.00
1.01
2.09
1.03
2.04
3.22
0.97
0.94
2.77
10.0 9.5 9.0 8.5 8.0 7.5 7.0 6.5 6.0 5.5 5.0 4.5 4.0 3.5 3.0 2.5 2.0 1.5 1.0 0.5 0.0 -0.5 -1.0
f1 (ppm)

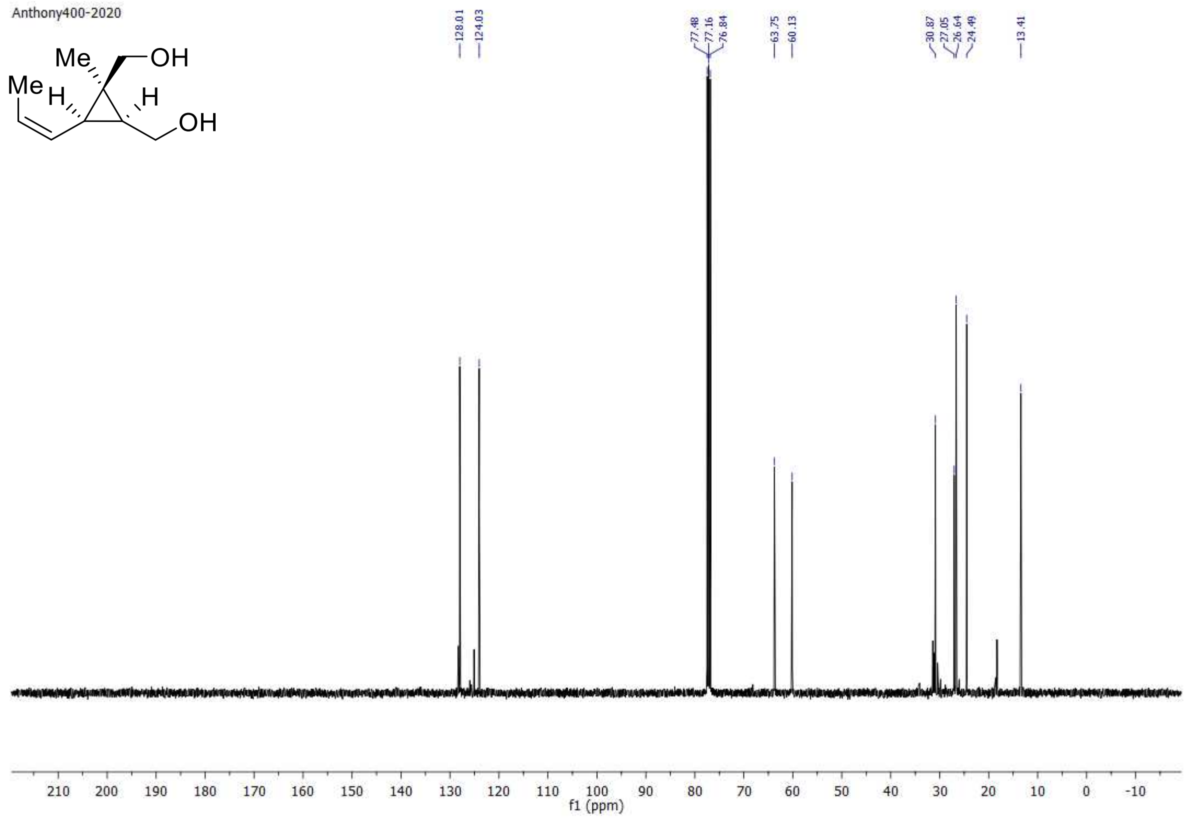
Anthony400-2020
Me
OH
Me
H
H
OH
128.01
124.03
77.48
77.16
76.84
63.75
60.13
30.87
27.05
26.64
24.49
13.41
210 200 190 180 170 160 150 140 130 120 110 100 90 80 70 60 50 40 30 20 10 0 -10
f1 (ppm)

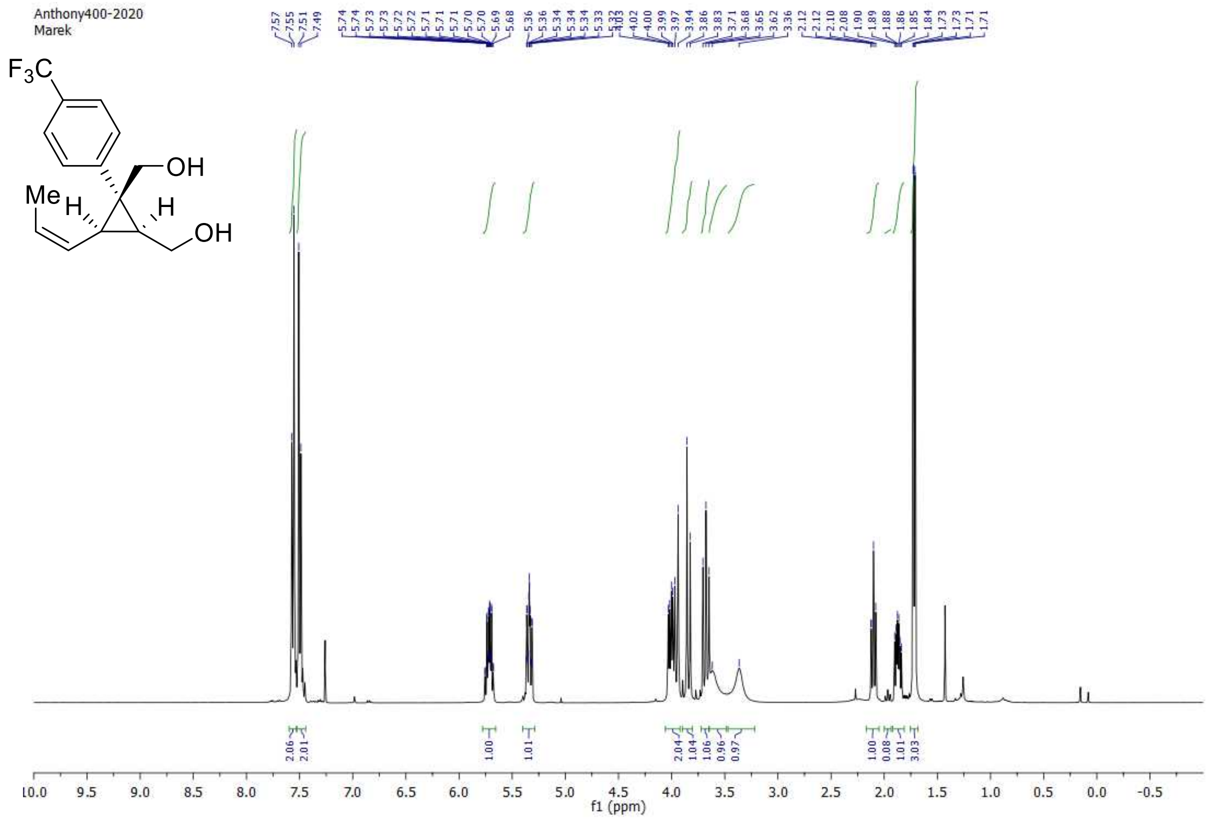
Anthony400-2020
Marek
F3C
OH
Me
H
H
OH
f1 (ppm)

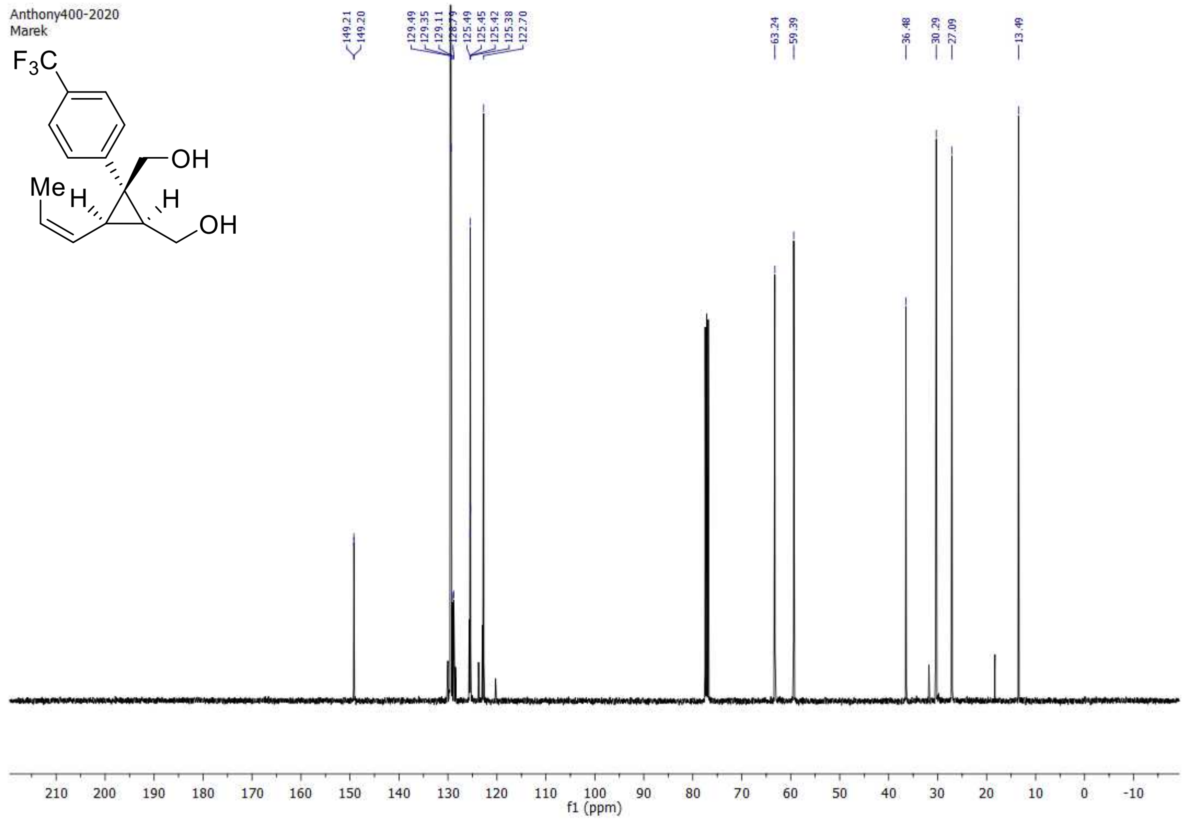
Anthony400-2020
Marek
F3C
OH
Me
H
H
OH
f1 (ppm)

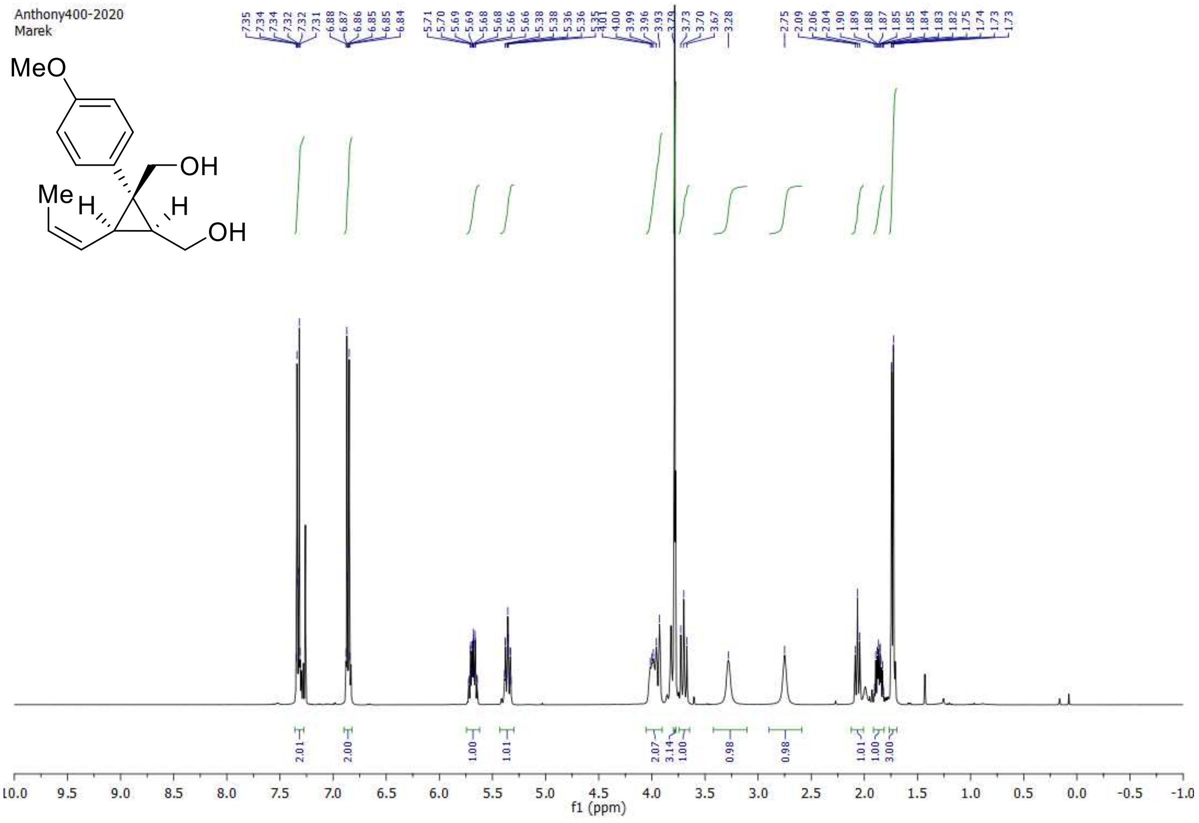
Anthony400-2020
Marek
MeO
OH
Me
H
H
OH
7.35 7.34 7.34 7.32 7.32 7.31 6.88 6.87 6.86 6.85 6.85 6.84 5.71 5.70 5.69 5.69 5.68 5.68 5.66 5.66 5.38 5.38 5.36 5.36 5.35 4.01 4.00 3.99 3.96 3.93 3.79 3.73 3.70 3.67 3.28 2.75 2.09 2.06 2.04 1.90 1.89 1.88 1.87 1.85 1.85 1.84 1.83 1.82 1.75 1.74 1.73 1.73
2.01 2.00 1.00 1.01 2.07 3.14 1.00 0.98 0.98 1.01 1.00 3.00
10.0 9.5 9.0 8.5 8.0 7.5 7.0 6.5 6.0 5.5 5.0 4.5 4.0 3.5 3.0 2.5 2.0 1.5 1.0 0.5 0.0 -0.5 -1.0
f1 (ppm)

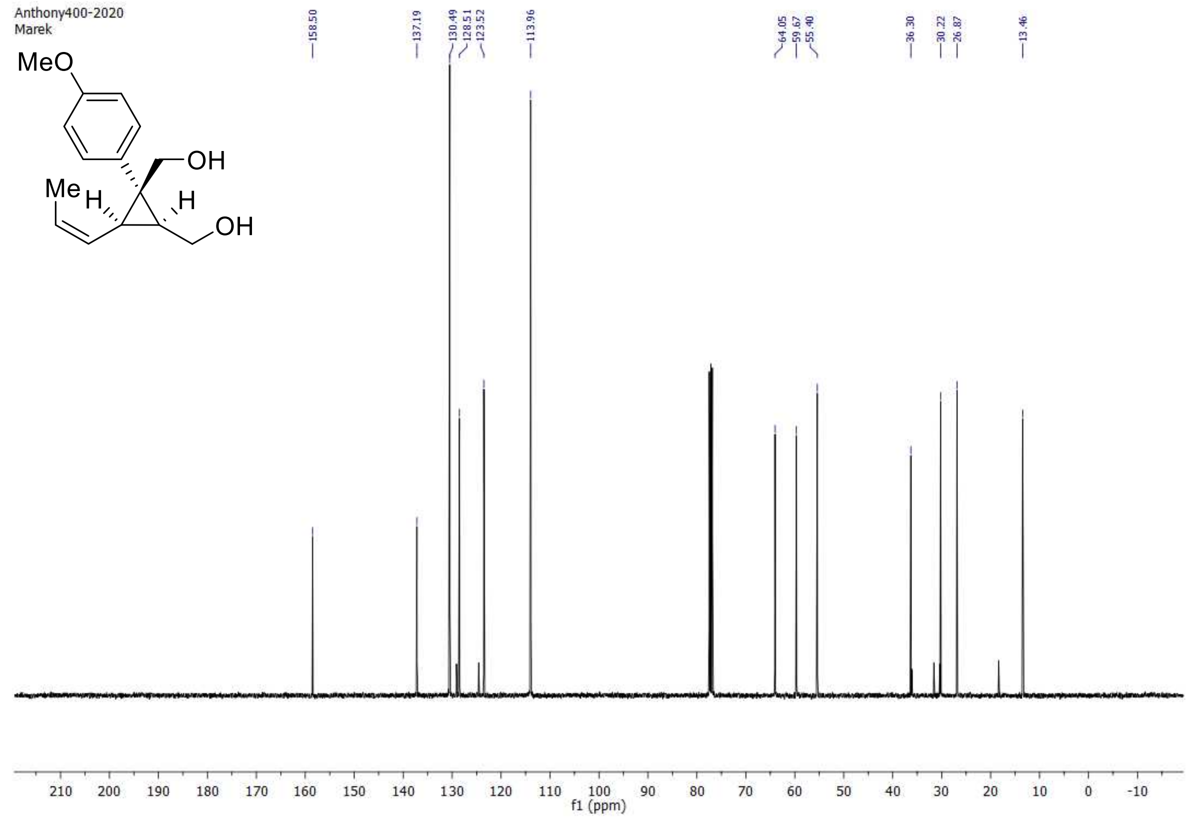
Anthony400-2020
Marek
MeO
OH
Me
H
H
OH
158.50 137.19 130.49 128.51 123.52 113.96 64.05 59.67 55.40 36.30 30.22 26.87 13.46
210 200 190 180 170 160 150 140 130 120 110 100 90 80 70 60 50 40 30 20 10 0 -10
f1 (ppm)

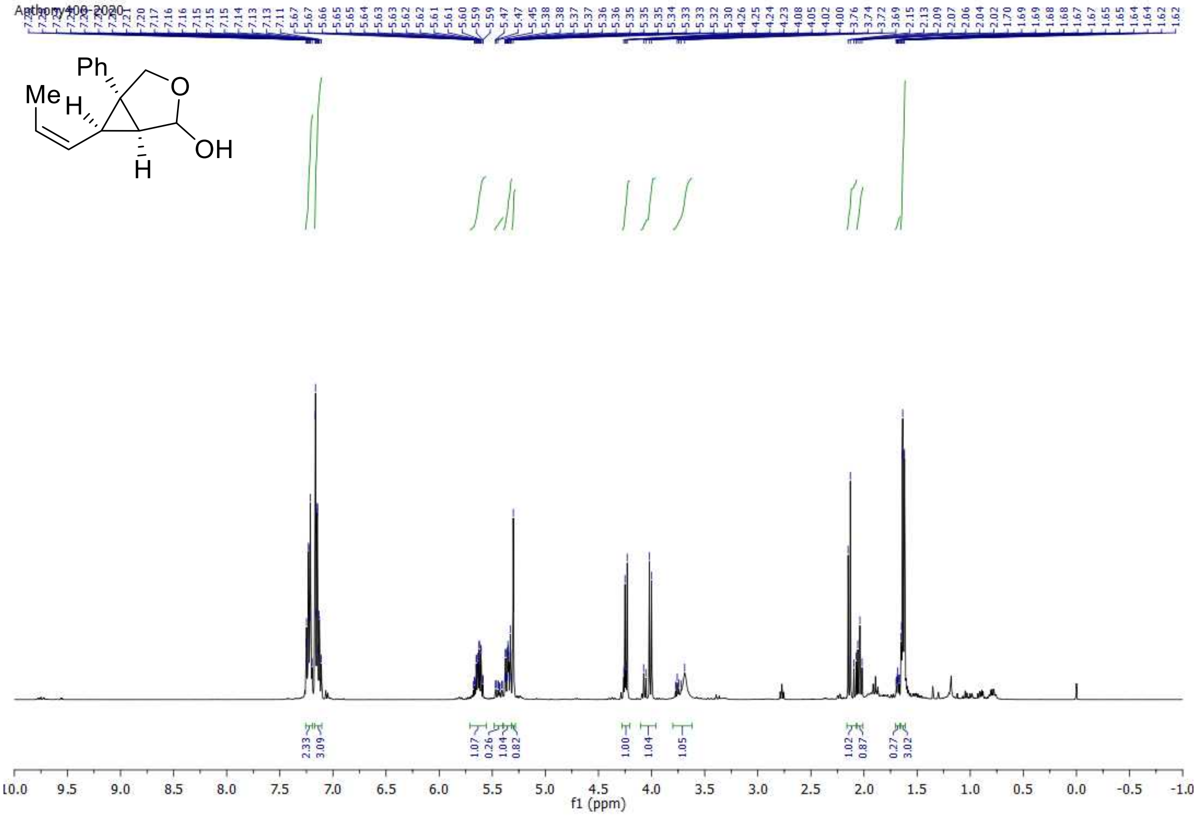

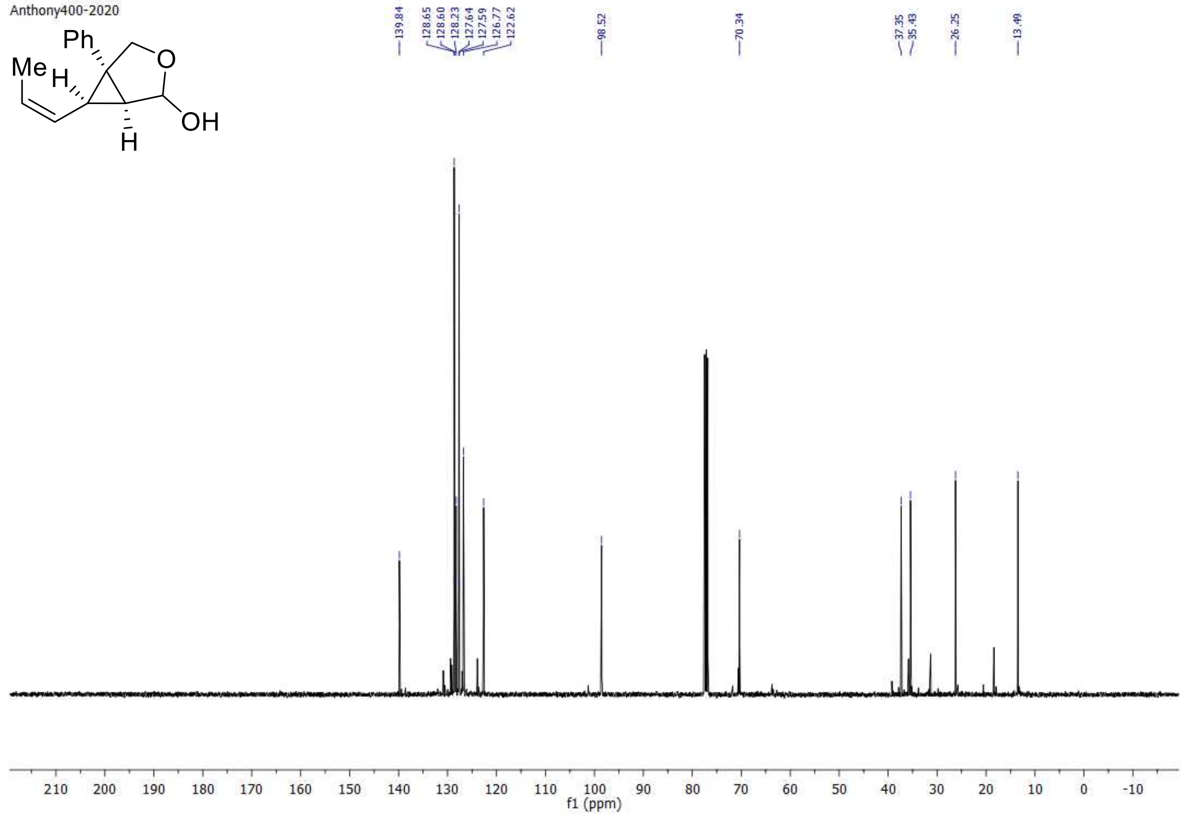

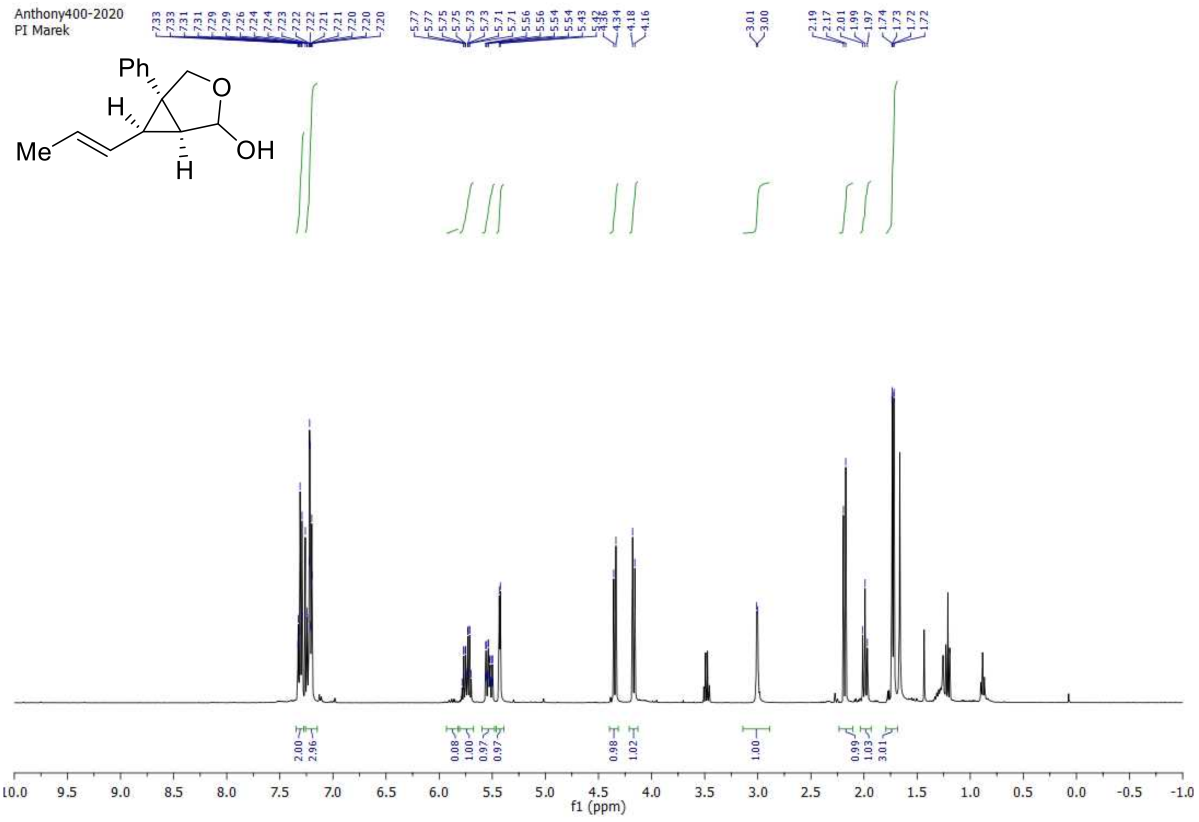
Anthony400-2020
PI Marek
Ph
O
H
Me
OH
H
f1 (ppm)

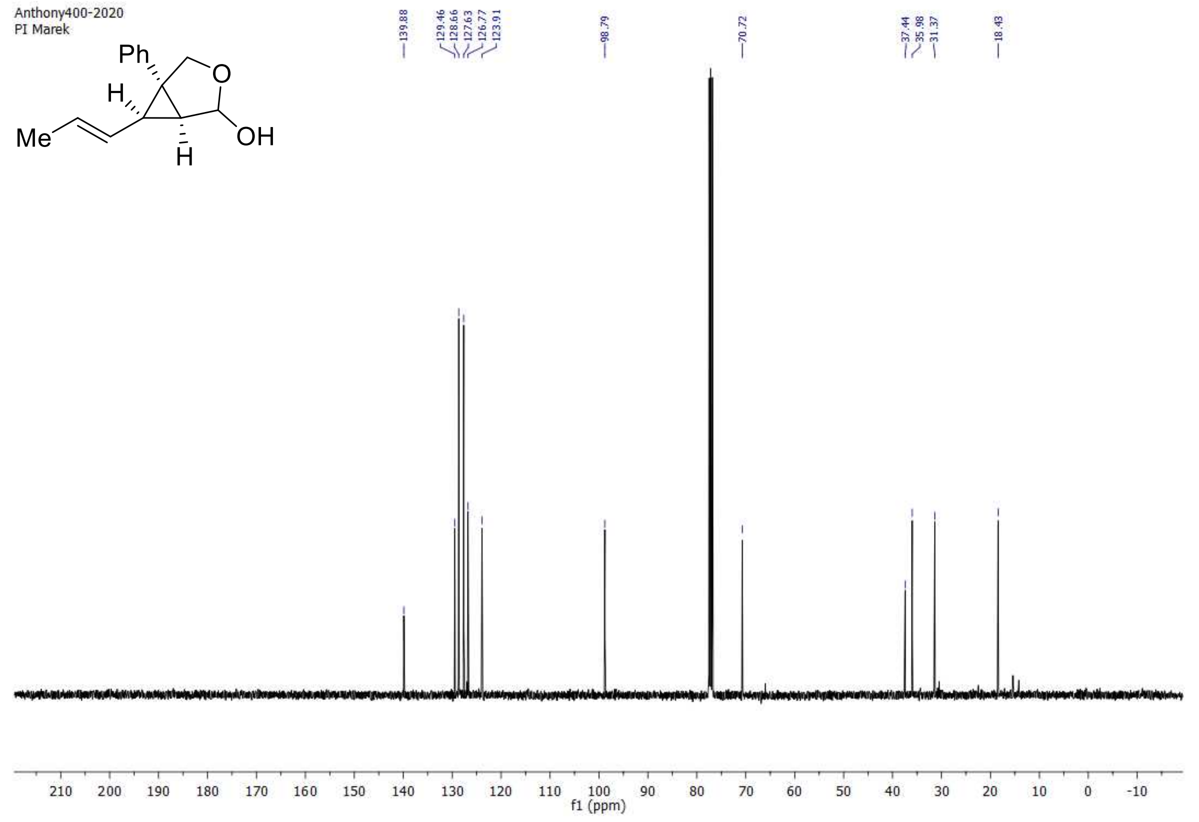
Anthony400-2020
PI Marek
Ph
O
H
Me
OH
H
139.88
129.46
128.66
127.63
126.77
123.91
98.79
70.72
37.44
35.98
31.37
18.43
f1 (ppm)

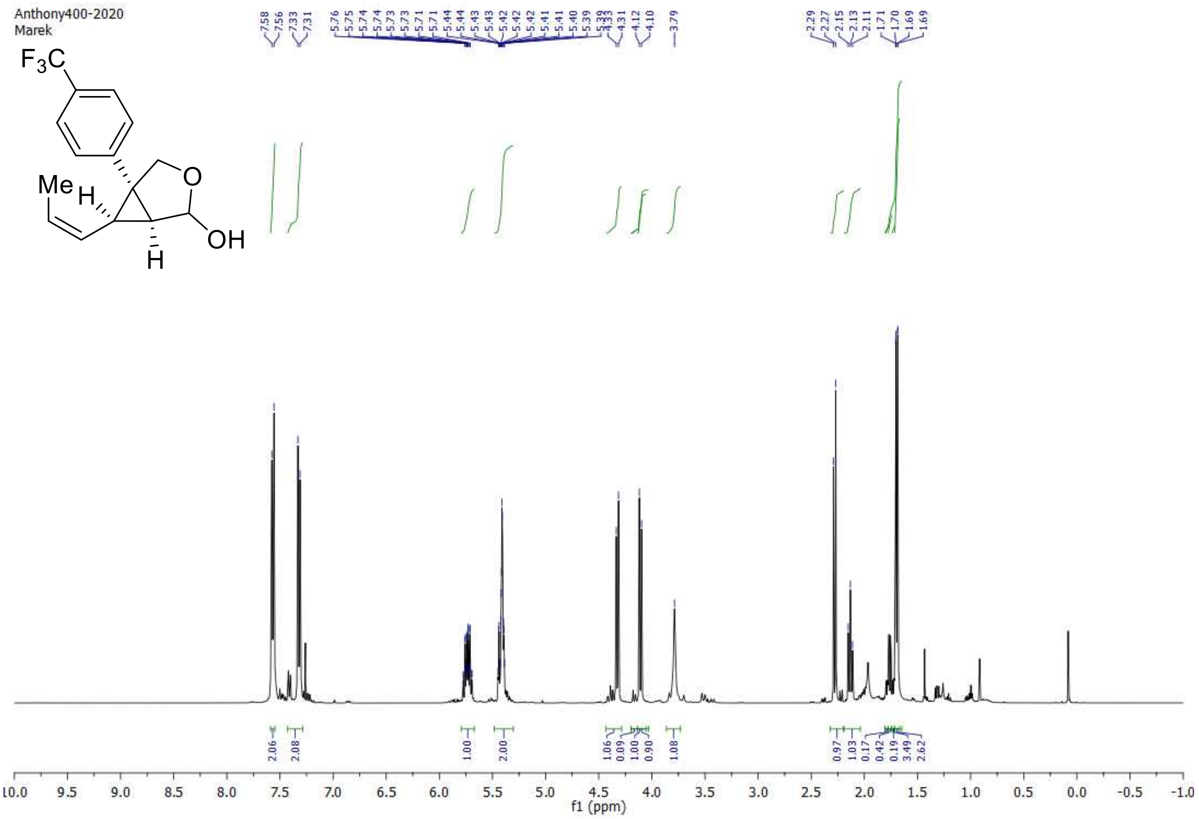
Anthony400-2020
Marek
F3C
Me
H
O
OH
H
7.58
7.56
7.33
7.31
5.76
5.75
5.74
5.74
5.73
5.73
5.71
5.71
5.44
5.44
5.43
5.43
5.42
5.42
5.42
5.41
5.41
5.40
5.39
4.31
4.12
4.10
3.79
2.29
2.27
2.15
2.13
2.11
1.71
1.70
1.69
1.69
2.06
2.08
1.00
2.00
1.06
0.09
1.00
0.90
1.08
0.97
1.03
0.17
0.42
0.19
3.49
2.62
f1 (ppm)

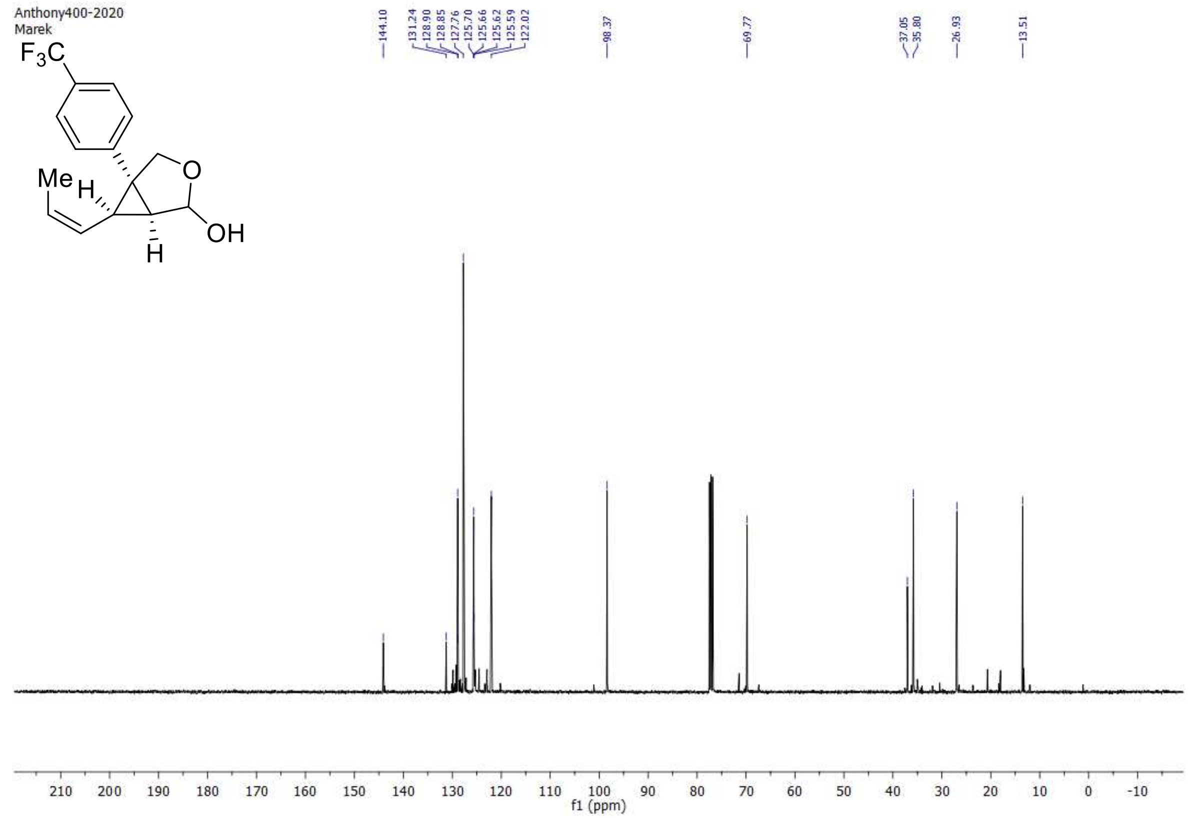
Anthony400-2020
Marek
F3C
Me
H
O
OH
H
144.10
131.24
128.90
128.85
127.76
125.70
125.66
125.62
125.59
122.02
98.37
69.77
37.05
35.80
26.93
13.51
f1 (ppm)

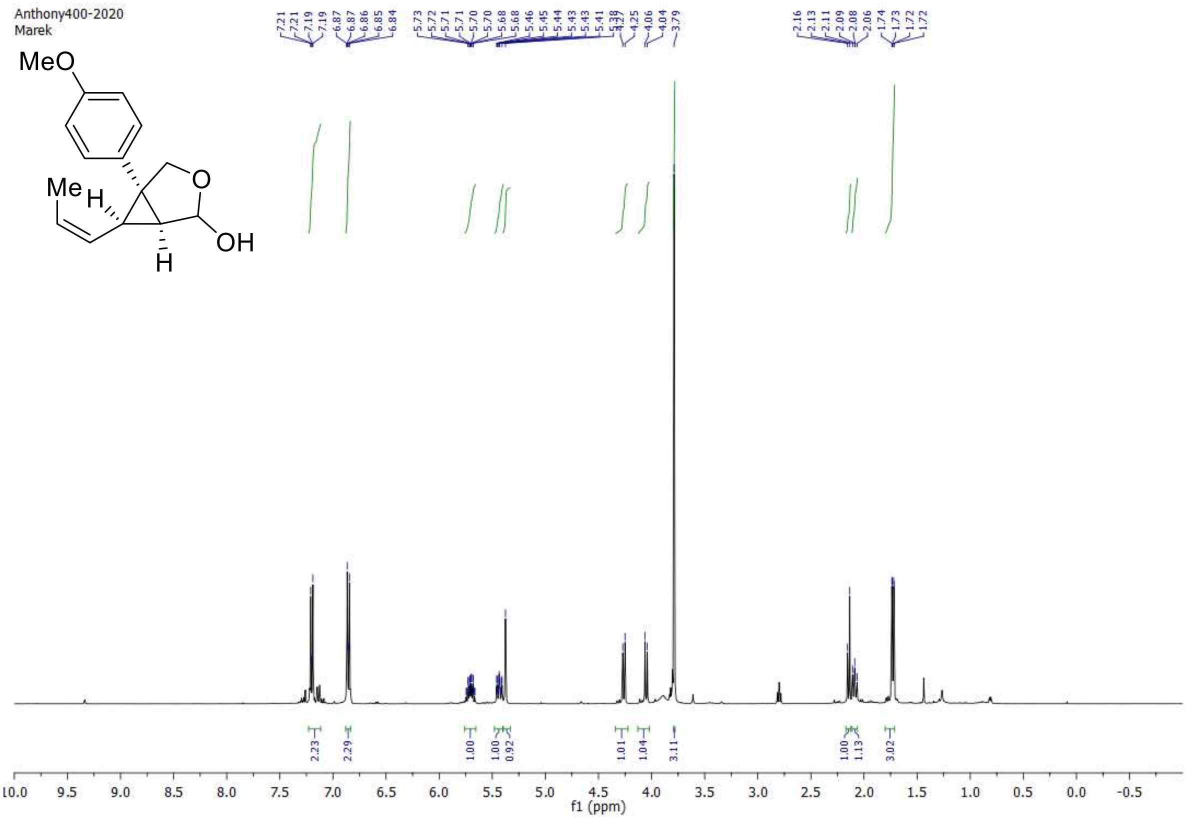

Anthony400-2020
Marek
MeO
Me
H
O
OH
H
7.21
7.21
7.19
7.19
6.87
6.87
6.86
6.85
6.85
6.84
5.73
5.72
5.71
5.70
5.70
5.70
5.68
5.68
5.46
5.45
5.44
5.43
5.43
5.41
4.25
4.06
4.04
3.79
2.16
2.13
2.11
2.09
2.08
2.06
1.74
1.73
1.72
1.72
2.23
2.29
1.00
1.00
0.92
1.01
1.04
3.11
1.00
1.13
3.02
f1 (ppm)

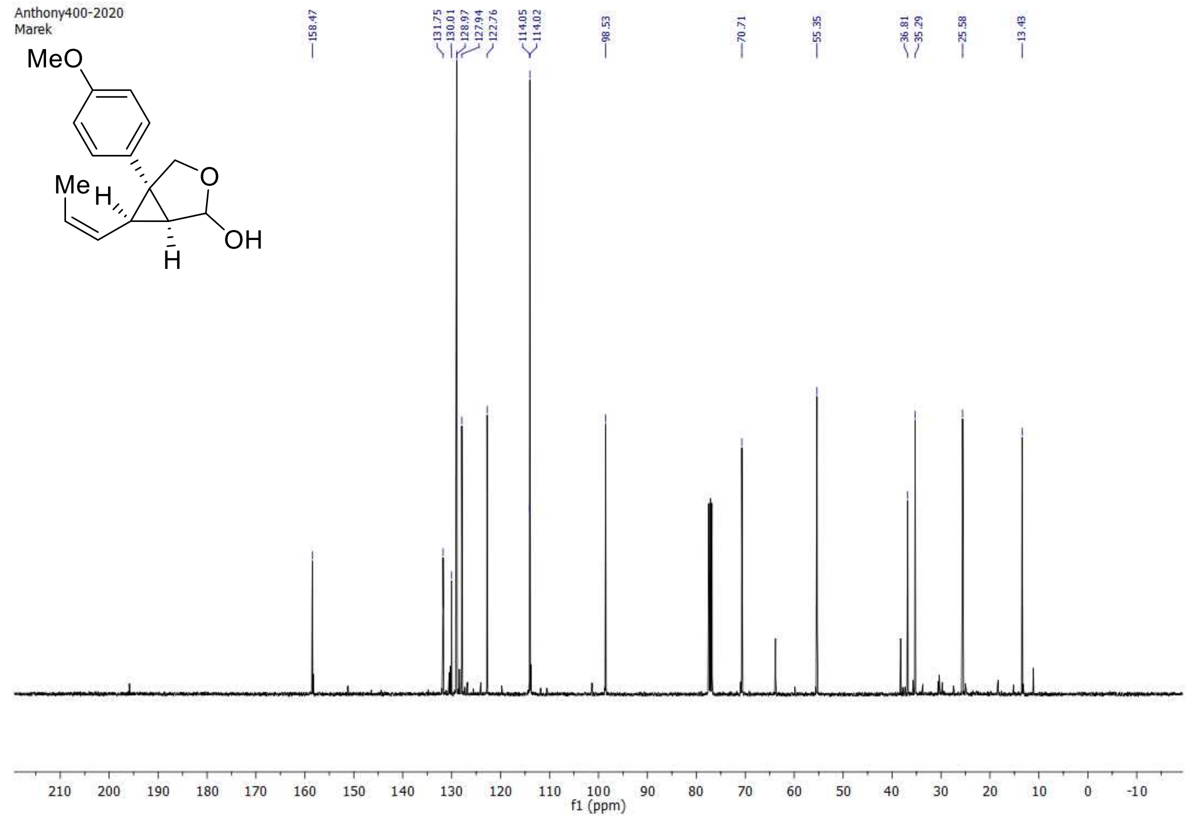

Anthony400-2020
Marek
MeO
Me
H
O
OH
H
158.47
131.75
130.01
128.97
127.94
122.76
114.05
114.02
98.53
70.71
55.35
36.81
35.29
25.58
13.43
f1 (ppm)

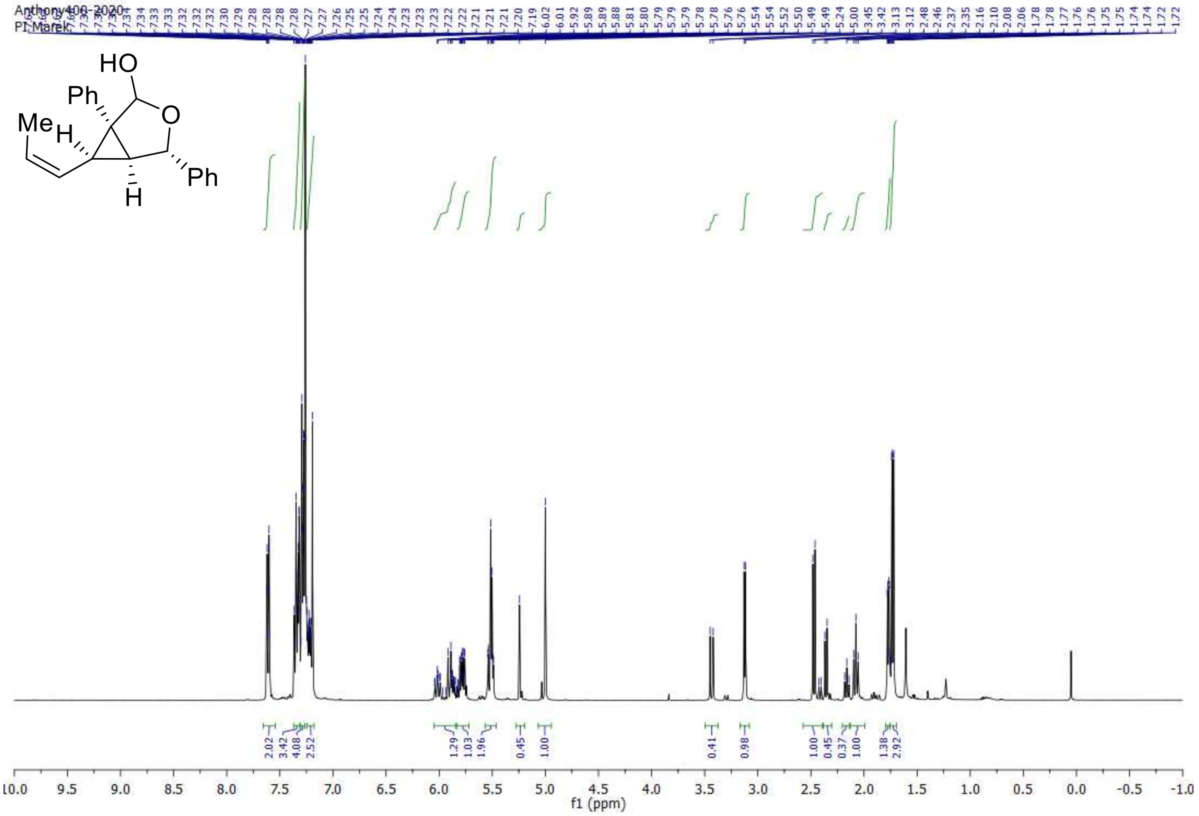
Anthony400-2020
PI Marek
HO
Ph
O
Me
H
Ph
H
f1 (ppm)

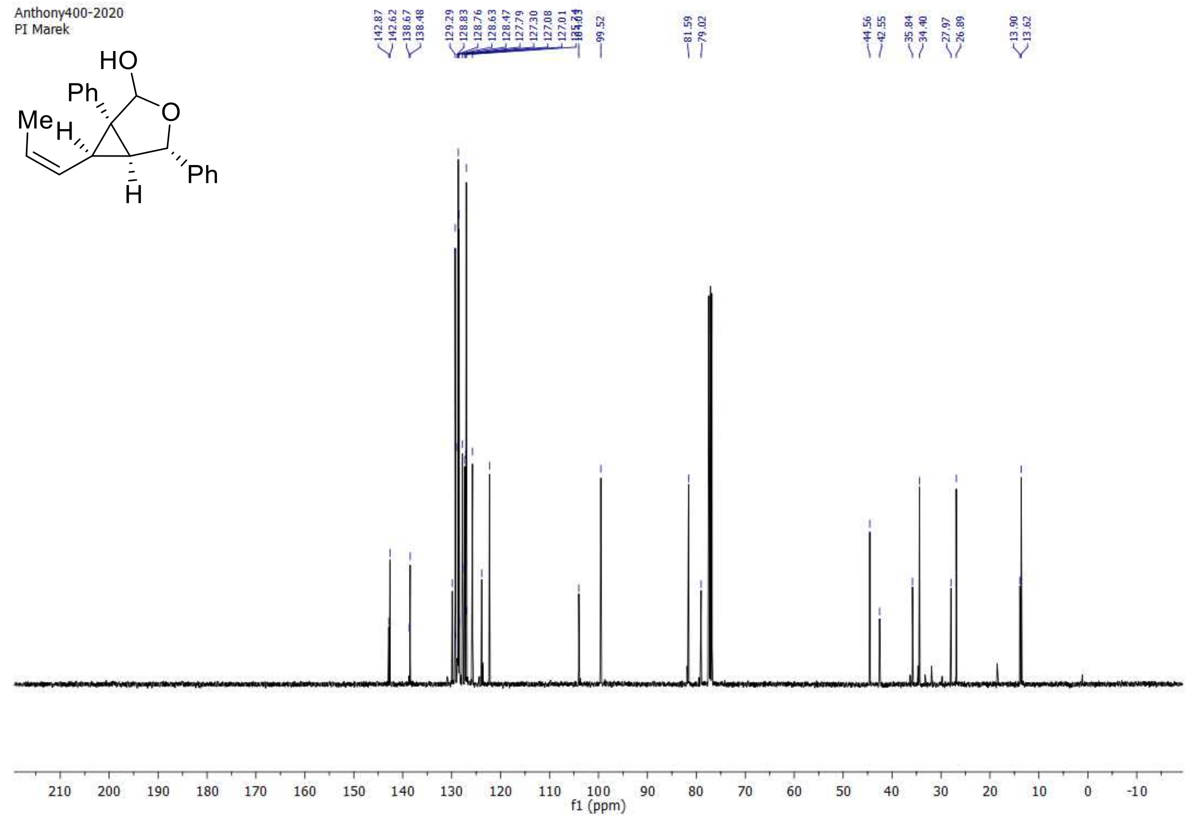
Anthony400-2020
PI Marek
142.87
142.62
138.67
138.48
129.29
128.83
128.76
128.63
128.47
127.79
127.30
127.08
127.01
99.52
81.59
79.02
44.56
42.55
35.84
34.40
27.97
26.89
13.90
13.62
HO
Ph
O
Me
H
Ph
H
f1 (ppm)

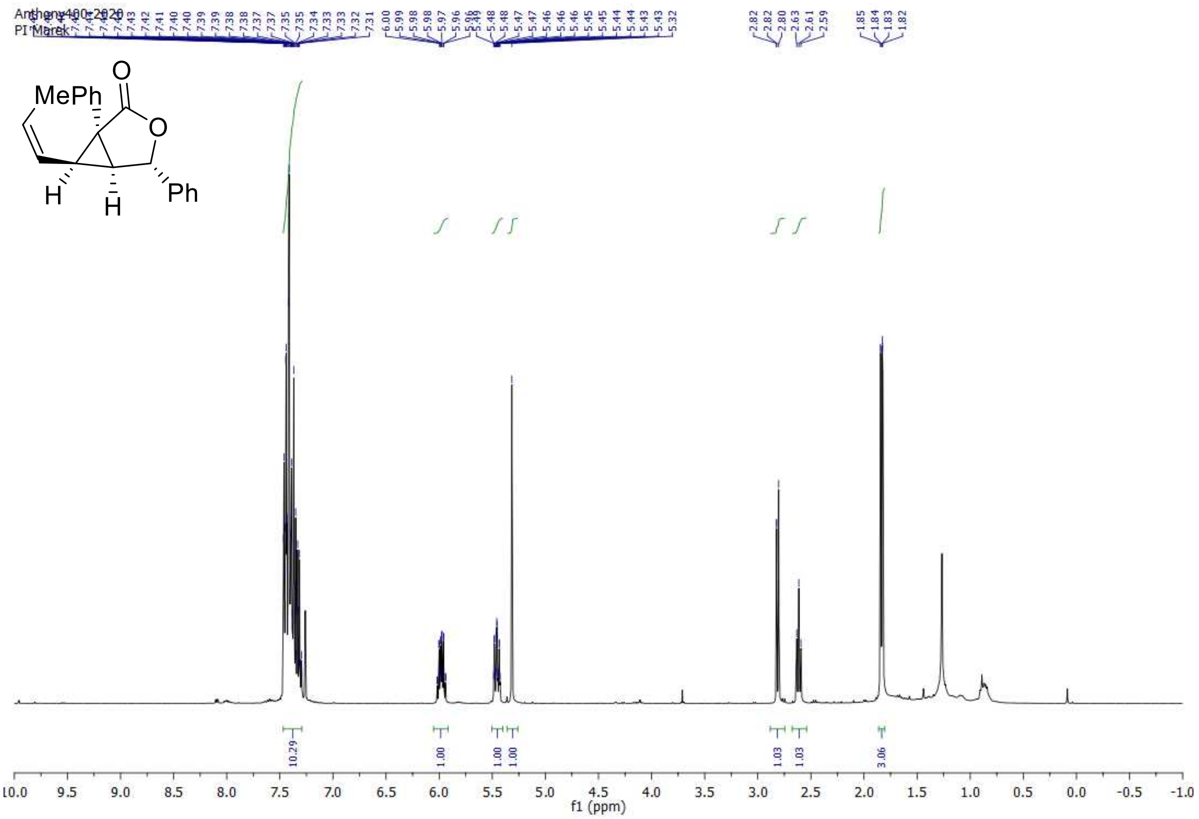
Anthony400-2020
PI Marek
MePh
O
O
H
H
Ph
10.29
1.00
1.00
1.00
1.03
1.03
3.06
f1 (ppm)

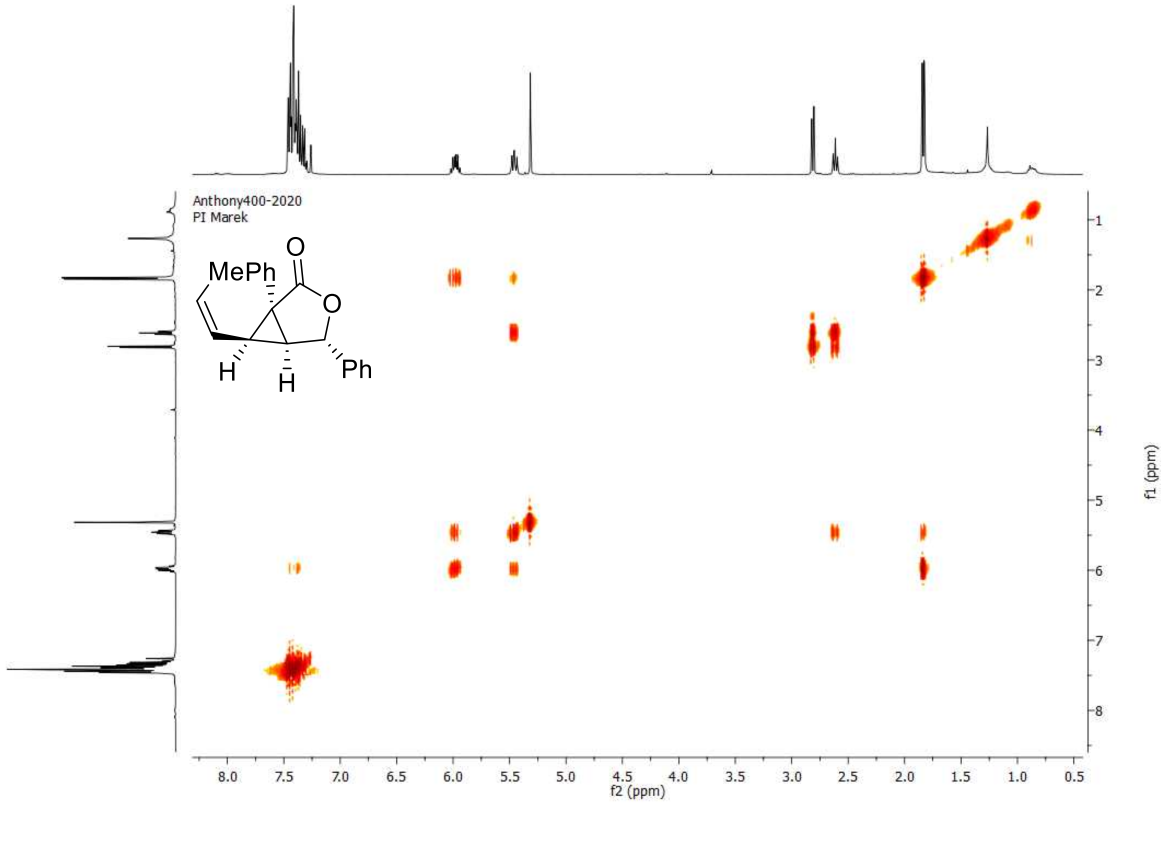
Anthony400-2020
PI Marek
MePh
O
O
H
H
Ph
f2 (ppm)
f1 (ppm)

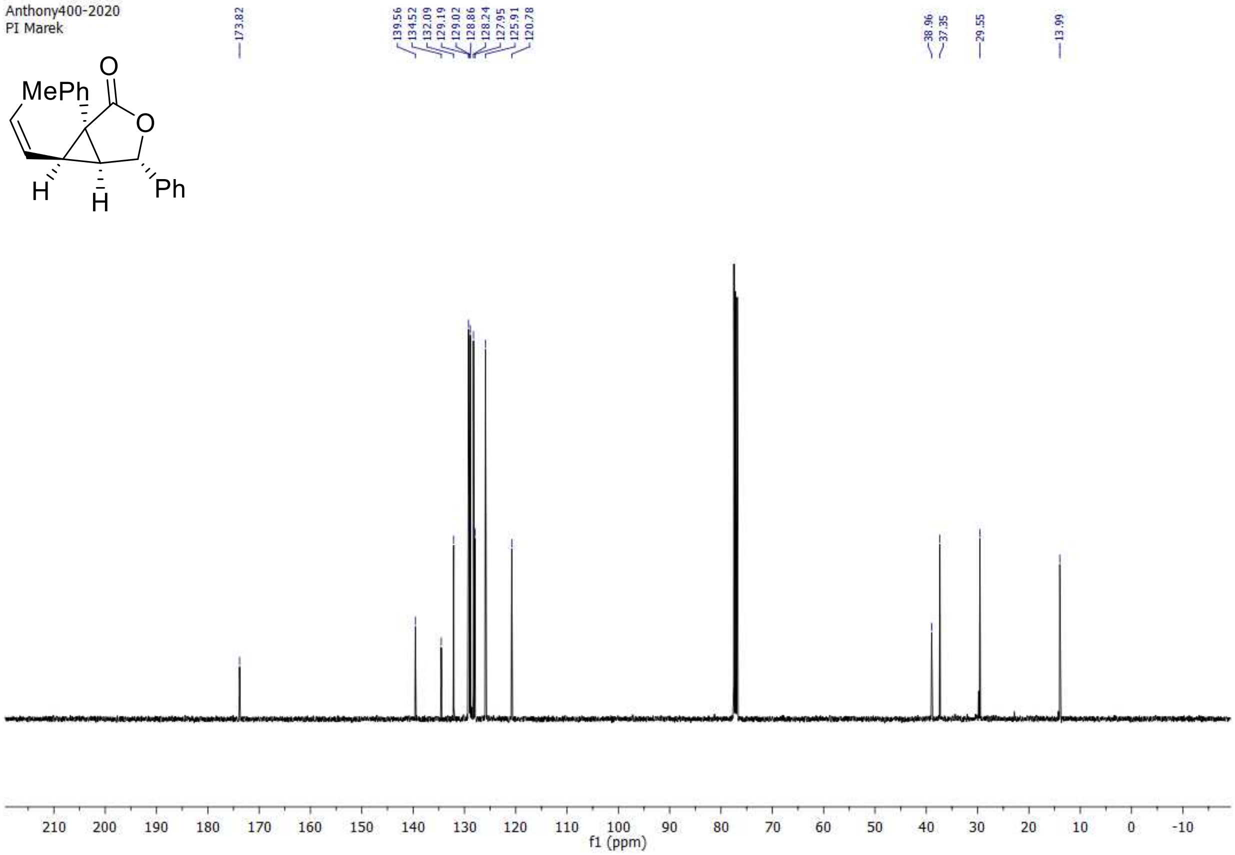
Anthony400-2020
PI Marek
MePh
O
O
H
H
Ph
173.82
139.56
134.52
132.09
129.19
129.02
128.86
128.24
127.95
125.91
120.78
38.96
37.35
29.55
13.99
210 200 190 180 170 160 150 140 130 120 110 100 90 80 70 60 50 40 30 20 10 0 -10
f1 (ppm)

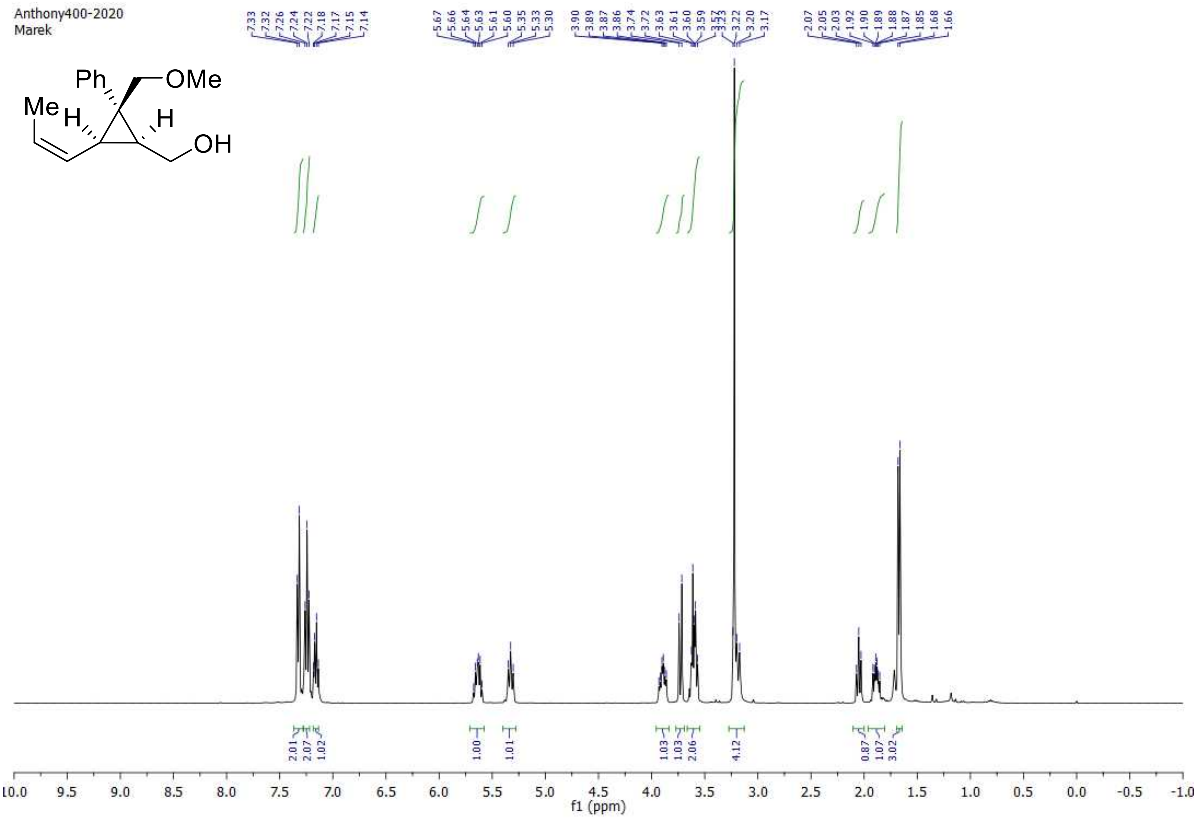
Anthony400-2020
Marek
Ph
OMe
Me
H
H
OH
7.33 7.32 7.26 7.24 7.22 7.18 7.17 7.15 7.14
5.67 5.66 5.64 5.63 5.61 5.60 5.35 5.33 5.30
3.90 3.89 3.87 3.86 3.74 3.72 3.63 3.61 3.60 3.59 3.57 3.23 3.22 3.20 3.17
2.07 2.05 2.03 1.92 1.90 1.89 1.88 1.87 1.85 1.68 1.66
2.01 2.07 1.02 1.00 1.01 1.03 1.03 2.06 4.12 0.87 1.07 3.02
f1 (ppm)

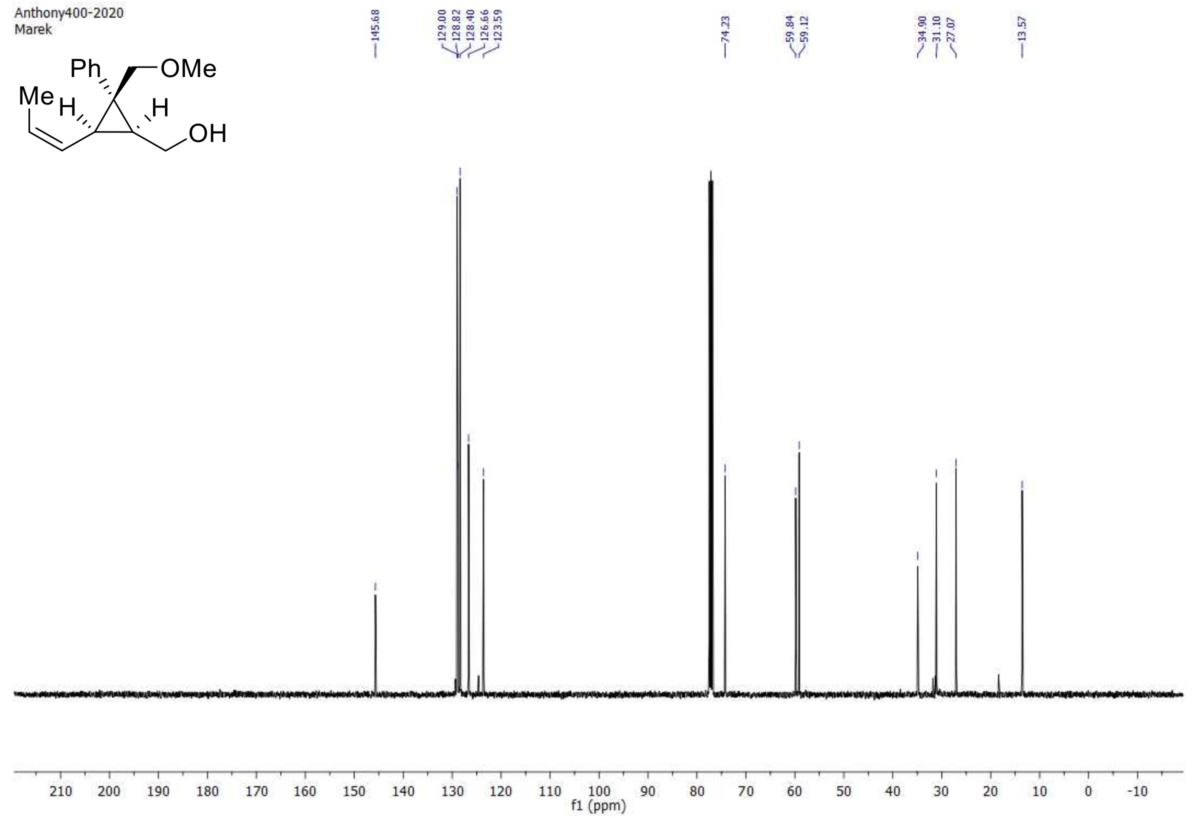
Anthony400-2020
Marek
Ph
OMe
Me
H
H
OH
145.68
129.00 128.82 128.40 126.66 123.59
74.23
59.84 59.12
34.90 31.10 27.07
13.57
f1 (ppm)

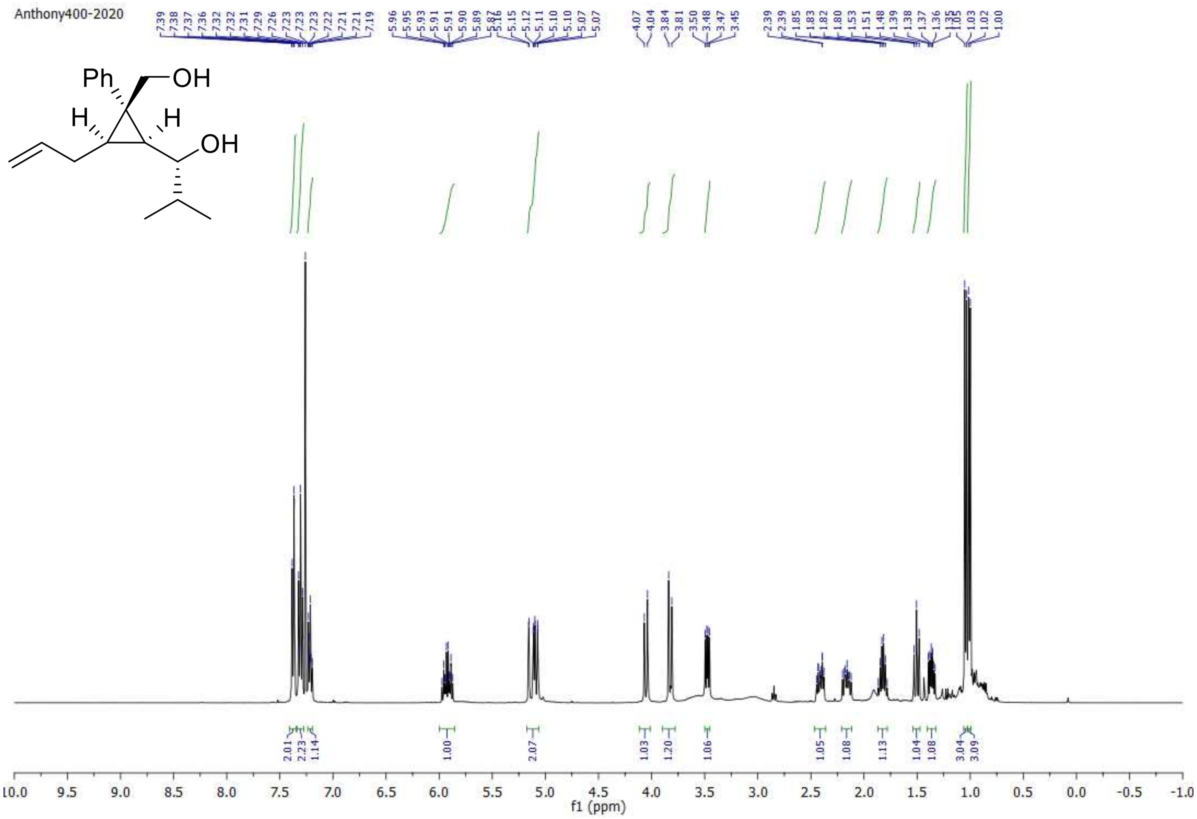
Anthony400-2020
Ph
OH
H
H
OH
f1 (ppm)

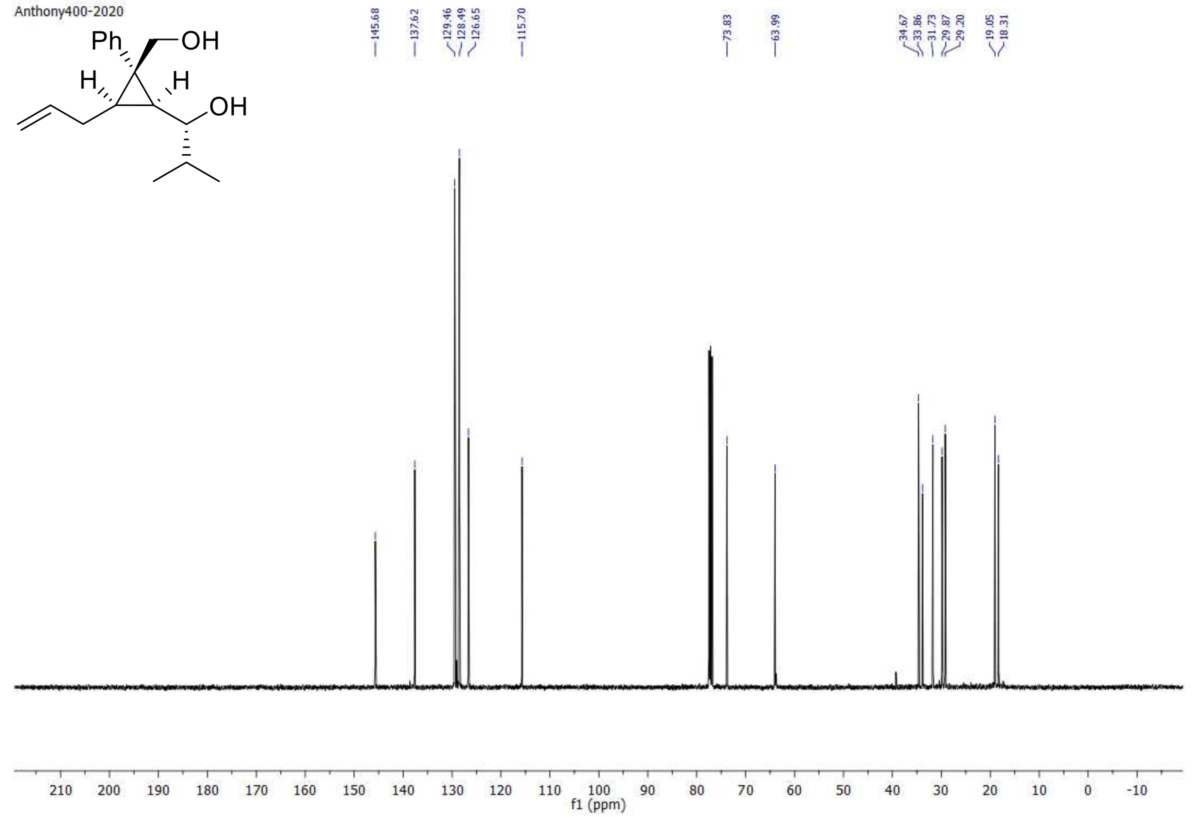
Anthony400-2020
Ph
OH
H
H
OH
145.68
137.62
129.46
128.49
126.65
115.70
73.83
63.99
34.67
33.86
31.73
29.87
29.20
19.05
18.31
f1 (ppm)

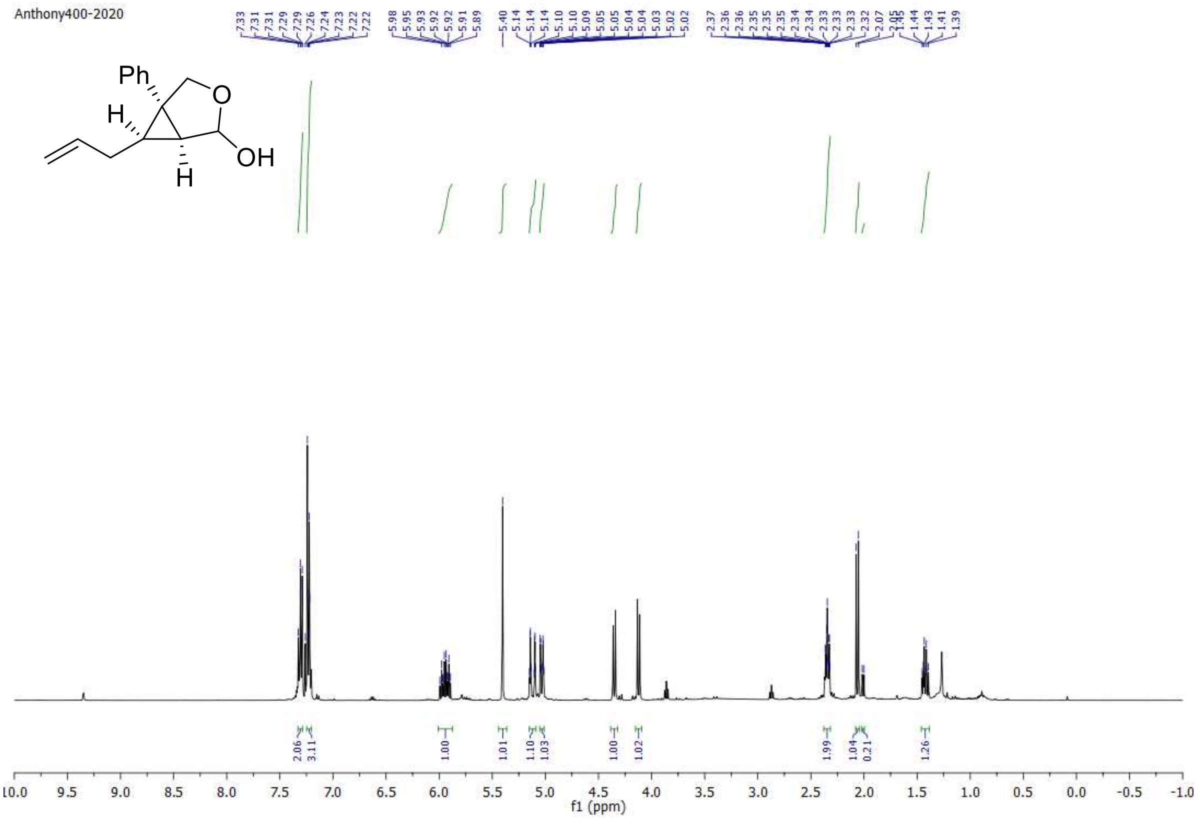
Anthony400-2020
7.33 7.31 7.31 7.29 7.29 7.26 7.24 7.24 7.23 7.23 7.22 7.22
5.98 5.95 5.93 5.92 5.92 5.91 5.89
5.40 5.14 5.14 5.14 5.10 5.10 5.09 5.05 5.05 5.04 5.04 5.03 5.02 5.02
2.37 2.36 2.35 2.35 2.35 2.34 2.34 2.33 2.33 2.33 2.32 2.07 2.05 1.45 1.44 1.43 1.41 1.39
Ph
O
H
OH
H
2.06 3.11 1.00 1.01 1.10 1.03 1.00 1.02 1.99 1.04 0.21 1.26
f1 (ppm)

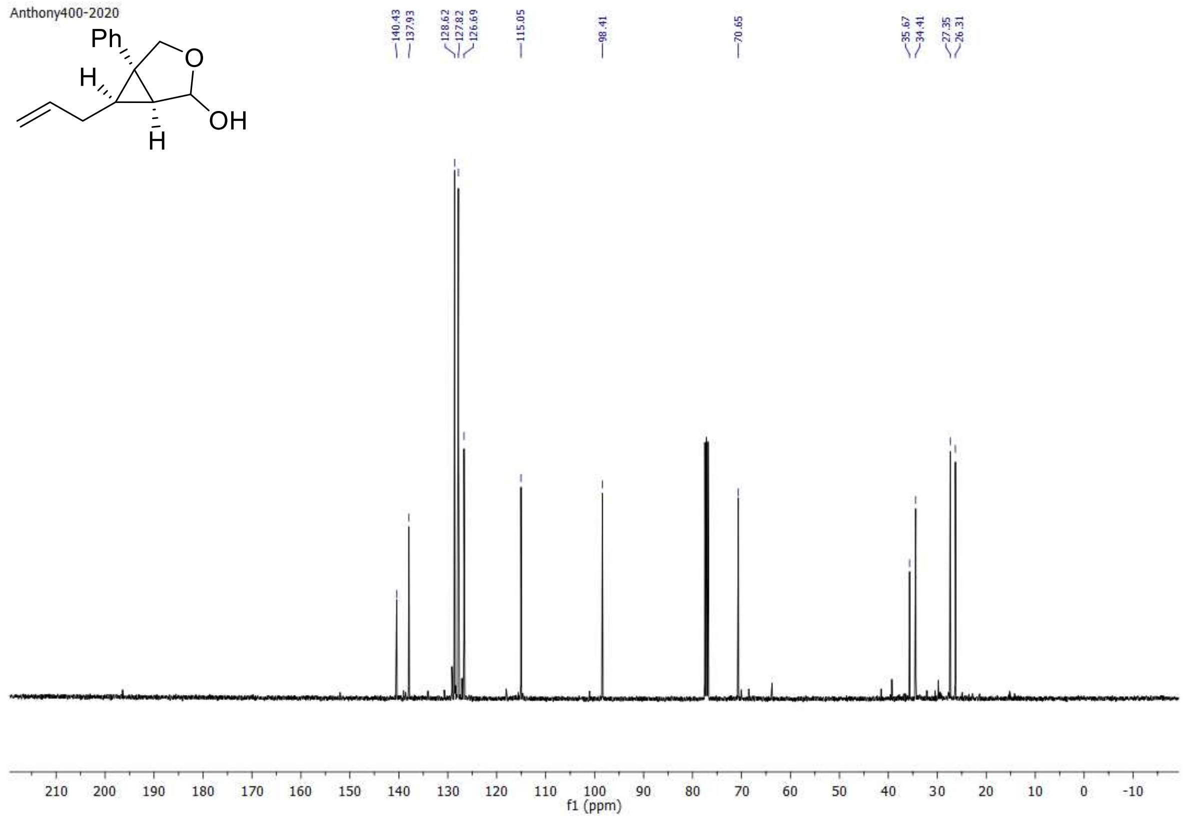
Anthony400-2020
140.43 137.93 128.62 127.82 126.69 115.05 98.41 70.65 35.67 34.41 27.35 26.31
Ph
O
H
OH
H
f1 (ppm)

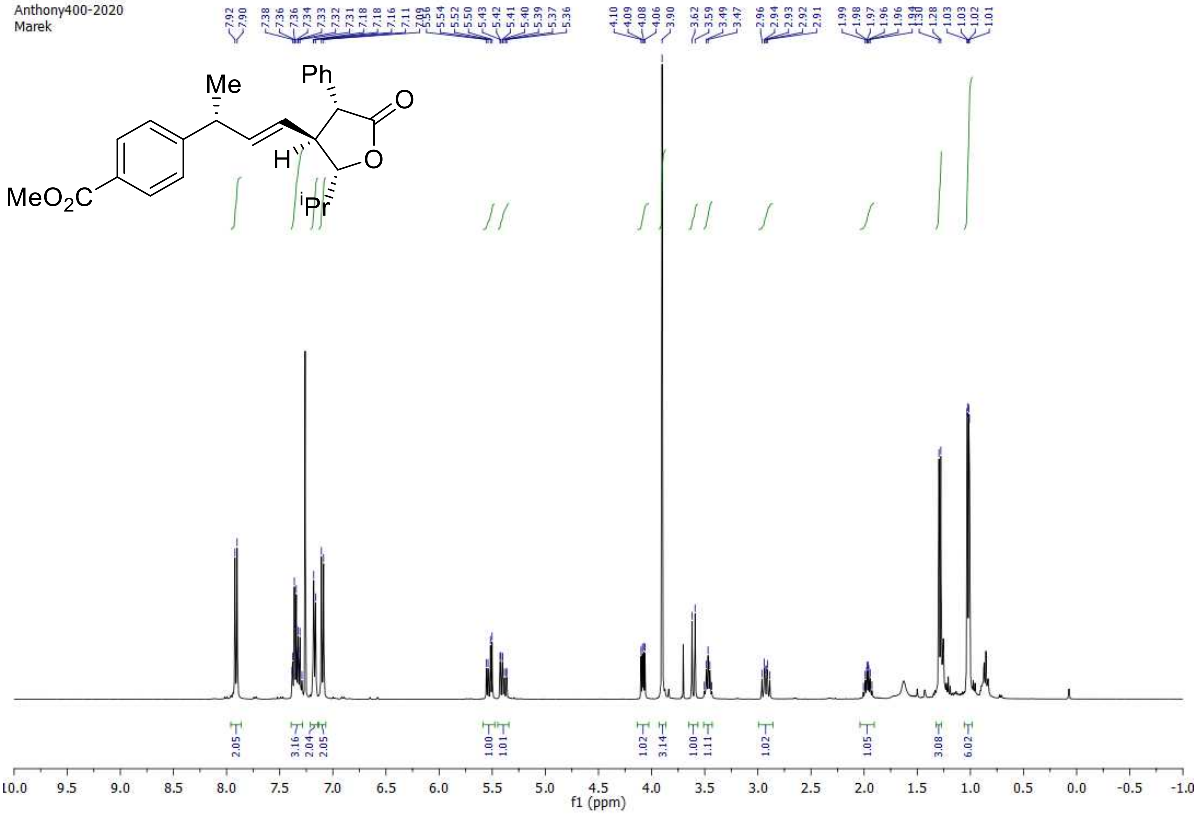
Anthony400-2020
Marek
Me
Ph
O
H
O
MeO2C
iPr
f1 (ppm)

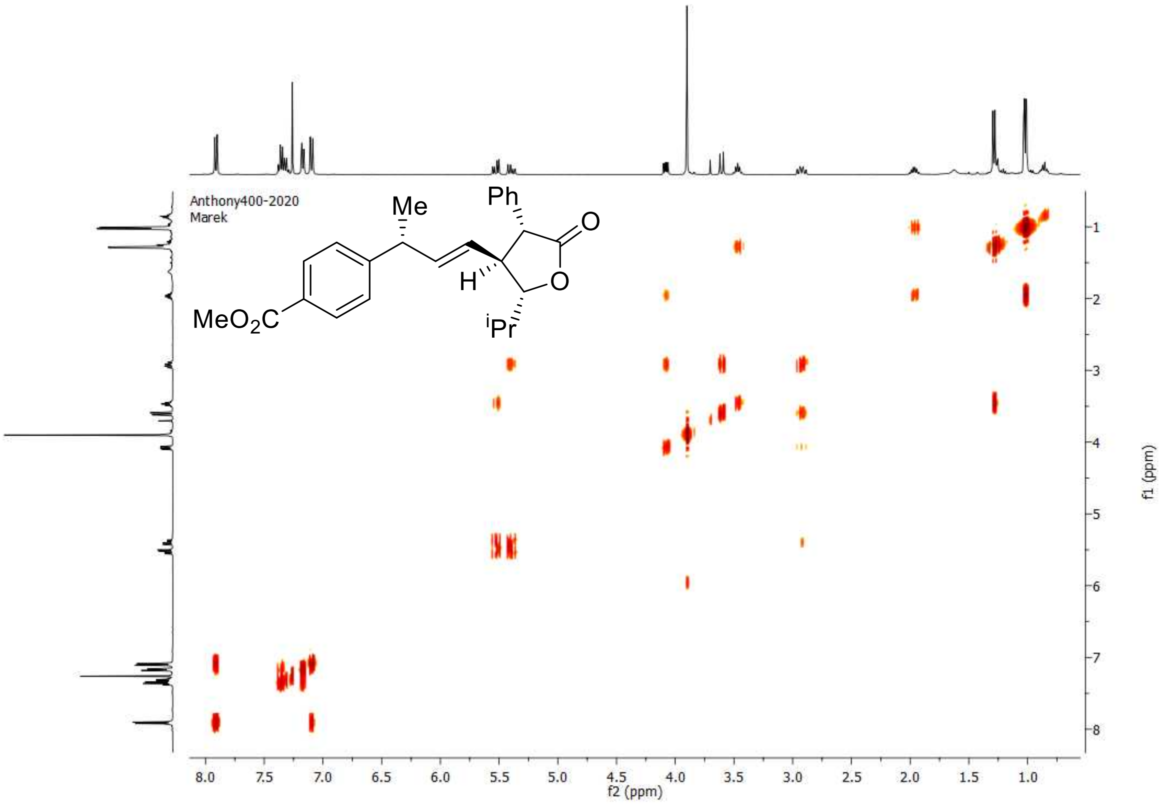
Anthony400-2020
Marek
Me
Ph
O
H
O
MeO2C
iPr
f2 (ppm)
f1 (ppm)

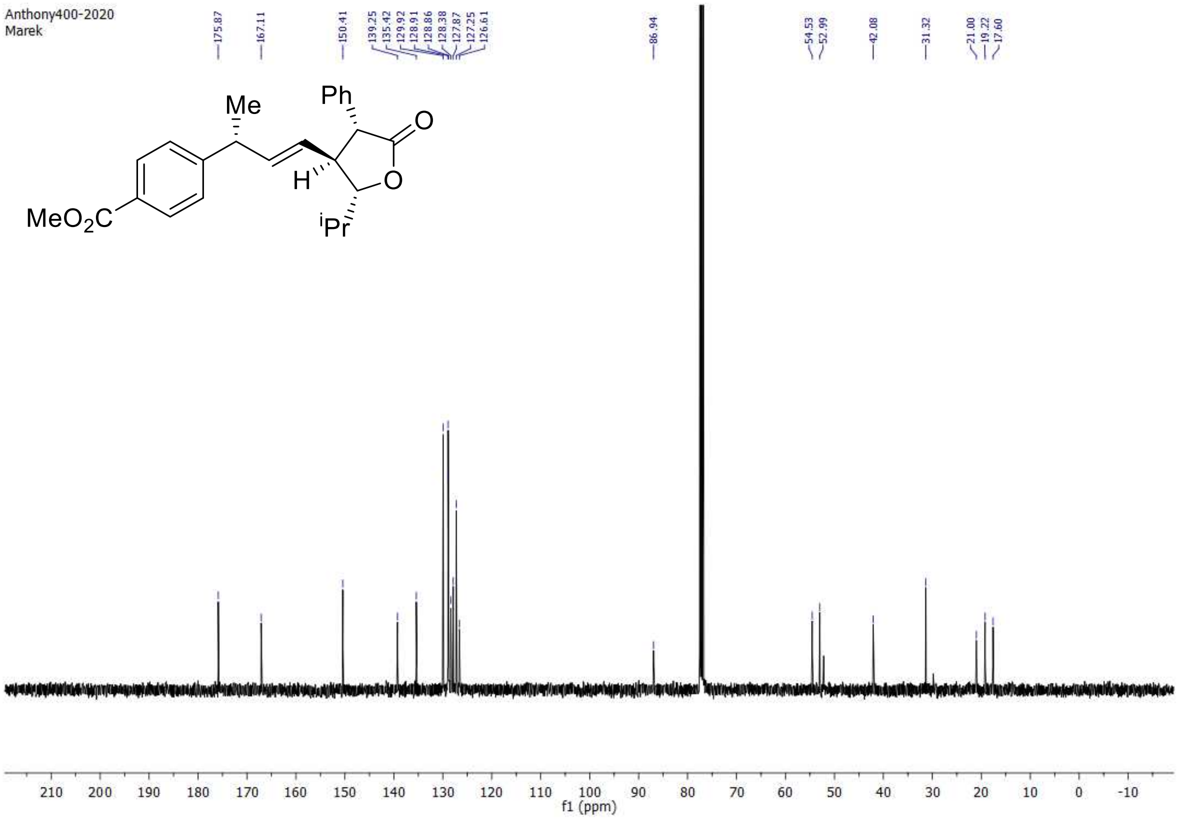
Anthony400-2020
Marek
175.87
167.11
150.41
139.25
135.42
129.92
128.91
128.86
128.38
127.87
127.25
126.61
86.94
54.53
52.99
42.08
31.32
21.00
19.22
17.60
Me
Ph
O
H
O
MeO2C
iPr
f1 (ppm)

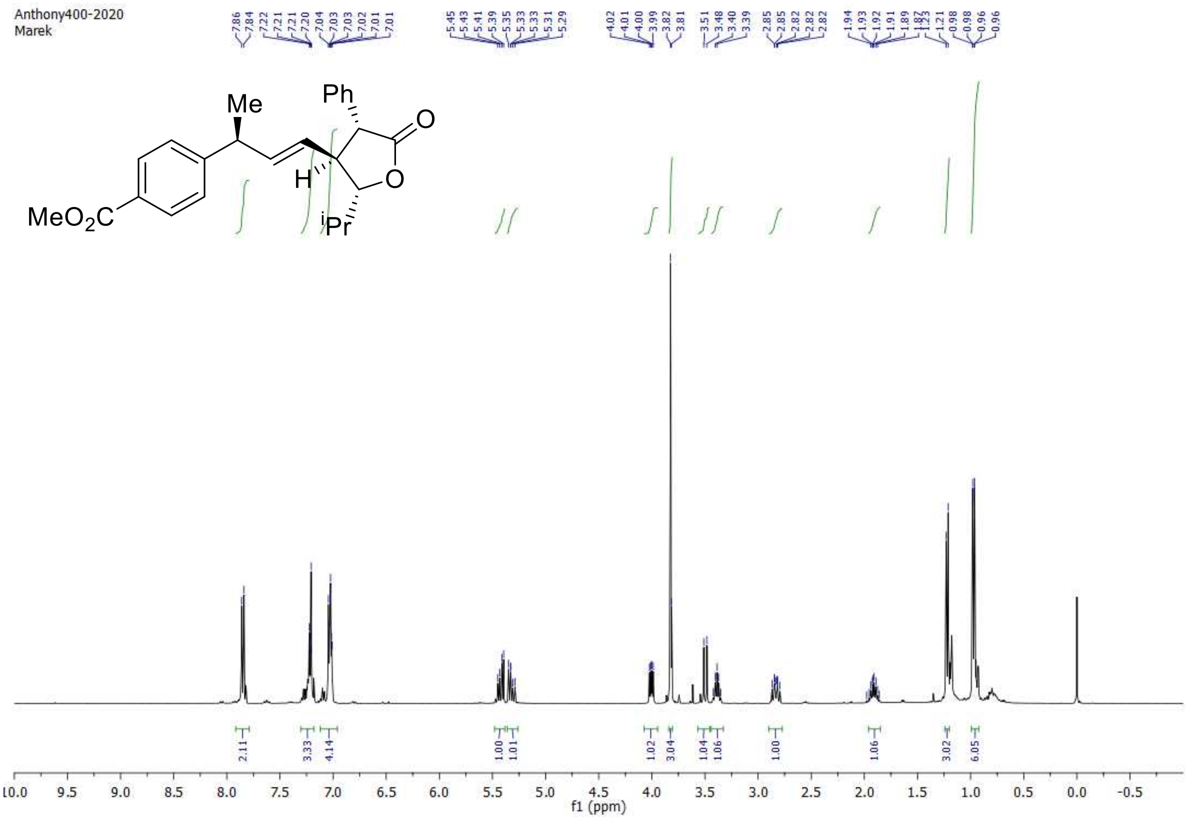
Anthony400-2020
Marek
Me
Ph
O
H
O
MeO2C
iPr
f1 (ppm)

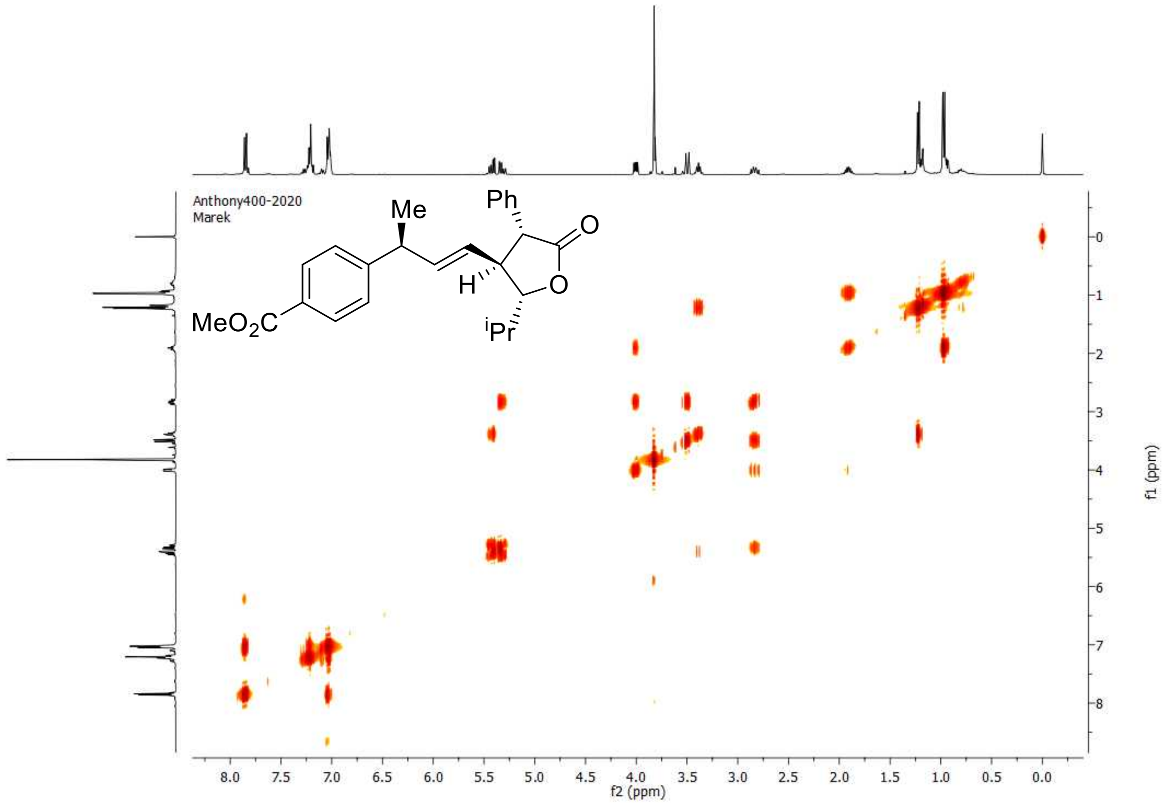
Anthony400-2020
Marek
Me
Ph
O
H
O
MeO2C
iPr
f2 (ppm)
f1 (ppm)

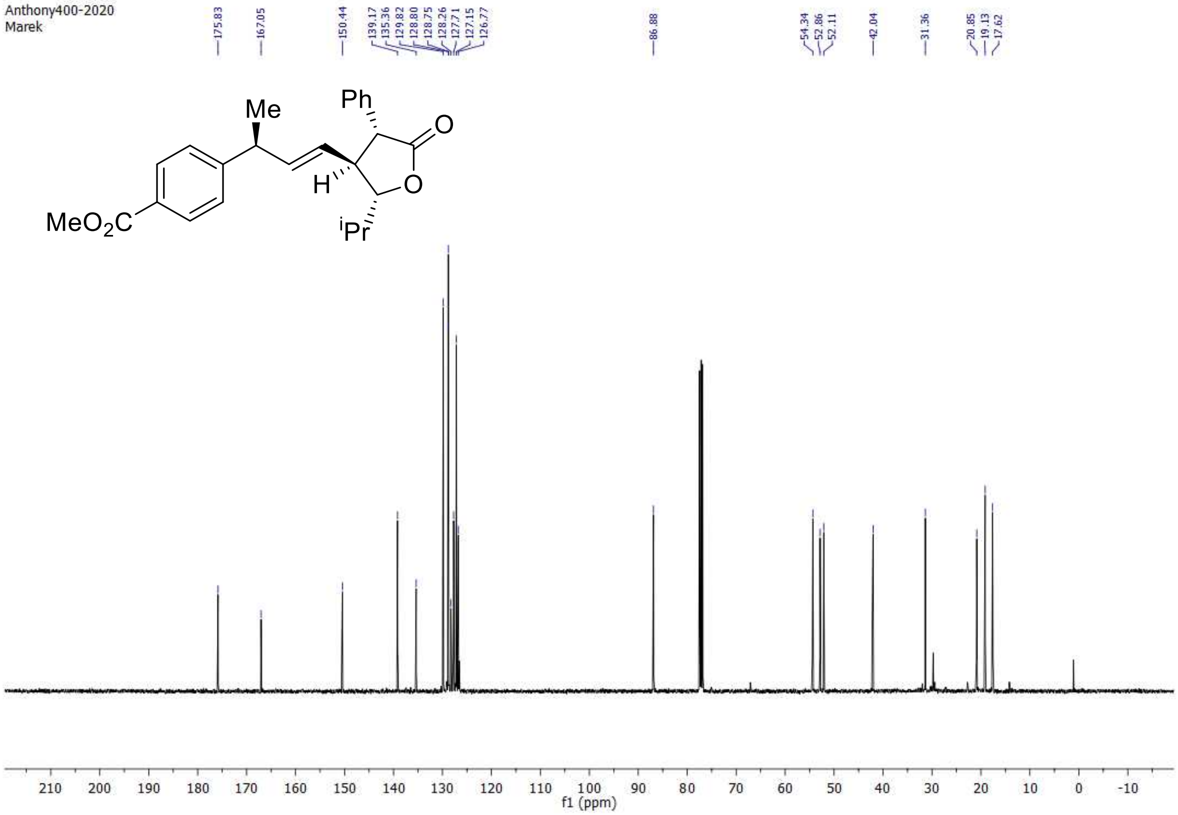
Anthony400-2020
Marek
175.83
167.05
150.44
139.17
135.36
129.82
128.80
128.75
128.26
127.71
127.15
126.77
86.88
54.34
52.86
52.11
42.04
31.36
20.85
19.13
17.62
Me
Ph
O
H
O
MeO2C
iPr
210
200
190
180
170
160
150
140
130
120
110
100
90
80
70
60
50
40
30
20
10
0
-10
f1 (ppm)

Overlay of ^{1}HNMR of 10a and 10b

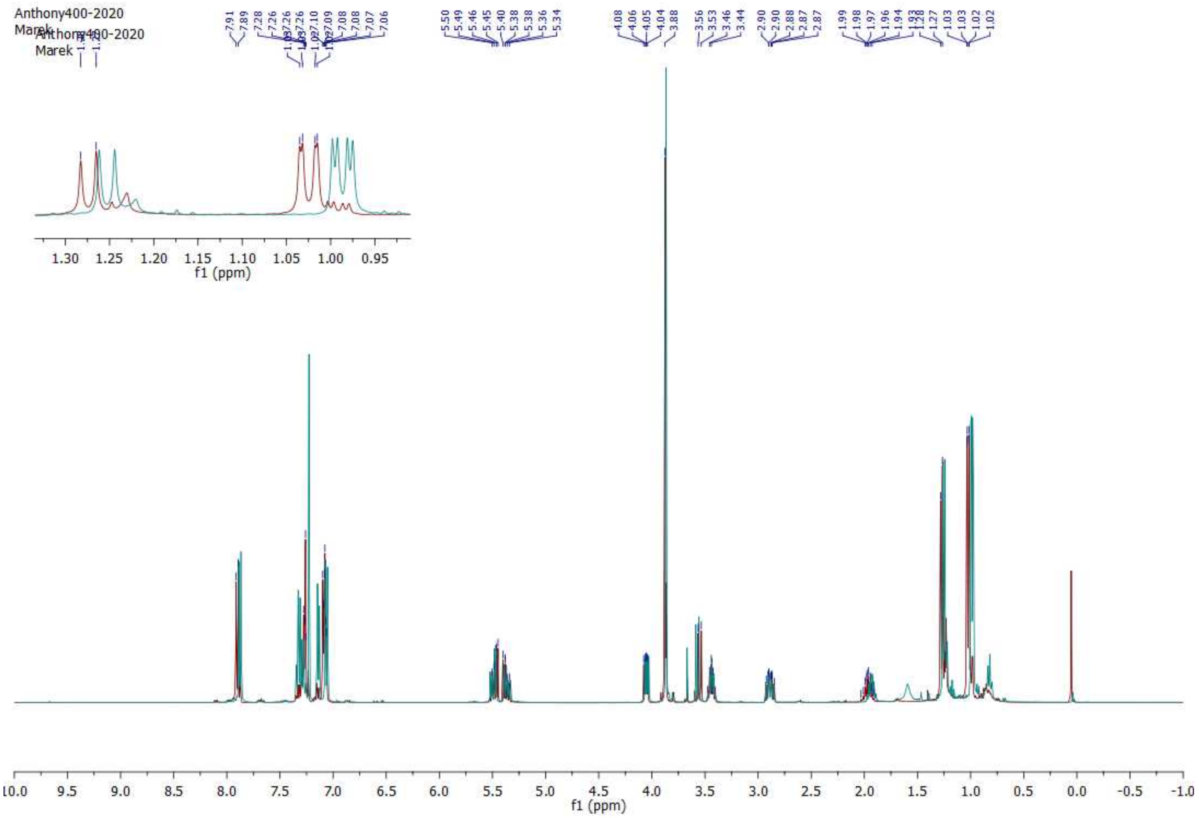

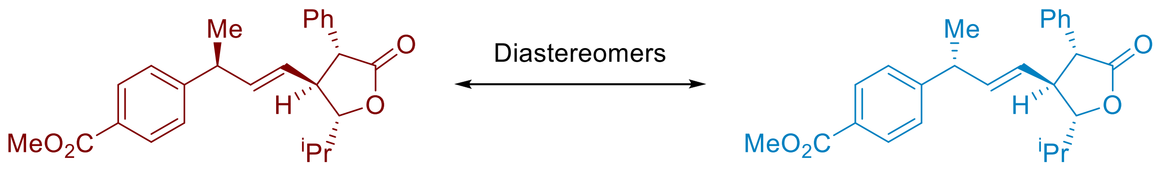

10a (in red)
dr = 10:1

10b (in blue)
dr > 95:5

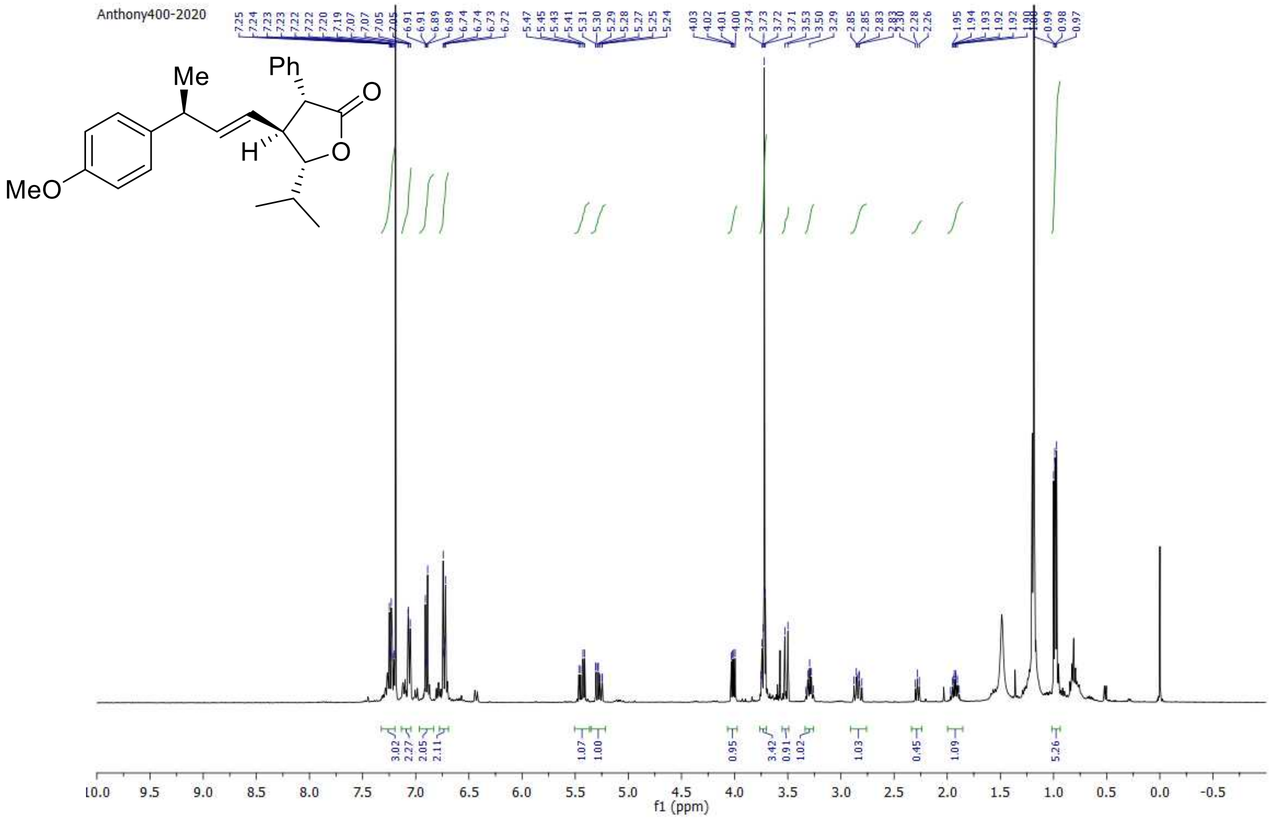
Anthony400-2020
Me
Ph
O
H
O
MeO
f1 (ppm)

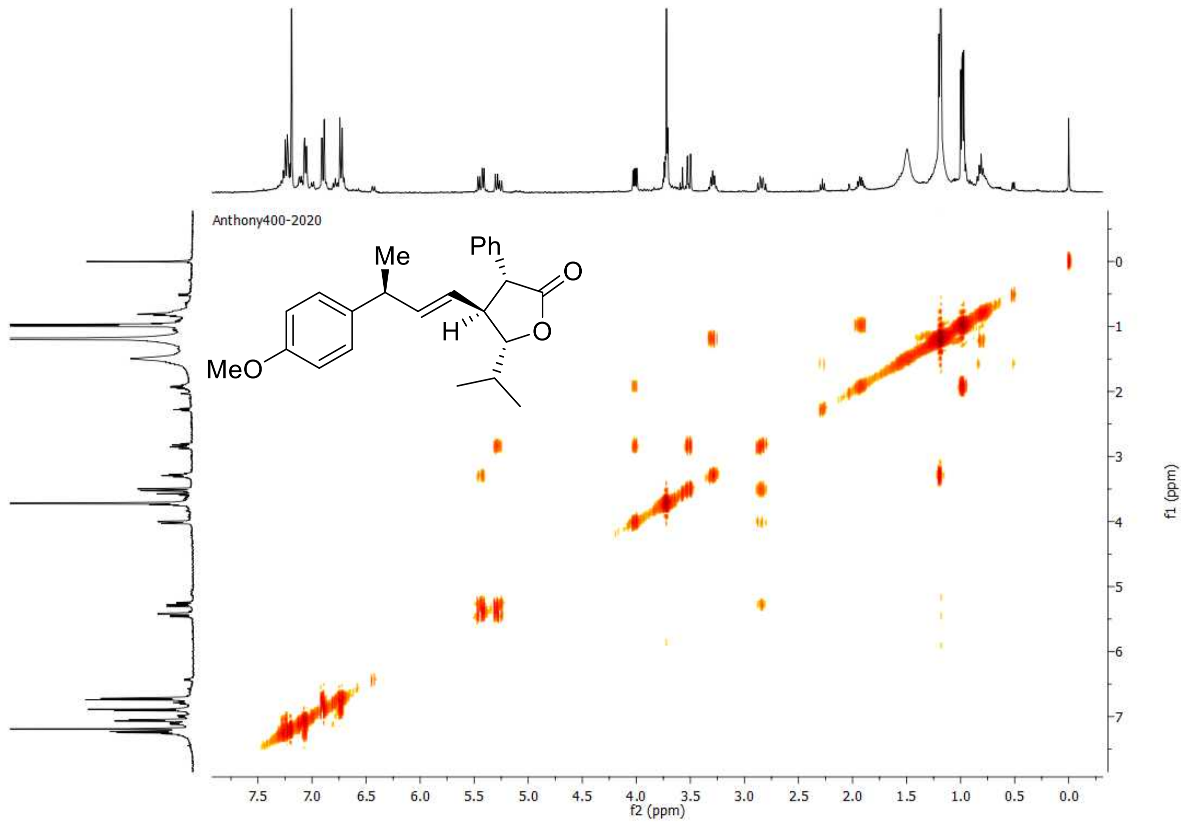
Anthony400-2020
Me
Ph
O
H
O
MeO
f2 (ppm)
f1 (ppm)

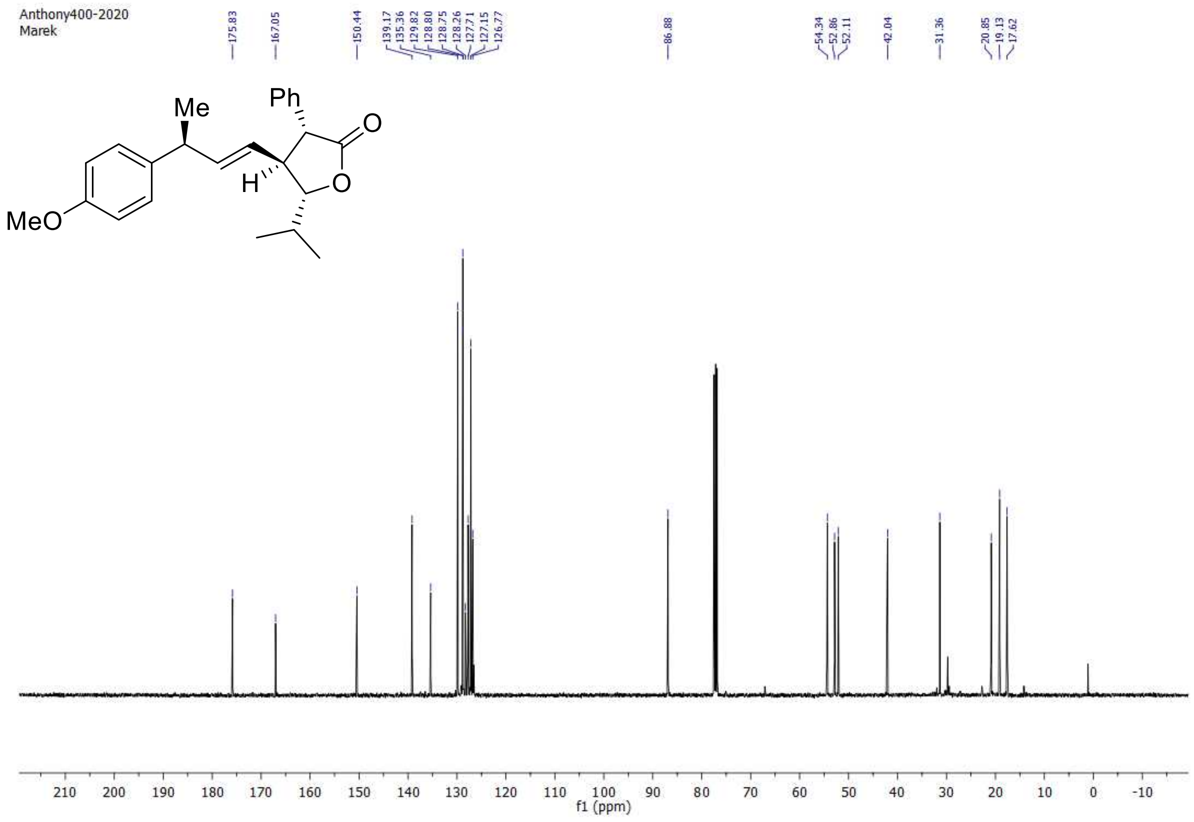
Anthony400-2020
Marek
175.83
167.05
150.44
139.17
135.36
129.82
128.80
128.75
128.26
127.71
127.15
126.77
86.88
54.34
52.86
52.11
42.04
31.36
20.85
19.13
17.62
Me
Ph
O
H
O
MeO
f1 (ppm)

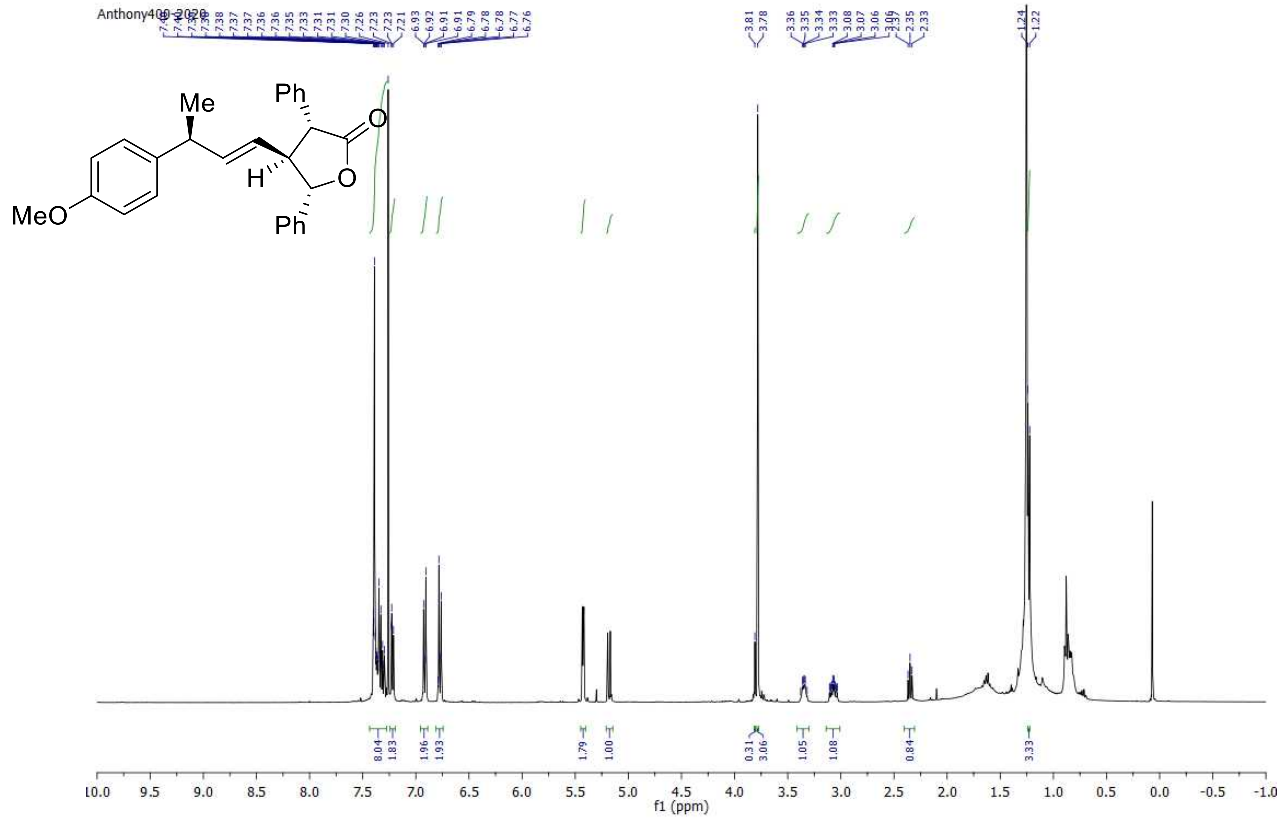

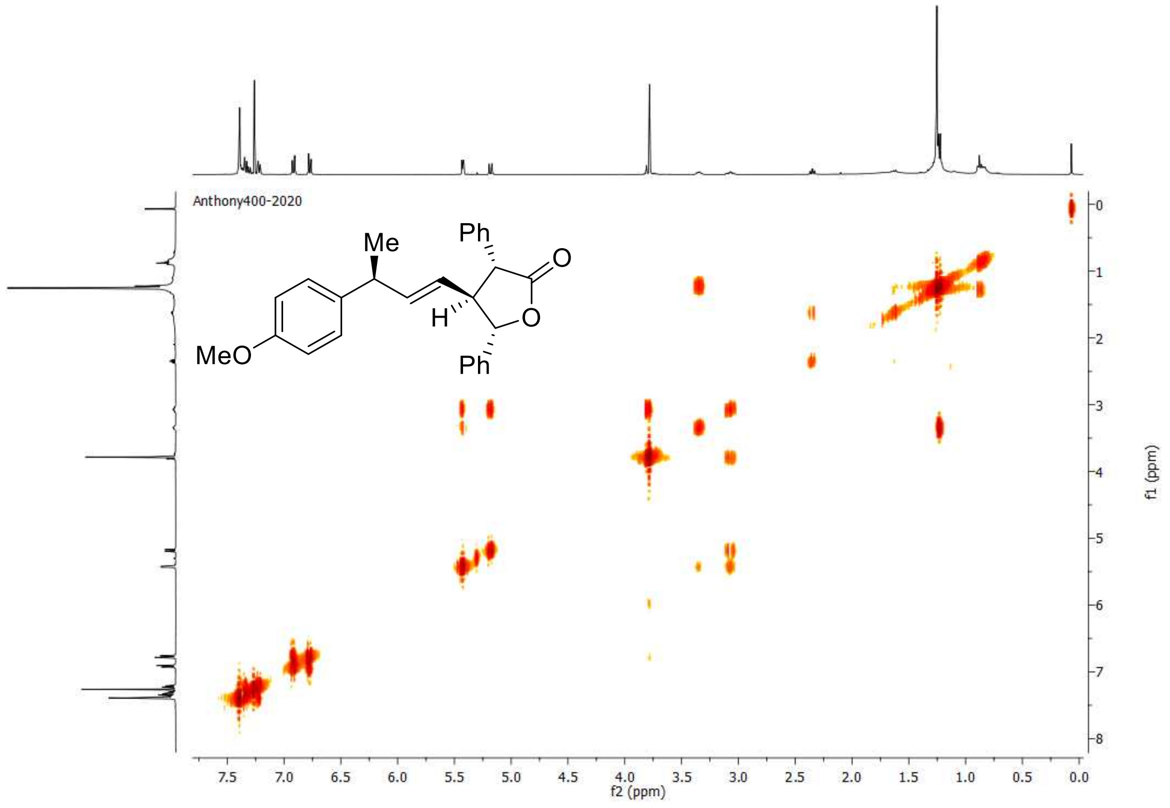

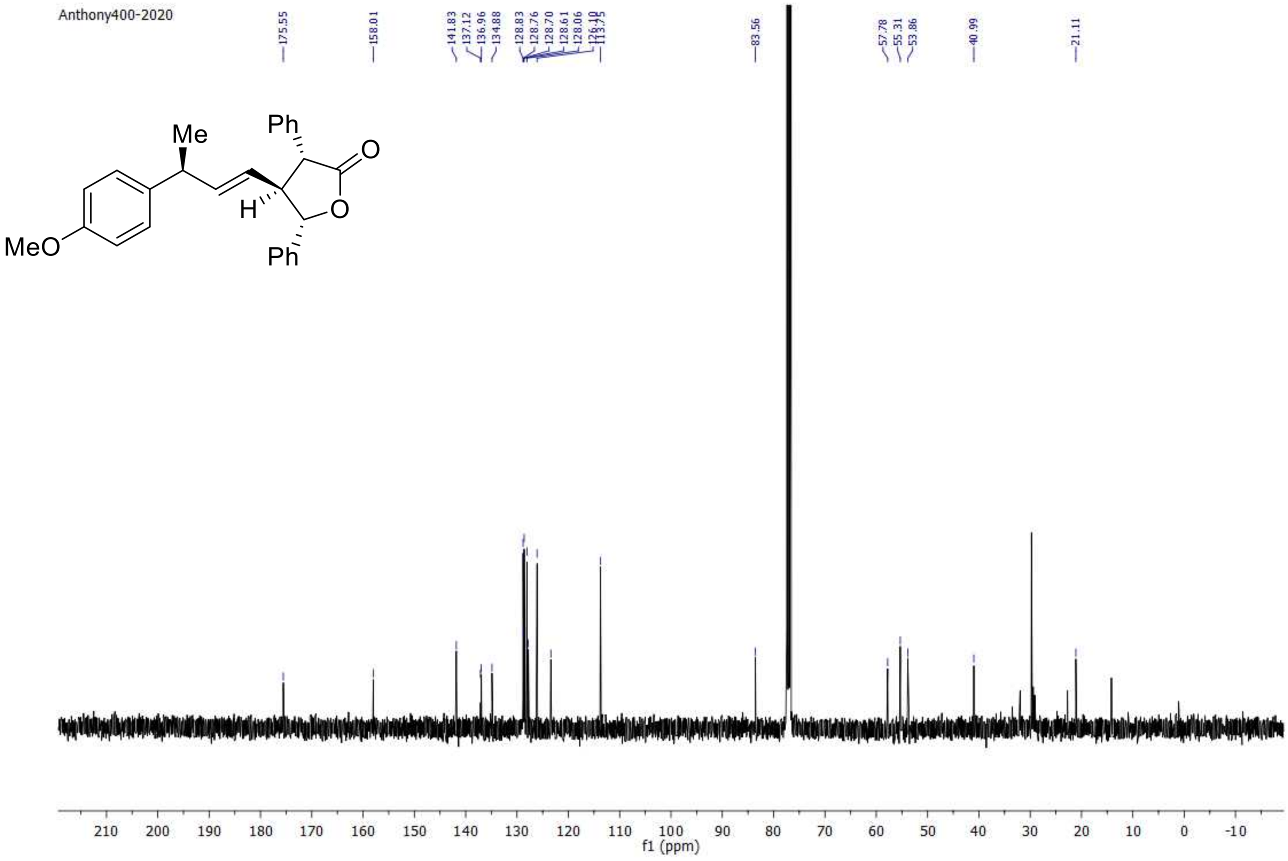
Anthony400-2020
175.55
158.01
141.83
137.12
136.96
134.88
128.83
128.76
128.70
128.61
128.06
126.10
113.75
83.56
57.78
55.31
53.86
40.99
21.11
Me
Ph
O
H
O
MeO
Ph
210 200 190 180 170 160 150 140 130 120 110 100 90 80 70 60 50 40 30 20 10 0 -10
f1 (ppm)

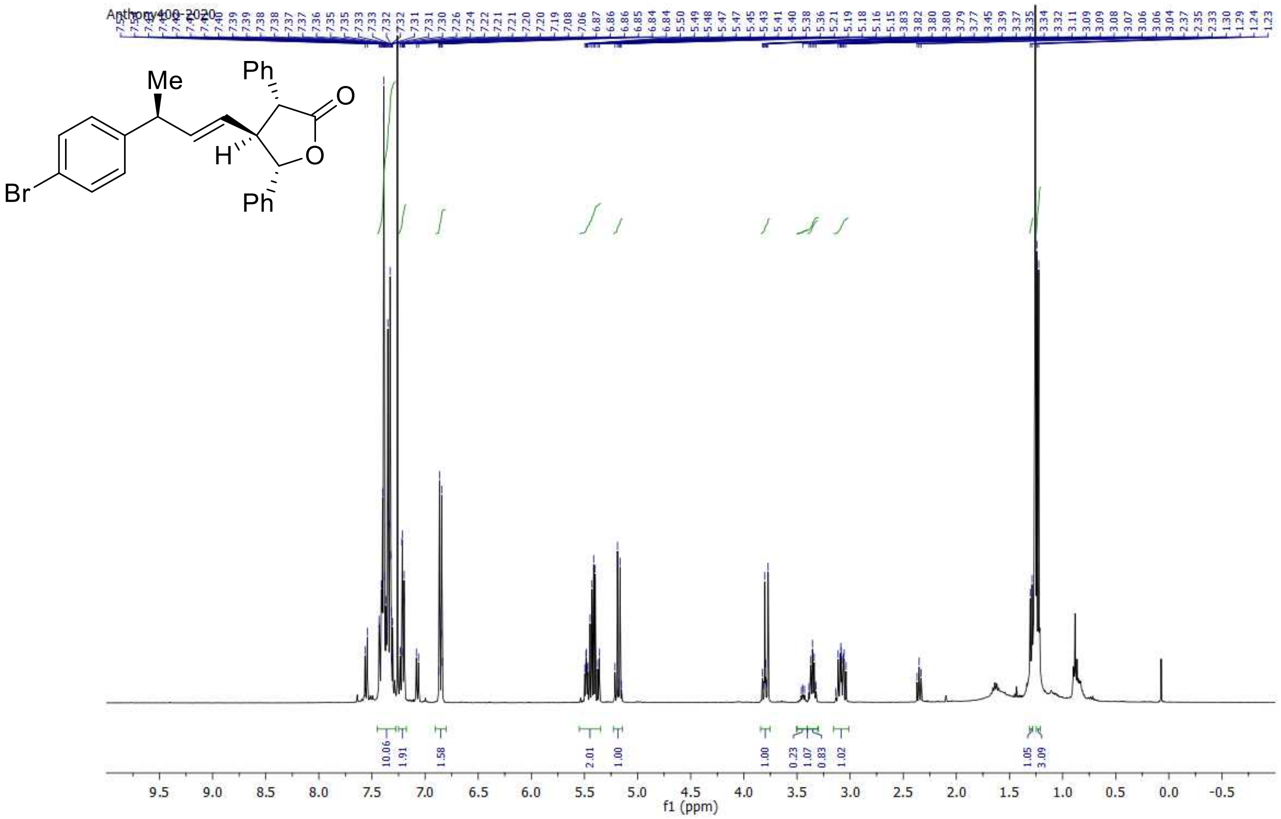
Anthony400-2020
Me
Ph
O
H
O
Ph
Br
f1 (ppm)

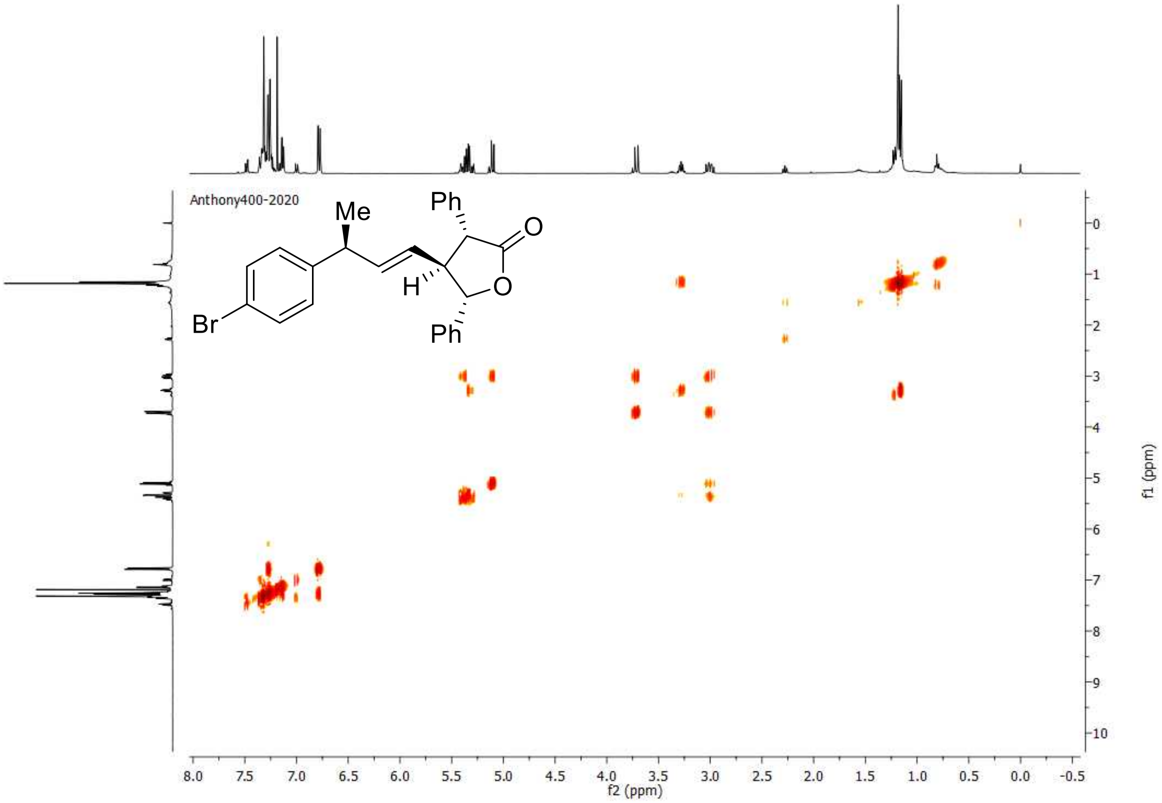
Anthony400-2020
Me
Ph
O
H
O
Ph
Br
f2 (ppm)
f1 (ppm)

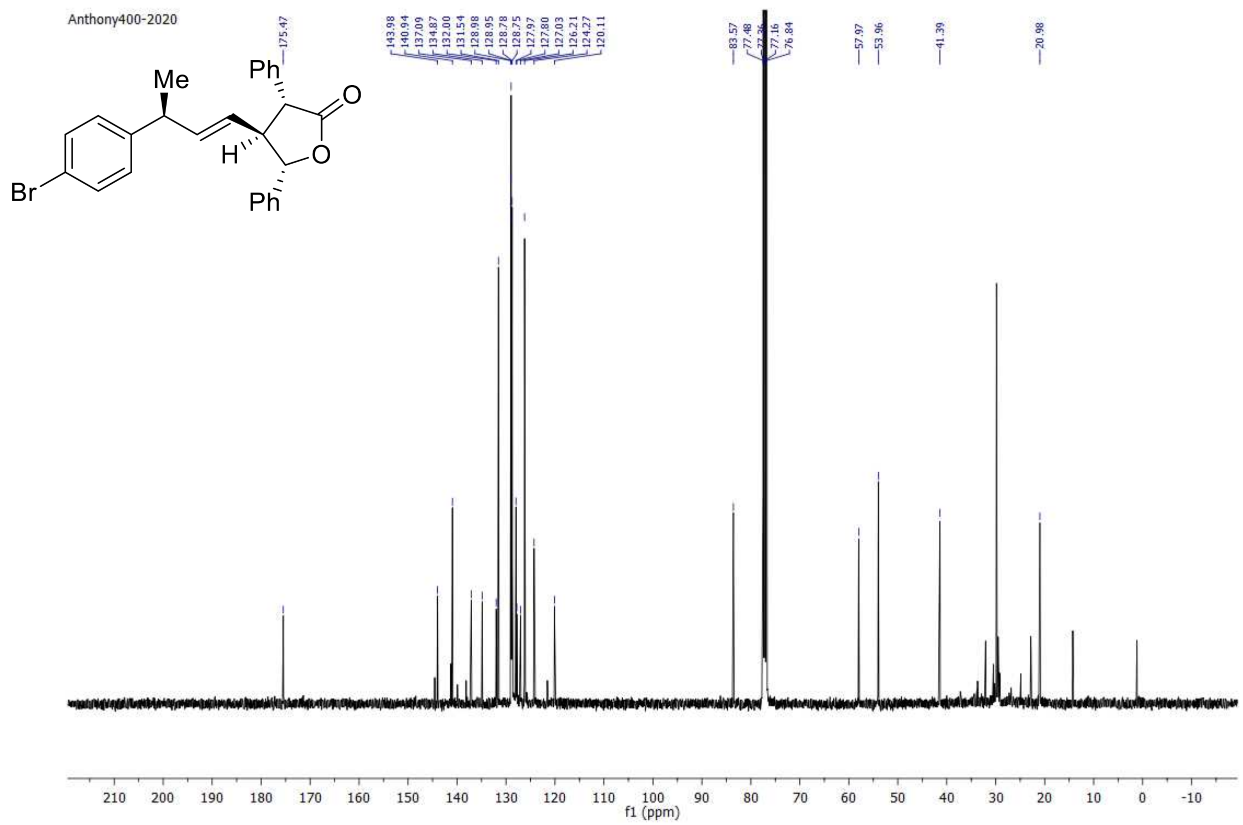

Anthony400-2020
Me
Ph
O
H
O
Br
Ph
175.47
143.98
140.94
137.09
134.87
132.00
131.54
128.98
128.95
128.78
128.75
127.97
127.80
127.03
126.21
124.27
120.11
83.57
77.48
77.16
76.84
57.97
53.96
41.39
20.98
210 200 190 180 170 160 150 140 130 120 110 100 90 80 70 60 50 40 30 20 10 0 -10
f1 (ppm)

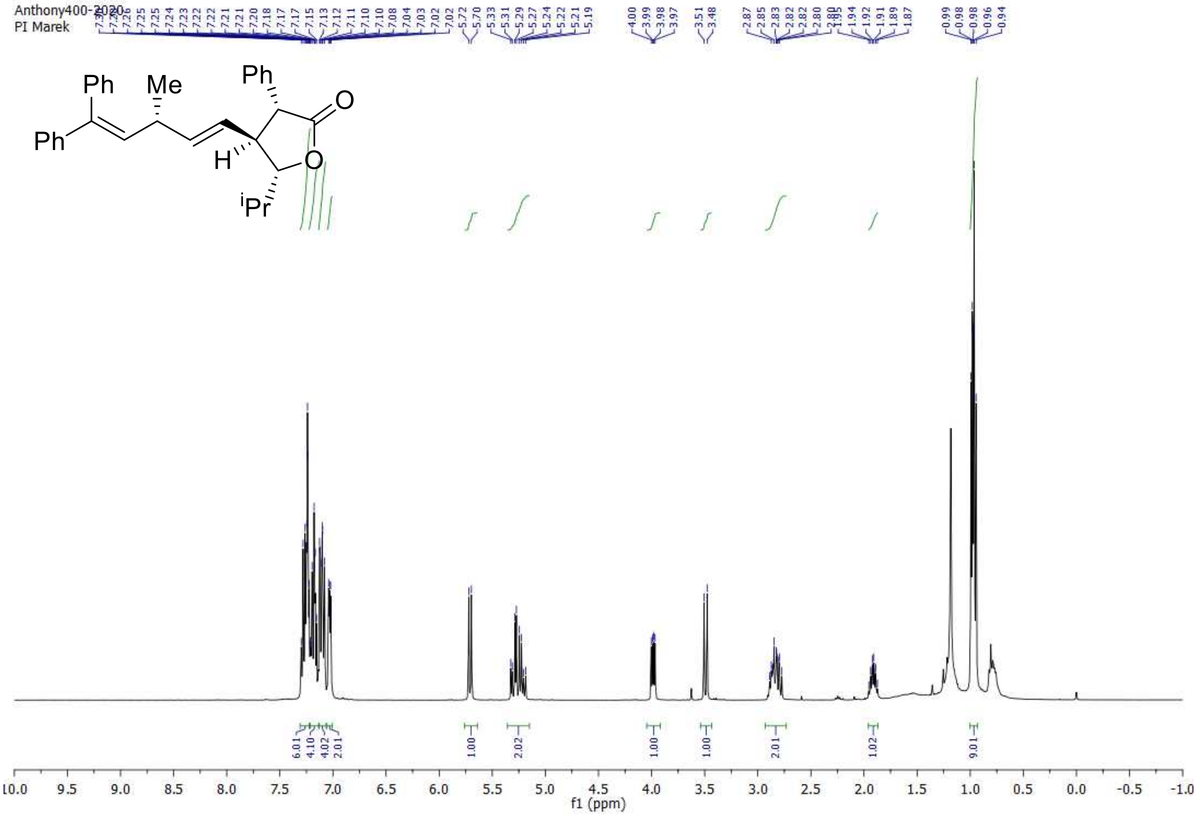
Anthony400-2020
PI Marek
Ph
Me
Ph
O
Ph
H
O
iPr
f1 (ppm)

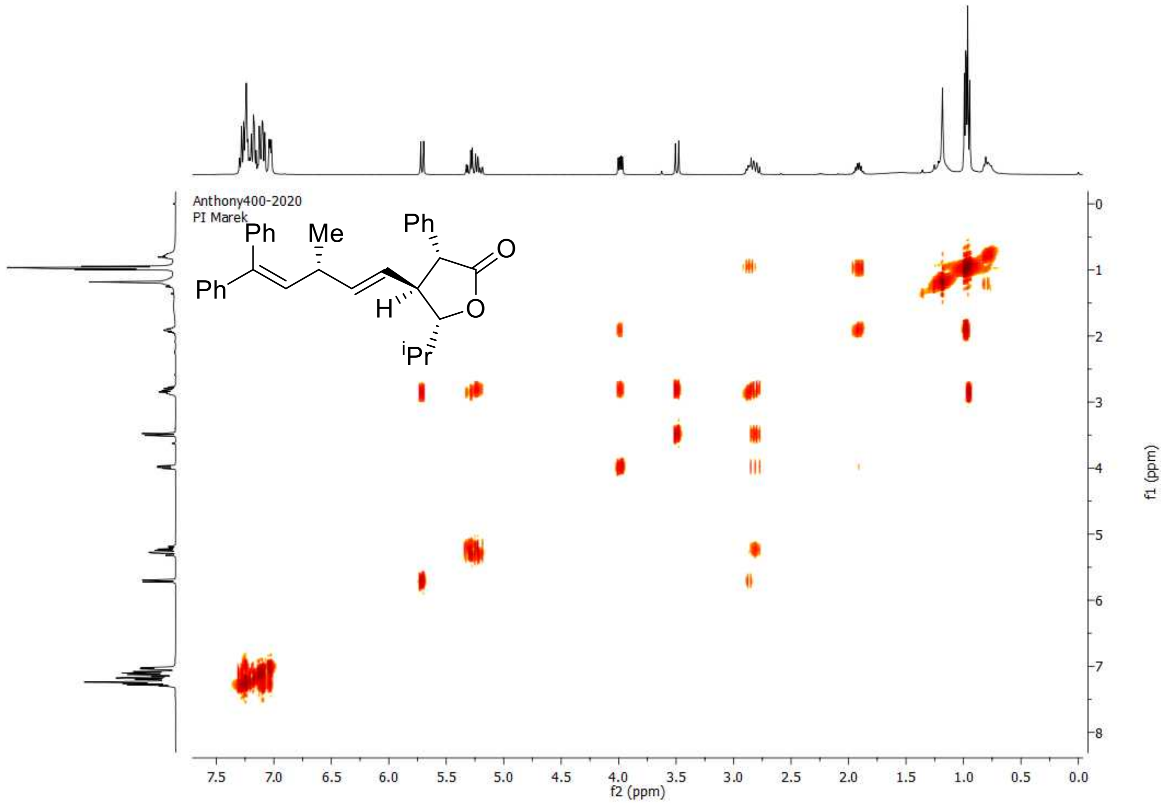
Anthony400-2020
PI Marek
Ph
Me
Ph
O
Ph
H
O
iPr
f2 (ppm)
f1 (ppm)

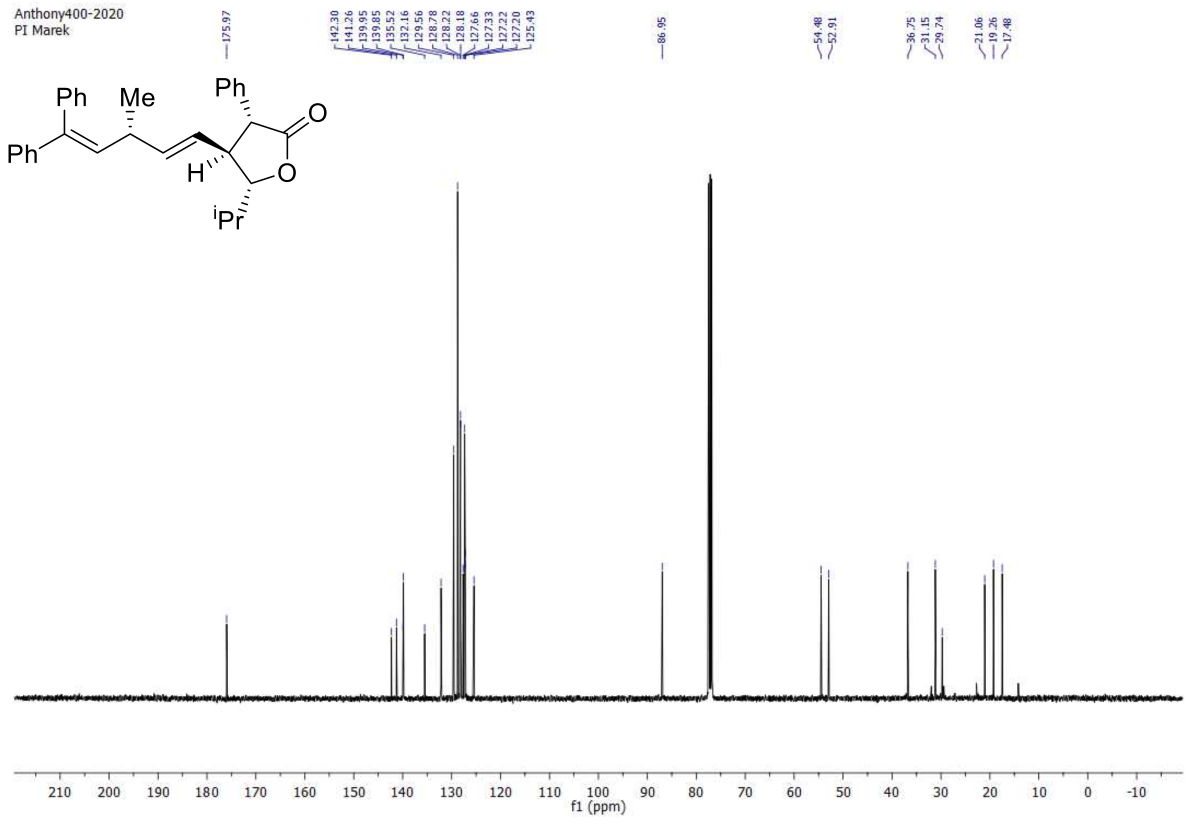
Anthony400-2020
PI Marek
175.97
142.30
141.26
139.95
139.85
135.52
132.16
129.56
128.78
128.22
128.18
127.66
127.33
127.22
127.20
125.43
86.95
54.48
52.91
36.75
31.15
29.74
21.06
19.26
17.48
Ph
Me
Ph
Ph
O
H
O
iPr
210 200 190 180 170 160 150 140 130 120 110 100 90 80 70 60 50 40 30 20 10 0 -10
f1 (ppm)

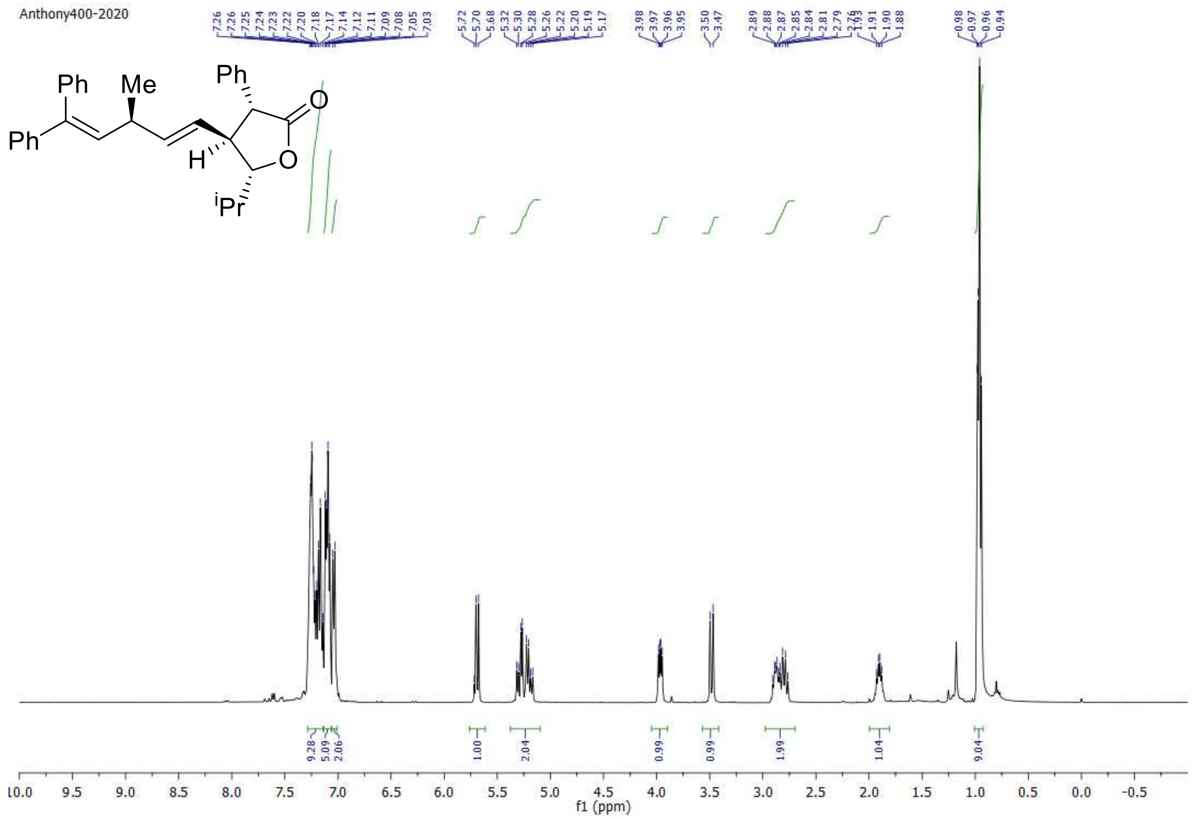
Anthony400-2020
Ph
Me
Ph
O
Ph
H
O
iPr
f1 (ppm)

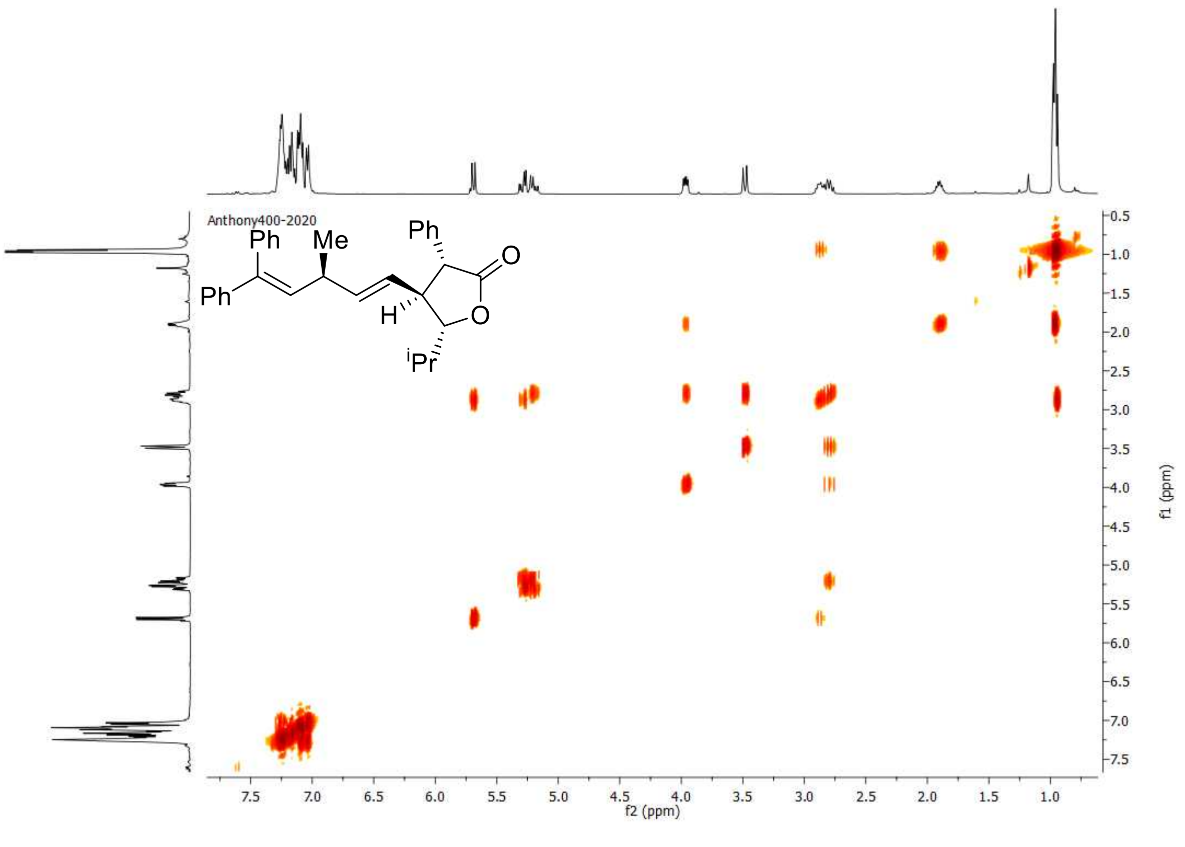
Anthony400-2020
Ph
Me
Ph
O
Ph
H
O
iPr
f2 (ppm)
f1 (ppm)

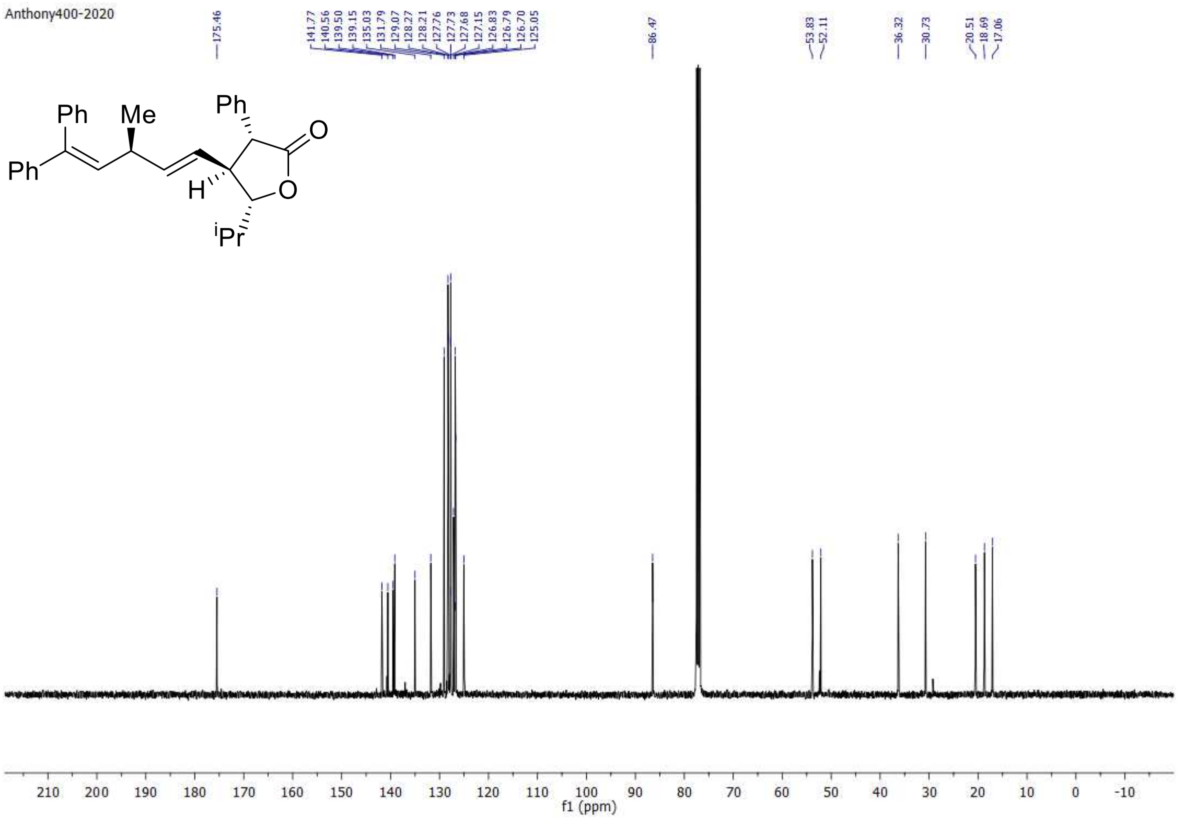
Anthony400-2020
175.46
141.77
140.56
139.50
139.15
135.03
131.79
129.07
128.27
128.21
127.76
127.73
127.68
127.15
126.83
126.79
126.70
125.05
86.47
53.83
52.11
36.32
30.73
20.51
18.69
17.06
Ph
Me
Ph
O
Ph
H
O
iPr
210 200 190 180 170 160 150 140 130 120 110 100 90 80 70 60 50 40 30 20 10 0 -10
f1 (ppm)

Overlay of ¹HNMR of 10g and 10h

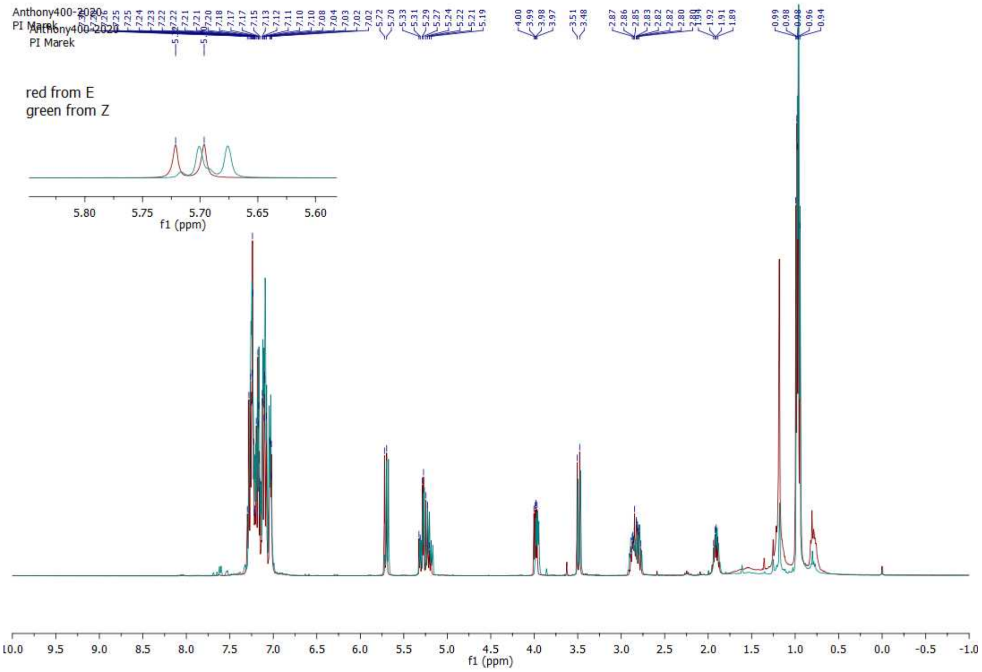

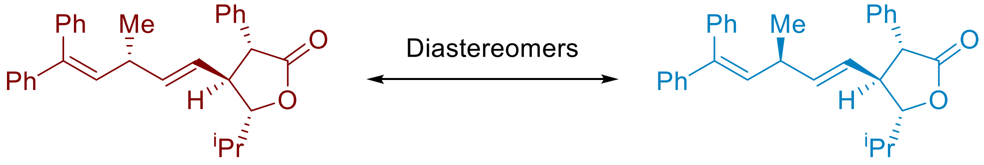

10g (in red)
dr > 95:5

10h (in blue)
dr > 95:5

Overlay of ^{13}CNMR of 10g and 10h

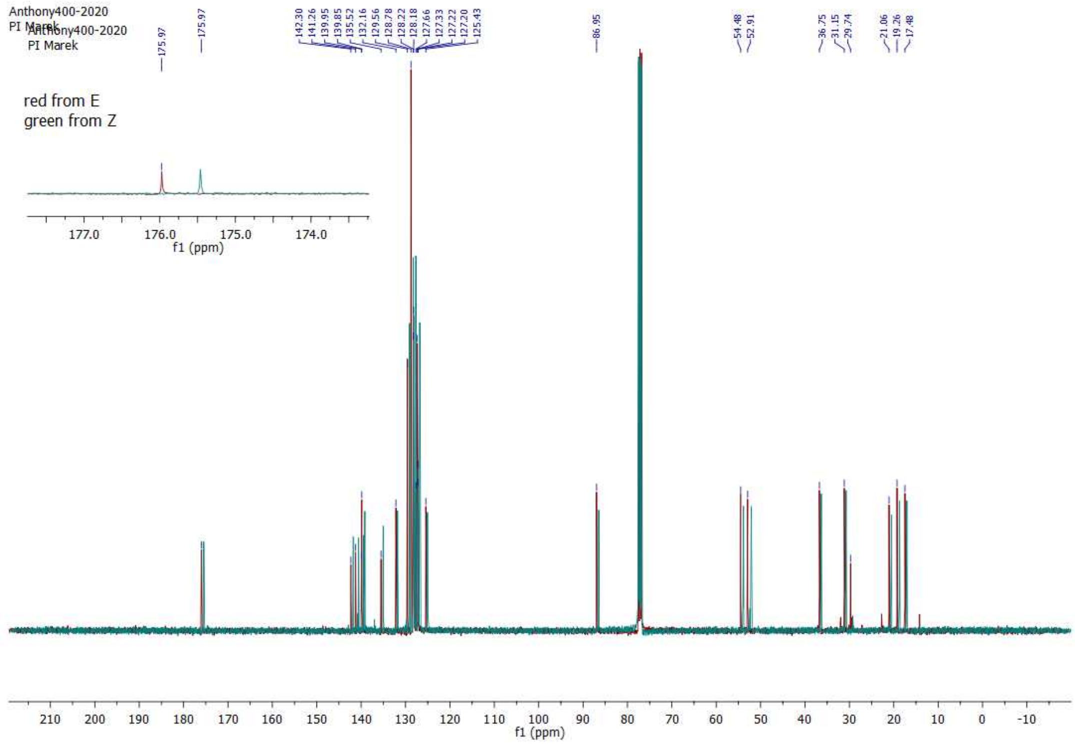

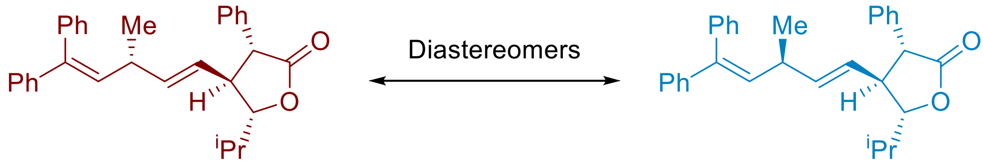

10g (in red)
dr > 95:5

10h (in blue)
dr > 95:5

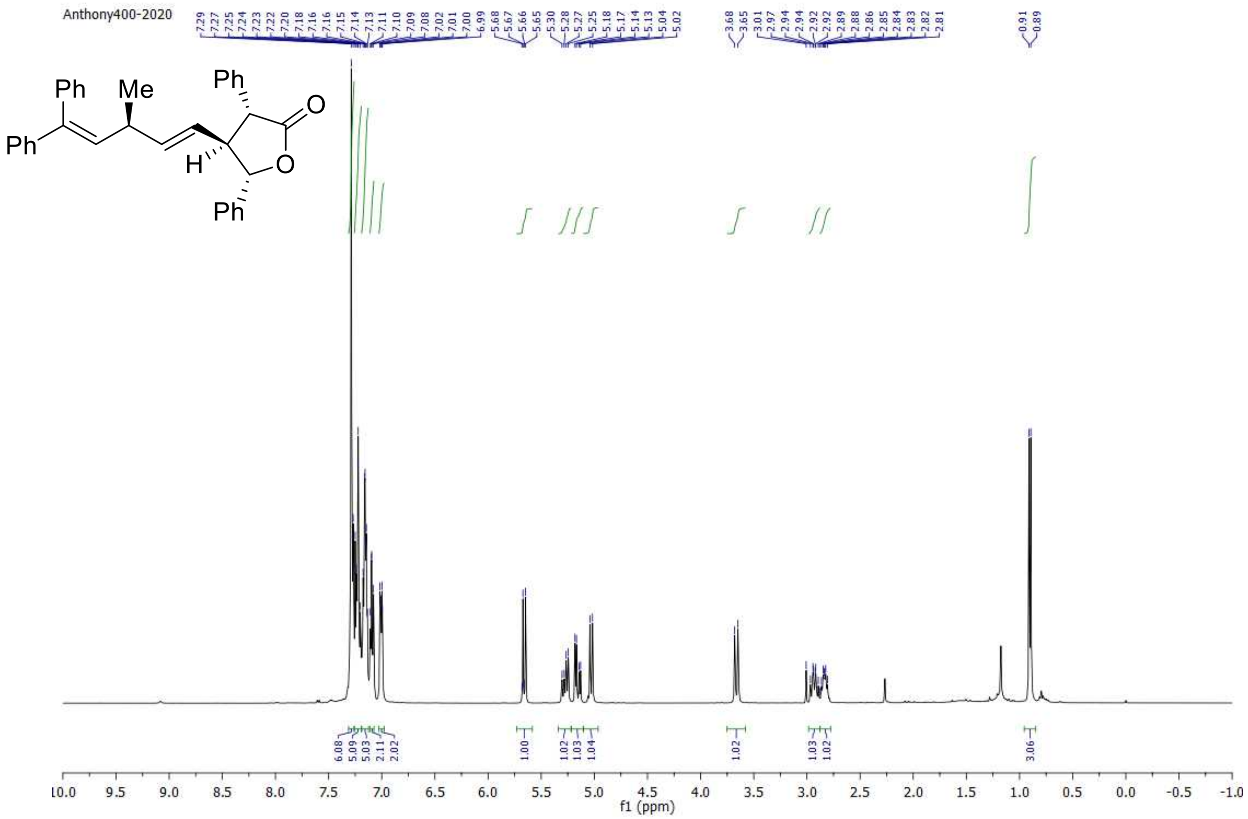

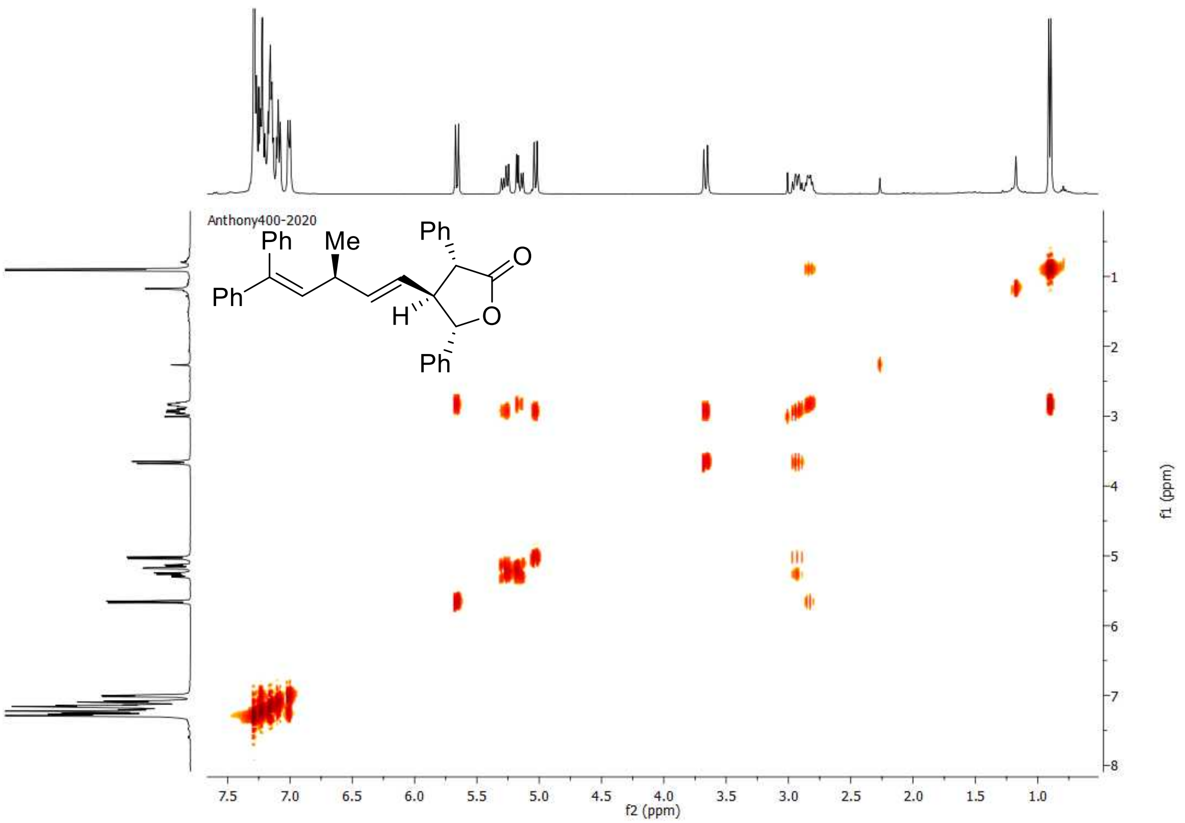

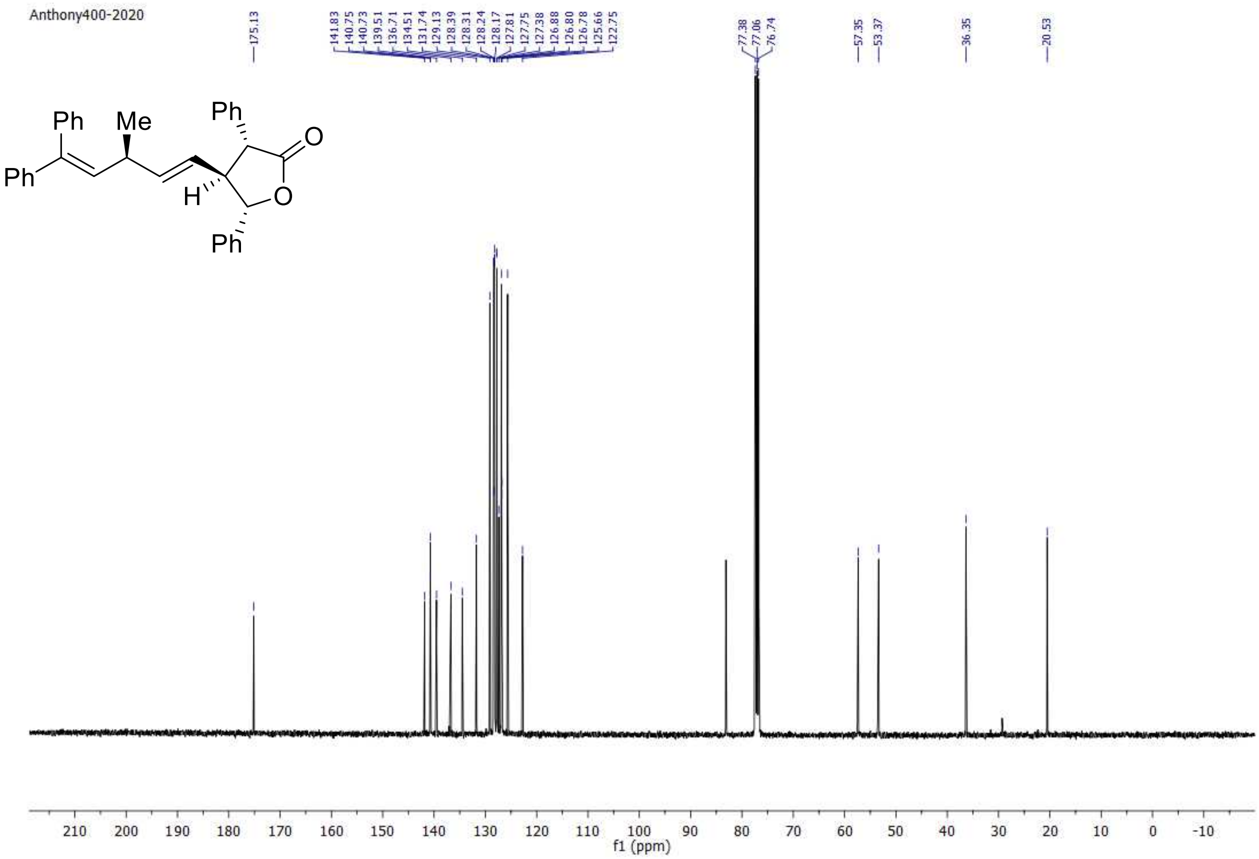
Anthony400-2020
175.13
141.83
140.75
140.73
139.51
136.71
134.51
131.74
129.13
128.39
128.31
128.24
128.17
127.81
127.75
127.38
126.88
126.80
126.78
125.66
122.75
77.38
77.06
76.74
57.35
53.37
36.35
20.53
Ph
Me
Ph
O
Ph
H
O
Ph
210 200 190 180 170 160 150 140 130 120 110 100 90 80 70 60 50 40 30 20 10 0 -10
f1 (ppm)

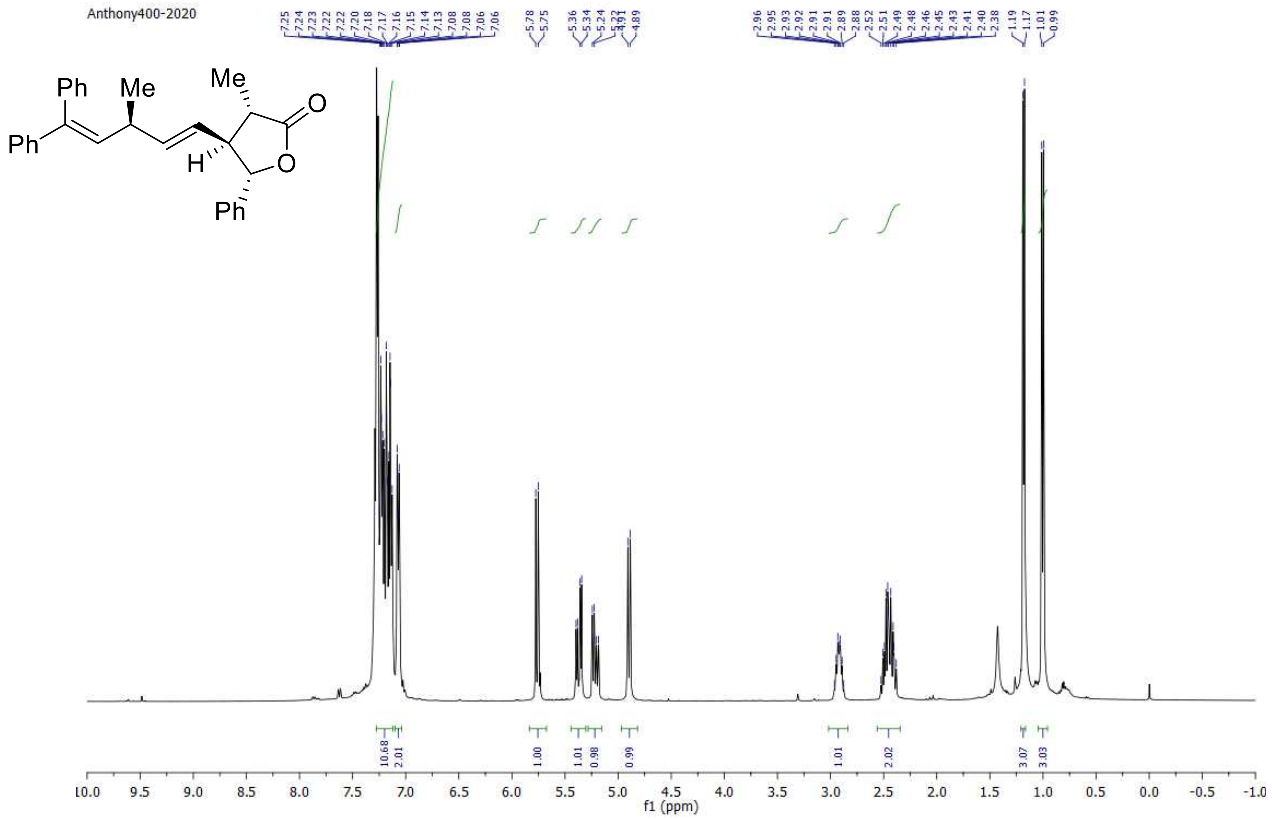
Anthony400-2020
Ph
Me
Me
O
Ph
H
O
Ph
f1 (ppm)

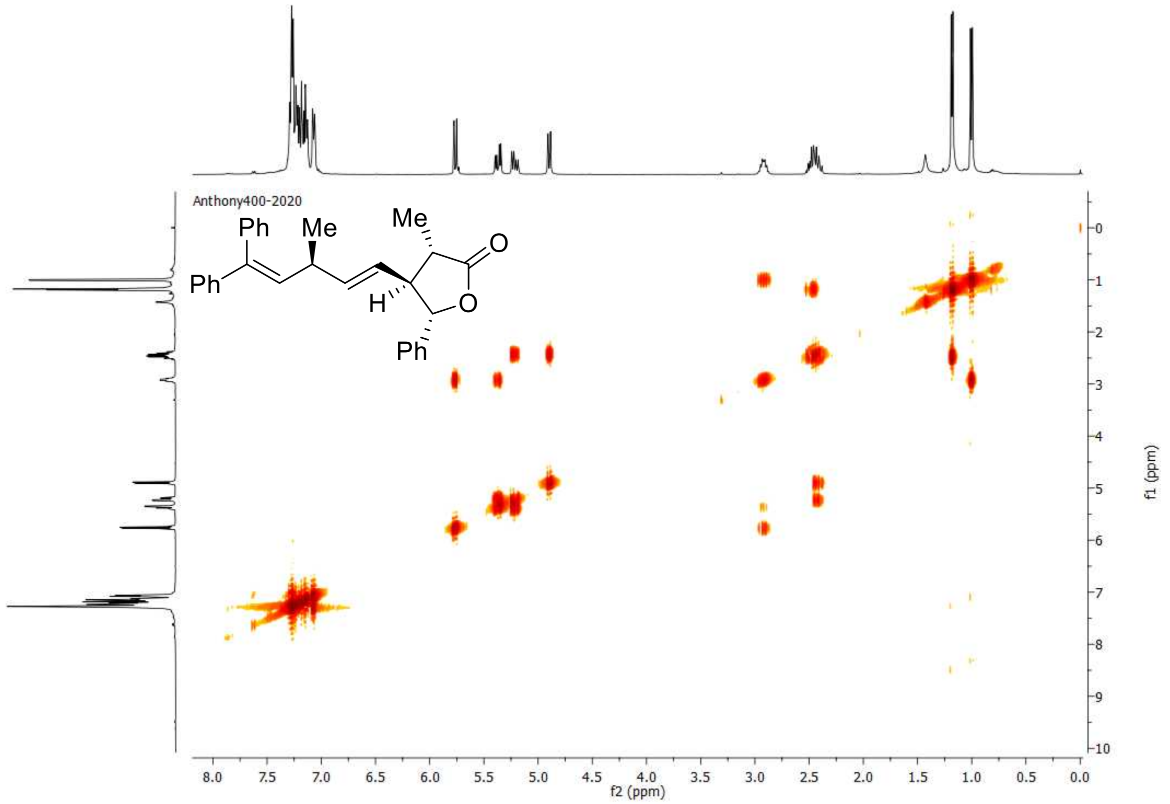
Anthony400-2020
f2 (ppm)
f1 (ppm)

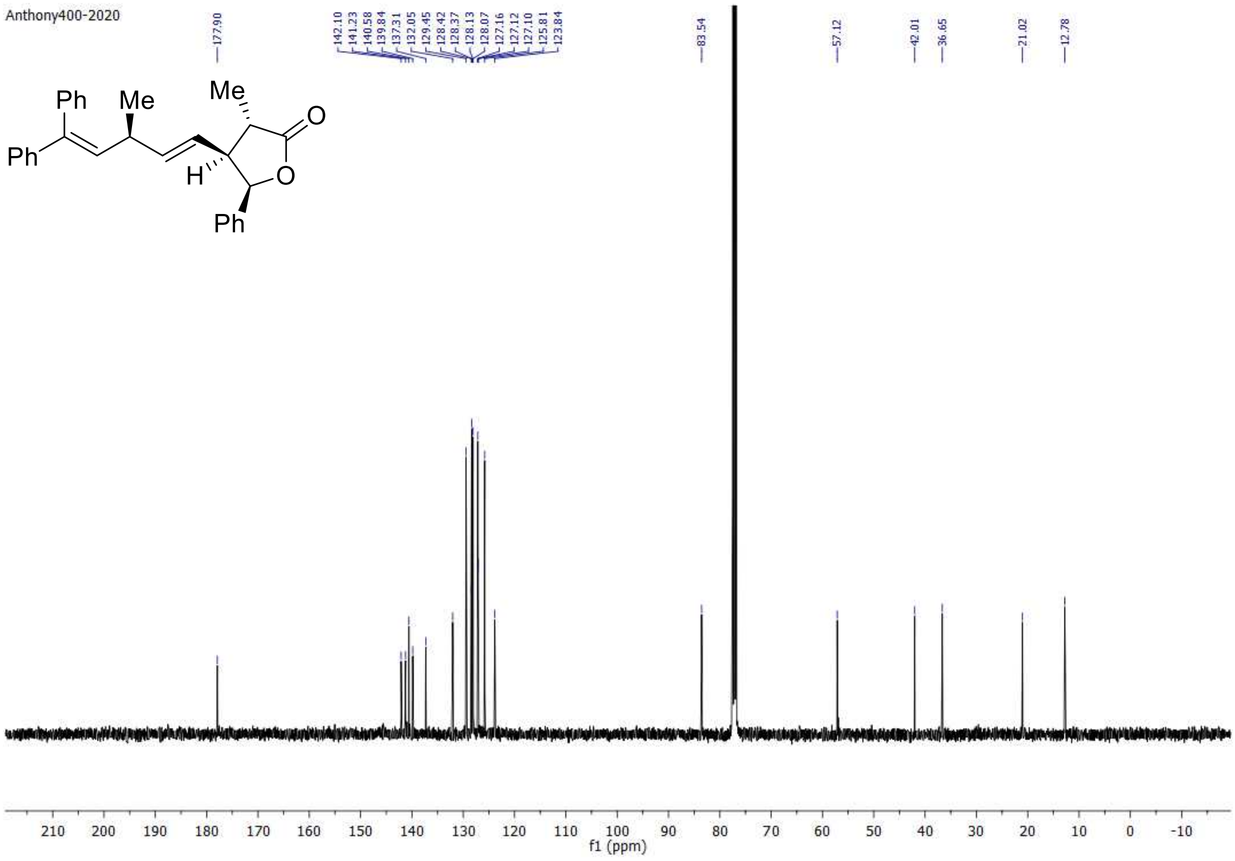
Anthony400-2020
177.90
142.10
141.23
140.58
139.84
137.31
132.05
129.45
128.42
128.37
128.13
128.07
127.16
127.12
127.10
125.81
123.84
83.54
57.12
42.01
36.65
21.02
12.78
Ph
Ph
Me
Me
O
O
H
Ph
210 200 190 180 170 160 150 140 130 120 110 100 90 80 70 60 50 40 30 20 10 0 -10
f1 (ppm)

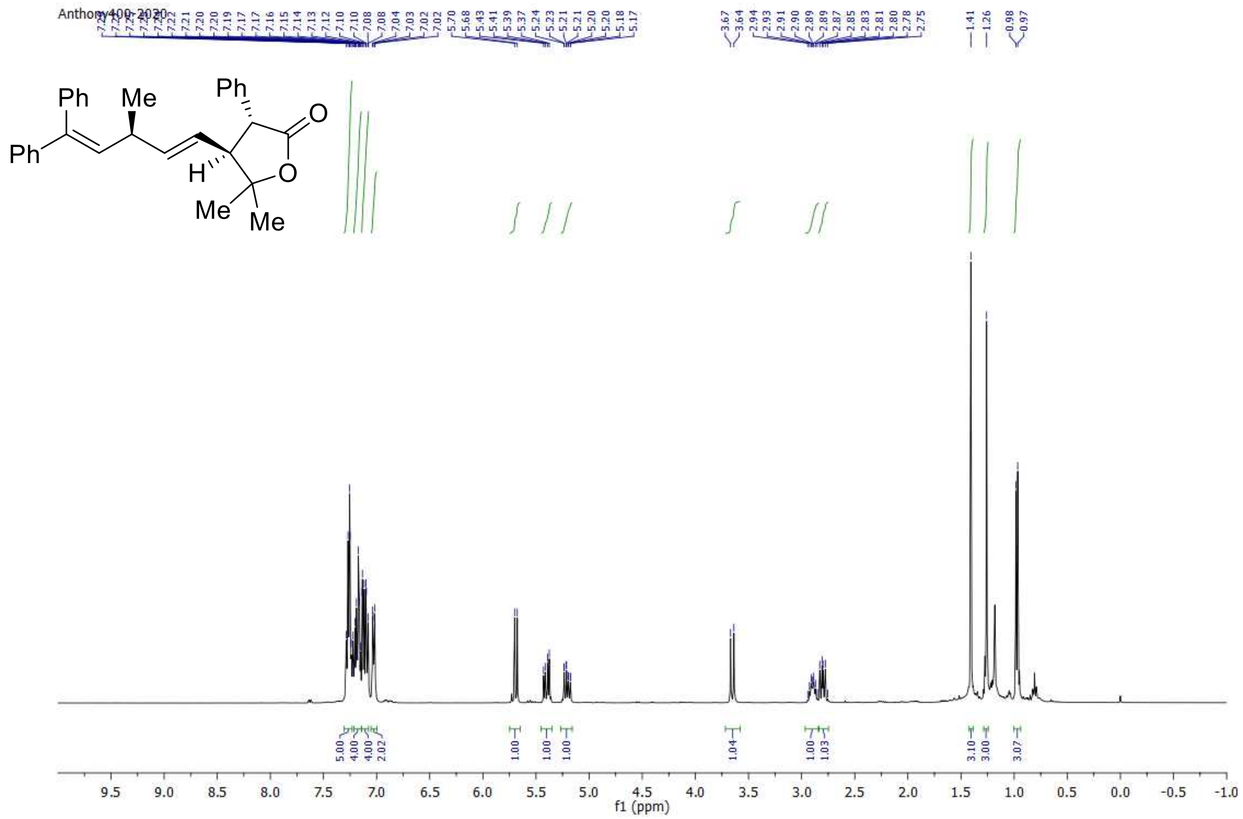
Anthony400-2020
Ph
Me
Ph
O
Ph
H
O
Me
Me
f1 (ppm)

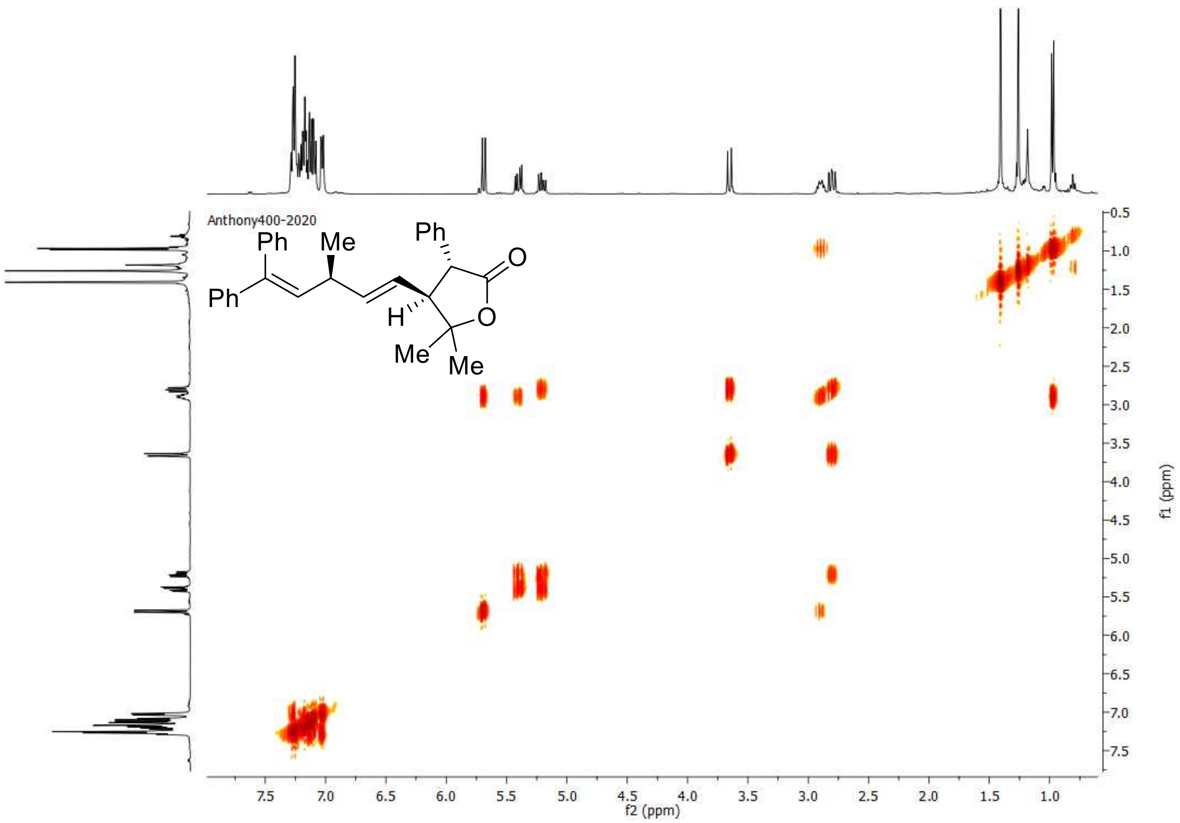
Anthony400-2020
Ph
Me
Ph
O
Ph
H
O
Me
Me
f2 (ppm)
f1 (ppm)

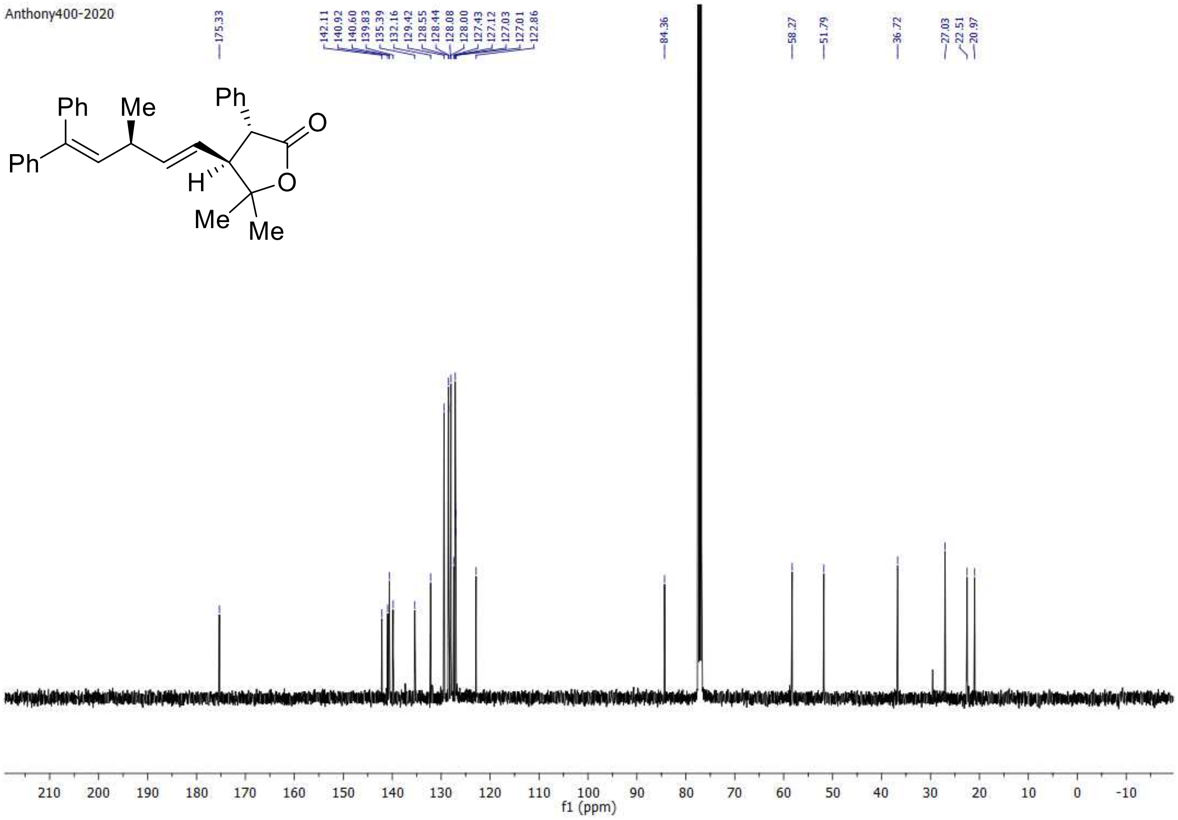
Anthony400-2020
175.33
142.11
140.92
140.60
139.83
135.39
132.16
129.42
128.55
128.44
128.08
128.00
127.43
127.12
127.03
127.01
122.86
84.36
58.27
51.79
36.72
27.03
22.51
20.97
Ph
Me
Ph
O
Ph
H
O
Me
Me
210 200 190 180 170 160 150 140 130 120 110 100 90 80 70 60 50 40 30 20 10 0 -10
f1 (ppm)

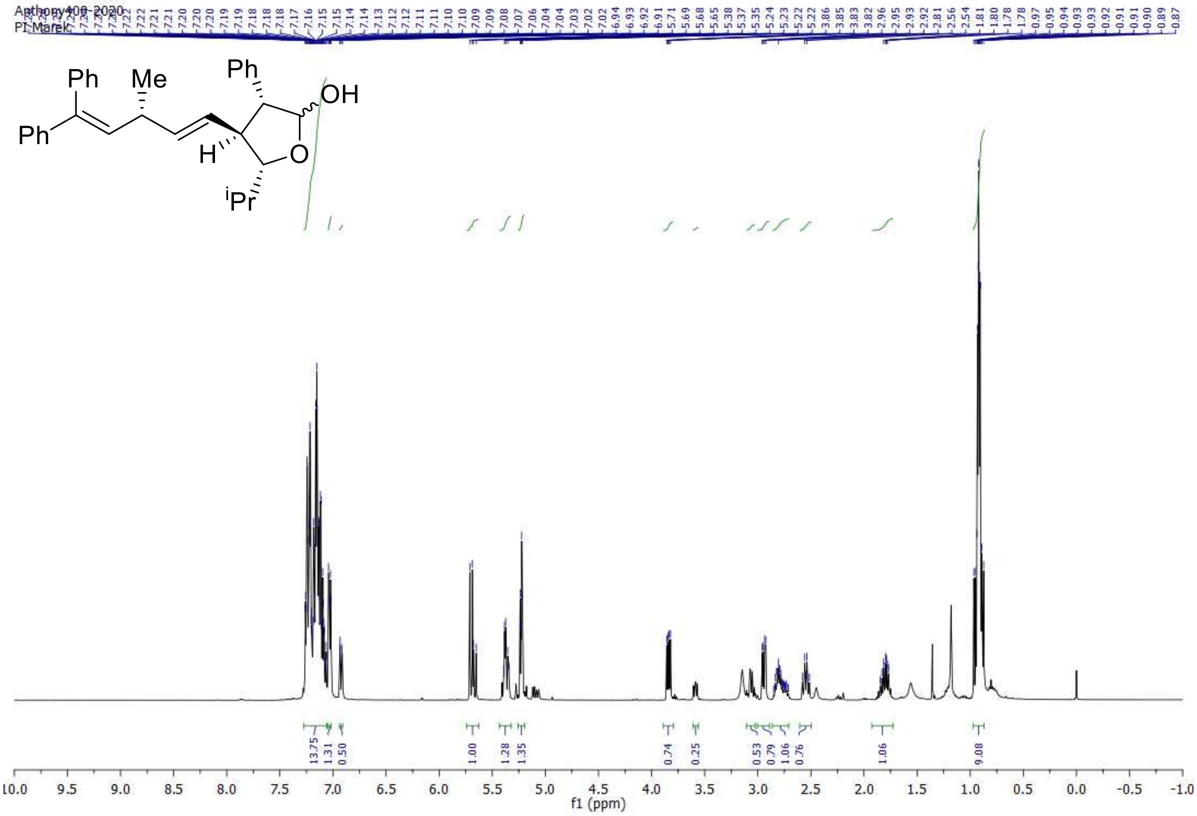
Anthony400-2020
PI Marek
Ph
Me
Ph
OH
Ph
H
O
iPr
13.75
1.31
0.50
1.00
1.28
1.35
0.74
0.25
0.53
0.79
1.06
0.76
1.06
9.08
f1 (ppm)

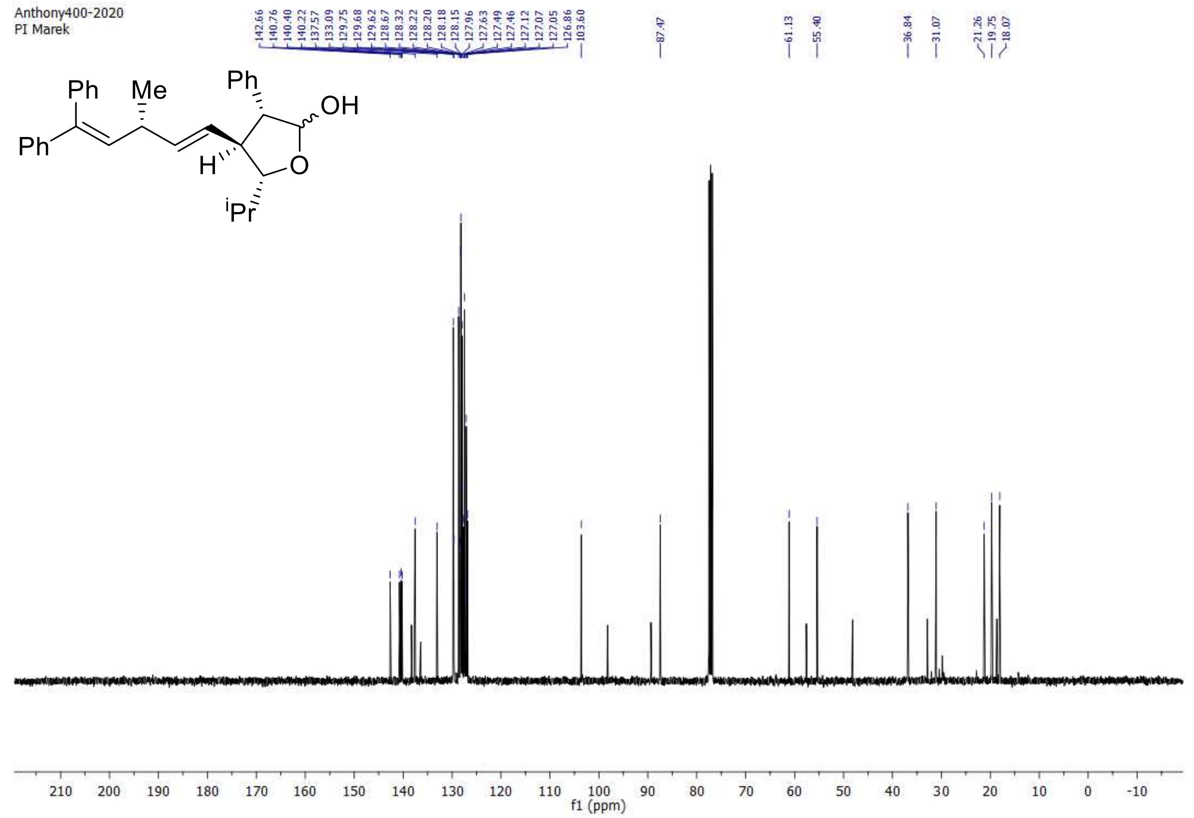
Anthony400-2020
PI Marek
142.66
140.76
140.40
140.22
137.57
133.09
129.75
129.68
129.62
128.67
128.32
128.22
128.20
128.18
128.15
127.96
127.63
127.49
127.46
127.12
127.07
127.05
126.86
103.60
87.47
61.13
55.40
36.84
31.07
21.26
19.75
18.07
Ph
Me
Ph
OH
Ph
H
O
iPr
f1 (ppm)

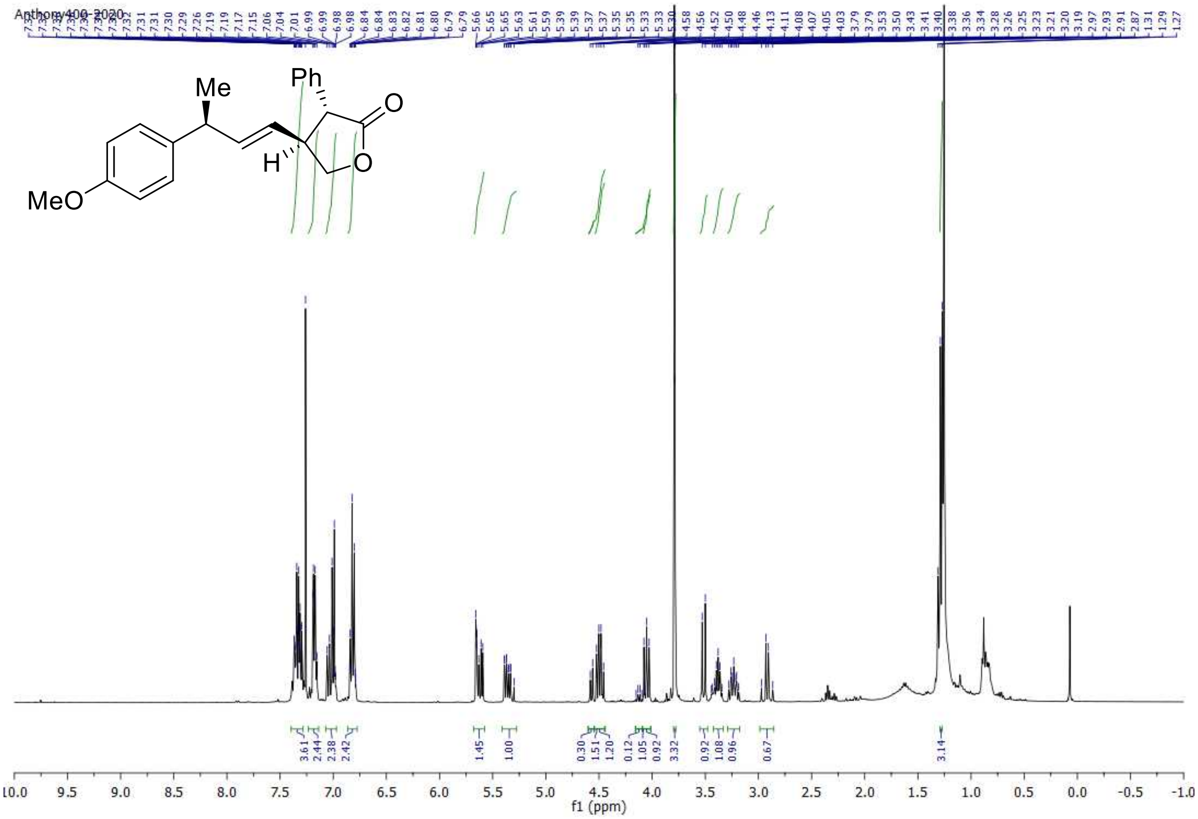

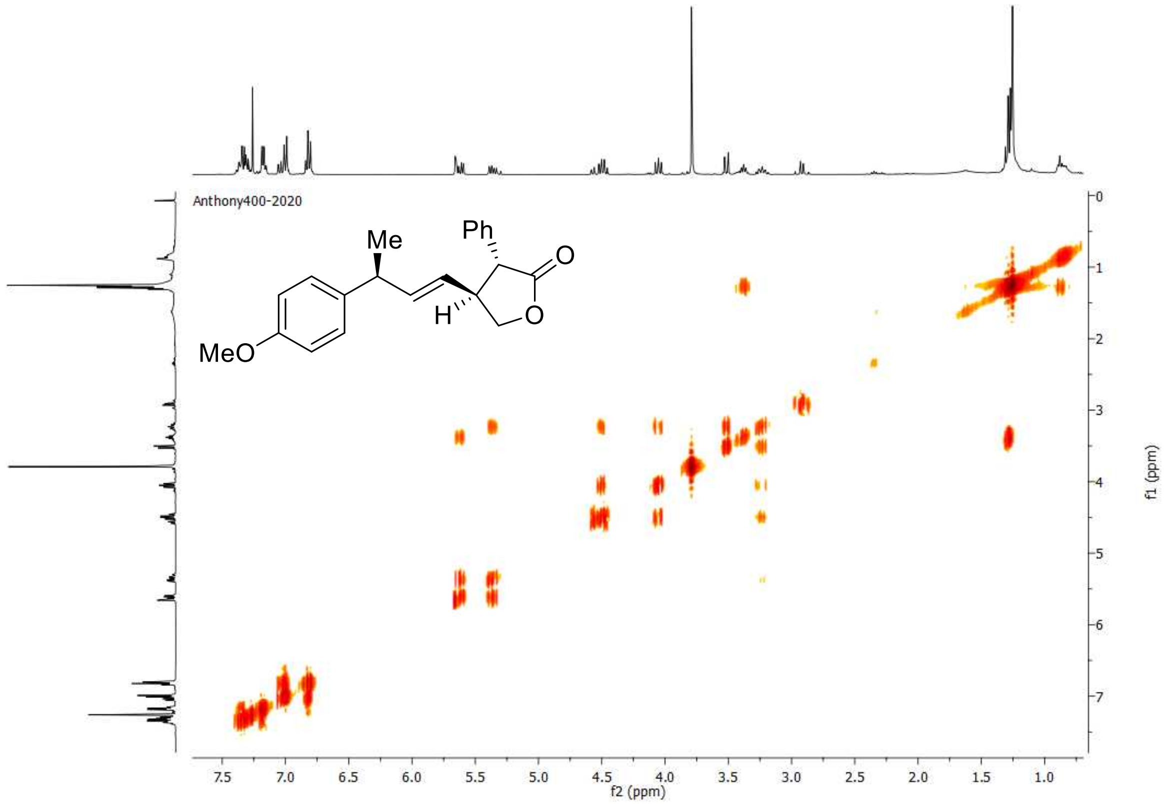

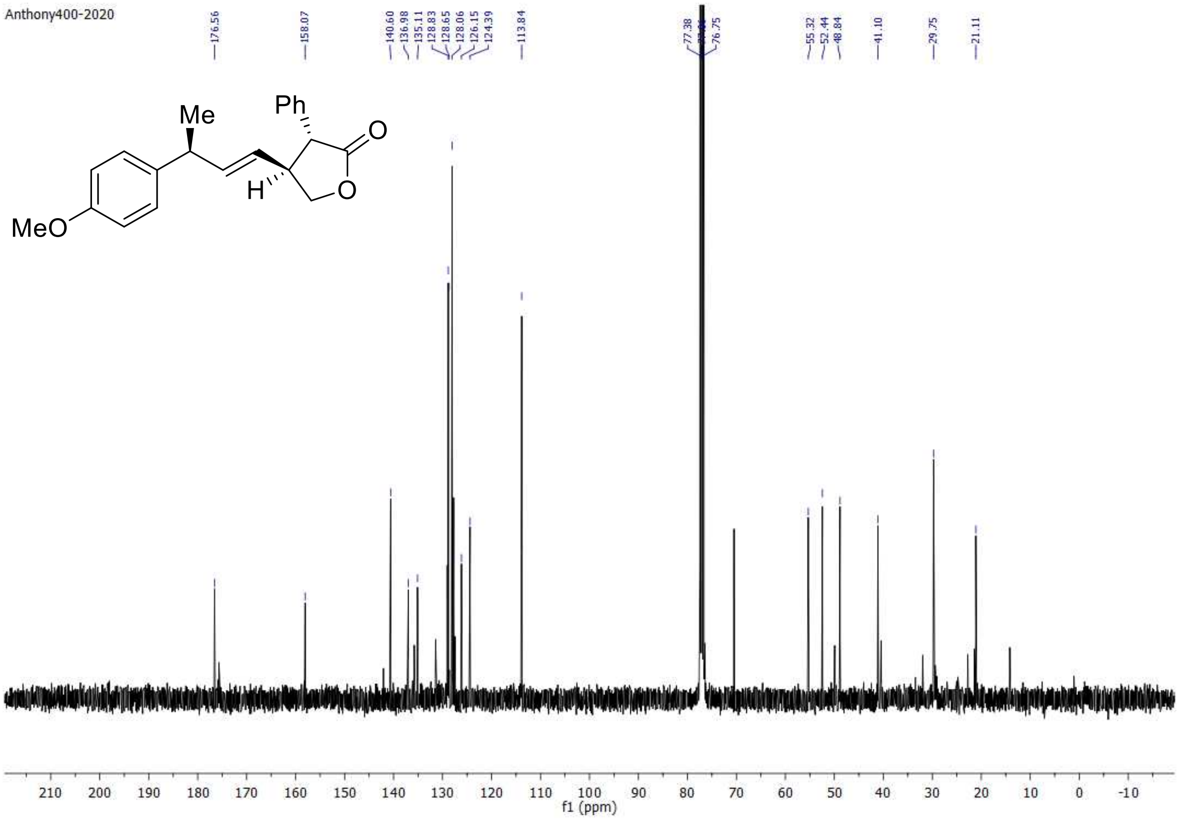
Anthony400-2020
176.56
158.07
140.60
136.98
135.11
128.83
128.65
128.06
126.15
124.39
113.84
77.38
76.75
55.32
52.44
48.84
41.10
29.75
21.11
Me
Ph
O
H
O
MeO
210 200 190 180 170 160 150 140 130 120 110 100 90 80 70 60 50 40 30 20 10 0 -10
f1 (ppm)

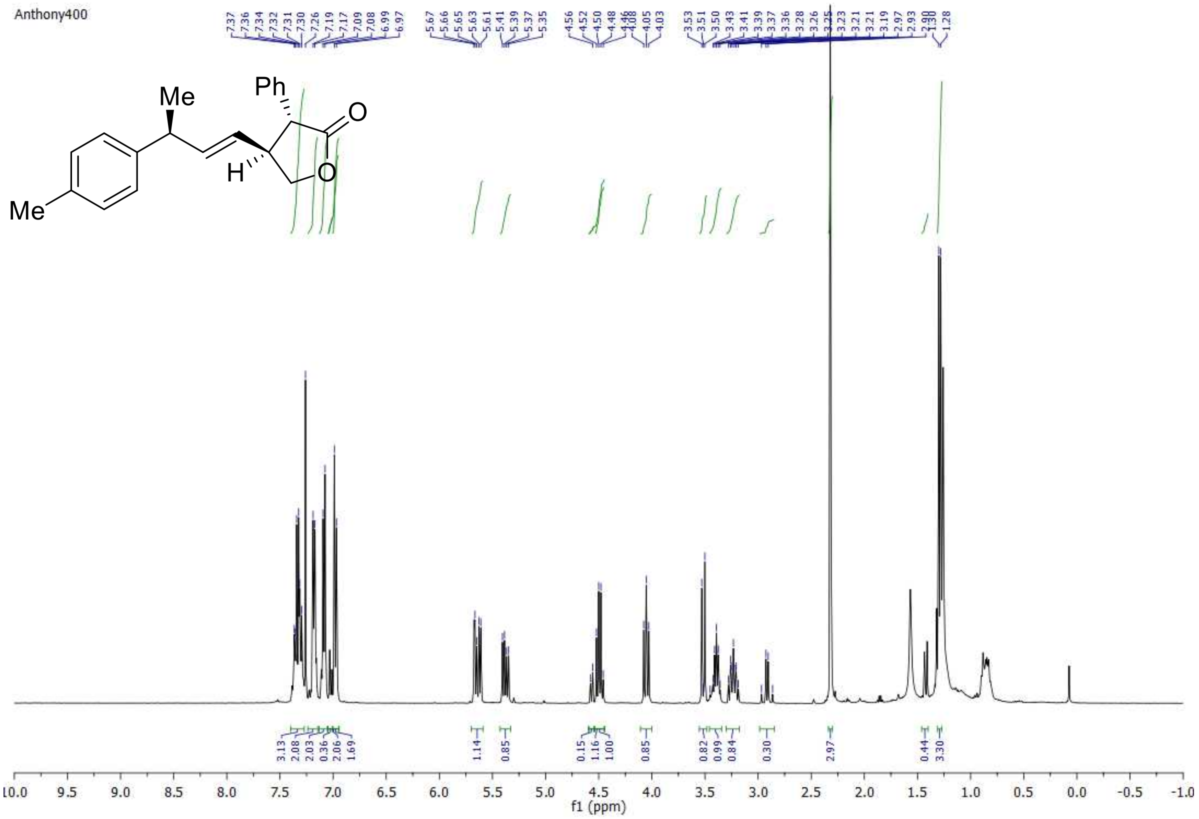
Anthony400
Me
Ph
O
H
O
Me
f1 (ppm)

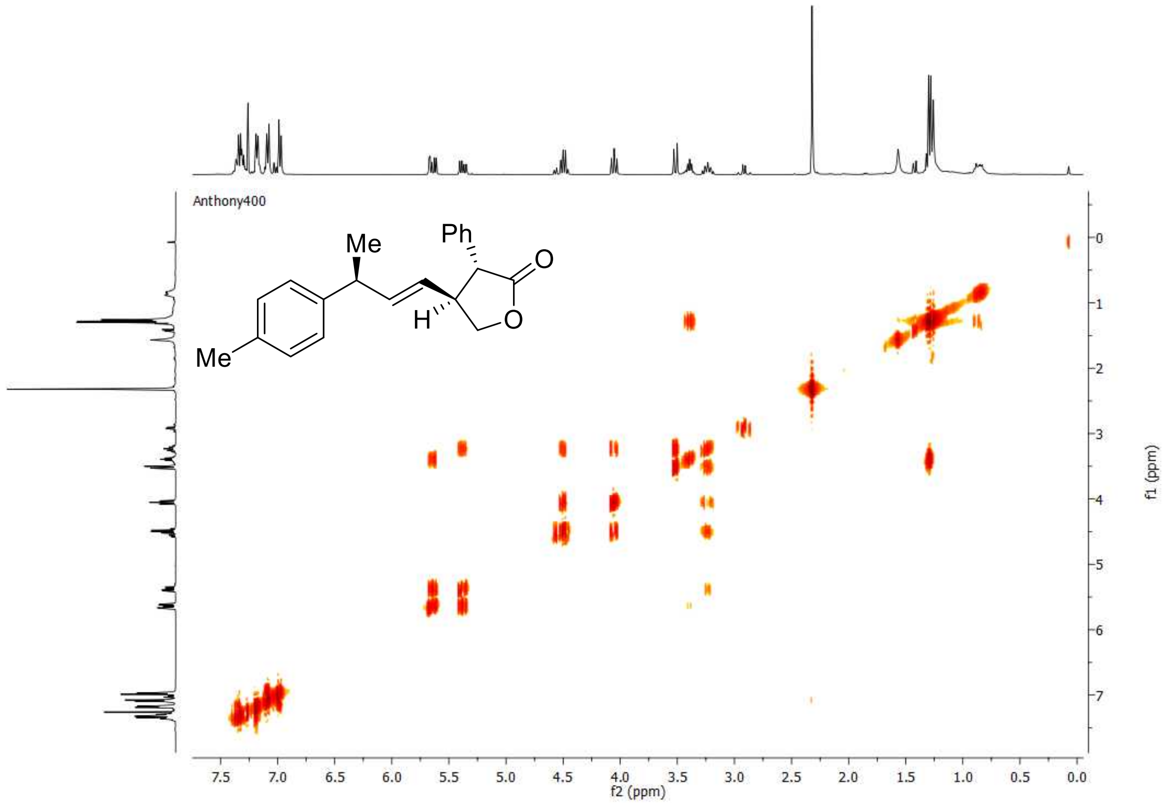
Anthony400
Me
Ph
O
H
O
Me
f2 (ppm)
f1 (ppm)

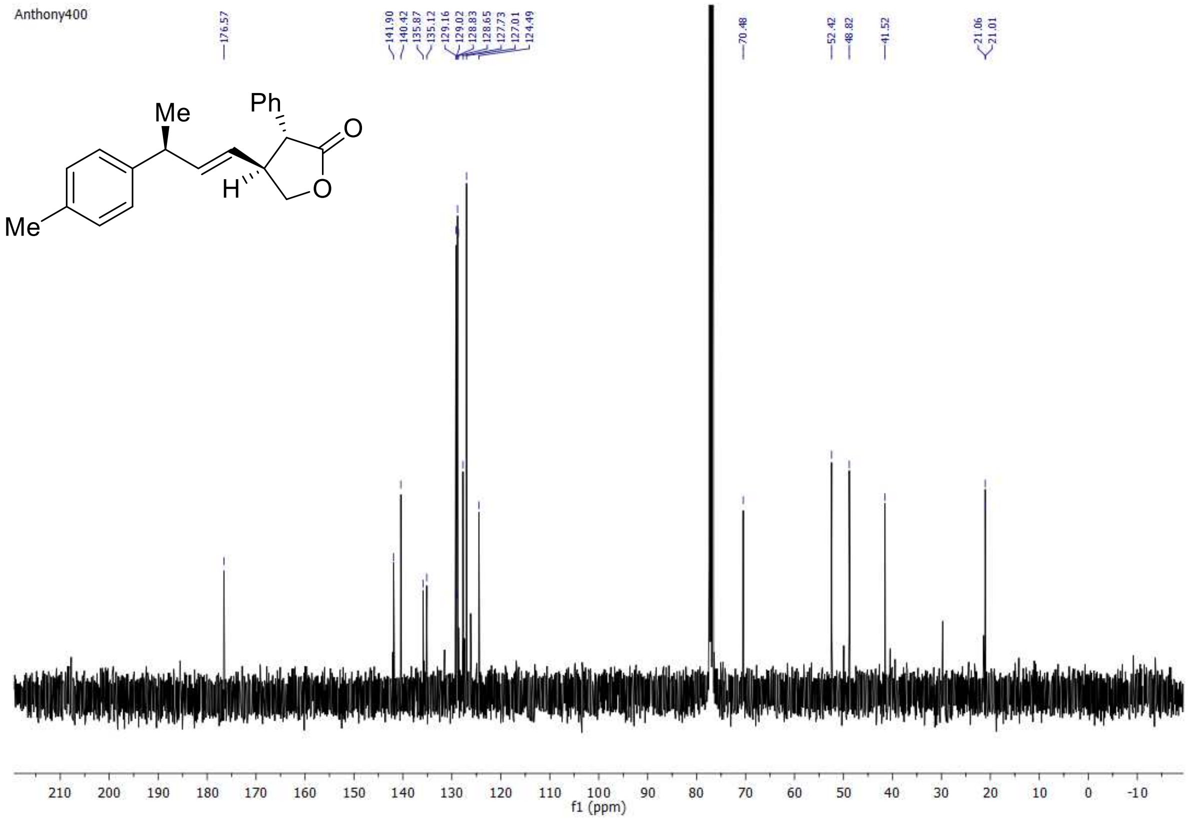
Anthony400
176.57
141.90
140.42
135.87
135.12
129.16
129.02
128.83
128.65
127.73
127.01
124.49
70.48
52.42
48.82
41.52
21.06
21.01
Me
Ph
O
H
O
Me
210 200 190 180 170 160 150 140 130 120 110 100 90 80 70 60 50 40 30 20 10 0 -10
f1 (ppm)

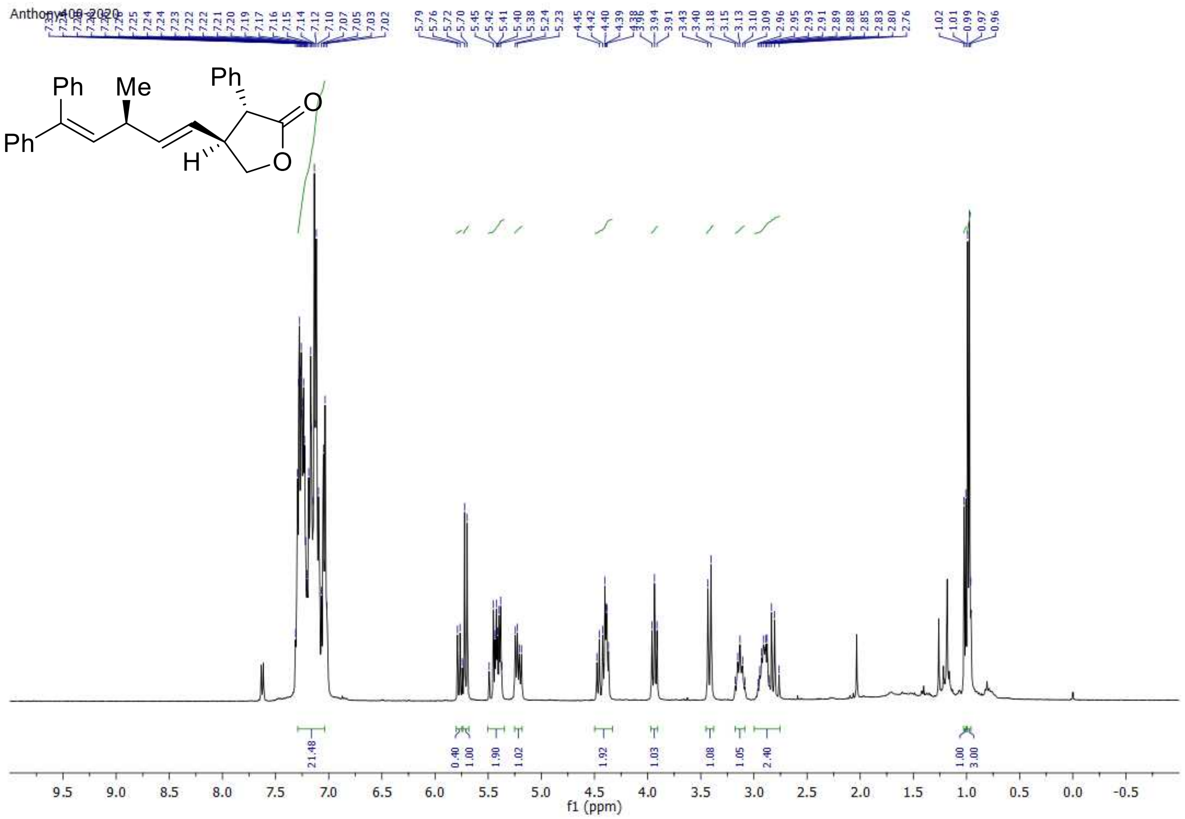
Anthony400-2020
Ph
Me
Ph
Ph
O
H
O
f1 (ppm)

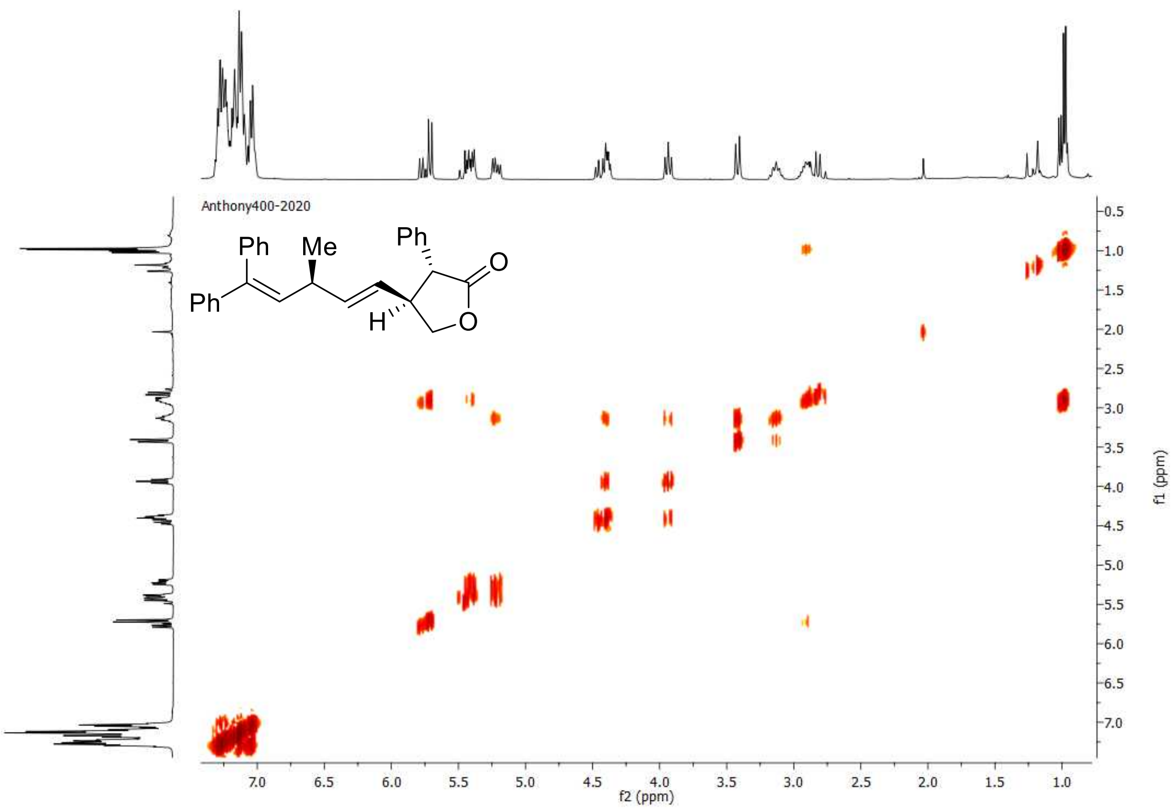
Anthony400-2020
Ph
Me
Ph
Ph
O
H
O
f2 (ppm)
f1 (ppm)

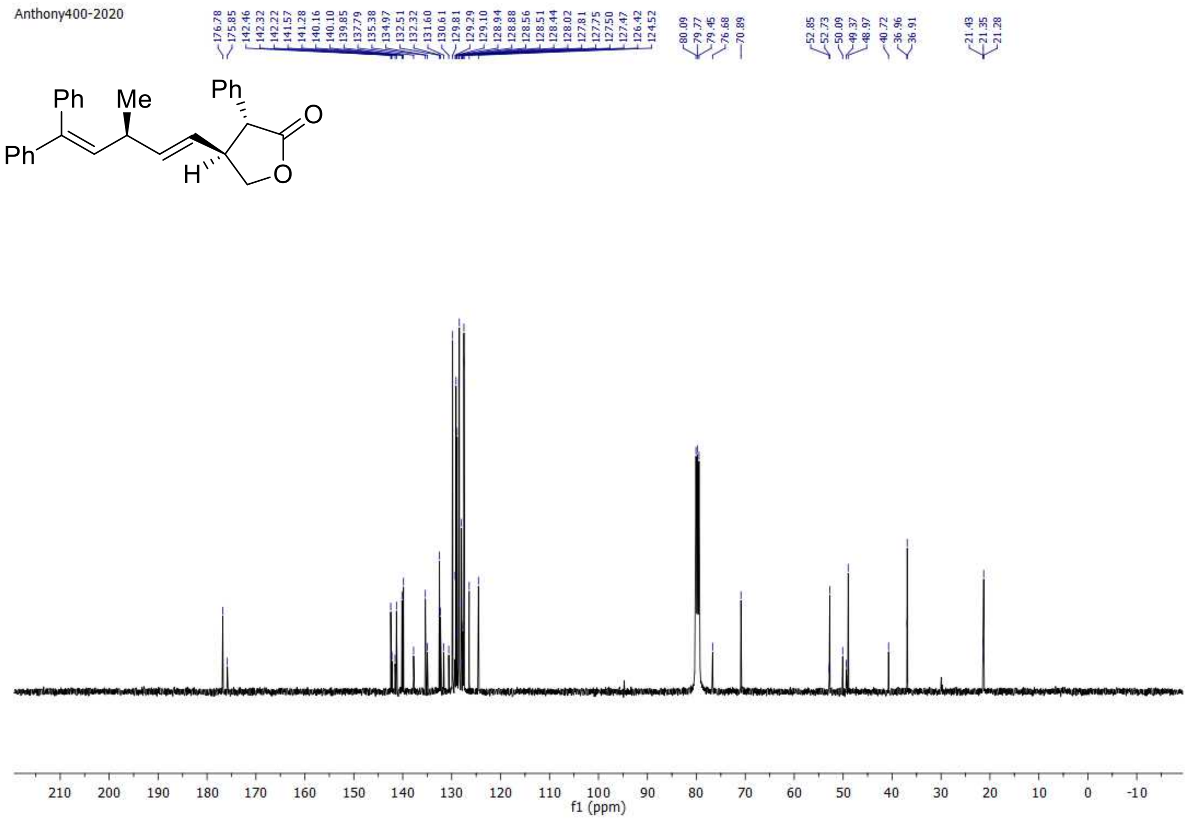
Anthony400-2020
f1 (ppm)

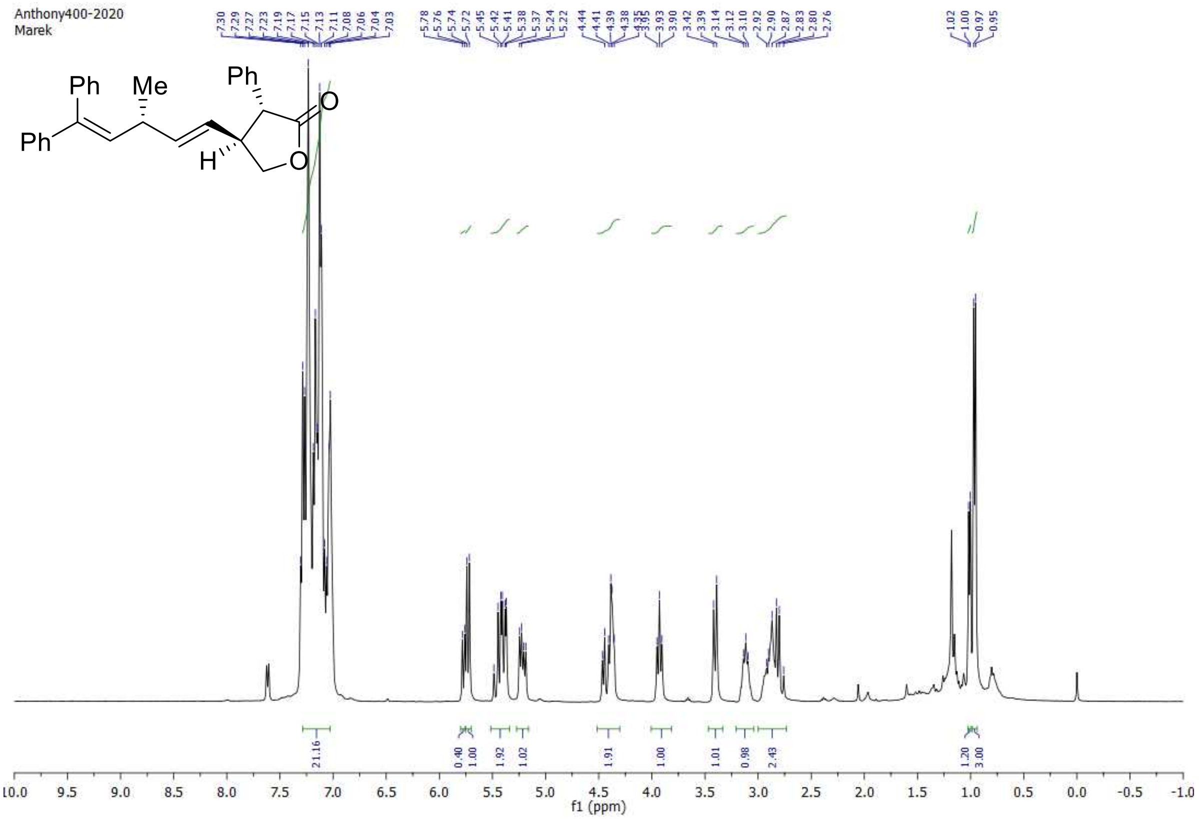
Anthony400-2020
Marek
Ph
Me
Ph
O
Ph
H
O
f1 (ppm)

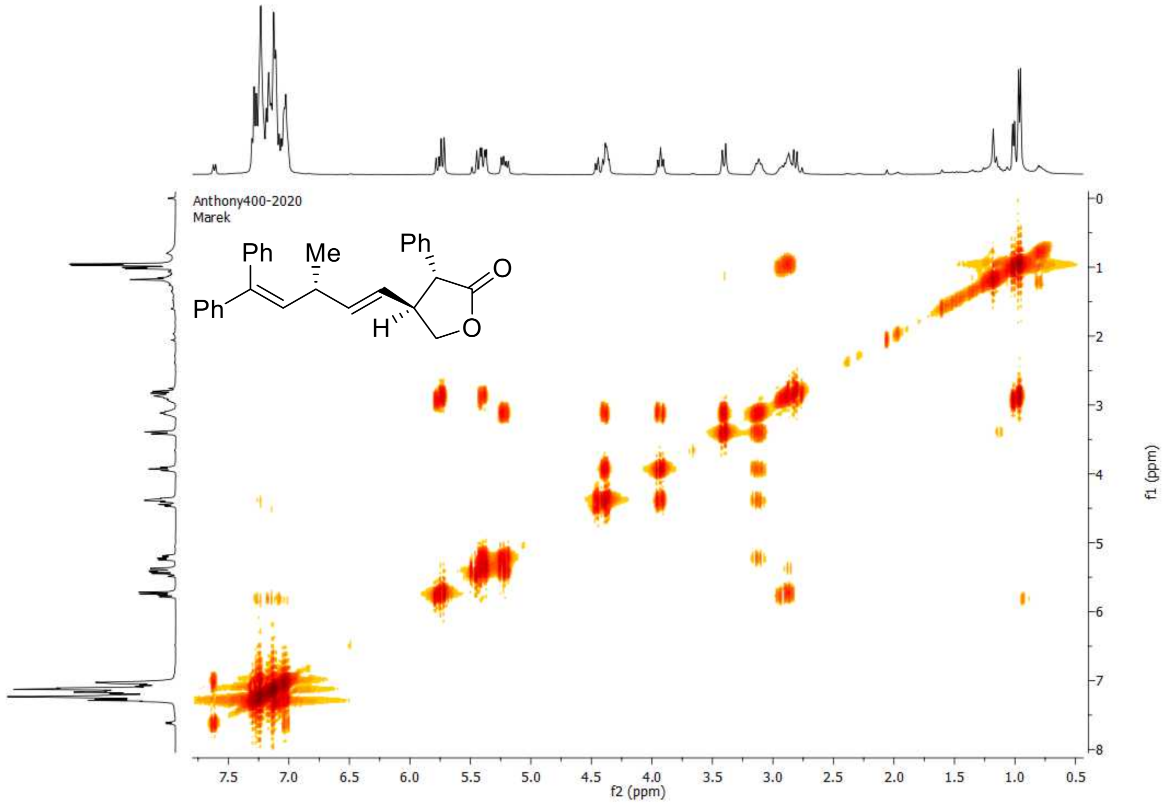
Anthony400-2020
Marek
Ph
Me
Ph
O
Ph
H
O
f2 (ppm)
f1 (ppm)

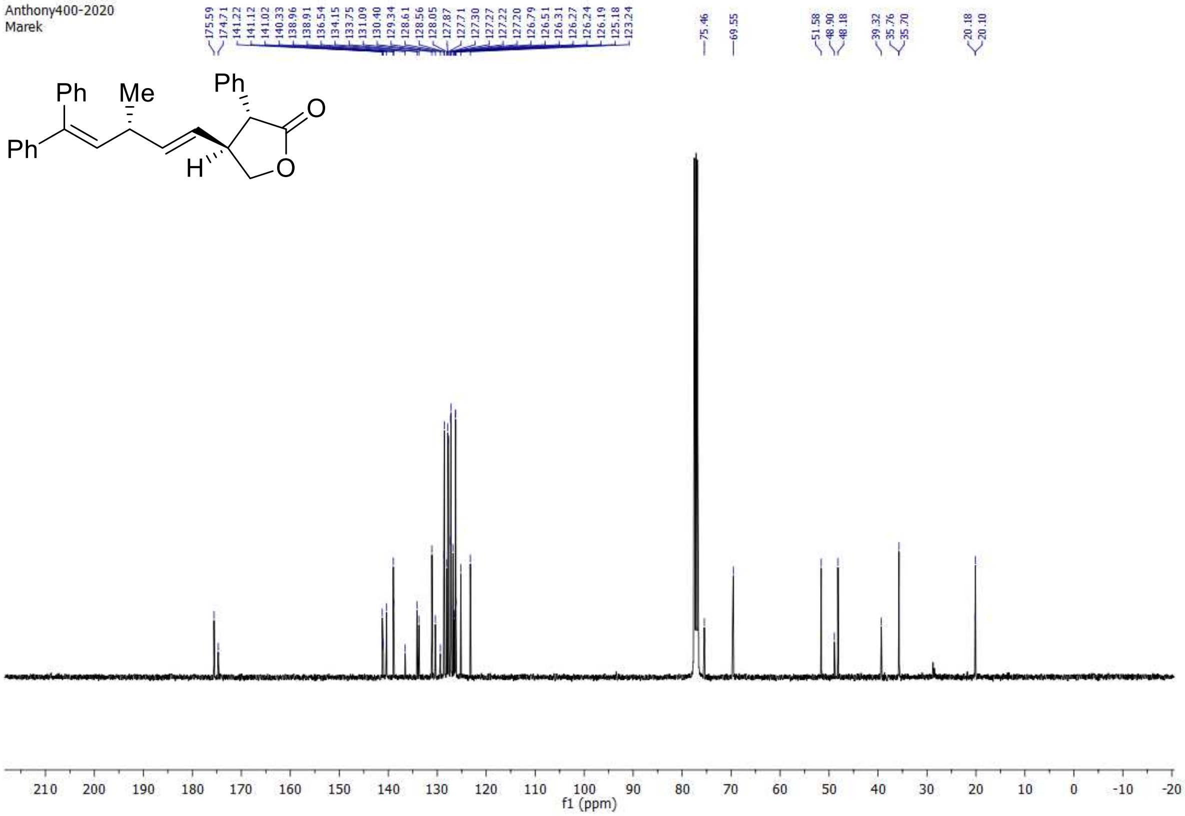

Anthony400-2020
Marek
f1 (ppm)

Overlay comparison of 1HNMR of 10o and 10p

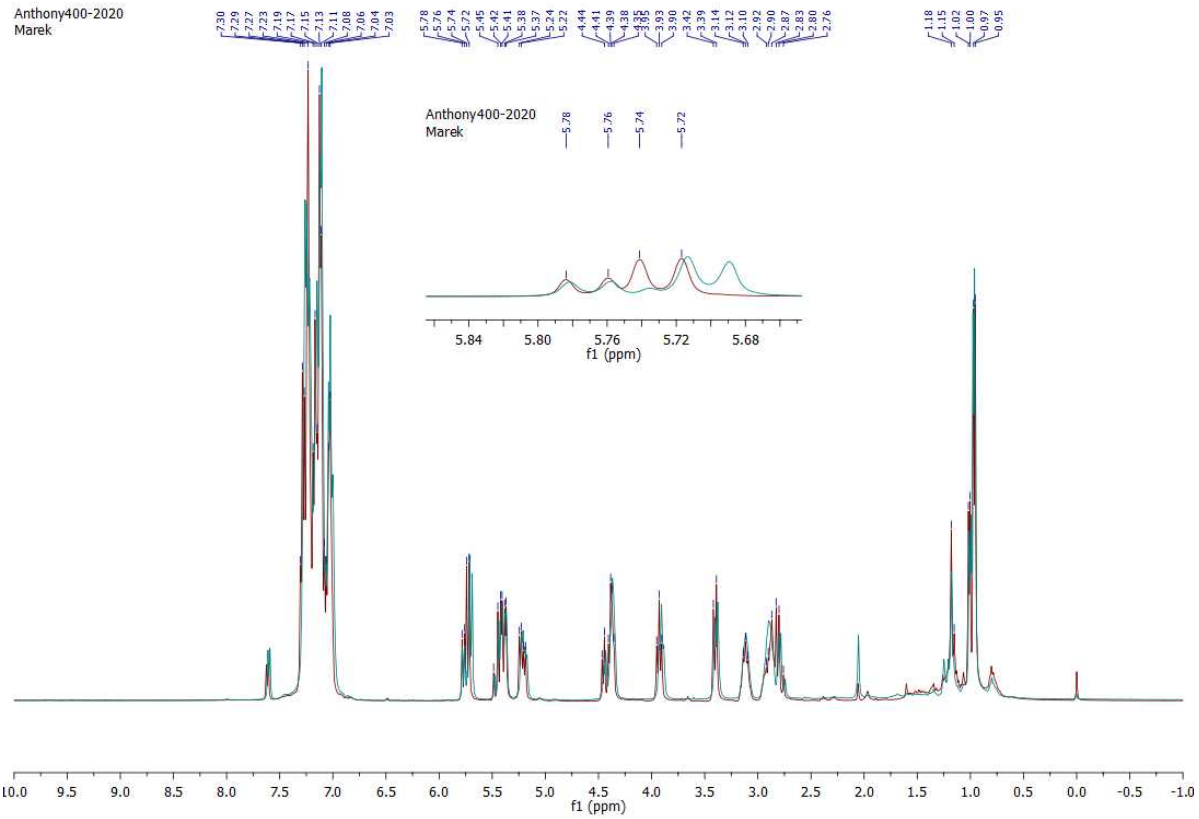

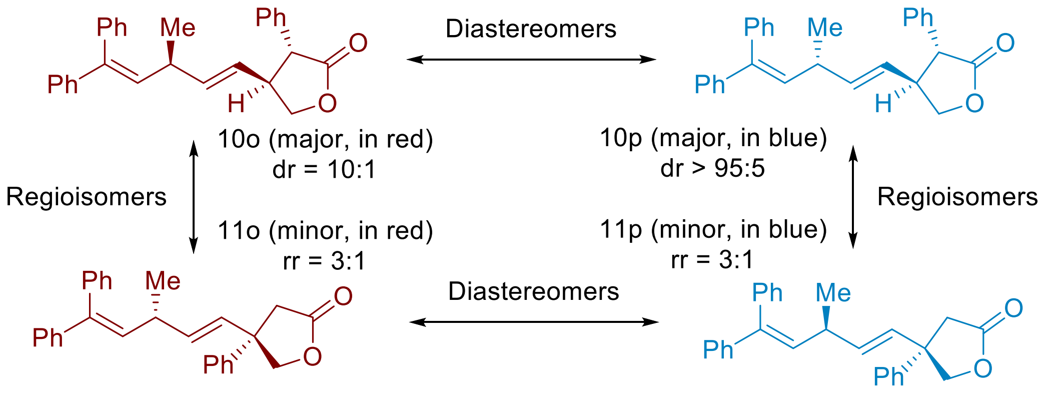

Overlay comparison of ^{13}CNMR of 10o and 10p

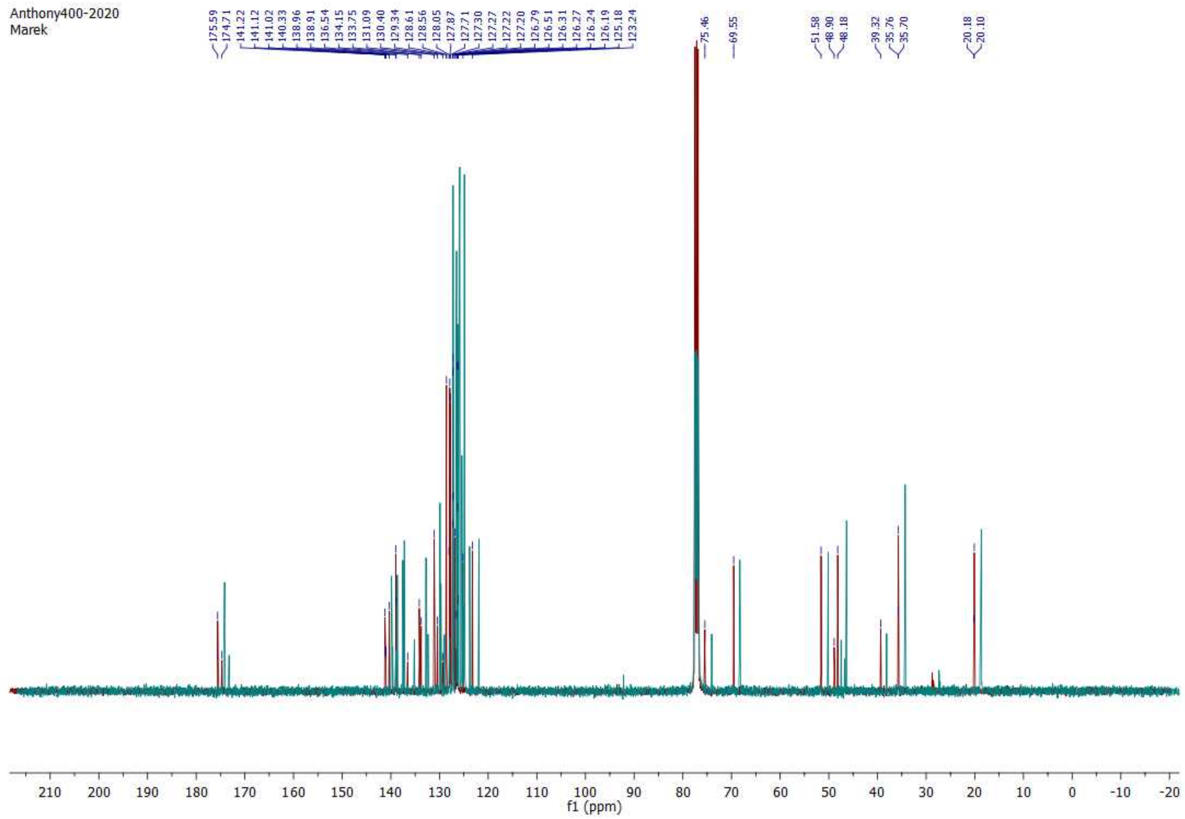

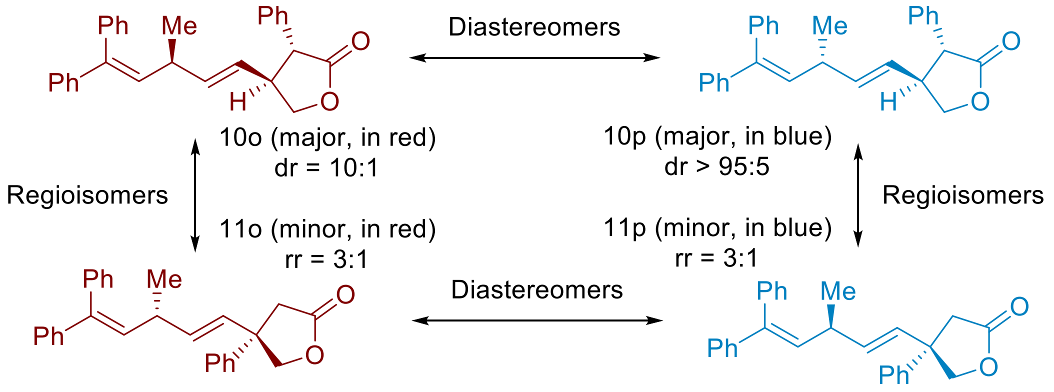

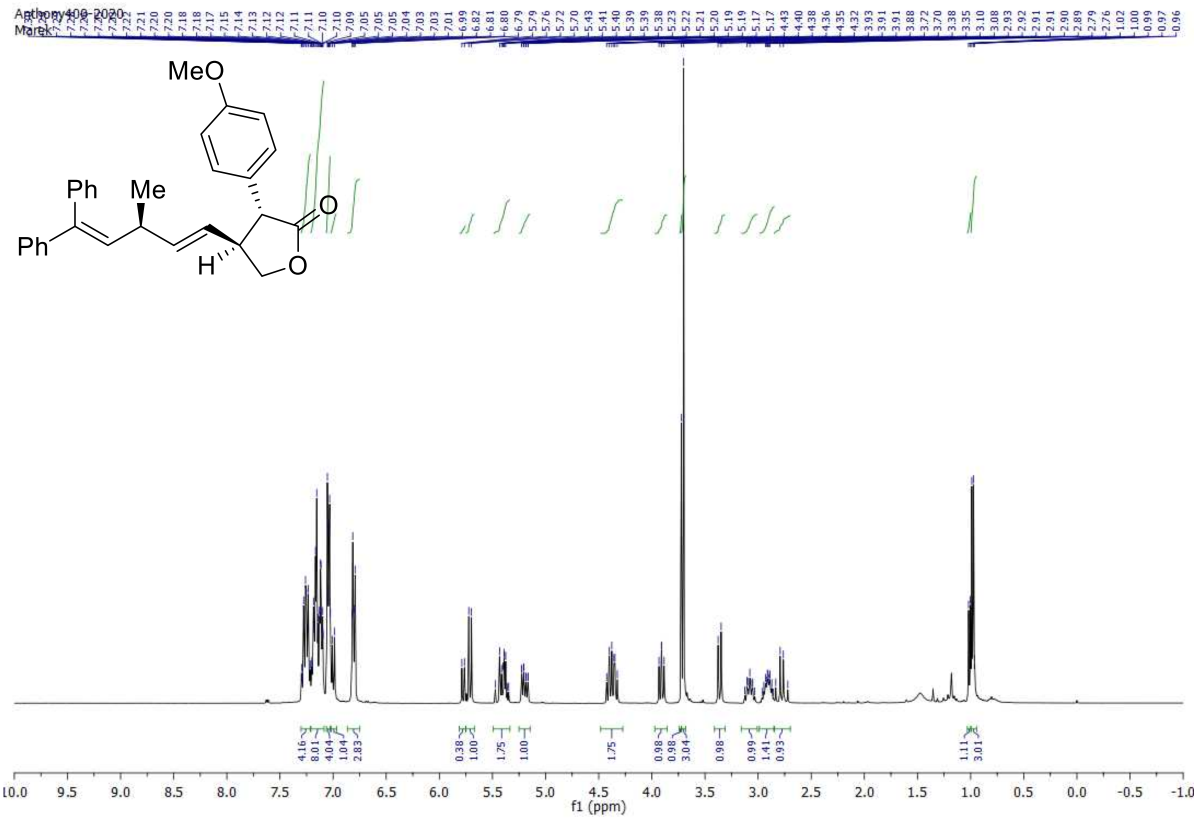

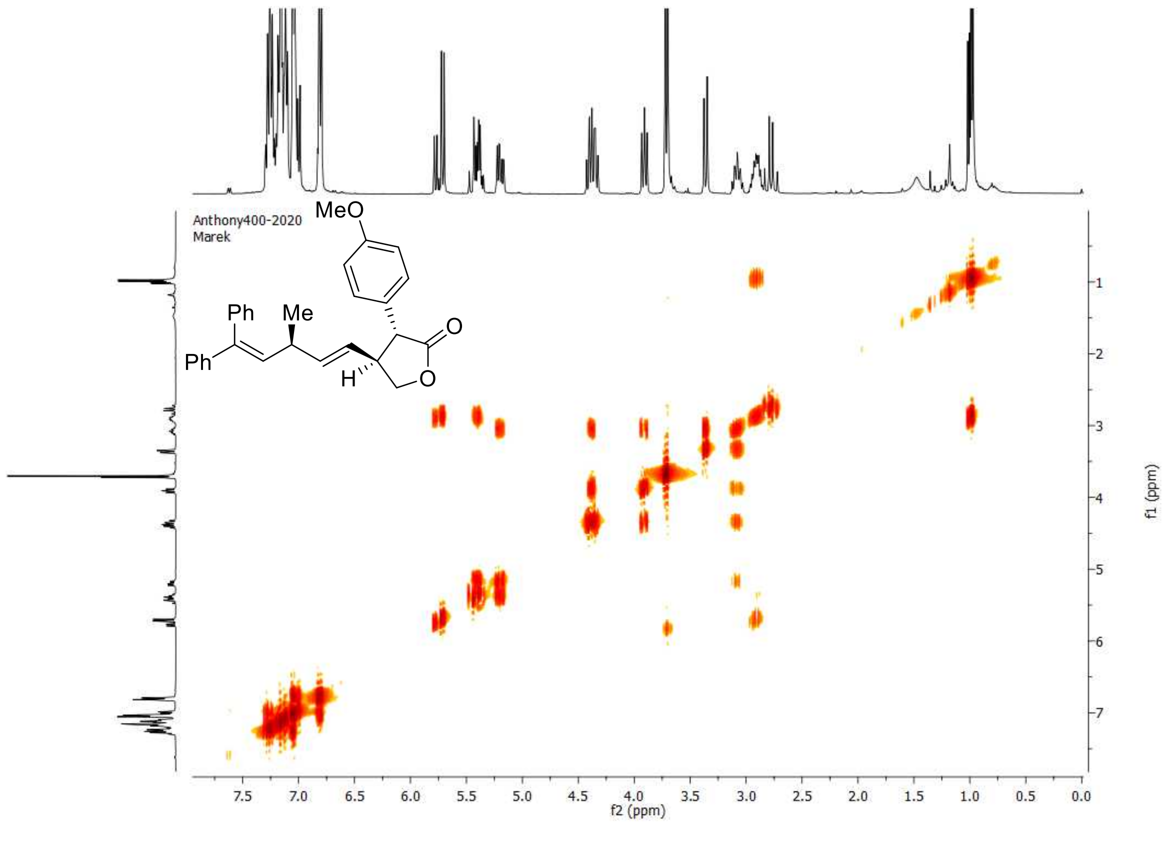

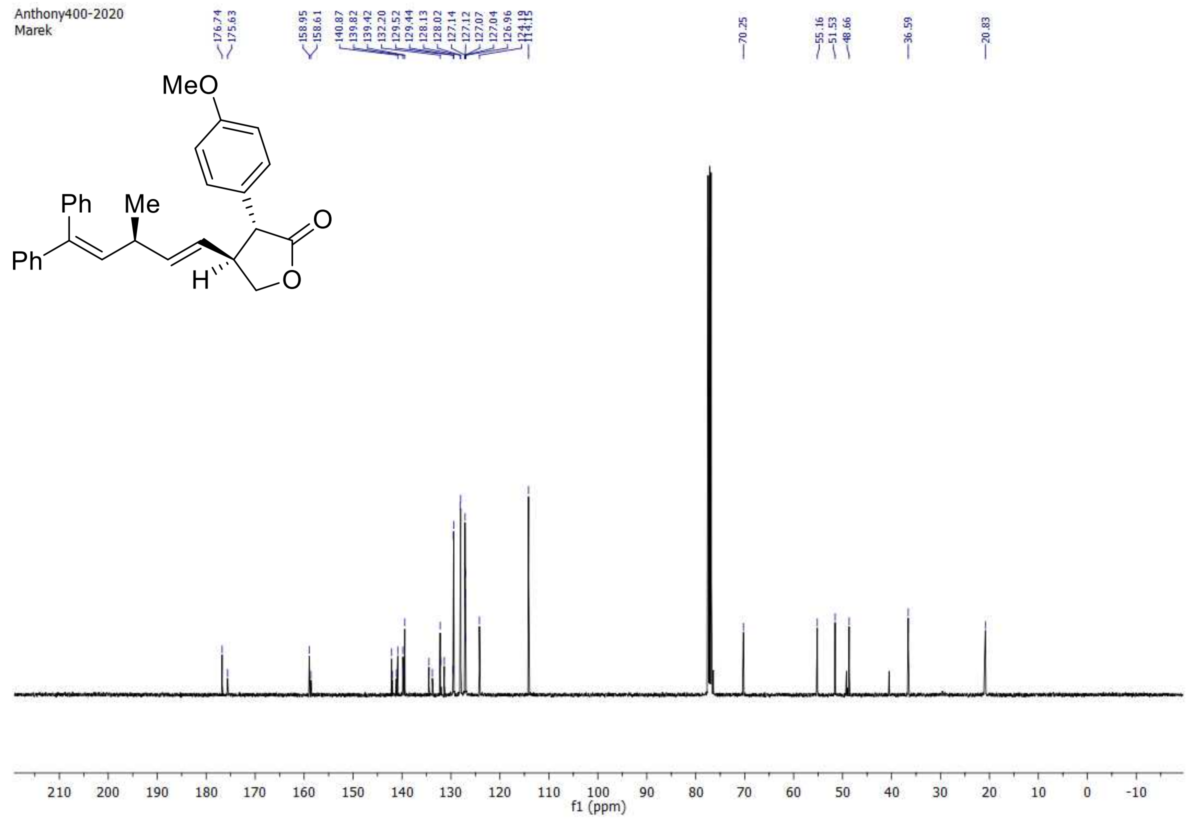
Anthony400-2020
Marek
176.74
175.63
158.95
158.61
140.87
139.82
139.42
132.20
129.52
129.44
128.13
128.02
127.14
127.12
127.07
127.04
126.96
70.25
55.16
51.53
48.66
36.59
20.83
MeO
Ph
Me
Ph
H
O
O
f1 (ppm)

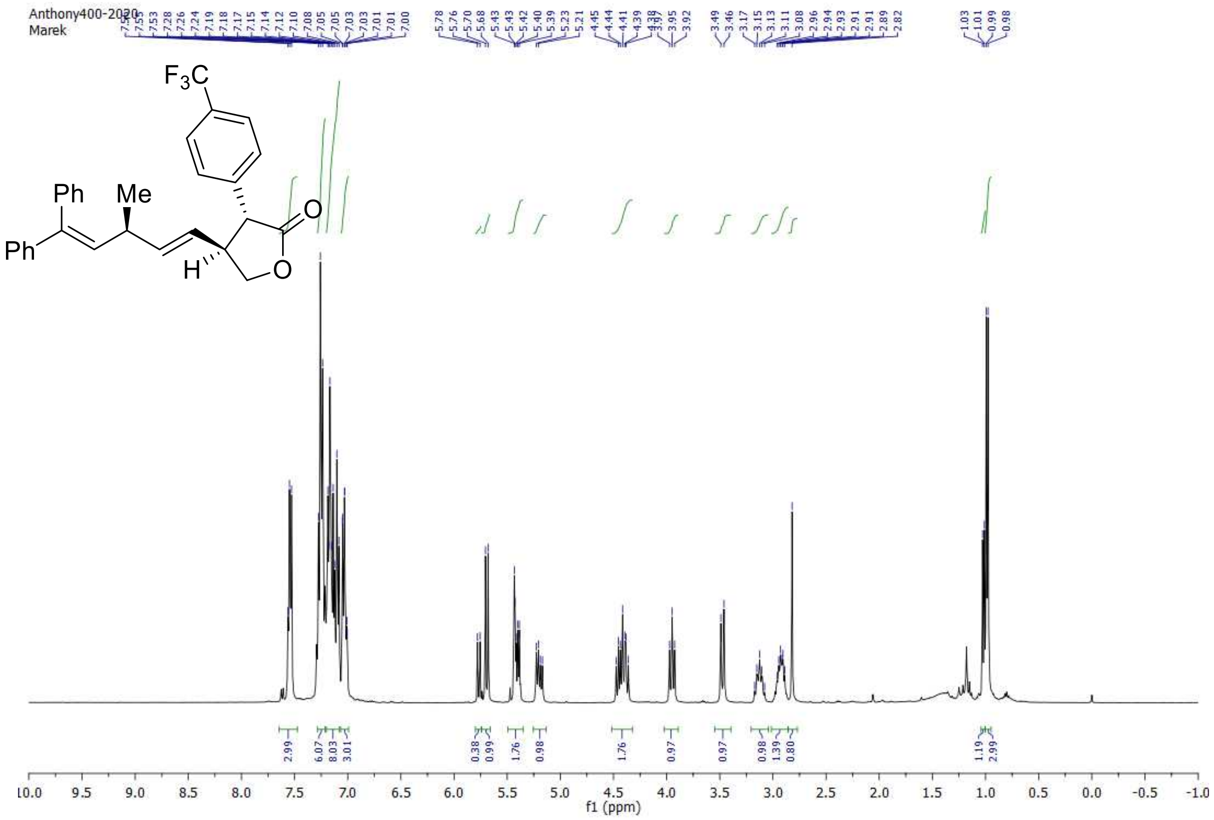

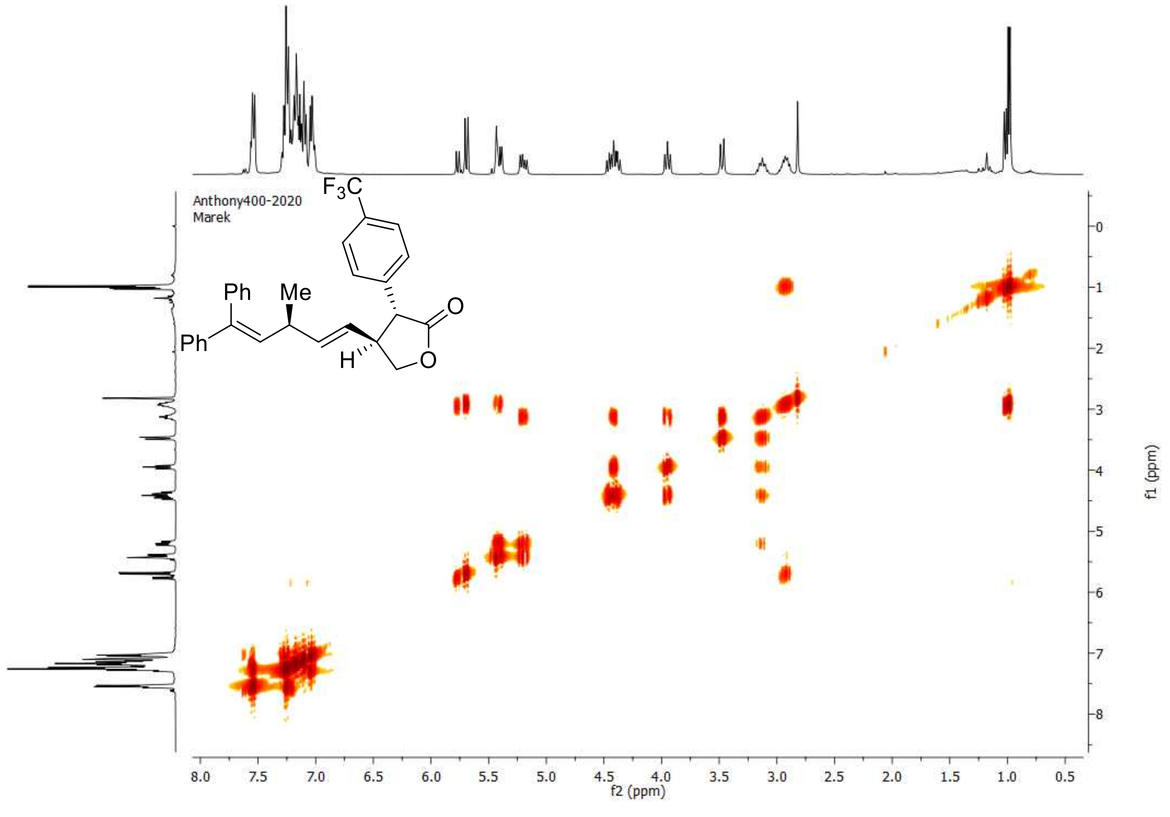

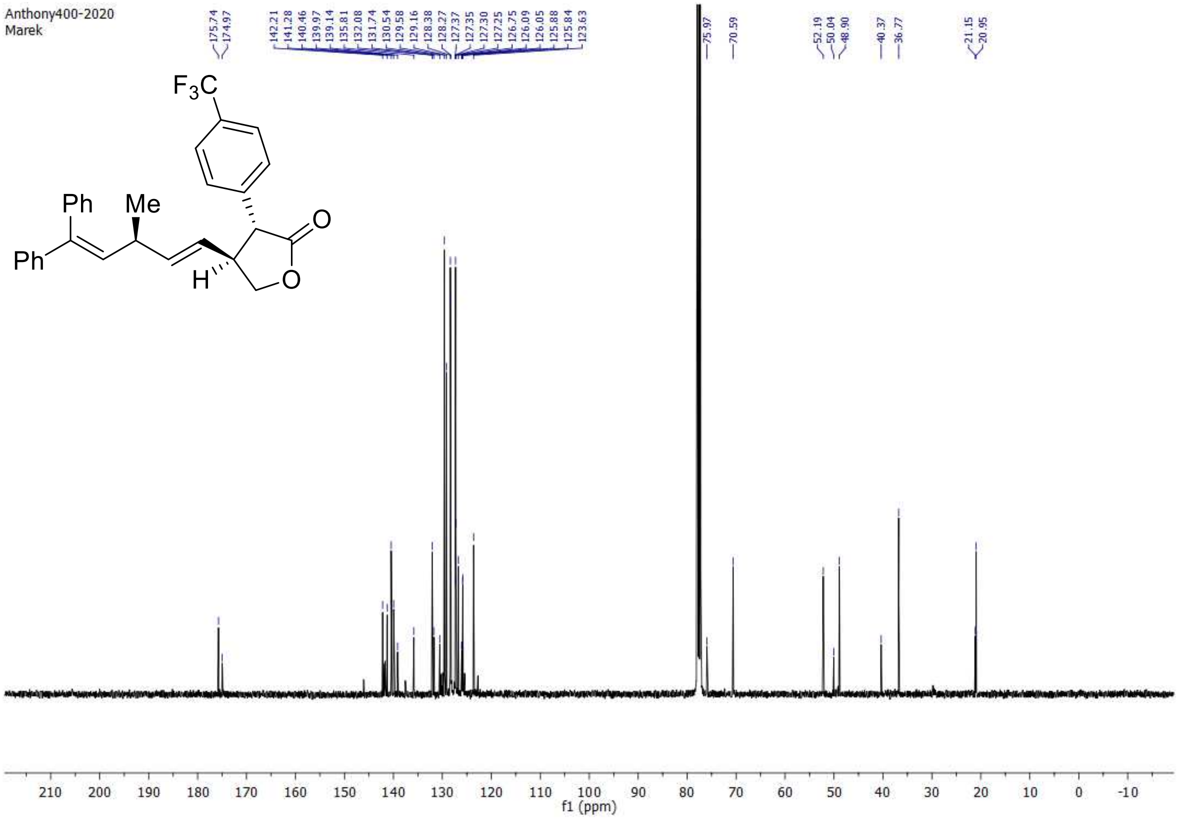

Anthony400-2020
Marek
175.74
174.97
142.21
141.28
140.46
139.97
139.14
135.81
132.08
131.74
130.54
129.58
129.16
128.38
128.27
127.37
127.35
127.30
127.25
126.75
126.09
126.05
125.88
125.84
123.63
75.97
70.59
52.19
50.04
48.90
40.37
36.77
21.15
20.95
F3C
Ph
Me
O
Ph
H
O
210
200
190
180
170
160
150
140
130
120
110
100
90
80
70
60
50
40
30
20
10
0
-10
f1 (ppm)

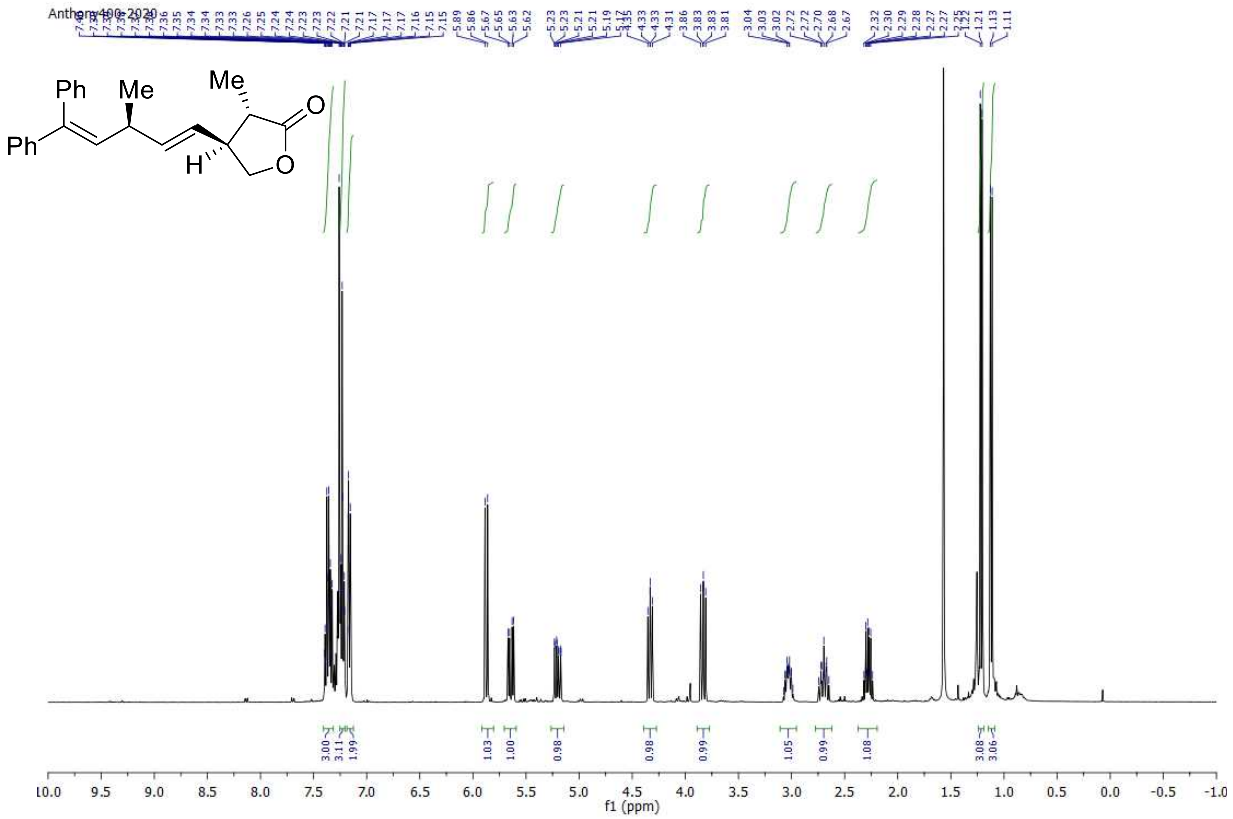
Anthony400-2020
Ph
Me
Me
O
Ph
H
O
f1 (ppm)

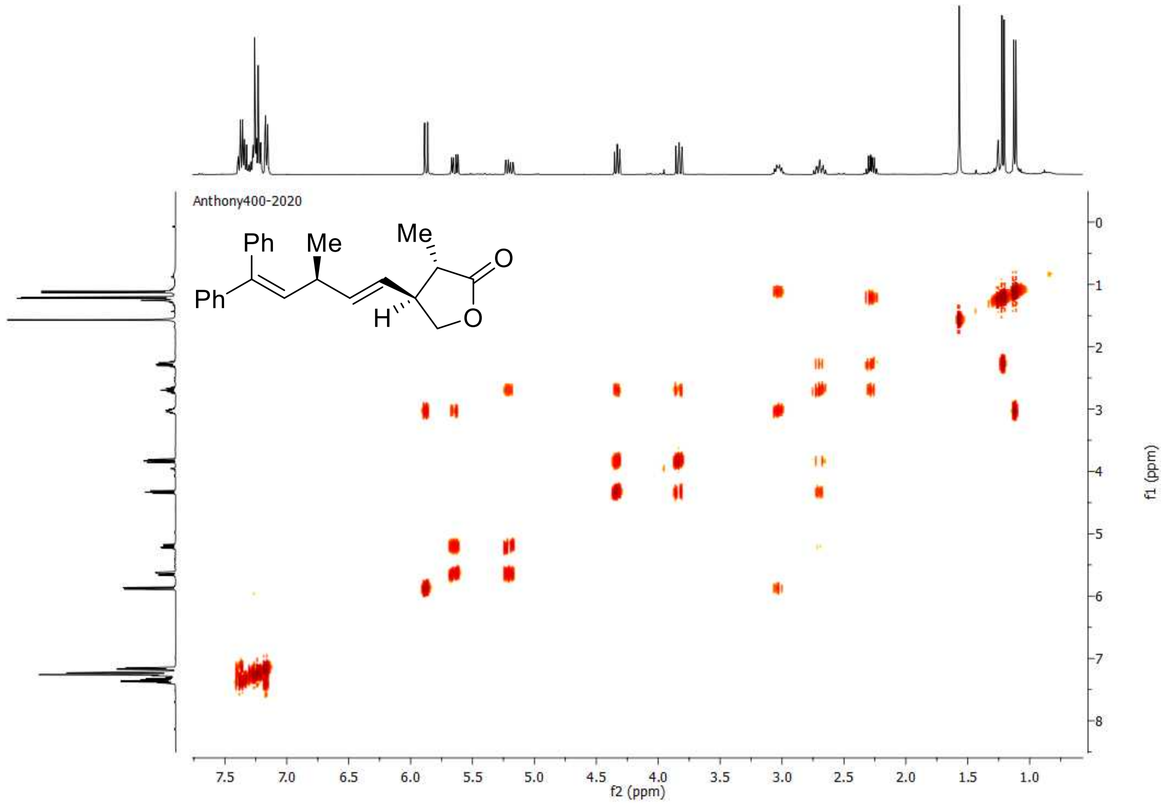
Anthony400-2020
Ph
Me
Me
O
Ph
H
O
f2 (ppm)
f1 (ppm)

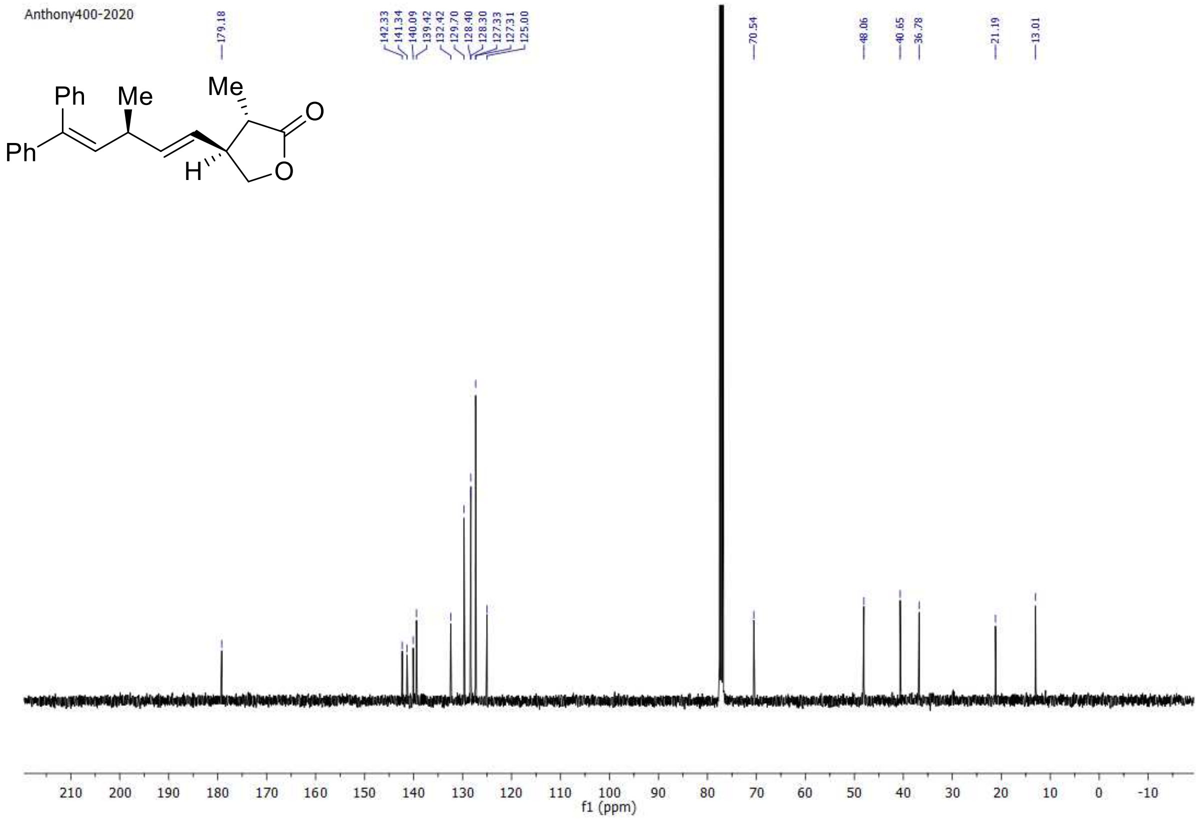

Anthony400-2020
179.18
142.33
141.34
140.09
139.42
132.42
129.70
128.40
128.30
127.33
127.31
125.00
70.54
48.06
40.65
36.78
21.19
13.01
Ph
Me
Me
O
Ph
H
O
210 200 190 180 170 160 150 140 130 120 110 100 90 80 70 60 50 40 30 20 10 0 -10
f1 (ppm)

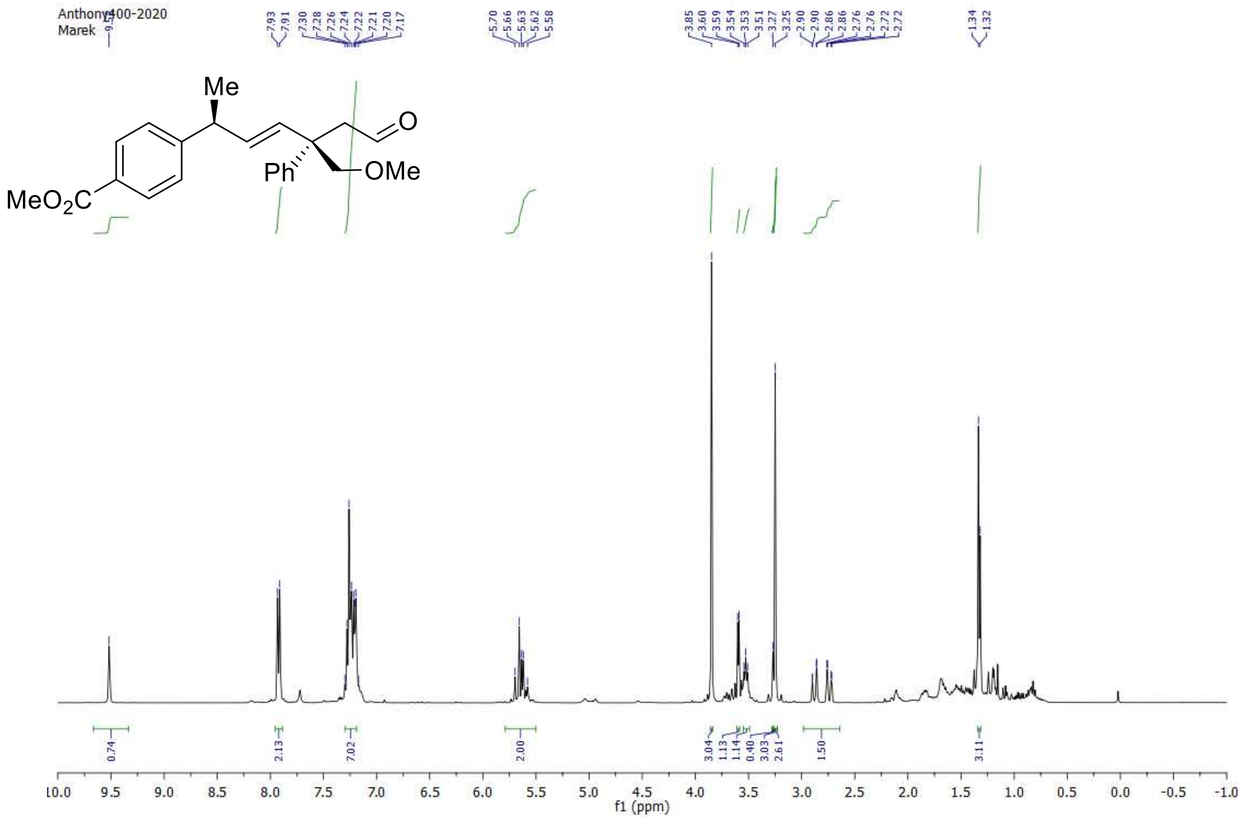
Anthony400-2020
Marek
Me
O
Ph
OMe
MeO_2C
f1 (ppm)

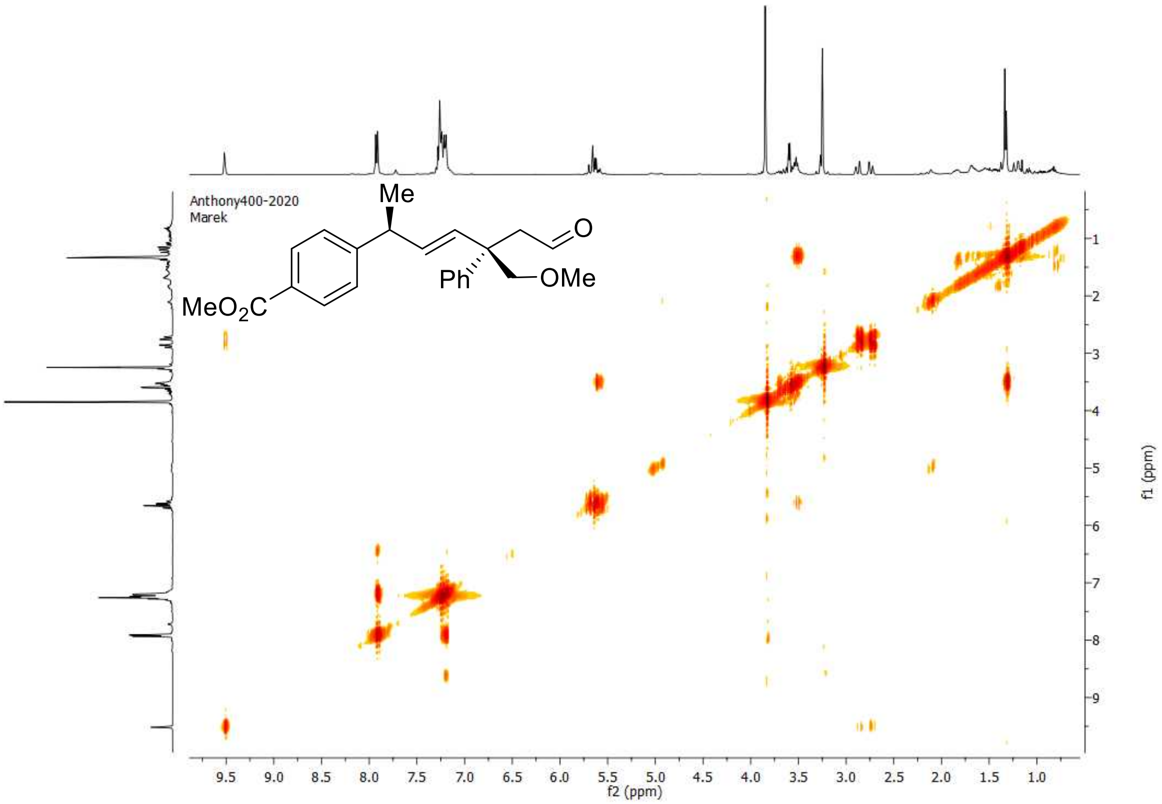
Anthony400-2020
Marek
Me
O
Ph
OMe
MeO_2C
f2 (ppm)
f1 (ppm)

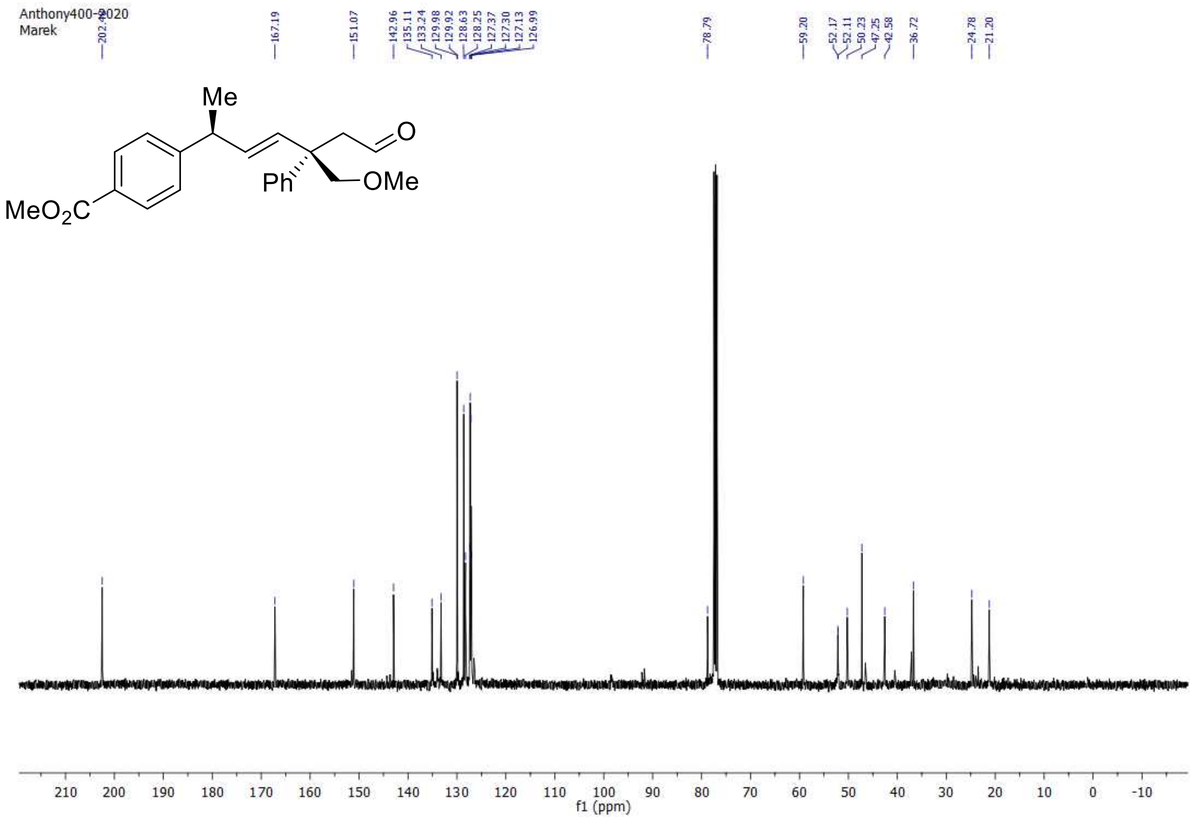
Anthony400-2020
Marek
202.40
167.19
151.07
142.96
135.11
133.24
129.98
129.92
128.63
128.25
127.37
127.30
127.13
126.99
78.79
59.20
52.17
52.11
50.23
47.25
42.58
36.72
24.78
21.20
Me
O
Ph
OMe
MeO2C
f1 (ppm)

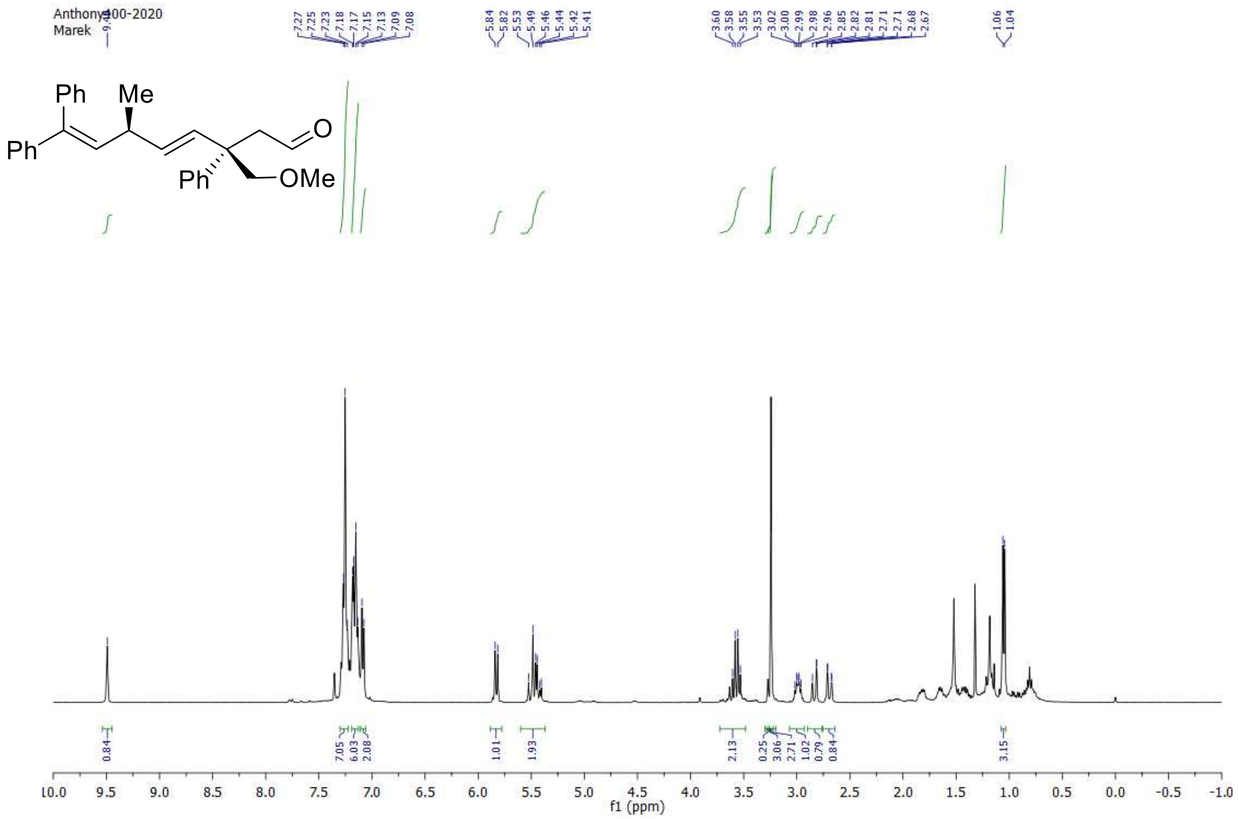

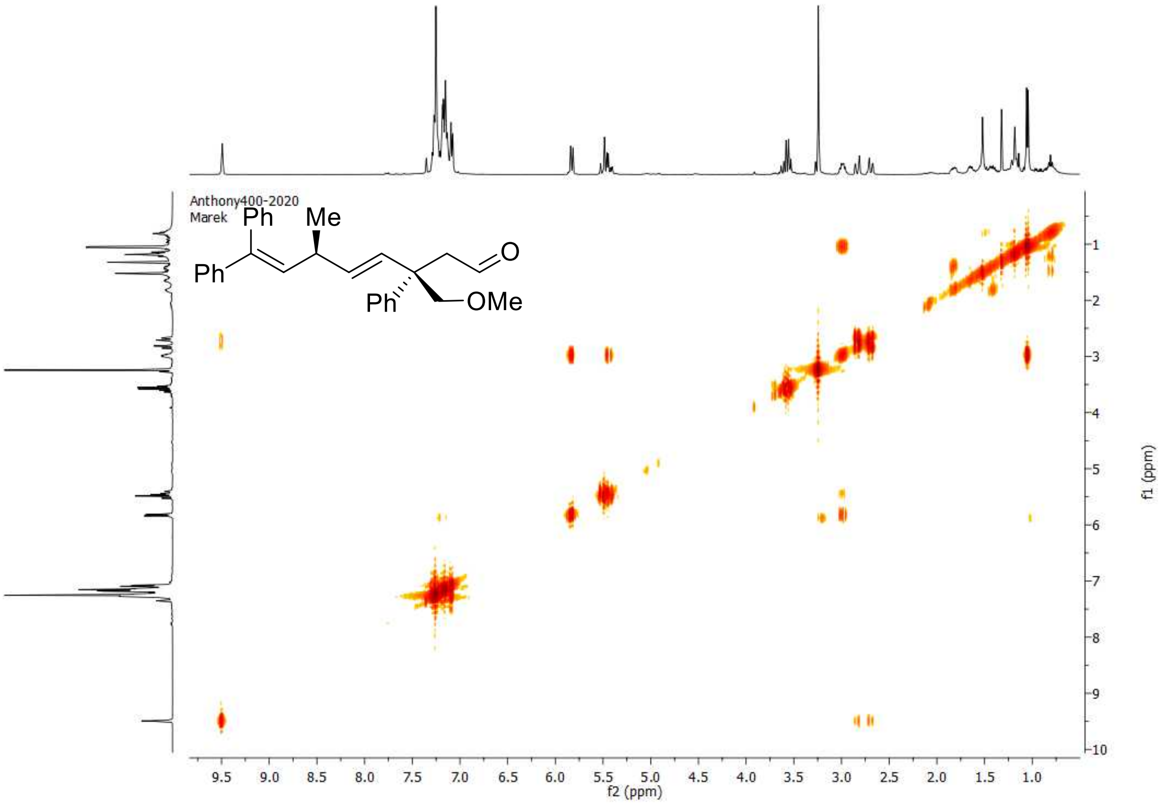

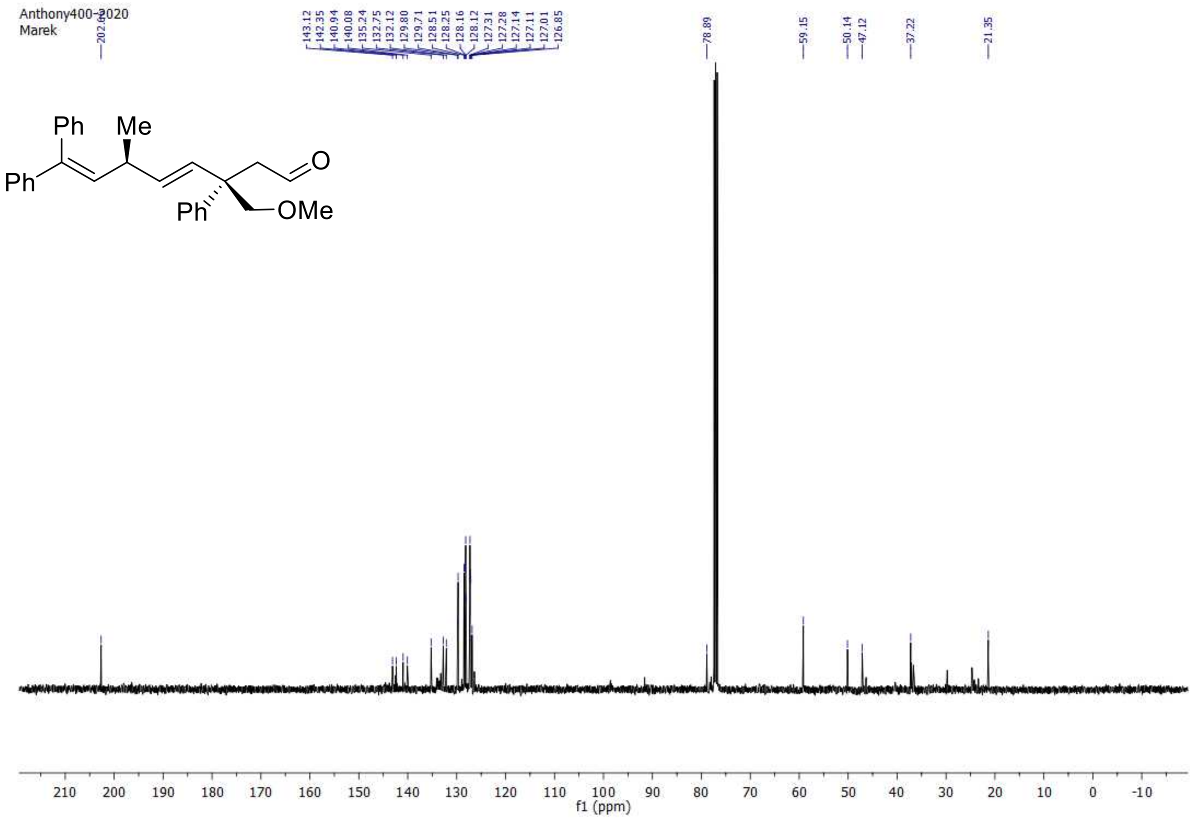
Anthony400-2020
Marek
202.60
143.12
142.35
140.94
140.08
135.24
132.75
132.12
129.80
129.71
128.51
128.25
128.16
128.12
127.31
127.28
127.14
127.11
127.01
126.85
78.89
59.15
50.14
47.12
37.22
21.35
Ph
Me
Ph
O
Ph
OMe
210
200
190
180
170
160
150
140
130
120
110
100
90
80
70
60
50
40
30
20
10
0
-10
f1 (ppm)

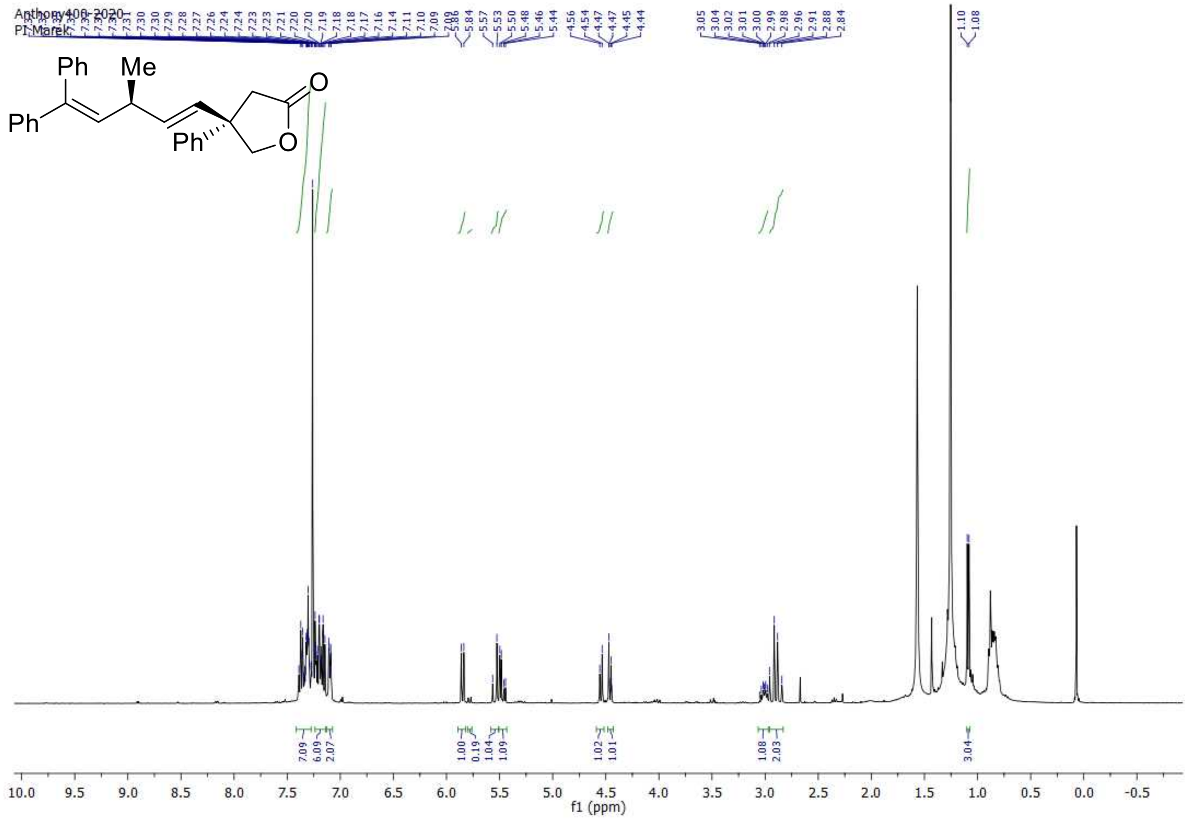

Anthony400-2020
PI Marek
Ph
Me
O
Ph
Ph
O
f1 (ppm)

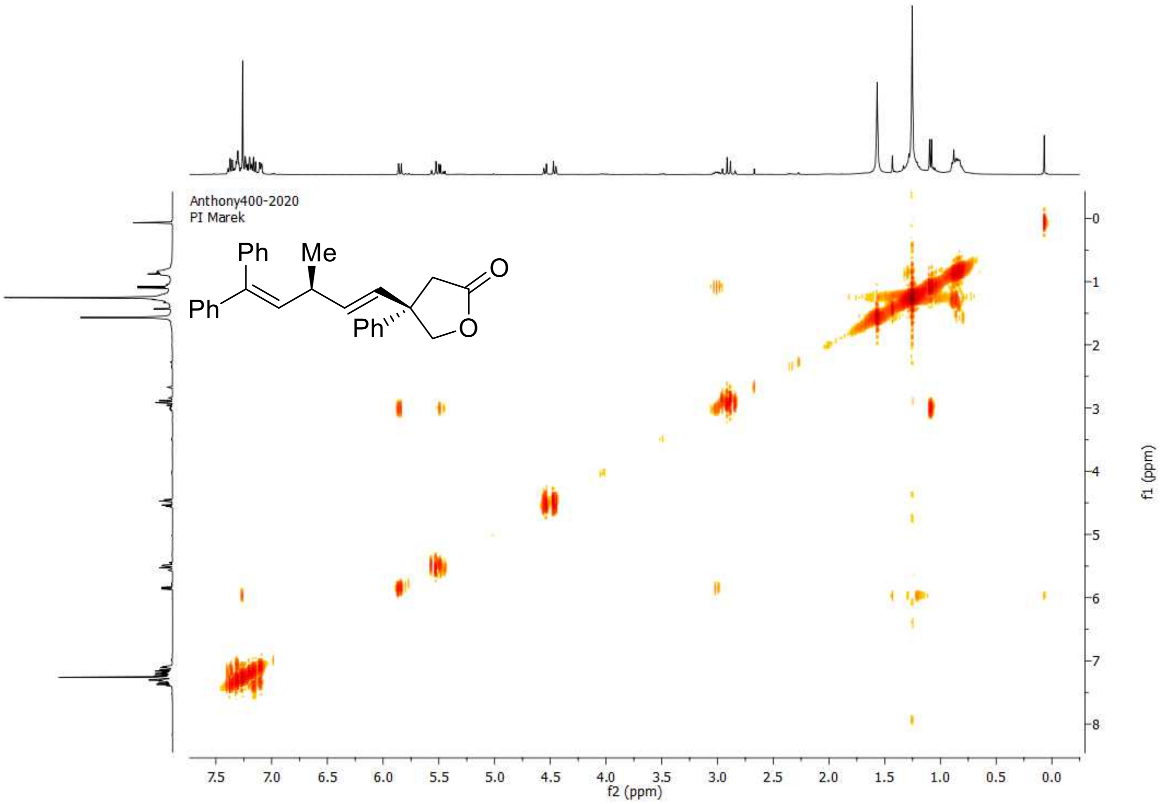

Anthony400-2020
PI Marek
Ph
Me
O
Ph
Ph
O
f2 (ppm)
f1 (ppm)

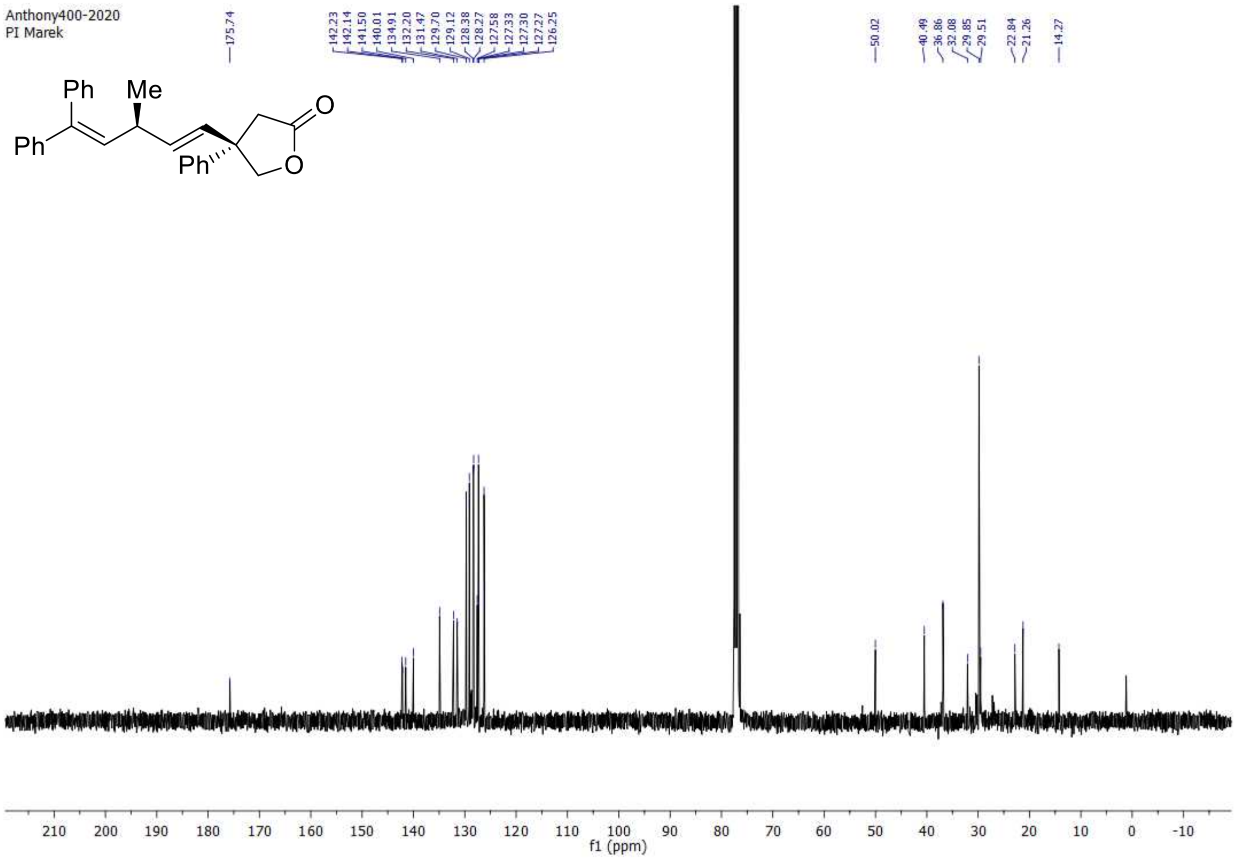

Anthony400-2020
PI Marek
175.74
142.23
142.14
141.50
140.01
134.91
132.20
131.47
129.70
129.12
128.38
128.27
127.58
127.33
127.30
127.27
126.25
50.02
40.49
36.86
32.08
29.85
29.51
22.84
21.26
14.27
Ph
Me
O
Ph
Ph
O
f1 (ppm)

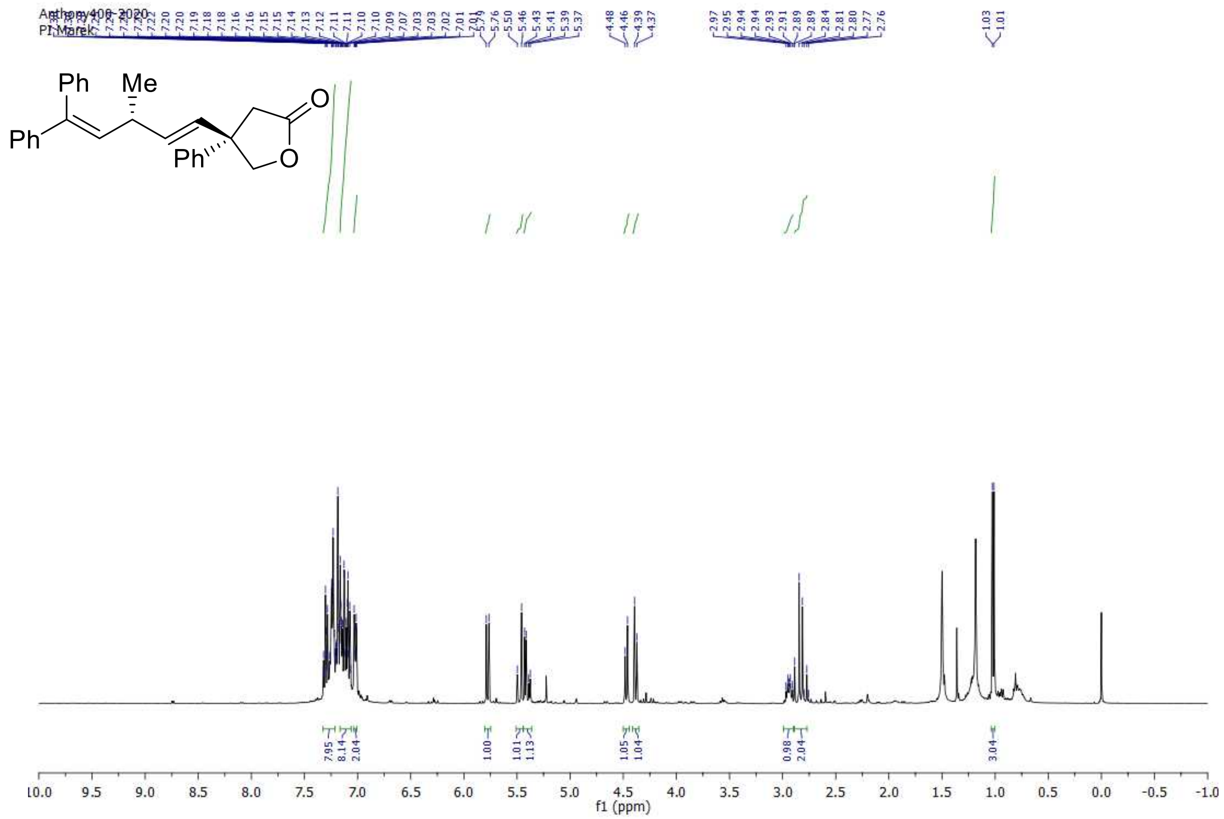
Anthony400-2020
PI Marek
Ph
Me
Ph
Ph
O
O
f1 (ppm)

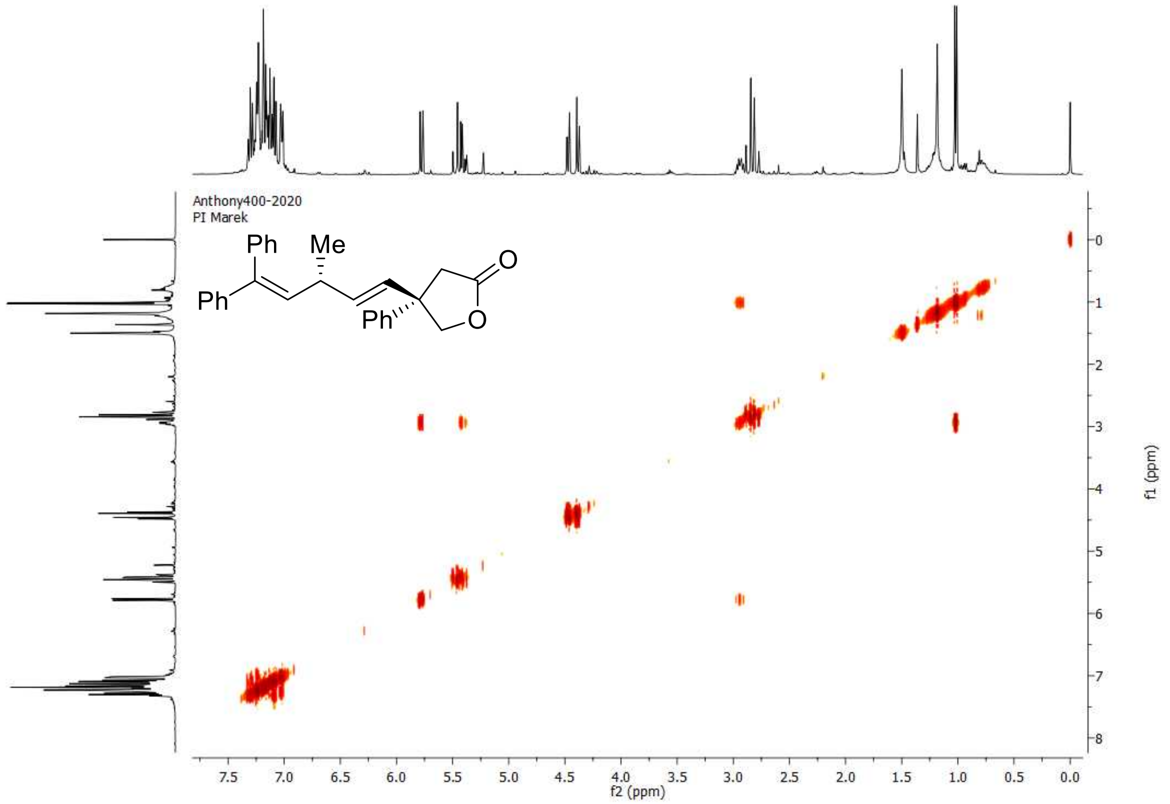
Anthony400-2020
PI Marek
Ph
Me
Ph
Ph
O
O
f2 (ppm)
f1 (ppm)

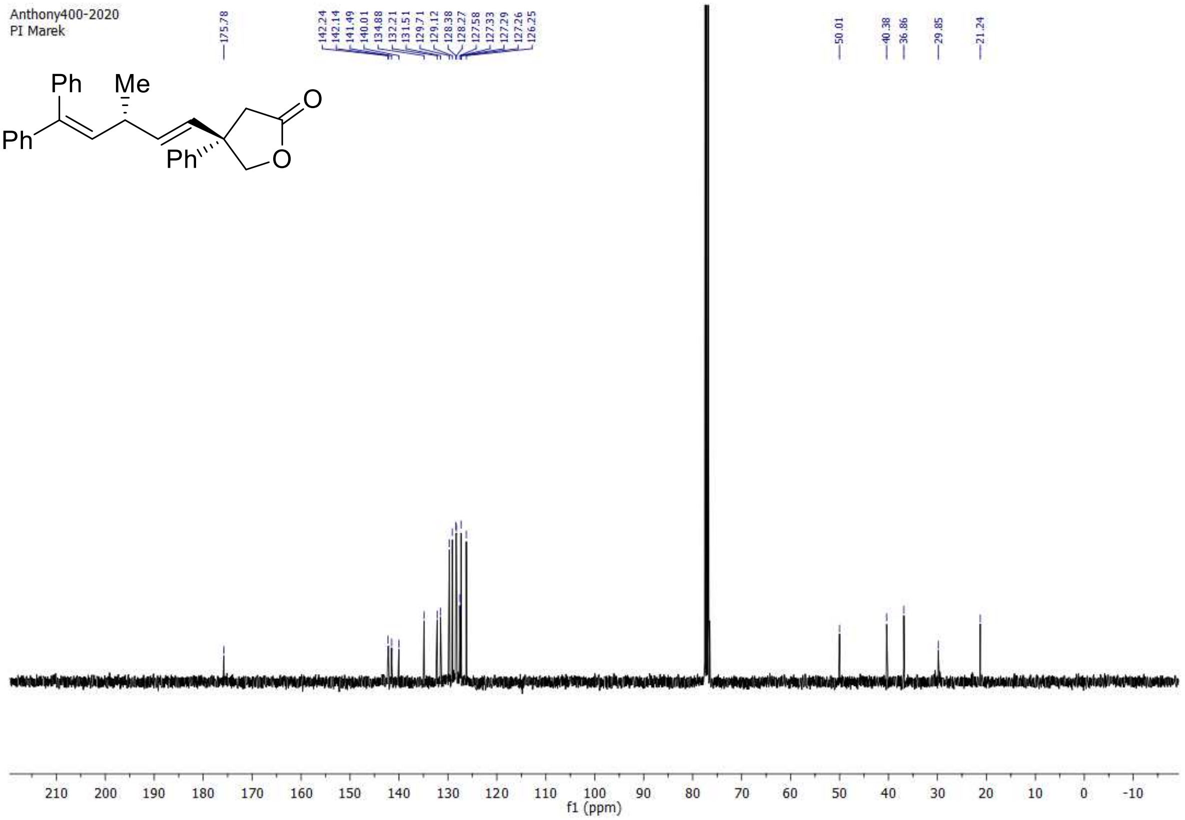
Anthony400-2020
PI Marek
Ph
Me
Ph
Ph
O
O
175.78
142.24
142.14
141.49
140.01
134.88
132.21
131.51
129.71
129.12
128.38
128.27
127.58
127.33
127.29
127.26
126.25
50.01
40.38
36.86
29.85
21.24
f1 (ppm)

Overlay comparison of ^{13}CNMR 14a and 14b

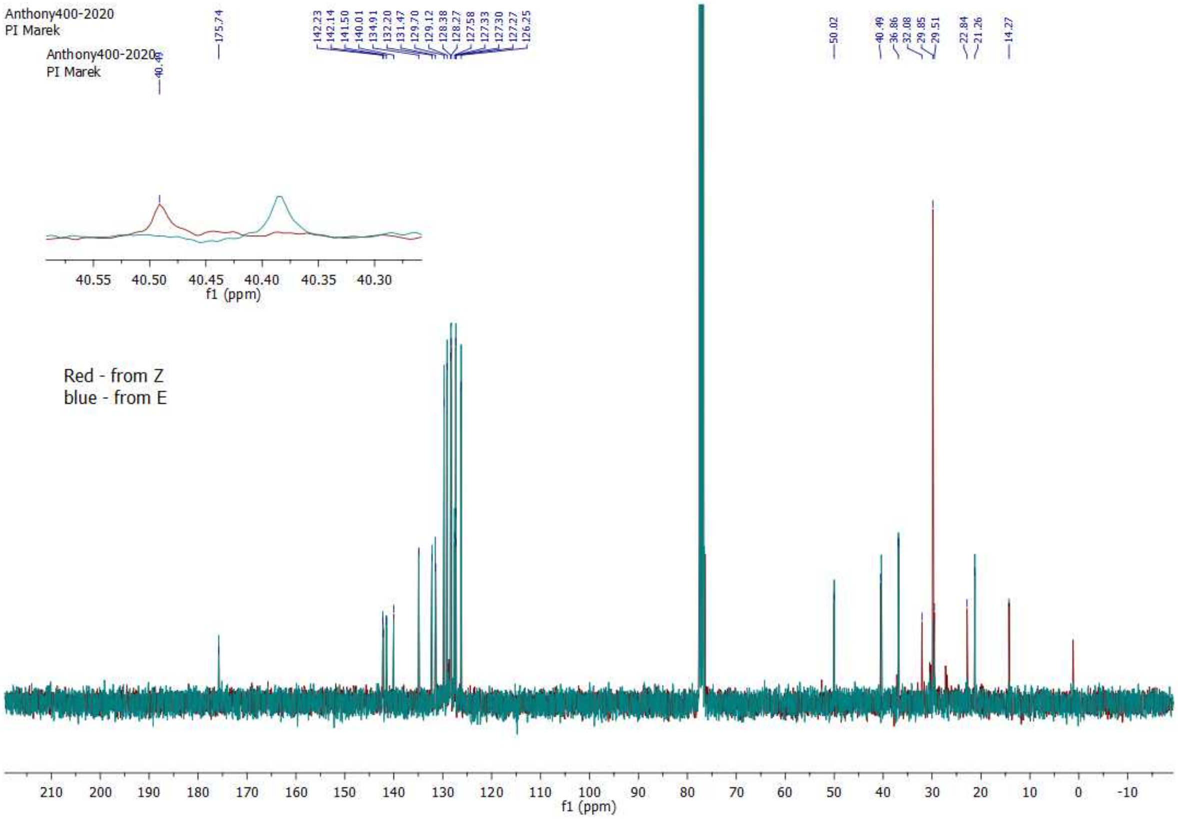

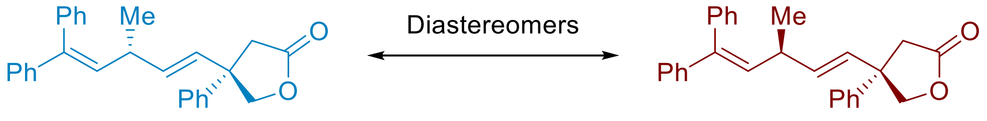

11o (in blue)
dr > 95:5

11p (in red)
dr = 10:1

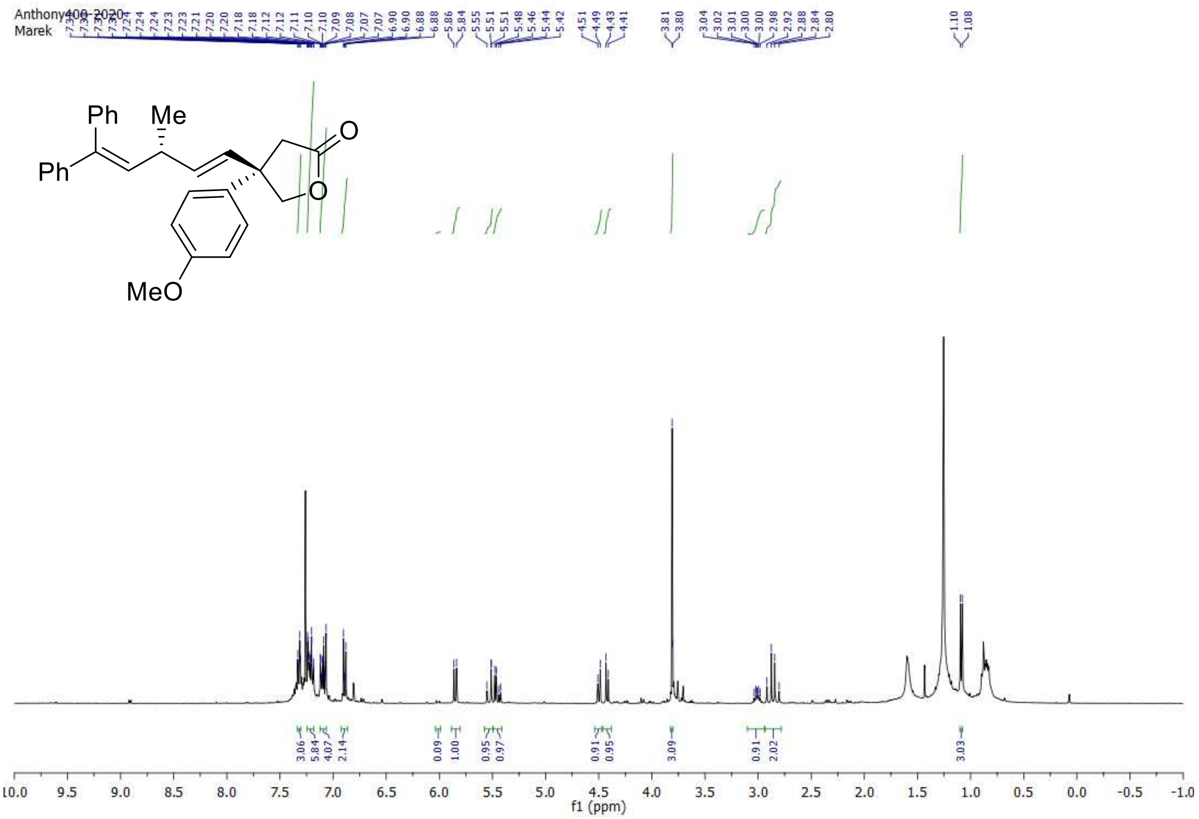
Anthony400-2020
Marek
Ph
Me
Ph
O
O
MeO
3.06
5.84
4.07
2.14
0.09
1.00
0.95
0.97
0.91
0.95
3.09
0.91
2.02
3.03
f1 (ppm)

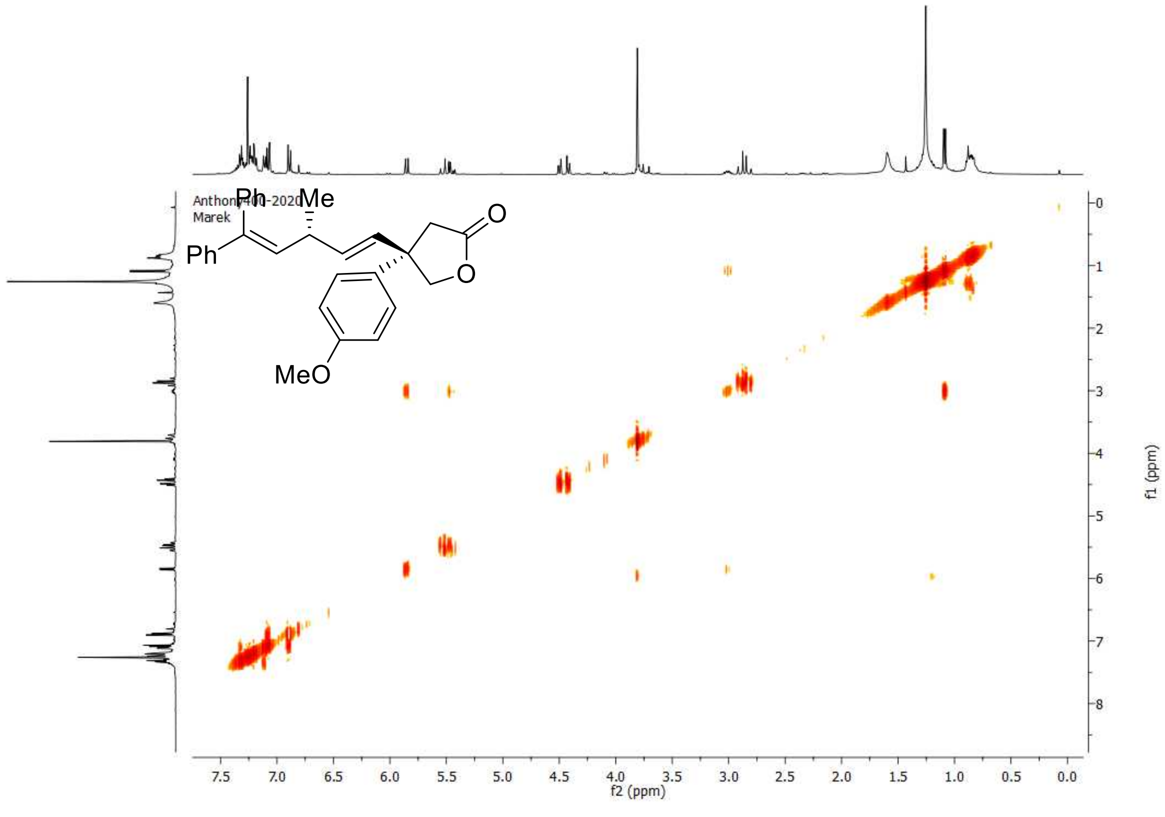
Anthony400-2020
Marek
Ph
Me
Ph
O
O
MeO
f2 (ppm)
f1 (ppm)

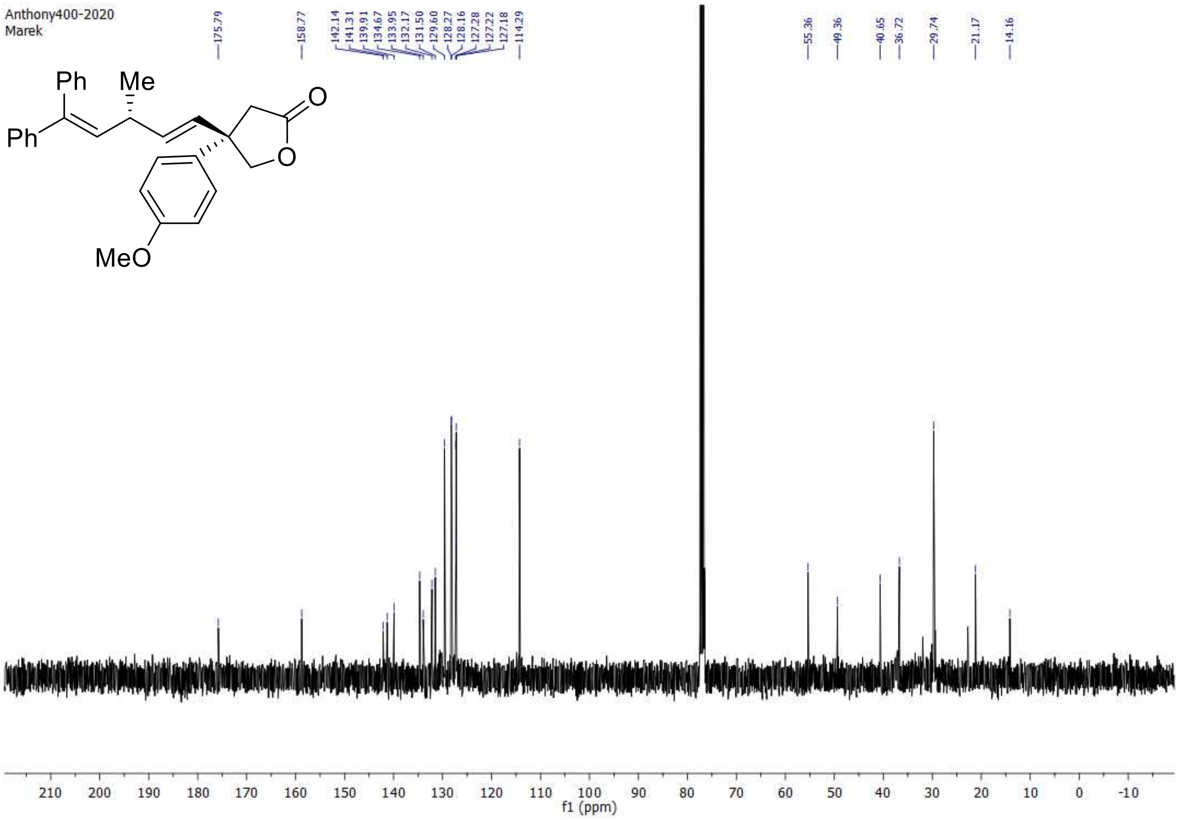
Anthony400-2020
Marek
175.79
158.77
142.14
141.31
139.91
134.67
133.95
132.17
131.50
129.60
128.27
128.16
127.28
127.22
127.18
114.29
55.36
49.36
40.65
36.72
29.74
21.17
14.16
Ph
Me
O
Ph
O
MeO
210
200
190
180
170
160
150
140
130
120
110
100
90
80
70
60
50
40
30
20
10
0
-10
f1 (ppm)

List of XYZ coordinates (geometries)

Key-role transition states accompanied by their respective reactants and products. For example:
Transition state 1 - TS1;
The reactant of transition state 1 – TS1_R:
The product of transition state 1 – TS1_P.

Path_I

49
I_a_maj

46	0.548756000	0.212666000	-0.597075000
6	-2.364743000	-0.923489000	-0.187101000
6	-3.769816000	-0.898662000	-0.327426000
6	-4.333416000	-0.229575000	-1.418805000
6	-3.484103000	0.378347000	-2.359329000
6	-2.101944000	0.319932000	-2.146632000
7	-1.549913000	-0.296089000	-1.078717000
1	-5.426490000	-0.194974000	-1.542964000
1	-4.399462000	-1.415157000	0.411164000
1	-1.398493000	0.803174000	-2.842752000
6	-1.753112000	-1.600550000	0.951713000
6	-0.127724000	-2.505698000	2.093147000
6	-2.187138000	-1.935727000	2.220272000
7	-1.135896000	-2.503403000	2.928004000
1	-3.178043000	-1.774787000	2.662450000
8	-0.427826000	-1.980240000	0.871414000
1	0.894758000	-2.878576000	2.236759000
1	-3.881297000	0.900302000	-3.241871000
6	2.456673000	0.634191000	0.026843000
6	3.081295000	-0.684903000	0.343636000
6	3.209983000	-1.781038000	-0.730878000
6	4.441182000	-1.077389000	-0.229077000
1	2.852128000	-1.065246000	1.352794000
1	3.036862000	-2.807217000	-0.363565000
1	5.131372000	-1.636541000	0.422342000
1	4.920125000	-0.346092000	-0.901073000
6	2.662609000	-1.494095000	-2.103811000
1	2.867204000	-2.333510000	-2.800007000
1	3.095781000	-0.570957000	-2.533355000
8	1.221690000	-1.262607000	-2.094651000
1	0.766283000	-2.109936000	-1.895107000

1	2.937676000	1.177384000	-0.813522000
6	2.080581000	1.531491000	1.211737000
6	0.563957000	1.709047000	1.013978000
6	-0.382750000	1.276067000	1.997951000
6	0.079014000	2.447623000	-0.122465000
6	-1.732044000	1.603784000	1.878124000
1	-0.018999000	0.694446000	2.859981000
6	-1.300484000	2.780399000	-0.216446000
1	0.791052000	2.919603000	-0.817445000
6	-2.192094000	2.363860000	0.772115000
1	-2.446206000	1.269772000	2.646238000
1	-1.654983000	3.373958000	-1.072921000
1	-3.259230000	2.624412000	0.696655000
1	2.222773000	0.964973000	2.155350000
6	2.852136000	2.851332000	1.291033000
1	2.492657000	3.472595000	2.138511000
1	3.935125000	2.658199000	1.439220000
1	2.737970000	3.441079000	0.358001000

49

TS1_E_s_trans_P

46	0.547748	0.213070	-0.596436
6	-2.363111	-0.926815	-0.185343
6	-3.768424	-0.904199	-0.323619
6	-4.334715	-0.234462	-1.413215
6	-3.487726	0.376245	-2.354015
6	-2.105168	0.319822	-2.143460
7	-1.550577	-0.296760	-1.077194
1	-5.428018	-0.201476	-1.535800
1	-4.396054	-1.422900	0.415139
1	-1.403511	0.805110	-2.839965
6	-1.748687	-1.604586	0.951592
6	-0.120696	-2.510872	2.088498
6	-2.179764	-1.940839	2.220853
7	-1.126981	-2.509436	2.925590
1	-3.169652	-1.780207	2.665420
8	-0.423492	-1.984059	0.867968
1	0.902029	-2.884131	2.229341
1	-3.887055	0.898846	-3.235214
6	2.454676	0.635939	0.029481
6	3.084117	-0.682227	0.340965
6	3.214542	-1.774748	-0.737037
6	4.444198	-1.068367	-0.235282

1	2.857701	-1.066658	1.349167
1	3.045615	-2.802542	-0.372273
1	5.137543	-1.627122	0.413129
1	4.919366	-0.333356	-0.905900
6	2.663927	-1.485977	-2.108270
1	2.869349	-2.323370	-2.806642
1	3.093912	-0.560764	-2.536590
8	1.222396	-1.258236	-2.096066
1	0.769379	-2.107369	-1.898753
1	2.934560	1.184553	-0.808020
6	2.074992	1.527227	1.217814
6	0.559399	1.707958	1.015445
6	-0.392027	1.275788	1.995217
6	0.080414	2.449234	-0.121789
6	-1.740178	1.606501	1.870462
1	-0.032827	0.692512	2.858052
6	-1.297861	2.785201	-0.220688
1	0.796360	2.920554	-0.813162
6	-2.194288	2.368915	0.763691
1	-2.458061	1.272560	2.635157
1	-1.647695	3.380930	-1.077590
1	-3.260572	2.631779	0.684312
1	2.213003	0.955267	2.158780
6	2.847608	2.845873	1.307235
1	2.485890	3.462426	2.157193
1	3.929974	2.651031	1.457769
1	2.736676	3.441162	0.377321

49

TS1_E_s_trans_R

46	0.619517	0.317003	-0.587247
6	-1.802157	-1.561125	-0.267836
6	-3.031082	-2.123803	-0.672760
6	-3.465518	-1.953261	-1.991107
6	-2.654333	-1.242456	-2.891997
6	-1.450915	-0.703500	-2.428398
7	-1.039510	-0.846812	-1.146090
1	-4.423857	-2.384556	-2.317726
1	-3.619309	-2.704778	0.051548
1	-0.789795	-0.119417	-3.086045
6	-1.344220	-1.716037	1.107675
6	0.112232	-1.846810	2.723767
6	-1.990531	-1.835813	2.323627

7	-1.044462	-1.910583	3.335330
1	-3.069737	-1.838655	2.520179
8	0.010470	-1.733691	1.369517
1	1.124385	-1.888651	3.146152
1	-2.946375	-1.096316	-3.941675
6	2.651832	1.073687	-0.172598
6	3.162087	0.117405	0.836981
6	3.452153	-1.319706	0.369360
6	4.589224	-0.404197	0.740571
1	2.729936	0.219486	1.845273
1	3.176175	-2.107321	1.089837
1	5.119009	-0.587815	1.688201
1	5.216538	-0.012544	-0.077274
6	3.191813	-1.635957	-1.084632
1	3.431422	-2.694733	-1.311755
1	3.805667	-1.000223	-1.750662
8	1.808454	-1.392412	-1.487446
1	1.273648	-2.173100	-1.227903
1	3.142334	1.048549	-1.163540
6	1.809724	2.156495	0.084993
6	-0.768986	1.512378	0.218734
6	-1.012519	1.466415	1.604487
6	-1.612439	2.264385	-0.620218
6	-2.114305	2.157840	2.146108
1	-0.345710	0.903558	2.277485
6	-2.711789	2.955270	-0.071448
1	-1.429777	2.308193	-1.705922
6	-2.967307	2.900655	1.310538
1	-2.301027	2.112376	3.231478
1	-3.372587	3.536408	-0.735434
1	-3.828257	3.439940	1.736581
1	1.508879	2.328464	1.132474
6	1.662141	3.322992	-0.851696
1	0.646884	3.764463	-0.803965
1	2.375924	4.121739	-0.548809
1	1.883655	3.044242	-1.901285

49

TS1_E_s_trans

46	0.656947000	0.352478000	-0.591526000
6	-1.974126000	-1.327581000	-0.477494000
6	-3.353933000	-1.530713000	-0.697986000
6	-3.993170000	-0.820117000	-1.720052000

6	-3.238881000	0.058314000	-2.514499000
6	-1.877168000	0.215242000	-2.231973000
7	-1.258609000	-0.442594000	-1.225987000
1	-5.069579000	-0.962620000	-1.904216000
1	-3.899874000	-2.255537000	-0.078022000
1	-1.250082000	0.910281000	-2.811507000
6	-1.291281000	-2.041555000	0.596587000
6	0.418244000	-2.897024000	1.656431000
6	-1.685114000	-2.531552000	1.827301000
7	-0.581145000	-3.061578000	2.483336000
1	-2.683225000	-2.498970000	2.279177000
8	0.064630000	-2.289993000	0.485420000
1	1.472054000	-3.184693000	1.768152000
1	-3.693780000	0.624473000	-3.340354000
6	2.439863000	1.162506000	0.067893000
6	3.169084000	0.036133000	0.719605000
6	3.546121000	-1.197550000	-0.124706000
6	4.618814000	-0.256105000	0.363197000
1	2.854944000	-0.186013000	1.752508000
1	3.453412000	-2.162068000	0.403235000
1	5.285600000	-0.598958000	1.169265000
1	5.090378000	0.404568000	-0.383912000
6	3.159731000	-1.222234000	-1.583072000
1	3.537975000	-2.141014000	-2.076063000
1	3.563001000	-0.347466000	-2.126019000
8	1.714923000	-1.164680000	-1.800022000
1	1.319260000	-1.986090000	-1.435397000
1	2.900072000	1.563987000	-0.857453000
6	1.624817000	2.093560000	0.813171000
6	-0.365743000	1.581639000	0.703673000
6	-0.796340000	0.919612000	1.879017000
6	-1.212620000	2.527931000	0.083343000
6	-2.088153000	1.145808000	2.379693000
1	-0.121431000	0.215468000	2.392581000
6	-2.509736000	2.740767000	0.582641000
1	-0.872938000	3.071657000	-0.811978000
6	-2.951209000	2.047490000	1.725733000
1	-2.424602000	0.611368000	3.282225000
1	-3.179438000	3.452552000	0.074104000
1	-3.966167000	2.220429000	2.116551000
1	1.605693000	1.920273000	1.901988000
6	1.621634000	3.546473000	0.402318000

1	0.837138000	4.126939000	0.924614000
1	2.608955000	3.980793000	0.672525000
1	1.489905000	3.666982000	-0.691379000

Path_II

49

TS1_E_s_cis_P

46	-0.697139	-0.182411	0.133699
6	2.464660	-0.278744	0.629709
6	3.658017	0.224493	1.195503
6	3.585882	1.104358	2.280376
6	2.326584	1.451500	2.795413
6	1.187542	0.917610	2.181513
7	1.245695	0.088679	1.116347
1	4.507352	1.510680	2.724511
1	4.627396	-0.081666	0.779512
1	0.177384	1.172554	2.538778
6	2.513945	-1.197063	-0.502288
6	3.519837	-2.290372	-2.098451
6	1.605166	-1.970247	-1.205294
7	2.266369	-2.653282	-2.214495
1	0.521859	-2.064377	-1.046509
8	3.748155	-1.415276	-1.084165
1	4.387098	-2.593485	-2.700277
1	2.218799	2.131029	3.652915
6	-2.536234	-0.242987	-0.765312
6	-3.649325	-0.783324	0.089137
6	-3.506857	-1.918418	1.085769
6	-3.754514	-0.504470	1.578487
1	-4.614041	-0.792874	-0.455892
1	-4.365044	-2.610107	1.134196
1	-4.738389	-0.262842	2.010710
1	-2.902639	0.022522	2.041634
6	-2.215862	-2.659179	1.342572
1	-1.966131	-3.329128	0.490742
1	-2.313995	-3.276756	2.260831
8	-1.121076	-1.731083	1.565967
1	-0.299744	-2.238420	1.744038
1	-2.371861	-0.871737	-1.669416
6	-2.613132	1.251844	-1.116791
6	-1.154337	1.694638	-0.937775
6	-0.128746	1.071911	-1.732814
6	-0.784053	2.779393	-0.079259

6 1.200647 1.583529 -1.706637
1 -0.403248 0.343983 -2.512268
6 0.528144 3.245476 -0.051835
1 -1.562310 3.250635 0.541810
6 1.523037 2.651934 -0.874040
1 1.964886 1.127641 -2.353634
1 0.797140 4.085256 0.607545
1 2.552620 3.041062 -0.849626
1 -3.210801 1.770018 -0.337836
6 -3.189725 1.571526 -2.499556
1 -3.185453 2.666087 -2.688786
1 -2.600284 1.080368 -3.301803
1 -4.237356 1.213088 -2.579259
49
TS1_E_s_cis_R
46 -0.759171 -0.064592 -0.097869
6 2.031011 -1.144128 0.628308
6 3.060743 -1.504127 1.526853
6 2.828797 -1.446930 2.904090
6 1.567593 -1.040035 3.370064
6 0.593692 -0.684454 2.432987
7 0.819727 -0.728223 1.099657
1 3.626780 -1.723395 3.609657
1 4.029634 -1.834111 1.128535
1 -0.402171 -0.335050 2.743525
6 2.249583 -1.217167 -0.811131
6 3.488236 -1.408184 -2.593686
6 1.458361 -1.223435 -1.948822
7 2.268519 -1.340318 -3.067462
1 0.363904 -1.148690 -2.016863
8 3.556844 -1.346010 -1.238202
1 4.437651 -1.504024 -3.137241
1 1.335592 -0.983912 4.443055
6 -2.635387 0.121170 -1.224029
6 -3.791864 -0.082654 -0.288966
6 -3.963098 -1.426275 0.389297
6 -3.642684 -0.203537 1.222159
1 -4.720943 0.395155 -0.650119
1 -4.995201 -1.809195 0.454700
1 -4.440735 0.215717 1.854387
1 -2.623449 -0.106285 1.635471
6 -2.980941 -2.550225 0.139987

1	-3.120585	-2.953363	-0.887834
1	-3.154613	-3.377706	0.861468
8	-1.609674	-2.103876	0.302541
1	-1.007722	-2.823549	0.018040
1	-2.518419	-0.655819	-2.007243
6	-1.988811	1.333474	-1.462478
6	0.130587	1.717493	0.128351
6	1.250872	2.098289	-0.629726
6	-0.310735	2.519975	1.197208
6	1.934979	3.289670	-0.311643
1	1.604277	1.477471	-1.466597
6	0.378409	3.709460	1.507604
1	-1.192287	2.230577	1.791878
6	1.502305	4.096383	0.755235
1	2.815146	3.581603	-0.907645
1	0.027944	4.335335	2.344586
1	2.039535	5.026483	0.999733
1	-2.264983	2.196134	-0.831317
6	-1.285553	1.647212	-2.751584
1	-0.420773	2.322698	-2.603552
1	-0.947592	0.732028	-3.277282
1	-2.000746	2.180152	-3.417930

49

TS1_E_s_cis

46	0.876431	-0.154884	0.112953
6	-2.052525	-1.238167	0.276708
6	-3.220236	-1.826520	-0.256645
6	-3.128702	-2.592688	-1.423397
6	-1.872651	-2.771454	-2.026523
6	-0.760415	-2.146861	-1.450899
7	-0.845160	-1.382752	-0.339680
1	-4.030232	-3.054437	-1.854115
1	-4.181969	-1.685664	0.255718
1	0.239291	-2.234006	-1.904025
6	-2.101603	-0.441828	1.497258
6	-3.062180	0.822203	2.988020
6	-1.188690	-0.069285	2.468957
7	-1.821303	0.740098	3.401208
1	-0.127685	-0.338371	2.551109
8	-3.310470	0.123140	1.847778
1	-3.905311	1.366522	3.433676
1	-1.750036	-3.372247	-2.939092

6	2.505766	1.054790	0.465756
6	3.774215	0.430817	-0.047608
6	4.163187	-0.986877	0.325531
6	3.792674	-0.660191	-1.108209
1	4.617555	1.146486	-0.077830
1	5.237246	-1.142146	0.523437
1	4.594830	-0.646330	-1.862613
1	2.813093	-1.007100	-1.479058
6	3.326231	-1.857221	1.238700
1	3.367733	-1.471045	2.281408
1	3.722611	-2.895098	1.235452
8	1.943092	-1.920649	0.801907
1	1.427172	-2.441389	1.453307
1	2.500231	1.227012	1.563662
6	1.793151	2.053445	-0.300022
6	-0.061287	1.372791	-0.912822
6	-1.161846	1.997365	-0.284403
6	-0.095025	1.136308	-2.308220
6	-2.315511	2.298676	-1.027253
1	-1.128754	2.235752	0.788491
6	-1.252173	1.438341	-3.044952
1	0.780096	0.696756	-2.813357
6	-2.369055	2.008374	-2.403849
1	-3.179995	2.760121	-0.523976
1	-1.280152	1.229177	-4.126360
1	-3.276410	2.243920	-2.982199
1	2.130957	2.160148	-1.345264
6	1.404987	3.342064	0.383749
1	0.728454	3.963946	-0.232612
1	0.937886	3.160351	1.371904
1	2.341938	3.916445	0.559088

Pd-> Double Bond_Dissociation

61

1DMF

46	-0.071088	0.144819	-0.550310
6	2.666824	1.372512	-0.112987
6	3.559122	2.280577	0.494218
6	3.058632	3.368747	1.216669
6	1.668028	3.546827	1.297979
6	0.832394	2.612890	0.680060
7	1.313841	1.539457	0.004323

1	3.747444	4.079700	1.697316
1	4.640160	2.124295	0.371665
1	-0.262046	2.694572	0.736694
6	3.188985	0.217313	-0.836426
6	3.251568	-1.322817	-2.370458
6	4.254606	-0.633435	-0.612100
7	4.268796	-1.610823	-1.596457
1	4.974023	-0.613486	0.215808
8	2.555625	-0.220330	-1.979803
1	2.905928	-1.833198	-3.279002
1	1.222272	4.392633	1.840548
6	-2.780546	1.103244	1.236378
6	-2.796507	2.519678	0.799552
6	-2.649896	2.939902	-0.671076
6	-3.985746	3.062464	0.016961
1	-2.334043	3.220479	1.515774
1	-2.101109	3.886700	-0.827306
1	-4.360430	4.068985	0.261084
1	-4.765209	2.333023	-0.261316
6	-2.464054	1.943912	-1.783885
1	-2.834352	2.362460	-2.745314
1	-3.004253	0.997649	-1.594089
8	-1.073817	1.559568	-1.973371
1	-0.542945	2.374116	-2.108136
1	-3.335101	0.381174	0.608867
6	-2.189504	0.662727	2.371624
6	0.890348	-1.167110	0.565291
6	1.287790	-0.861919	1.886056
6	1.226604	-2.423563	0.014268
6	2.019024	-1.799420	2.639853
1	1.039512	0.113574	2.333121
6	1.965993	-3.353279	0.771076
1	0.926958	-2.684998	-1.013134
6	2.366982	-3.043654	2.082973
1	2.322685	-1.548915	3.669627
1	2.235093	-4.324333	0.324126
1	2.948148	-3.771522	2.671262
1	-1.639692	1.395407	2.993874
6	-2.221057	-0.750368	2.871065
1	-2.865544	-1.396875	2.242141
1	-1.201095	-1.195708	2.896761
1	-2.603251	-0.798703	3.914992

8	-1.582941	-1.171094	-1.094814
6	-2.038449	-2.087484	-0.345200
1	-1.425309	-2.536684	0.462984
7	-3.267492	-2.588574	-0.480073
6	-4.205077	-2.071842	-1.471580
1	-3.744256	-1.224888	-2.009744
1	-4.470286	-2.869606	-2.195980
1	-5.131474	-1.733420	-0.963477
6	-3.726752	-3.680584	0.371775
1	-3.985544	-4.564072	-0.248336
1	-2.929303	-3.960064	1.084996
1	-4.629768	-3.367475	0.935311

49

1NoDMF

46	0.651464	-0.096765	-0.716031
6	0.794697	2.657164	0.244600
6	1.299722	3.816020	0.864973
6	2.628721	3.836853	1.299618
6	3.430351	2.703010	1.089572
6	2.871324	1.577254	0.477630
7	1.577219	1.541514	0.076639
1	3.038580	4.735246	1.784818
1	0.644465	4.690593	0.983046
1	3.461121	0.667351	0.305173
6	-0.569422	2.607007	-0.256076
6	-2.211368	1.726044	-1.440412
6	-1.720131	3.354673	-0.123372
7	-2.734059	2.778217	-0.879849
1	-1.874426	4.260449	0.475306
8	-0.882535	1.554781	-1.113644
1	-2.651340	0.995599	-2.131917
1	4.486164	2.678438	1.394911
6	-4.036681	-1.109598	0.776370
6	-2.875550	-1.829384	1.356607
6	-1.681557	-2.276142	0.492304
6	-1.533755	-1.124422	1.461200
1	-3.129414	-2.530404	2.169686
1	-1.219591	-3.227682	0.808268
1	-0.916923	-1.286344	2.358774
1	-1.483741	-0.099423	1.054303
6	-1.691189	-2.064171	-0.995336
1	-2.326155	-1.212536	-1.296481

1	-2.048459	-2.966663	-1.532533
8	-0.360265	-1.751818	-1.550127
1	0.223217	-2.536726	-1.417469
1	-3.813410	-0.321389	0.031344
6	-5.324857	-1.331462	1.121763
6	2.036578	-1.365050	-0.158679
6	2.809341	-2.015401	-1.140991
6	2.212080	-1.655357	1.208213
6	3.773341	-2.965217	-0.741882
1	2.681908	-1.785172	-2.211395
6	3.176199	-2.607878	1.590948
1	1.609064	-1.144549	1.974908
6	3.957053	-3.261370	0.619577
1	4.383537	-3.469757	-1.508519
1	3.314373	-2.837738	2.659990
1	4.711537	-4.003003	0.926037
1	-5.539946	-2.113073	1.876698
6	-6.506296	-0.591302	0.568009
1	-7.078392	-0.080041	1.374801
1	-6.203797	0.173690	-0.177012
1	-7.227041	-1.285145	0.079505

12

DMF

6	-0.860372000	-0.633035000	-0.000159000
1	-0.766928000	-1.730893000	-0.000180000
8	-1.949788000	-0.081042000	-0.000006000
7	0.333670000	-0.021388000	-0.000193000
6	1.567074000	-0.773715000	0.000073000
1	2.160448000	-0.534535000	-0.886400000
1	2.159401000	-0.535763000	0.887585000
1	1.352124000	-1.841729000	-0.000811000
6	0.447334000	1.418749000	0.000157000
1	-0.546891000	1.858519000	0.000562000
1	0.990360000	1.754980000	0.887208000
1	0.989883000	1.755487000	-0.886991000

49

1Z

46	0.721920	0.267657	-0.351046
6	-2.035126	1.217846	0.740858
6	-3.191768	2.024544	0.709984
6	-3.187932	3.199319	-0.049340
6	-2.018629	3.564917	-0.736991

6	-0.904421	2.723059	-0.659634
7	-0.917807	1.566963	0.043235
1	-4.085332	3.835162	-0.089911
1	-4.071527	1.726458	1.297781
1	0.028078	2.959157	-1.194446
6	-2.019039	-0.022383	1.506092
6	-1.120581	-1.635266	2.661936
6	-2.975314	-0.965595	1.829656
7	-2.378431	-1.983950	2.559185
1	-4.036049	-0.978622	1.550870
8	-0.827426	-0.450039	2.056569
1	-0.295707	-2.150433	3.171131
1	-1.963011	4.487156	-1.332796
6	2.435485	-1.088035	-0.598486
6	3.719353	-0.743264	0.095878
6	3.893101	0.269644	1.237945
6	4.030677	-1.214943	1.494504
1	4.572781	-0.722328	-0.605345
1	4.829253	0.850347	1.182420
1	5.032249	-1.621661	1.704103
1	3.204720	-1.695979	2.045628
6	2.713124	1.036041	1.771948
1	3.035916	1.771097	2.538774
1	1.954086	0.371509	2.227380
8	1.992260	1.754836	0.723528
1	2.645085	2.245922	0.176620
1	1.993546	-2.065826	-0.340350
6	2.088962	-0.533574	-1.848712
6	-0.601874	-1.045786	-1.041293
6	-0.671913	-2.340334	-0.489997
6	-1.543473	-0.643708	-2.010532
6	-1.711185	-3.211105	-0.874164
1	0.057836	-2.679877	0.260802
6	-2.572635	-1.523199	-2.396368
1	-1.492140	0.361360	-2.457870
6	-2.665259	-2.804621	-1.822470
1	-1.771754	-4.212789	-0.418532
1	-3.310911	-1.196855	-3.146959
1	-3.478347	-3.486587	-2.117790
6	2.851655	0.566113	-2.530003
1	2.178781	1.201793	-3.140632
1	3.416309	1.209324	-1.827105

1	3.589058	0.113648	-3.232267
1	1.434529	-1.131706	-2.505961

PathI

49

TS1_s_trans_PathI_P

46	-0.643353	-0.171893	0.051201
6	2.469844	-0.227158	0.763956
6	3.643503	0.265355	1.377999
6	3.538106	1.250542	2.364283
6	2.262990	1.710233	2.735829
6	1.146711	1.176181	2.082735
7	1.240518	0.242756	1.109947
1	4.442934	1.647833	2.848933
1	4.622448	-0.138714	1.083307
1	0.127177	1.514023	2.327793
6	2.575175	-1.223677	-0.294974
6	1.870382	-2.737662	-1.695145
6	3.602537	-1.616119	-1.134596
7	3.128785	-2.574577	-2.018885
1	4.634587	-1.245678	-1.164609
8	1.465146	-1.957132	-0.655696
1	1.120790	-3.409773	-2.133491
1	2.128841	2.474044	3.515291
6	-2.451344	-0.342528	-0.889511
6	-3.592828	-0.703081	0.023190
6	-3.493775	-1.758430	1.106507
6	-3.659124	-0.298429	1.485795
1	-4.565046	-0.695710	-0.507372
1	-4.385666	-2.395513	1.233756
1	-4.623343	0.029557	1.904854
1	-2.781988	0.237940	1.890081
6	-2.219812	-2.533645	1.376965
1	-1.982905	-3.220520	0.539257
1	-2.325107	-3.142336	2.299665
8	-1.038914	-1.705532	1.533358
1	-1.079537	-1.267941	2.412207
1	-2.283509	-1.136972	-1.649421
6	-2.461585	1.066937	-1.533139
6	-0.992159	1.494857	-1.318191
6	0.035917	0.744992	-1.993407
6	-0.599685	2.667681	-0.594114

6 1.379303 1.207116 -1.999528
1 -0.248186 -0.076041 -2.670319
6 0.730836 3.087318 -0.588975
1 -1.361514 3.252960 -0.059560
6 1.721791 2.364475 -1.300299
1 2.138408 0.647861 -2.566386
1 1.012703 3.993963 -0.031484
1 2.763817 2.719987 -1.298057
6 -3.482758 2.046593 -0.959828
1 -3.365425 2.188261 0.133244
1 -3.389357 3.039729 -1.445683
1 -4.513296 1.681842 -1.140303
1 -2.631683 0.983029 -2.629019
49
TS1_s_trans_PathI_R
46 -0.722921 -0.125275 0.370132
6 1.957709 -1.388732 -0.580262
6 3.063060 -2.251759 -0.429543
6 2.987381 -3.310620 0.481601
6 1.796345 -3.511157 1.199274
6 0.734714 -2.622911 0.997066
7 0.819559 -1.573956 0.147191
1 3.845283 -3.986629 0.616698
1 3.960735 -2.087698 -1.042384
1 -0.213168 -2.731501 1.545579
6 2.018644 -0.264635 -1.506026
6 1.223760 1.233874 -2.872606
6 3.032398 0.565309 -1.944500
7 2.501416 1.513134 -2.807605
1 4.092154 0.547528 -1.662212
8 0.856367 0.160861 -2.117478
1 0.432453 1.727885 -3.451601
1 1.684074 -4.340435 1.912220
6 -2.317844 1.375247 0.259081
6 -3.450893 1.112266 -0.684640
6 -3.622516 -0.064806 -1.654031
6 -4.588432 0.115624 -0.513546
1 -3.753704 2.062612 -1.157242
1 -3.983383 0.217301 -2.658159
1 -5.608060 0.466715 -0.738742
1 -4.545610 -0.594440 0.326728
6 -2.677868 -1.242334 -1.732102

1 -1.803060 -1.007397 -2.369847
1 -3.201540 -2.115260 -2.179299
8 -2.120207 -1.649994 -0.459716
1 -2.846819 -1.952450 0.129548
1 -1.756075 2.279169 -0.033126
6 -2.125443 0.987754 1.604106
6 0.680319 1.163331 0.934976
6 0.894657 2.354239 0.212604
6 1.543214 0.822911 1.996789
6 1.993132 3.178785 0.526925
1 0.231316 2.642233 -0.617259
6 2.632591 1.657430 2.311567
1 1.385240 -0.102884 2.572280
6 2.865572 2.832043 1.572665
1 2.165157 4.095698 -0.059902
1 3.307477 1.379435 3.137591
1 3.725187 3.477192 1.814104
6 -2.964434 0.075855 2.457635
1 -3.373621 -0.806357 1.933012
1 -2.377525 -0.284067 3.325828
1 -3.831449 0.646541 2.861388
1 -1.473203 1.661998 2.186974
49
TS1_s_trans_PathI
46 -0.823790 -0.181573 -0.017678
6 2.110534 -0.983815 0.774299
6 3.254331 -0.951658 1.603150
6 3.123684 -0.552744 2.936695
6 1.850236 -0.213876 3.425587
6 0.763387 -0.264562 2.546490
7 0.885095 -0.625539 1.248531
1 4.006105 -0.518813 3.593640
1 4.228379 -1.254073 1.193123
1 -0.249913 0.013053 2.877345
6 2.244824 -1.369278 -0.625536
6 1.579091 -2.034304 -2.589958
6 3.298238 -1.344100 -1.521152
7 2.849095 -1.765105 -2.764411
1 4.331146 -1.019640 -1.344973
8 1.143014 -1.826171 -1.316588
1 0.842554 -2.410666 -3.312160
1 1.695246 0.092276 4.470188

6	-2.450577	0.404164	-1.129972
6	-3.742265	0.199692	-0.386844
6	-4.076998	-1.126818	0.267123
6	-3.823785	0.094822	1.128558
1	-4.598259	0.703614	-0.875428
1	-5.129586	-1.446222	0.180759
1	-4.680899	0.558469	1.641330
1	-2.869998	0.156386	1.682201
6	-3.137112	-2.317975	0.204557
1	-3.061648	-2.698853	-0.834525
1	-3.518985	-3.143363	0.840933
8	-1.770373	-2.045583	0.611913
1	-1.735045	-2.049987	1.593272
1	-2.370521	-0.182797	-2.069014
6	-1.808528	1.707298	-1.178253
6	0.121493	1.579516	-0.542703
6	1.046230	1.447546	-1.606836
6	0.432861	2.418246	0.552659
6	2.295987	2.082909	-1.534886
1	0.788583	0.839439	-2.489108
6	1.687314	3.046695	0.620732
1	-0.292518	2.552255	1.369507
6	2.623400	2.874784	-0.417282
1	3.018809	1.956599	-2.356368
1	1.935366	3.673946	1.491874
1	3.605060	3.371130	-0.361874
6	-2.397857	2.867739	-0.417508
1	-2.585413	2.630903	0.646940
1	-1.759280	3.769275	-0.481061
1	-3.381274	3.111027	-0.877836
1	-1.410960	1.971243	-2.170548

PathII

49

TS1_s_cis_PathII_P

46	0.464470	0.248654	-0.759929
6	-2.270874	-1.049076	0.095808
6	-3.678321	-1.153651	0.120170
6	-4.413996	-0.688916	-0.975518
6	-3.729772	-0.153573	-2.080078
6	-2.333367	-0.070575	-2.024139
7	-1.617309	-0.486722	-0.957674
1	-5.512499	-0.758096	-0.974097

1	-4.175752	-1.610379	0.987638
1	-1.755235	0.366147	-2.853760
6	-1.475217	-1.513997	1.227795
6	0.348153	-2.149757	2.247534
6	-1.712897	-1.672144	2.579509
7	-0.538464	-2.067914	3.207113
1	-2.645034	-1.494031	3.129507
8	-0.147772	-1.834674	1.017291
1	1.404398	-2.443928	2.298875
1	-4.265174	0.206421	-2.970529
6	2.388955	0.837950	-0.385898
6	3.026726	-0.382577	0.193885
6	3.126137	-1.678498	-0.633814
6	4.370308	-0.890267	-0.324589
1	2.832514	-0.553250	1.263558
1	2.966593	-2.604940	-0.055640
1	5.080637	-1.303326	0.409090
1	4.827144	-0.317030	-1.148334
6	2.540246	-1.684913	-2.020836
1	2.730845	-2.650510	-2.533349
1	2.957590	-0.872100	-2.644657
8	1.099224	-1.458136	-2.017536
1	0.659103	-2.243873	-1.624873
1	2.812311	1.134141	-1.366824
6	2.079505	2.030170	0.540334
6	0.534092	1.986967	0.584456
6	-0.212900	1.607635	1.746737
6	-0.189105	2.487314	-0.555445
6	-1.597966	1.770470	1.781871
1	0.317335	1.216046	2.627370
6	-1.598602	2.657244	-0.490441
1	0.368314	2.923542	-1.400030
6	-2.292999	2.302018	0.666699
1	-2.155510	1.487696	2.687868
1	-2.132322	3.079328	-1.355537
1	-3.384477	2.437541	0.718396
6	2.758556	2.027771	1.908074
1	3.860905	2.052352	1.789101
1	2.456177	2.920625	2.494089
1	2.509111	1.131364	2.510054
1	2.356237	2.965535	0.012054

49

TS1_s_cis_PathII_R

46	-0.359833	-0.491708	-0.391211
6	1.389298	1.976514	-0.227199
6	2.535099	2.762891	-0.451118
6	3.399517	2.426900	-1.501883
6	3.088606	1.327501	-2.317015
6	1.942949	0.576499	-2.028439
7	1.123805	0.884924	-1.000561
1	4.301352	3.028800	-1.690660
1	2.720353	3.636938	0.189062
1	1.677317	-0.316763	-2.613034
6	0.465439	2.287741	0.866802
6	-1.486758	2.553921	1.818201
6	0.621868	2.603281	2.198833
7	-0.630481	2.759037	2.783503
1	1.557145	2.698929	2.764014
8	-0.899152	2.267478	0.618090
1	-2.584230	2.586722	1.836609
1	3.728797	1.037449	-3.162456
6	-2.184441	-1.687943	0.055557
6	-3.391267	-0.836073	0.220582
6	-3.860429	0.140190	-0.873718
6	-4.630453	-1.123442	-0.614720
1	-3.564385	-0.472997	1.245118
1	-4.293375	1.077522	-0.484072
1	-5.609608	-1.044773	-0.117381
1	-4.546638	-1.931515	-1.360590
6	-2.987094	0.298563	-2.086239
1	-3.425175	1.024006	-2.803383
1	-2.829747	-0.660943	-2.614743
8	-1.655118	0.760980	-1.731102
1	-1.732670	1.621158	-1.260656
1	-2.183353	-2.394405	-0.794480
6	-1.264283	-1.917309	1.090613
6	1.212098	-1.535543	0.261896
6	2.002447	-1.080476	1.333031
6	1.614775	-2.652515	-0.494181
6	3.207115	-1.744071	1.640891
1	1.697507	-0.201846	1.923419
6	2.822562	-3.308312	-0.181118
1	0.993136	-3.020110	-1.326684
6	3.620281	-2.856397	0.885648

1	3.826715	-1.380107	2.476911
1	3.135878	-4.181026	-0.777265
1	4.562808	-3.372072	1.129167
6	-1.321528	-1.301706	2.460977
1	-1.971972	-1.920712	3.120019
1	-0.314536	-1.284276	2.922961
1	-1.720787	-0.269112	2.458836
1	-0.666110	-2.838980	1.014083

49

TS1_s_cis_PathII

46	0.480583	0.484591	-0.600379
6	-1.583194	-1.781696	-0.206802
6	-2.890442	-2.304046	-0.154969
6	-3.875702	-1.754812	-0.985626
6	-3.524647	-0.718540	-1.865127
6	-2.205136	-0.247680	-1.857192
7	-1.259069	-0.748402	-1.034715
1	-4.905262	-2.142642	-0.955677
1	-3.113598	-3.138706	0.524845
1	-1.891964	0.581599	-2.509850
6	-0.544052	-2.298627	0.683322
6	1.490204	-2.836071	1.286814
6	-0.527774	-2.720085	1.996261
7	0.774091	-3.048411	2.358589
1	-1.369302	-2.774095	2.698034
8	0.760935	-2.383751	0.222629
1	2.566954	-2.978142	1.126110
1	-4.260314	-0.267244	-2.546315
6	2.093655	1.614415	0.024983
6	3.150252	0.655677	0.460104
6	3.709493	-0.379745	-0.537794
6	4.565210	0.774585	-0.087851
1	3.054825	0.276000	1.488298
1	3.931256	-1.363534	-0.090004
1	5.401014	0.567628	0.598793
1	4.745983	1.584500	-0.814271
6	3.138737	-0.426759	-1.931821
1	3.657425	-1.194274	-2.542891
1	3.230036	0.547273	-2.448916
8	1.711391	-0.732498	-1.953267
1	1.578350	-1.624881	-1.559932
1	2.347886	2.234895	-0.857029

6	1.149467	2.209709	0.953887
6	-0.776788	1.678283	0.508799
6	-1.472475	1.014410	1.544478
6	-1.450991	2.638006	-0.284725
6	-2.848233	1.238734	1.719710
1	-0.952003	0.284006	2.182164
6	-2.825610	2.861554	-0.099377
1	-0.902498	3.199092	-1.058597
6	-3.529547	2.153737	0.893948
1	-3.392576	0.689364	2.504450
1	-3.349824	3.592037	-0.736303
1	-4.608219	2.326469	1.034579
6	1.252059	1.945650	2.436609
1	2.210873	2.375029	2.802476
1	0.425892	2.429703	2.990873
1	1.254786	0.866031	2.682786
1	0.882772	3.246212	0.696002

Intermediates

49

A1

46	-0.709509	-0.505364	0.021017
6	2.296793	0.192581	0.329742
6	3.375038	0.836865	0.978521
6	3.175314	1.430337	2.228511
6	1.903702	1.364117	2.819125
6	0.883638	0.697781	2.133365
7	1.067808	0.133340	0.917211
1	4.007303	1.941256	2.736447
1	4.356986	0.869118	0.487844
1	-0.126893	0.615882	2.556844
6	2.482506	-0.397618	-0.990829
6	3.648553	-0.889792	-2.766775
6	1.693561	-1.056318	-1.919292
7	2.455731	-1.358699	-3.037938
1	0.628257	-1.316313	-1.835999
8	3.742949	-0.295460	-1.548668
1	4.558842	-0.914405	-3.381116
1	1.694623	1.816560	3.798964
6	-2.869859	-0.574270	-0.366930
6	-2.263509	-1.693627	-0.936552
6	-0.713588	-2.475322	0.673732

6	-1.957528	-2.931895	-0.090651
1	-2.055992	-1.716863	-2.020870
1	0.237806	-2.867091	0.260843
1	-1.762622	-3.817307	-0.726053
1	-2.803162	-3.167775	0.589092
6	-0.780499	-2.609774	2.173849
1	-1.030126	-3.685480	2.386135
1	-1.632334	-2.011300	2.579717
8	0.459938	-2.249804	2.763737
1	0.341220	-2.283216	3.733519
1	-3.285612	-0.644151	0.656283
6	-3.132784	0.728193	-1.116104
6	-1.939216	1.630907	-0.757500
6	-0.783869	1.616483	-1.588171
6	-1.916010	2.435961	0.410520
6	0.336644	2.418793	-1.281908
1	-0.796982	1.026947	-2.519209
6	-0.792970	3.219326	0.715708
1	-2.788442	2.451437	1.081495
6	0.335092	3.217053	-0.130005
1	1.211415	2.404081	-1.950120
1	-0.794847	3.838513	1.626462
1	1.212277	3.834694	0.117168
1	-3.063255	0.518424	-2.203805
6	-4.507328	1.320884	-0.796024
1	-4.651108	2.290427	-1.315427
1	-5.309292	0.629495	-1.126861
1	-4.640631	1.489438	0.292400

49

A2

46	0.159162	-1.127237	-0.326224
6	2.335333	1.035227	-0.395862
6	3.613263	1.541360	-0.722549
6	4.422659	0.843249	-1.623083
6	3.939680	-0.344010	-2.199420
6	2.667990	-0.795355	-1.836467
7	1.892382	-0.136273	-0.941230
1	5.420169	1.228805	-1.882954
1	3.949385	2.483882	-0.269133
1	2.244749	-1.721767	-2.252058
6	1.463932	1.743080	0.531734
6	1.027665	3.171120	2.114578

6	0.106216	1.752968	0.803094
7	-0.143620	2.664760	1.819154
1	-0.696031	1.184724	0.312020
8	2.049177	2.669021	1.371133
1	1.284546	3.928018	2.867496
1	4.535252	-0.921147	-2.920971
6	-1.891124	-1.633957	0.338372
6	-1.143173	-2.800698	0.148998
6	1.043830	-2.638305	1.263038
6	-0.291178	-3.379166	1.277351
1	-1.294782	-3.398875	-0.767210
1	1.886544	-3.090291	0.707645
1	-0.123189	-4.462214	1.124564
1	-0.803537	-3.242713	2.251251
6	1.276897	-1.518743	2.028566
1	0.500155	-1.085173	2.682174
1	-0.513865	-0.632451	-1.629033
8	2.453690	-0.874258	2.153867
1	3.124934	-1.298904	1.571619
1	-1.901931	-1.193865	1.354893
6	-3.020838	-1.170941	-0.565499
6	-3.055564	0.351726	-0.631103
6	-2.690404	1.024870	-1.815531
6	-3.387789	1.119606	0.506317
6	-2.643541	2.429505	-1.861574
1	-2.432953	0.436867	-2.711932
6	-3.347317	2.523753	0.462587
1	-3.671381	0.616196	1.444385
6	-2.968436	3.183883	-0.720042
1	-2.351245	2.937171	-2.794785
1	-3.600435	3.105710	1.362832
1	-2.926541	4.284213	-0.751417
1	-2.831895	-1.558287	-1.587864
6	-4.346153	-1.774402	-0.050878
1	-5.195855	-1.432430	-0.677017
1	-4.307895	-2.882358	-0.088062
1	-4.551588	-1.478339	0.998615

49

A3

46	0.882980	-0.473749	-0.066354
6	-2.182256	-0.900359	-0.034271
6	-3.410517	-1.333535	-0.580123

6	-3.408500	-2.254902	-1.632291
6	-2.181439	-2.742730	-2.110697
6	-1.001147	-2.272793	-1.523914
7	-0.996244	-1.362168	-0.524741
1	-4.358836	-2.595967	-2.070275
1	-4.351477	-0.945728	-0.166655
1	-0.014677	-2.616980	-1.870275
6	-2.151028	0.057894	1.062793
6	-3.009011	1.568622	2.377146
6	-1.206082	0.492591	1.976357
7	-1.772771	1.455210	2.797074
1	-0.157642	0.179937	2.077403
8	-3.314560	0.746386	1.338136
1	-3.810252	2.219586	2.751738
1	-2.129089	-3.473015	-2.930893
6	2.592318	0.767989	0.435973
6	3.054846	-0.263980	-0.394140
6	2.912899	-2.065700	1.333945
6	3.685501	-1.539837	0.124400
1	3.215178	-0.045453	-1.464704
1	3.296487	-3.053365	1.675112
1	4.754113	-1.356592	0.375349
1	3.679572	-2.300272	-0.684436
6	1.445124	-2.201414	0.943660
1	1.279948	-2.943691	0.133282
1	3.008443	-1.371627	2.198506
8	0.572985	-2.487039	1.973485
1	0.852133	-1.974304	2.764617
1	2.706168	0.666879	1.529502
6	2.254488	2.178487	-0.071572
6	0.815303	2.194170	-0.600352
6	0.457937	1.462825	-1.767601
6	-0.190589	2.930925	0.065704
6	-0.877195	1.456081	-2.225905
1	1.232032	0.955781	-2.366263
6	-1.510971	2.938748	-0.408017
1	0.073466	3.501288	0.970362
6	-1.863098	2.184943	-1.544331
1	-1.138017	0.876115	-3.124859
1	-2.277047	3.525382	0.123119
1	-2.905041	2.173512	-1.899706
1	2.274283	2.845612	0.812953

6	3.259218	2.713517	-1.105218
1	3.251275	2.114553	-2.038690
1	3.012129	3.761056	-1.375052
1	4.290681	2.694424	-0.696410

49

I_a_maj

46	0.548756000	0.212666000	-0.597075000
6	-2.364743000	-0.923489000	-0.187101000
6	-3.769816000	-0.898662000	-0.327426000
6	-4.333416000	-0.229575000	-1.418805000
6	-3.484103000	0.378347000	-2.359329000
6	-2.101944000	0.319932000	-2.146632000
7	-1.549913000	-0.296089000	-1.078717000
1	-5.426490000	-0.194974000	-1.542964000
1	-4.399462000	-1.415157000	0.411164000
1	-1.398493000	0.803174000	-2.842752000
6	-1.753112000	-1.600550000	0.951713000
6	-0.127724000	-2.505698000	2.093147000
6	-2.187138000	-1.935727000	2.220272000
7	-1.135896000	-2.503403000	2.928004000
1	-3.178043000	-1.774787000	2.662450000
8	-0.427826000	-1.980240000	0.871414000
1	0.894758000	-2.878576000	2.236759000
1	-3.881297000	0.900302000	-3.241871000
6	2.456673000	0.634191000	0.026843000
6	3.081295000	-0.684903000	0.343636000
6	3.209983000	-1.781038000	-0.730878000
6	4.441182000	-1.077389000	-0.229077000
1	2.852128000	-1.065246000	1.352794000
1	3.036862000	-2.807217000	-0.363565000
1	5.131372000	-1.636541000	0.422342000
1	4.920125000	-0.346092000	-0.901073000
6	2.662609000	-1.494095000	-2.103811000
1	2.867204000	-2.333510000	-2.800007000
1	3.095781000	-0.570957000	-2.533355000
8	1.221690000	-1.262607000	-2.094651000
1	0.766283000	-2.109936000	-1.895107000
1	2.937676000	1.177384000	-0.813522000
6	2.080581000	1.531491000	1.211737000
6	0.563957000	1.709047000	1.013978000
6	-0.382750000	1.276067000	1.997951000
6	0.079014000	2.447623000	-0.122465000

6	-1.732044000	1.603784000	1.878124000
1	-0.018999000	0.694446000	2.859981000
6	-1.300484000	2.780399000	-0.216446000
1	0.791052000	2.919603000	-0.817445000
6	-2.192094000	2.363860000	0.772115000
1	-2.446206000	1.269772000	2.646238000
1	-1.654983000	3.373958000	-1.072921000
1	-3.259230000	2.624412000	0.696655000
1	2.222773000	0.964973000	2.155350000
6	2.852136000	2.851332000	1.291033000
1	2.492657000	3.472595000	2.138511000
1	3.935125000	2.658199000	1.439220000
1	2.737970000	3.441079000	0.358001000

TSA1

49

TSA1_P

46	-0.813804	0.341317	0.088637
6	2.230404	0.743245	0.230454
6	3.464803	1.171024	-0.308049
6	3.483323	2.159419	-1.295673
6	2.268571	2.730335	-1.710801
6	1.081679	2.274860	-1.129895
7	1.055413	1.292184	-0.195773
1	4.439622	2.492087	-1.726954
1	4.399088	0.725953	0.061775
1	0.101892	2.700564	-1.393428
6	2.223183	-0.326675	1.221922
6	1.392068	-1.665259	2.728089
6	3.156956	-1.282594	1.583391
7	2.604037	-2.123966	2.535982
1	4.171073	-1.423527	1.190080
8	1.095204	-0.570166	1.974862
1	0.612190	-2.028041	3.410416
1	2.232259	3.521802	-2.473219
6	-2.715084	-0.736114	0.029819
6	-2.318800	-0.539837	1.358485
6	-1.660615	1.713491	1.390533
6	-2.606134	0.750842	2.120410
1	-1.829070	-1.369544	1.898433

1	-0.815846	2.084133	2.004904
1	-2.347849	0.639185	3.189752
1	-3.672177	1.053936	2.046178
6	-2.378790	2.807193	0.622468
1	-2.937218	3.417754	1.382554
1	-3.152526	2.369758	-0.043000
8	-1.561794	3.637431	-0.193856
1	-0.893827	4.051451	0.391344
1	-3.422182	-0.032207	-0.445632
6	-2.396973	-2.037206	-0.715425
6	-0.925075	-1.950942	-1.139389
6	-0.510467	-1.007243	-2.122608
6	0.060628	-2.769631	-0.532803
6	0.847454	-0.923936	-2.506869
1	-1.260636	-0.390203	-2.643436
6	1.405512	-2.677615	-0.917937
1	-0.246136	-3.492005	0.240141
6	1.803328	-1.754254	-1.907045
1	1.148486	-0.194971	-3.274912
1	2.152975	-3.330521	-0.442008
1	2.861597	-1.682701	-2.201687
1	-2.478352	-2.876813	0.006426
6	-3.354816	-2.282166	-1.886389
1	-3.101085	-3.221324	-2.418844
1	-4.399901	-2.362569	-1.521983
1	-3.317731	-1.450039	-2.619856

49

TSA1_R

46	-0.548608	-0.212492	-0.597238
6	2.364831	0.923680	-0.186436
6	3.769888	0.899185	-0.326927
6	4.333479	0.230703	-1.418695
6	3.484158	-0.376858	-2.359436
6	2.101997	-0.318746	-2.146590
7	1.549997	0.296564	-1.078265
1	5.426550	0.196301	-1.542979
1	4.399478	1.415437	0.411884
1	1.398549	-0.801750	-2.842883
6	1.753145	1.599851	0.952876
6	0.127526	2.503668	2.095039
6	2.186783	1.933091	2.222060
7	1.135380	2.499911	2.930257

1	3.177502	1.771289	2.664353
8	0.427946	1.980008	0.872603
1	-0.894932	2.876549	2.238856
1	3.881328	-0.898313	-3.242289
6	-2.456928	-0.634053	0.025508
6	-3.081041	0.684994	0.343638
6	-3.208548	1.782689	-0.729467
6	-4.440414	1.079179	-0.229105
1	-2.852082	1.063936	1.353373
1	-3.034804	2.808268	-0.360771
1	-5.130349	1.637983	0.422890
1	-4.919764	0.349222	-0.902272
6	-2.660856	1.497123	-2.102560
1	-2.864868	2.337379	-2.797916
1	-3.094264	0.574623	-2.533244
8	-1.220025	1.264962	-2.093159
1	-0.764311	2.111867	-1.892484
1	-2.938013	-1.176110	-0.815544
6	-2.081561	-1.532745	1.209611
6	-0.564827	-1.710209	1.012541
6	-0.079155	-2.448019	-0.124086
6	0.381280	-1.277562	1.997241
6	1.300449	-2.780549	-0.217462
1	-0.790722	-2.919610	-0.819817
6	1.730714	-1.604945	1.877961
1	0.016867	-0.696506	2.859378
6	2.191463	-2.364379	0.771809
1	1.655510	-3.373683	-1.074002
1	2.444408	-1.271211	2.646632
1	3.258673	-2.624771	0.696786
1	-2.224197	-0.967266	2.153785
6	-2.853361	-2.852537	1.287037
1	-2.494460	-3.474848	2.134006
1	-3.936381	-2.659325	1.434962
1	-2.738888	-3.441270	0.353402

49

TSA1

46	-0.758874	0.428024	-0.226823
6	2.260574	0.475433	0.662816
6	3.630125	0.739706	0.431573
6	3.995197	1.728021	-0.485886
6	2.982502	2.446494	-1.144376

6	1.649460	2.128787	-0.867450
7	1.282549	1.159341	0.004946
1	5.057415	1.942785	-0.677917
1	4.391185	0.173936	0.987407
1	0.824512	2.652376	-1.373940
6	1.890054	-0.578329	1.600887
6	0.578340	-1.719510	2.914781
6	2.553412	-1.687278	2.095382
7	1.697078	-2.400359	2.922109
1	3.576579	-2.018933	1.879985
8	0.623879	-0.598641	2.141396
1	-0.359709	-1.919945	3.449111
1	3.214572	3.240896	-1.868290
6	-2.605472	-0.481708	-0.325955
6	-2.594137	-0.199087	1.096700
6	-2.220635	1.485673	1.518702
6	-3.487070	0.749276	1.834581
1	-2.052436	-0.914662	1.736261
1	-1.485409	1.574232	2.335155
1	-3.671373	0.488631	2.888807
1	-4.382721	1.093966	1.290094
6	-2.395309	2.685801	0.611980
1	-2.777133	3.519892	1.250881
1	-3.175347	2.482109	-0.148365
8	-1.230963	3.093289	-0.106253
1	-0.540783	3.317765	0.553241
1	-3.321707	0.079195	-0.956141
6	-2.138878	-1.845767	-0.847916
6	-0.681726	-1.589250	-1.273460
6	-0.399309	-0.600708	-2.280883
6	0.396407	-2.381084	-0.777837
6	0.927492	-0.443463	-2.768422
1	-1.228144	-0.101663	-2.809239
6	1.690156	-2.206010	-1.269391
1	0.188713	-3.136342	-0.003002
6	1.962206	-1.230611	-2.261588
1	1.127491	0.305985	-3.549711
1	2.509484	-2.829585	-0.879435
1	2.989622	-1.101954	-2.635552
1	-2.110535	-2.566621	-0.004773
6	-3.015818	-2.406938	-1.969292
1	-2.614264	-3.374383	-2.336047

1	-4.050971	-2.576802	-1.605925
1	-3.066998	-1.706692	-2.828953

TSA2

49

TSA2_P

46	1.350143	-0.816670	-0.154058
6	-1.452652	-1.595641	0.438017
6	-2.709104	-2.217856	0.296829
6	-3.026598	-2.846294	-0.910647
6	-2.085198	-2.833213	-1.953053
6	-0.848750	-2.214092	-1.744157
7	-0.528809	-1.613700	-0.572243
1	-4.003379	-3.335681	-1.040271
1	-3.419709	-2.199257	1.134878
1	-0.078383	-2.194585	-2.526722
6	-1.105194	-0.873115	1.647510
6	0.246690	0.337112	2.907778
6	-1.755402	-0.443678	2.784592
7	-0.882936	0.315551	3.555439
1	-2.796601	-0.623208	3.076533
8	0.193196	-0.378444	1.731635
1	1.203588	0.810631	3.159791
1	-2.294208	-3.304813	-2.923954
6	0.998851	2.616352	0.026930
6	2.184000	2.073233	-0.319728
6	3.235119	-0.047182	0.606710
6	3.138944	1.475367	0.686693
1	2.481036	2.015567	-1.381416
1	3.390696	-0.579071	1.565191
1	2.814277	1.746861	1.711026
1	4.162233	1.897373	0.556505
6	3.685898	-0.721925	-0.537346
1	3.837789	-0.184947	-1.490695
1	1.844625	-1.115518	-1.562141
8	4.249215	-1.943798	-0.431869
1	4.417884	-2.297600	-1.332352
1	0.731654	2.649528	1.099948
6	-0.064812	3.143767	-0.916017
6	-1.345049	2.322444	-0.705774
6	-1.820206	1.405950	-1.667994
6	-2.076996	2.472056	0.493950
6	-3.008051	0.685165	-1.451543

1 -1.265969 1.253879 -2.605910
6 -3.256937 1.744151 0.717963
1 -1.721165 3.180590 1.260216
6 -3.732865 0.851802 -0.259768
1 -3.364491 -0.017873 -2.220733
1 -3.814017 1.882401 1.658170
1 -4.662960 0.286990 -0.091286
1 -0.309482 4.174806 -0.568764
6 0.389889 3.232321 -2.374812
1 1.266242 3.905601 -2.467235
1 0.685460 2.243968 -2.783398
1 -0.418249 3.635800 -3.018026
49
TSA2_R
46 -1.433921 0.116979 -0.182169
6 1.420501 0.658977 -1.322053
6 2.562829 0.478384 -2.131651
6 2.601465 -0.597939 -3.022604
6 1.494074 -1.460125 -3.101622
6 0.395575 -1.215877 -2.269547
7 0.361377 -0.195539 -1.386050
1 3.486770 -0.760302 -3.655781
1 3.403224 1.182626 -2.053492
1 -0.497878 -1.857984 -2.299632
6 1.354907 1.760450 -0.375662
6 0.409614 3.001232 1.162958
6 2.240366 2.716376 0.083589
7 1.619770 3.484666 1.058922
1 3.283070 2.874905 -0.215547
8 0.172016 1.958755 0.310658
1 -0.421468 3.313042 1.808913
1 1.474905 -2.311750 -3.796817
6 -1.291829 -1.399040 1.342176
6 -2.447494 -1.618325 0.547581
6 -3.266446 0.548510 0.619783
6 -3.739723 -0.879978 0.902692
1 -2.471803 -2.426030 -0.203358
1 -3.100510 1.183993 1.511647
1 -4.050770 -1.053189 1.952433
1 -4.570120 -1.181595 0.234797
6 -3.805019 1.282790 -0.597677
1 -4.394940 2.186396 -0.332643

1	-4.432416	0.620010	-1.227389
8	-2.620415	1.706988	-1.375042
1	-2.763575	1.460980	-2.313309
1	-1.436525	-0.777390	2.244794
6	-0.083889	-2.324755	1.436574
6	1.228301	-1.530850	1.461164
6	2.312687	-1.853918	0.621296
6	1.377194	-0.442867	2.353587
6	3.509948	-1.115391	0.669809
1	2.230299	-2.684279	-0.094406
6	2.570448	0.293014	2.406343
1	0.548744	-0.167601	3.025222
6	3.645467	-0.039551	1.561282
1	4.340050	-1.385264	-0.002106
1	2.658714	1.139400	3.105691
1	4.580231	0.541867	1.595418
1	-0.181120	-2.764832	2.459189
6	-0.102431	-3.498103	0.454975
1	-1.044246	-4.073126	0.556805
1	-0.012822	-3.173306	-0.599642
1	0.732474	-4.195515	0.666003

49

TSA2

46	1.311031	-0.968960	0.080749
6	-1.587684	-1.577979	0.542230
6	-2.874302	-2.125903	0.379402
6	-3.113628	-2.976157	-0.705095
6	-2.062817	-3.263637	-1.592578
6	-0.806037	-2.690438	-1.364658
7	-0.568595	-1.861341	-0.322978
1	-4.112029	-3.413460	-0.855821
1	-3.664547	-1.879498	1.102039
1	0.045040	-2.889571	-2.031566
6	-1.281646	-0.658876	1.625597
6	0.109218	0.505840	2.889456
6	-1.977152	0.113550	2.529401
7	-1.077703	0.833590	3.310868
1	-3.063156	0.199614	2.650035
8	0.070635	-0.413519	1.862470
1	1.102607	0.819742	3.234534
1	-2.206497	-3.927492	-2.457078
6	1.093108	2.608589	0.168950

6	2.268452	2.077631	-0.227124
6	3.216863	-0.189020	0.478005
6	3.174552	1.315883	0.708579
1	2.590306	2.165905	-1.280014
1	3.646593	-0.772146	1.316755
1	2.860363	1.491568	1.758183
1	4.222182	1.693994	0.630107
6	3.429775	-0.774637	-0.816105
1	3.494006	-0.103345	-1.696595
1	2.034375	-1.202247	-1.297896
8	4.173585	-1.917982	-0.879070
1	4.210096	-2.223117	-1.811861
1	0.797451	2.494921	1.229115
6	0.073717	3.317760	-0.695594
6	-1.178953	2.438054	-0.789657
6	-1.197150	1.291642	-1.614489
6	-2.333472	2.755289	-0.046184
6	-2.359319	0.515644	-1.735891
1	-0.294941	1.009748	-2.181016
6	-3.492991	1.963383	-0.147136
1	-2.327739	3.639309	0.612804
6	-3.513505	0.848167	-1.002951
1	-2.361449	-0.366050	-2.396153
1	-4.387842	2.226444	0.439481
1	-4.422710	0.233746	-1.094358
1	-0.232050	4.234994	-0.144912
6	0.600714	3.740923	-2.072489
1	1.471285	4.421281	-1.969748
1	0.922510	2.873859	-2.682257
1	-0.187392	4.276006	-2.640694

TSA3

49

TSA3_P

46	-1.618586	-0.459679	-0.347318
6	0.726989	-2.260762	0.357807
6	1.748585	-3.224747	0.242697
6	2.013999	-3.779864	-1.014173
6	1.255588	-3.360512	-2.120660
6	0.256515	-2.397313	-1.929563
7	-0.006621	-1.854035	-0.720456
1	2.806238	-4.534920	-1.128972
1	2.313531	-3.529523	1.135226

1	-0.357177	-2.034910	-2.767863
6	0.417992	-1.629431	1.632384
6	-0.823602	-0.390059	2.972621
6	1.030425	-1.474014	2.857253
7	0.228074	-0.690233	3.679620
1	2.002645	-1.864311	3.181272
8	-0.787546	-0.937761	1.709194
1	-1.709784	0.204806	3.228839
1	1.431952	-3.769240	-3.126077
6	-0.089143	2.957721	-0.857500
6	-1.373954	2.722376	-0.518358
6	-3.052325	1.026997	-1.367072
6	-2.476241	2.450374	-1.506836
1	-1.653347	2.707853	0.549304
1	-3.858533	0.790824	-2.093295
1	-2.110312	2.588955	-2.544030
1	-3.322799	3.156802	-1.356031
6	-3.361821	0.574807	-0.002356
1	-3.216961	1.259994	0.855028
1	-2.202713	0.263756	-1.834851
8	-4.391906	-0.315363	0.135161
1	-4.540687	-0.475891	1.093594
1	0.177450	2.980895	-1.931469
6	1.064086	3.168418	0.097304
6	2.221005	2.225569	-0.234664
6	3.558202	2.648912	-0.075294
6	1.978605	0.903739	-0.663912
6	4.626429	1.773958	-0.338033
1	3.762085	3.680988	0.256038
6	3.044371	0.028667	-0.937085
1	0.937932	0.560132	-0.792784
6	4.372637	0.459929	-0.772943
1	5.664072	2.122566	-0.210523
1	2.838812	-0.993561	-1.289051
1	5.208496	-0.224509	-0.989022
1	1.446223	4.202490	-0.071911
6	0.685303	3.048577	1.583968
1	1.569957	3.239153	2.224296
1	-0.102182	3.778138	1.864773
1	0.313633	2.029581	1.821622

49

TSA3_R

46	1.641465	0.653506	-0.374469
6	-0.851671	1.922057	0.719332
6	-1.947450	2.801440	0.828364
6	-2.128880	3.786132	-0.146826
6	-1.214766	3.870648	-1.210687
6	-0.144119	2.971808	-1.257657
7	0.040407	2.017166	-0.313555
1	-2.979821	4.480432	-0.080377
1	-2.643929	2.699118	1.672118
1	0.596773	3.004317	-2.068053
6	-0.633380	0.874073	1.700382
6	0.510130	-0.776800	2.614888
6	-1.272925	0.406601	2.829339
7	-0.529470	-0.626437	3.385834
1	-2.215517	0.756184	3.267088
8	0.517570	0.106491	1.558441
1	1.358661	-1.467651	2.695132
1	-1.320049	4.625803	-2.002674
6	0.225170	-2.712351	-1.268093
6	1.425962	-2.497369	-0.692730
6	3.230476	-0.703579	-1.034447
6	2.627817	-2.031332	-1.474130
1	1.557204	-2.654190	0.392664
1	3.878569	-0.219800	-1.786263
1	2.360325	-1.949896	-2.547160
1	3.448945	-2.786516	-1.414506
6	3.496382	-0.355533	0.300360
1	3.146942	-0.985787	1.141135
1	2.249382	1.287529	-1.611090
8	4.473394	0.538311	0.593998
1	4.440629	0.739274	1.555029
1	0.138803	-2.539378	-2.357943
6	-1.057032	-3.118597	-0.584485
6	-2.124713	-2.024977	-0.720639
6	-3.466979	-2.307419	-0.377001
6	-1.812544	-0.726011	-1.174628
6	-4.461359	-1.320842	-0.474033
1	-3.736448	-3.318845	-0.030054
6	-2.810506	0.258526	-1.293424
1	-0.773602	-0.474825	-1.444176
6	-4.137064	-0.030650	-0.936090
1	-5.499847	-1.563182	-0.196046

1	-2.545626	1.258288	-1.670740
1	-4.917466	0.742138	-1.021537
1	-1.453531	-3.999517	-1.143601
6	-0.882320	-3.551490	0.879989
1	-1.842547	-3.905723	1.304807
1	-0.146665	-4.377364	0.967539
1	-0.534234	-2.711206	1.512111

49

TSA3

46	1.621548	0.535240	-0.306414
6	-0.781980	2.199942	0.453487
6	-1.803785	3.166596	0.373705
6	-1.982521	3.864218	-0.825332
6	-1.139103	3.580512	-1.913167
6	-0.135911	2.616149	-1.759735
7	0.046559	1.938960	-0.602667
1	-2.775672	4.622121	-0.911066
1	-2.441968	3.355516	1.248655
1	0.549405	2.365162	-2.581690
6	-0.582065	1.406067	1.654361
6	0.518559	-0.053653	2.892271
6	-1.275065	1.131552	2.813875
7	-0.559482	0.211981	3.572534
1	-2.245657	1.530224	3.132239
8	0.580941	0.645661	1.706660
1	1.363301	-0.715115	3.123381
1	-1.248811	4.100338	-2.875694
6	0.204125	-2.806028	-1.163438
6	1.468280	-2.621141	-0.731787
6	3.196034	-0.804367	-1.132779
6	2.593336	-2.128661	-1.602029
1	1.710279	-2.812926	0.328046
1	3.961160	-0.373874	-1.806052
1	2.251719	-2.019624	-2.650980
1	3.433974	-2.861888	-1.613610
6	3.383148	-0.534687	0.255479
1	3.048934	-1.257401	1.023338
1	2.244524	0.377960	-1.728382
8	4.367665	0.329699	0.633857
1	4.334123	0.434617	1.609939
1	-0.020578	-2.618237	-2.230714
6	-0.980694	-3.205320	-0.315059

6	-2.135336	-2.214183	-0.480163
6	-3.463029	-2.628081	-0.231128
6	-1.908441	-0.869617	-0.841248
6	-4.532131	-1.722846	-0.335050
1	-3.658995	-3.677276	0.046896
6	-2.978038	0.035688	-0.962877
1	-0.878383	-0.526736	-1.034902
6	-4.293511	-0.385616	-0.705386
1	-5.560480	-2.064883	-0.135115
1	-2.783377	1.074636	-1.269866
1	-5.131607	0.323382	-0.798588
1	-1.353357	-4.182389	-0.704191
6	-0.650671	-3.396550	1.176065
1	-1.555011	-3.709132	1.735688
1	0.126754	-4.174211	1.324602
1	-0.287070	-2.450625	1.629522

49

B1

46	0.486299	-0.638073	-0.684360
6	-2.466685	-0.905299	-0.345667
6	-3.697763	-1.356454	0.171856
6	-3.732931	-2.541307	0.913068
6	-2.539759	-3.254170	1.118919
6	-1.351328	-2.747695	0.583711
7	-1.308250	-1.600720	-0.128873
1	-4.684762	-2.905712	1.327602
1	-4.609900	-0.771818	-0.011988
1	-0.394437	-3.268349	0.735174
6	-2.386094	0.332873	-1.102820
6	-1.317702	1.918444	-2.198978
6	-3.257850	1.332424	-1.482778
7	-2.559027	2.315575	-2.171060
1	-4.334458	1.401186	-1.286972
8	-1.136429	0.703291	-1.580877
1	-0.432626	2.385235	-2.648985
1	-2.519278	-4.190955	1.693767
6	1.987114	0.957944	-0.518827
6	2.551196	-0.148832	-1.168809
6	3.106309	-1.398309	-0.473569
6	1.822534	-1.917595	0.170313
1	2.638698	-0.090967	-2.267589
1	3.423664	-2.096416	-1.276142

1	1.511544	-2.936820	-0.131306
1	1.741571	-1.756907	1.265030
6	4.306088	-1.174995	0.461944
1	4.638300	-2.169251	0.849424
1	4.006400	-0.574450	1.344817
8	5.365200	-0.464410	-0.165465
1	5.681304	-1.018986	-0.907957
1	1.682194	1.790123	-1.182738
6	1.989351	1.344488	0.955477
6	0.539540	1.596673	1.375193
6	-0.199908	2.681646	0.855534
6	-0.118514	0.669219	2.212225
6	-1.567493	2.820558	1.148144
1	0.291808	3.414444	0.196014
6	-1.485645	0.806081	2.507121
1	0.449497	-0.181711	2.621480
6	-2.216930	1.878250	1.967059
1	-2.133654	3.663065	0.720862
1	-1.984008	0.062303	3.148758
1	-3.292713	1.979183	2.180550
1	2.357269	0.495200	1.561176
6	2.925275	2.547678	1.172594
1	2.926043	2.848816	2.240280
1	3.963540	2.286559	0.882823
1	2.612207	3.422812	0.566496

49

B2

46	-0.566696	-0.526586	-0.736245
6	-3.409182	-0.304895	0.236363
6	-4.592256	-0.739254	0.867444
6	-4.832126	-2.111812	1.002621
6	-3.897163	-3.024348	0.487208
6	-2.736927	-2.530485	-0.119274
7	-2.485894	-1.204557	-0.217132
1	-5.750558	-2.466843	1.494216
1	-5.315851	0.004934	1.228976
1	-1.964483	-3.202336	-0.521473
6	-3.118367	1.112370	0.040346
6	-3.126220	3.223688	0.558474
6	-2.478374	1.845181	-0.943337
7	-2.490235	3.186004	-0.585839
1	-2.041458	1.485940	-1.883731

8	-3.547831	2.005818	0.997440
1	-3.356422	4.089027	1.194253
1	-4.052048	-4.110472	0.558098
6	1.562438	-0.593203	-1.094383
6	1.115152	0.743704	-1.230544
6	1.038398	1.809302	-0.205749
6	1.063439	3.111851	-0.579564
1	0.994577	1.108869	-2.265276
1	-0.723926	-1.107945	-2.138142
1	0.970137	3.918654	0.164898
1	1.198976	3.398430	-1.635203
6	0.839233	1.416823	1.235499
1	0.579186	2.300624	1.851376
1	1.754179	0.952371	1.658886
8	-0.264593	0.460700	1.281551
1	-0.144388	-0.140341	2.048047
1	1.732941	-1.111741	-2.053991
6	2.281362	-1.242007	0.081855
6	3.675536	-0.643756	0.270088
6	4.175634	-0.435717	1.573312
6	4.495668	-0.313752	-0.830599
6	5.464089	0.088775	1.775263
1	3.542366	-0.688530	2.440127
6	5.783754	0.212132	-0.630888
1	4.126582	-0.464881	-1.857345
6	6.272891	0.415867	0.672282
1	5.835543	0.247069	2.800487
1	6.409544	0.466877	-1.501333
1	7.281153	0.831754	0.827399
1	1.727975	-1.055506	1.023744
6	2.343483	-2.768025	-0.113718
1	1.319969	-3.188996	-0.206107
1	2.908512	-3.025105	-1.034039
1	2.845031	-3.255813	0.746061

49

B3

46	0.821387	0.047645	-0.521126
6	-0.767478	-2.180421	-0.248777
6	-1.803802	-3.100734	-0.052270
6	-3.021607	-2.856577	-0.709601
6	-3.158569	-1.717653	-1.519765
6	-2.083643	-0.822899	-1.636667

7	-0.922315	-1.055890	-1.002028
1	-3.860444	-3.559110	-0.592342
1	-1.652306	-3.988194	0.578100
1	-2.149540	0.100743	-2.229363
6	0.602642	-2.250561	0.283165
6	2.206355	-2.541139	1.718508
6	1.846768	-2.094613	-0.349634
7	2.843147	-2.291973	0.610671
1	2.060256	-2.073053	-1.427348
8	0.840744	-2.544550	1.599882
1	2.613016	-2.739875	2.719339
1	-4.098095	-1.507714	-2.050484
6	0.338807	2.150461	-0.672348
6	1.753702	1.945104	-0.722835
6	2.502806	1.238393	0.270796
6	2.301075	1.254024	1.771558
1	2.240506	2.029075	-1.709355
1	2.668995	2.211642	2.201755
1	1.247129	1.130940	2.079395
1	2.885552	0.433941	2.235318
6	3.904202	0.805879	-0.113631
1	4.116997	-0.163473	0.394000
1	4.607408	1.559249	0.327630
8	4.057080	0.713508	-1.520569
1	4.980104	0.441795	-1.692775
1	-0.121995	2.391055	-1.649464
6	-0.489925	2.647749	0.506933
6	-1.793337	1.865375	0.634759
6	-2.916300	2.141810	-0.172304
6	-1.874977	0.805007	1.563918
6	-4.095755	1.386616	-0.043159
1	-2.875796	2.957773	-0.911030
6	-3.048874	0.044832	1.691201
1	-1.003327	0.577286	2.198935
6	-4.166134	0.335701	0.887572
1	-4.964210	1.617888	-0.680533
1	-3.091468	-0.778004	2.422445
1	-5.088177	-0.258905	0.983870
1	0.077220	2.492489	1.442173
6	-0.697158	4.167449	0.336817
1	-1.208690	4.411547	-0.616773
1	-1.308514	4.570027	1.170790

1	0.280383	4.691741	0.337107
49			
B9			
46	0.222209	-0.835086	-0.923141
6	-2.411622	-0.431775	0.152257
6	-3.682251	0.106668	0.407037
6	-4.320475	0.821416	-0.616797
6	-3.673705	0.981224	-1.852784
6	-2.394084	0.439469	-2.027634
7	-1.780219	-0.248611	-1.042126
1	-5.320352	1.249713	-0.450038
1	-4.159758	-0.048166	1.384712
1	-1.831769	0.561452	-2.964360
6	-1.641557	-1.231061	1.111472
6	-0.770374	-1.773090	3.028637
6	-0.916570	-2.411457	0.989412
7	-0.369666	-2.725820	2.230342
1	-0.857449	-3.088356	0.127384
8	-1.559011	-0.834834	2.422510
1	-0.560000	-1.627739	4.096615
1	-4.145280	1.532666	-2.678639
6	3.264318	1.288216	-0.625402
6	3.038902	0.048776	-1.125168
6	2.337330	-1.092288	-0.480815
6	2.454929	-1.344012	1.010088
1	3.409317	-0.158054	-2.145395
1	2.143571	-2.372913	1.265787
1	3.508008	-1.200422	1.331970
1	1.828494	-0.650213	1.606652
6	1.963145	-2.161482	-1.337946
1	2.157492	-2.093177	-2.422764
1	0.551433	-0.254204	-2.291766
8	1.742986	-3.408632	-0.848311
1	1.465962	-3.990203	-1.588954
1	3.835293	1.982132	-1.268360
6	2.722628	1.875311	0.662500
6	1.218954	2.141322	0.561795
6	0.374253	1.898037	1.665559
6	0.656471	2.706673	-0.605394
6	-0.990619	2.234615	1.616174
1	0.797529	1.449356	2.579407
6	-0.706371	3.041866	-0.657540

1	1.296973	2.890231	-1.482903
6	-1.533817	2.813582	0.456928
1	-1.633723	2.038665	2.487861
1	-1.126618	3.484210	-1.574756
1	-2.601941	3.076238	0.417017
1	2.865230	1.153891	1.491642
6	3.468845	3.170755	1.039123
1	4.555267	2.977126	1.161962
1	3.344412	3.943184	0.250578
1	3.083710	3.590680	1.990723

49

B5

46	0.753507	-0.488800	-0.619984
6	-2.090048	-1.181172	-0.450054
6	-3.286857	-1.672452	0.102479
6	-3.243928	-2.842437	0.871496
6	-2.015816	-3.501231	1.048929
6	-0.861652	-2.938540	0.491085
7	-0.888142	-1.787699	-0.212941
1	-4.165647	-3.246019	1.316822
1	-4.231984	-1.143707	-0.084311
1	0.119239	-3.424232	0.614731
6	-2.034274	0.003923	-1.299457
6	-2.639444	1.949591	-2.056483
6	-1.151765	0.427543	-2.286624
7	-1.553568	1.680224	-2.735736
1	-0.324581	-0.130693	-2.754432
8	-3.000095	0.969781	-1.178615
1	-3.285607	2.835621	-2.105919
1	-1.941450	-4.435120	1.624134
6	1.927963	1.372669	-0.502403
6	2.716115	0.339419	-1.022186
6	3.455344	-0.674412	-0.143498
6	4.495372	-1.488363	-0.913623
1	2.887332	0.302867	-2.112637
1	5.292170	-0.825740	-1.310409
1	4.968294	-2.246448	-0.256814
1	4.029713	-2.014628	-1.773698
6	2.290180	-1.516951	0.359845
1	2.166650	-2.509034	-0.129921
1	3.932890	-0.166720	0.721776
8	2.187576	-1.545412	1.728458

1	1.482873	-2.180340	1.985453
1	1.495639	2.079619	-1.235659
6	1.822482	1.835636	0.941895
6	0.342223	1.843258	1.328297
6	-0.173057	0.833809	2.171683
6	-0.551871	2.799021	0.796947
6	-1.544039	0.776447	2.475609
1	0.515953	0.083512	2.587695
6	-1.919658	2.752427	1.113801
1	-0.177720	3.587727	0.125312
6	-2.422651	1.737608	1.947592
1	-1.925678	-0.025632	3.127280
1	-2.601731	3.509328	0.695618
1	-3.498049	1.694887	2.180987
1	2.329075	1.102686	1.599352
6	2.514566	3.201614	1.106770
1	2.072855	3.968933	0.438058
1	2.421574	3.559975	2.152759
1	3.593870	3.120904	0.862462

TSB1

49

TSB1_P

46	0.506915	0.494999	-0.459998
6	-2.359936	-0.489031	-0.658065
6	-3.353223	-1.492942	-0.685480
6	-2.993183	-2.818364	-0.946323
6	-1.643695	-3.123736	-1.180812
6	-0.705765	-2.087529	-1.137458
7	-1.047383	-0.802554	-0.880188
1	-3.761677	-3.605916	-0.965185
1	-4.399869	-1.217245	-0.499444
1	0.364185	-2.281191	-1.300847
6	-2.715432	0.893872	-0.372374
6	-4.083885	2.481585	0.224287
6	-2.062638	2.115798	-0.366863
7	-2.951700	3.107885	0.017306
1	-1.011100	2.322866	-0.621491
8	-4.020565	1.141931	0.006347
1	-5.052464	2.889062	0.543545
1	-1.307940	-4.149612	-1.388478
6	2.233668	1.501999	0.542254
6	2.353520	1.699711	-0.832025

6	2.769583	0.789516	-1.995934
6	1.368875	0.287697	-2.338551
1	2.056142	2.708141	-1.170951
1	3.181002	1.440307	-2.796705
1	0.809937	0.930928	-3.044030
1	1.277035	-0.789614	-2.571770
6	3.811127	-0.303659	-1.762143
1	4.703404	0.143080	-1.262256
1	4.140323	-0.667544	-2.763811
8	3.267674	-1.382061	-0.999310
1	3.973356	-2.050438	-0.893653
1	1.879359	2.399646	1.084310
6	2.596098	0.347648	1.468009
6	1.262635	-0.340513	1.814095
6	1.154669	-1.753375	1.799766
6	0.100925	0.423783	2.141779
6	-0.066841	-2.381796	2.076878
1	2.041108	-2.353186	1.542922
6	-1.126183	-0.220785	2.415560
1	0.164824	1.518216	2.246467
6	-1.216278	-1.618149	2.373363
1	-0.131427	-3.480973	2.050752
1	-2.011489	0.386785	2.658696
1	-2.175831	-2.118710	2.574382
1	3.196241	-0.398184	0.913762
6	3.358272	0.838360	2.708185
1	2.760898	1.576522	3.283931
1	3.595535	-0.011822	3.380410
1	4.313184	1.322124	2.414335

49

TSB1_R

46	-0.175873	0.130891	-0.405043
6	-2.734186	0.987895	-0.015435
6	-4.004117	1.146000	0.556049
6	-4.971848	0.169651	0.261732
6	-4.642591	-0.901052	-0.585170
6	-3.337288	-0.988054	-1.101004
7	-2.403947	-0.069995	-0.806077
1	-5.983712	0.252433	0.686994
1	-4.228659	2.007728	1.200464
1	-3.027648	-1.817921	-1.753909
6	-1.577332	1.881269	0.144757

6	-0.309656	3.425567	1.003328
6	-0.641994	2.336547	-0.817744
7	0.140473	3.325711	-0.213755
1	-0.682484	2.210650	-1.909218
8	-1.368390	2.600379	1.293922
1	0.042542	4.073194	1.818140
1	-5.383689	-1.671586	-0.843077
6	1.709772	0.536012	0.357429
6	2.536997	1.070616	-0.759213
6	4.083565	0.973435	-0.865807
6	3.206844	0.235109	-1.838400
1	2.176498	2.055137	-1.098819
1	4.521204	1.919472	-1.226642
1	3.130673	0.586339	-2.880134
1	3.180338	-0.858346	-1.711535
6	4.994604	0.246307	0.111904
1	4.757378	0.549481	1.158818
1	6.037539	0.591572	-0.087214
8	4.906252	-1.171608	-0.050710
1	5.318651	-1.575790	0.738448
1	1.465224	1.319874	1.108557
6	1.984337	-0.823185	1.010622
6	0.725397	-1.636648	0.651832
6	0.597481	-2.161776	-0.677731
6	-0.295299	-1.956001	1.599352
6	-0.464886	-3.037435	-1.007957
1	1.407100	-1.987689	-1.403550
6	-1.341912	-2.818153	1.254954
1	-0.234189	-1.548995	2.618993
6	-1.420675	-3.372791	-0.042560
1	-0.523826	-3.456783	-2.024021
1	-2.107771	-3.069571	2.005019
1	-2.239950	-4.064166	-0.293111
1	2.850496	-1.313741	0.522684
6	2.259080	-0.713307	2.509211
1	1.452399	-0.165600	3.040119
1	2.360816	-1.716207	2.974640
1	3.204890	-0.160670	2.682558

49

TSB1

46	0.120290	0.261156	-0.165476
6	-2.719199	0.461822	-0.566863

6	-4.083901	0.136040	-0.544677
6	-4.479779	-1.089071	-1.108137
6	-3.512822	-1.932107	-1.677264
6	-2.164057	-1.540321	-1.640747
7	-1.778632	-0.379887	-1.083594
1	-5.541837	-1.377901	-1.106756
1	-4.815682	0.829688	-0.106579
1	-1.364660	-2.180588	-2.044570
6	-2.125939	1.687001	-0.023894
6	-1.744696	3.303314	1.378466
6	-1.038216	2.450337	-0.456803
7	-0.825455	3.473204	0.467382
1	-0.537296	2.424163	-1.435020
8	-2.579912	2.243031	1.141795
1	-1.933065	3.884155	2.291059
1	-3.789951	-2.894438	-2.131722
6	1.892823	0.868758	0.668088
6	2.373510	1.494465	-0.584847
6	3.587823	1.131100	-1.446009
6	2.240958	0.746871	-1.971751
1	2.107947	2.561358	-0.651895
1	4.022895	2.036452	-1.901264
1	1.789208	1.313116	-2.799692
1	2.010535	-0.330827	-1.943088
6	4.641310	0.088907	-1.098341
1	4.998428	0.239365	-0.052334
1	5.516566	0.270283	-1.765415
8	4.136287	-1.234277	-1.293161
1	4.801678	-1.852966	-0.930939
1	1.667122	1.607309	1.462924
6	2.325339	-0.501892	1.195956
6	1.009794	-1.302525	1.128235
6	0.914031	-2.544566	0.426394
6	-0.138574	-0.848006	1.872674
6	-0.252603	-3.308354	0.489637
1	1.784652	-2.892437	-0.151558
6	-1.314228	-1.645070	1.925974
1	-0.046300	0.019696	2.545337
6	-1.369930	-2.858467	1.238830
1	-0.308113	-4.266643	-0.050198
1	-2.176529	-1.294832	2.514030
1	-2.282976	-3.472553	1.278845

1	3.016031	-0.984856	0.480028
6	2.953241	-0.465600	2.591766
1	2.267285	-0.004837	3.333347
1	3.197066	-1.492138	2.937649
1	3.891947	0.127216	2.583983

TSB2

49

TSB2_P

46	0.222836	-0.812598	-0.364633
6	-2.558448	-0.367610	-0.075773
6	-3.847314	0.169557	-0.192429
6	-4.196672	0.799940	-1.397102
6	-3.253674	0.875137	-2.433948
6	-1.972684	0.340717	-2.237093
7	-1.638344	-0.261828	-1.077975
1	-5.202387	1.227832	-1.524397
1	-4.558236	0.082934	0.641404
1	-1.188392	0.400538	-3.004577
6	-2.026194	-1.051870	1.108461
6	-1.518506	-1.339381	3.202602
6	-1.204385	-2.169937	1.252125
7	-0.893651	-2.315163	2.604953
1	-0.964120	-2.938559	0.503952
8	-2.237712	-0.534769	2.357794
1	-1.542767	-1.079223	4.269326
1	-3.494002	1.358529	-3.391719
6	3.532888	1.122101	-0.207334
6	3.450785	-0.076738	-0.840615
6	2.612905	-1.254916	-0.560802
6	1.969987	-1.553169	0.654660
1	4.099592	-0.208491	-1.725069
1	0.903556	-0.293577	-1.628917
1	1.713722	-2.607818	0.850819
1	2.101136	-0.912482	1.539596
6	2.707303	-2.348634	-1.624233
1	3.678615	-2.872180	-1.468408
1	2.769228	-1.866584	-2.628531
8	1.689924	-3.324511	-1.555072
1	0.836798	-2.819593	-1.554550
1	4.266153	1.834839	-0.626891
6	2.709019	1.646071	0.948065

6	1.280525	1.995344	0.525280
6	0.202914	1.785725	1.415107
6	1.022334	2.631698	-0.710408
6	-1.089329	2.243295	1.097172
1	0.390660	1.285096	2.379556
6	-0.273165	3.062976	-1.038892
1	1.848080	2.794195	-1.421285
6	-1.331639	2.881697	-0.129924
1	-1.914369	2.086140	1.808126
1	-0.456762	3.552671	-2.008537
1	-2.345176	3.229159	-0.382393
1	2.633550	0.864517	1.732581
6	3.384703	2.875587	1.587360
1	4.407160	2.623393	1.938916
1	3.466434	3.705640	0.854318
1	2.799199	3.240153	2.455505

49

TSB2_R

46	0.157924	-0.815929	-0.210093
6	-2.531410	-0.345905	-0.038694
6	-3.829174	0.178718	-0.016554
6	-4.272226	0.856050	-1.165500
6	-3.413987	0.989066	-2.269740
6	-2.114876	0.460260	-2.197273
7	-1.692473	-0.187826	-1.098621
1	-5.287781	1.278782	-1.196711
1	-4.470533	0.051308	0.866759
1	-1.392094	0.565004	-3.020575
6	-1.845499	-1.086725	1.030830
6	-1.227000	-1.634856	3.041204
6	-1.074593	-2.274426	0.993371
7	-0.696660	-2.571075	2.313559
1	-1.057593	-3.042076	0.206334
8	-1.956667	-0.705768	2.341232
1	-1.162439	-1.483055	4.127523
1	-3.737309	1.514040	-3.180218
6	3.583534	1.154137	-0.205777
6	3.464796	0.012528	-0.918294
6	2.429318	-1.078050	-0.798864
6	1.935911	-1.509290	0.530011
1	4.186529	-0.155566	-1.737366
1	1.468336	-0.509093	-1.350120

1	1.871710	-2.598709	0.694371
1	2.248493	-0.944832	1.424748
6	2.675446	-2.235490	-1.800139
1	3.611474	-2.750469	-1.497109
1	2.843016	-1.801334	-2.813155
8	1.648237	-3.208188	-1.797177
1	0.811614	-2.748962	-2.036991
1	4.425557	1.812823	-0.485920
6	2.704194	1.660415	0.921784
6	1.265566	1.962154	0.502672
6	0.212227	1.790345	1.431764
6	0.973956	2.564960	-0.742774
6	-1.080891	2.268504	1.149395
1	0.426104	1.316595	2.403959
6	-0.321106	3.025539	-1.030928
1	1.779074	2.691312	-1.484297
6	-1.348817	2.894458	-0.078476
1	-1.883637	2.142613	1.891930
1	-0.528523	3.499716	-2.003397
1	-2.360935	3.265607	-0.300843
1	2.653472	0.884237	1.715294
6	3.321556	2.921041	1.563210
1	4.347719	2.710277	1.930731
1	3.382112	3.747935	0.824672
1	2.710928	3.269920	2.420291

49

TSB2

46	0.202502	-0.856073	-0.265799
6	-2.523671	-0.355072	-0.080662
6	-3.811816	0.189188	-0.152604
6	-4.179345	0.843715	-1.340094
6	-3.257398	0.936101	-2.395227
6	-1.974399	0.390514	-2.235629
7	-1.624492	-0.235963	-1.097498
1	-5.184894	1.279489	-1.439625
1	-4.505605	0.092549	0.694334
1	-1.203795	0.462227	-3.017178
6	-1.937500	-1.079331	1.056503
6	-1.379641	-1.500049	3.113830
6	-1.161536	-2.246967	1.114703
7	-0.819998	-2.471320	2.452971
1	-1.039725	-3.019839	0.343070

8	-2.088271	-0.620555	2.336801
1	-1.355295	-1.290350	4.191885
1	-3.517156	1.442475	-3.336004
6	3.509147	1.187960	-0.231931
6	3.412857	0.017206	-0.907500
6	2.523124	-1.150222	-0.677087
6	1.990085	-1.537312	0.596761
1	4.069646	-0.107549	-1.786758
1	1.210361	-0.411330	-1.391944
1	1.847568	-2.617139	0.770842
1	2.205561	-0.935708	1.493736
6	2.678095	-2.249630	-1.738357
1	3.665067	-2.731810	-1.558968
1	2.731106	-1.773752	-2.745621
8	1.699586	-3.263497	-1.668413
1	0.823828	-2.804833	-1.695557
1	4.268072	1.897638	-0.608442
6	2.676718	1.680367	0.931930
6	1.239189	2.010902	0.528120
6	0.182633	1.807179	1.444319
6	0.951426	2.635978	-0.706436
6	-1.116893	2.261699	1.154052
1	0.393651	1.316646	2.408924
6	-0.350338	3.070033	-1.004637
1	1.760484	2.792909	-1.437644
6	-1.386653	2.896815	-0.068952
1	-1.925265	2.107434	1.884734
1	-0.556167	3.555885	-1.971734
1	-2.405088	3.245762	-0.298577
1	2.621982	0.886669	1.706098
6	3.332455	2.912904	1.586809
1	4.360968	2.674017	1.929905
1	3.396007	3.754618	0.865399
1	2.745277	3.254877	2.463004

TSB3

49

TSB3_P

46	-0.353097	-0.689385	-0.751438
6	2.050974	-1.277958	0.395898
6	3.344486	-1.317347	0.931477
6	4.411480	-1.028633	0.064221

6	4.150508	-0.708066	-1.278577
6	2.820311	-0.657037	-1.725577
7	1.799638	-0.935337	-0.896562
1	5.446406	-1.056489	0.437356
1	3.509520	-1.580740	1.985617
1	2.560270	-0.377886	-2.758135
6	0.782003	-1.587802	1.072649
6	-0.686570	-1.736446	2.667740
6	-0.272423	-2.452432	0.682903
7	-1.182030	-2.510051	1.747267
1	-0.264407	-3.210043	-0.114223
8	0.517530	-1.160413	2.347217
1	-1.100589	-1.485566	3.653792
1	4.968391	-0.479567	-1.977140
6	-2.298102	1.547760	1.114092
6	-2.632621	0.305413	0.660023
6	-2.407352	-0.358378	-0.634821
6	-2.095630	0.353439	-1.911961
1	-3.105565	-0.356776	1.406726
1	-0.954657	0.243837	-2.221547
1	-2.594442	-0.105362	-2.786673
1	-2.219339	1.449821	-1.908981
6	-3.201076	-1.655800	-0.813445
1	-3.142648	-2.260773	0.119508
1	-2.764837	-2.254986	-1.638907
8	-4.552439	-1.379113	-1.184869
1	-4.970732	-0.940311	-0.415063
1	-2.550196	1.751685	2.170804
6	-1.603450	2.677808	0.386303
6	-0.103093	2.430939	0.223321
6	0.537437	2.700402	-1.005598
6	0.683605	2.021142	1.322108
6	1.932599	2.581372	-1.128795
1	-0.067216	3.020226	-1.869621
6	2.077625	1.900079	1.199732
1	0.197934	1.796983	2.284417
6	2.707871	2.188086	-0.024048
1	2.417267	2.797134	-2.094351
1	2.677057	1.582749	2.067428
1	3.800770	2.098048	-0.118511
1	-2.028206	2.768543	-0.635611
6	-1.846841	4.017845	1.108981

1	-1.431753	3.989979	2.138638
1	-1.361825	4.854051	0.565794
1	-2.933194	4.234734	1.181382

49

TSB3_R

46	-0.457551	-0.310253	-1.107657
6	2.229154	-1.019757	0.234356
6	3.638111	-1.044904	0.313656
6	4.398146	-0.776399	-0.828953
6	3.738499	-0.505974	-2.038723
6	2.340467	-0.496207	-2.051841
7	1.601901	-0.726105	-0.940942
1	5.497417	-0.787613	-0.777951
1	4.118362	-1.284334	1.272457
1	1.776604	-0.274595	-2.969754
6	1.411236	-1.289653	1.410558
6	0.989216	-1.335153	3.546091
6	0.146289	-1.797498	1.635334
7	-0.102742	-1.810963	3.000525
1	-0.572472	-2.168579	0.897387
8	1.957872	-1.009339	2.647033
1	1.218837	-1.170229	4.607238
1	4.291285	-0.295344	-2.965265
6	-2.552880	0.907183	1.343646
6	-2.771788	-0.301992	0.762694
6	-2.589658	-0.732246	-0.638667
6	-2.446316	0.109053	-1.768580
1	-3.078430	-1.114275	1.445016
1	-0.533813	-1.622666	-1.873481
1	-2.604562	-0.337270	-2.764918
1	-2.595846	1.196319	-1.716650
6	-2.952151	-2.200738	-0.881834
1	-2.406323	-2.865194	-0.173472
1	-2.666950	-2.494544	-1.912094
8	-4.363355	-2.365469	-0.776143
1	-4.589272	-2.375281	0.176729
1	-2.724637	0.965198	2.433346
6	-2.042777	2.171630	0.686586
6	-0.560130	2.063104	0.313408
6	-0.104984	2.402786	-0.990047
6	0.410653	1.797753	1.315681
6	1.276332	2.541955	-1.257134

1	-0.838497	2.660044	-1.770467
6	1.777109	1.943254	1.044338
1	0.080432	1.514992	2.328057
6	2.214500	2.327676	-0.239776
1	1.605400	2.826156	-2.268493
1	2.512796	1.757321	1.841635
1	3.289817	2.444488	-0.443826
1	-2.607584	2.343347	-0.252627
6	-2.275816	3.398808	1.588669
1	-1.738874	3.291799	2.554129
1	-1.918883	4.325679	1.095903
1	-3.357010	3.518082	1.808324

49

TSB3

46	-0.351521	-0.650233	-0.948269
6	2.018327	-1.316865	0.446330
6	3.338668	-1.345382	0.916126
6	4.368644	-1.065193	0.004987
6	4.047921	-0.766927	-1.328998
6	2.701764	-0.728055	-1.718972
7	1.712851	-0.992596	-0.841785
1	5.417833	-1.083365	0.335912
1	3.547644	-1.596687	1.965396
1	2.396328	-0.465981	-2.742131
6	0.819684	-1.627149	1.233513
6	-0.571824	-1.590883	2.901382
6	-0.288453	-2.428065	0.948356
7	-1.155802	-2.375589	2.037276
1	-0.433677	-3.115373	0.103363
8	0.642838	-1.110761	2.489266
1	-0.918011	-1.270431	3.893292
1	4.830968	-0.545671	-2.068250
6	-2.226560	1.444782	1.159626
6	-2.599376	0.237469	0.649114
6	-2.497031	-0.328975	-0.709105
6	-2.058226	0.347359	-1.880189
1	-3.021175	-0.474493	1.379461
1	-0.323535	-0.101135	-2.384986
1	-2.348116	-0.084143	-2.852764
1	-1.857316	1.427406	-1.899479
6	-3.275562	-1.633164	-0.904163
1	-3.086593	-2.321126	-0.048535

1	-2.939776	-2.137924	-1.831944
8	-4.667514	-1.360150	-1.059150
1	-4.997285	-1.058800	-0.187175
1	-2.398719	1.573620	2.244038
6	-1.592749	2.627446	0.462580
6	-0.088901	2.427375	0.259768
6	0.521464	2.752960	-0.970656
6	0.727024	1.980148	1.321615
6	1.912123	2.636835	-1.136581
1	-0.104964	3.104555	-1.806472
6	2.117513	1.863824	1.158116
1	0.268751	1.719548	2.288184
6	2.715695	2.194530	-0.071049
1	2.370321	2.890446	-2.105835
1	2.739157	1.516210	1.997877
1	3.805313	2.104936	-0.199252
1	-2.050783	2.742321	-0.542014
6	-1.862556	3.927046	1.246383
1	-1.410043	3.877367	2.259144
1	-1.430584	4.801921	0.719399
1	-2.953232	4.097416	1.364699

TSB4

49

TSB4_P

46	0.218883	-0.750836	-0.877425
6	-2.592950	-0.151322	-0.031084
6	-3.888701	0.403619	-0.058397
6	-4.281938	1.158967	-1.166499
6	-3.375798	1.343289	-2.224985
6	-2.107928	0.761356	-2.137638
7	-1.721314	0.026930	-1.067161
1	-5.288375	1.602127	-1.203616
1	-4.566862	0.239209	0.790519
1	-1.366492	0.878479	-2.939449
6	-2.128428	-0.903182	1.121235
6	-0.648396	-2.048769	2.283817
6	-2.620571	-1.200005	2.374599
7	-1.667956	-1.920246	3.084035
1	-3.592873	-0.927718	2.802359
8	-0.855744	-1.459291	1.056155
1	0.307701	-2.562346	2.438008

1	-3.641205	1.931456	-3.114991
6	3.442993	0.966839	-0.668243
6	3.082706	-0.228739	-1.195845
6	2.300953	-1.312711	-0.540900
6	2.549392	-1.662808	0.913802
1	3.375601	-0.434996	-2.241370
1	3.644404	-1.689128	1.098240
1	2.118435	-0.926745	1.618160
1	2.142000	-2.661199	1.158050
6	1.712193	-2.287159	-1.391904
1	1.849809	-2.231036	-2.486176
1	0.637417	-0.157196	-2.206539
8	1.325275	-3.491834	-0.895024
1	0.910687	-4.009786	-1.618424
1	4.041354	1.633139	-1.315261
6	3.029172	1.540010	0.675090
6	1.530153	1.848225	0.724635
6	0.906043	2.583375	-0.309535
6	0.755161	1.474493	1.843439
6	-0.444896	2.955788	-0.213328
1	1.490586	2.873356	-1.197671
6	-0.600477	1.837896	1.938206
1	1.227194	0.903352	2.659780
6	-1.203128	2.587929	0.913263
1	-0.912347	3.533109	-1.026550
1	-1.186138	1.536316	2.820718
1	-2.263098	2.876367	0.986119
1	3.228135	0.793785	1.470851
6	3.846437	2.802788	1.012023
1	3.564268	3.205786	2.005901
1	4.932982	2.574602	1.026792
1	3.674881	3.600426	0.258414

49

TSB4_R

46	0.524330	-0.270554	-0.535986
6	-2.623292	0.024438	-0.386882
6	-3.864540	0.637799	-0.669855
6	-3.924589	1.651347	-1.630171
6	-2.748559	2.017861	-2.307245
6	-1.555363	1.370279	-1.971898
7	-1.482339	0.411243	-1.020664
1	-4.882784	2.142149	-1.859000

1	-4.767893	0.301773	-0.141179
1	-0.607264	1.632338	-2.465304
6	-2.548327	-1.017652	0.628958
6	-1.652933	-2.674578	1.727326
6	-3.349665	-1.371055	1.699710
7	-2.755949	-2.420895	2.385958
1	-4.292438	-0.912722	2.022591
8	-1.464370	-1.868500	0.645417
1	-0.887787	-3.438132	1.919016
1	-2.747588	2.797506	-3.082639
6	3.675929	0.820659	-0.320491
6	3.358812	-0.263601	-1.066416
6	2.344352	-1.285293	-0.670856
6	2.634518	-2.164526	0.529714
1	3.860909	-0.406979	-2.040016
1	3.377722	-2.943279	0.244026
1	3.064416	-1.590031	1.370137
1	1.726555	-2.692234	0.880461
6	1.632445	-1.941530	-1.803049
1	2.111386	-1.748889	-2.790089
1	0.616344	-1.270126	-2.008052
8	1.281259	-3.269587	-1.593306
1	0.804077	-3.584499	-2.389118
1	4.459502	1.508715	-0.682018
6	2.992571	1.156907	0.987593
6	1.466296	1.308911	0.880185
6	0.915470	2.459719	0.223802
6	0.608665	0.578411	1.767267
6	-0.370252	2.913026	0.530243
1	1.548726	3.025089	-0.478191
6	-0.687742	1.063289	2.082134
1	1.028206	-0.263341	2.340834
6	-1.165025	2.233050	1.484056
1	-0.762880	3.813524	0.033114
1	-1.300902	0.519202	2.816084
1	-2.165175	2.615471	1.739488
1	3.157703	0.311800	1.690011
6	3.595644	2.414632	1.641250
1	3.092316	2.647026	2.601643
1	4.674905	2.260460	1.847840
1	3.499148	3.300381	0.979517

49

TSB4

46	0.529988	-1.098122	-0.024412
6	-2.382912	-0.489123	0.586483
6	-3.783100	-0.457789	0.421665
6	-4.367343	-1.250355	-0.570527
6	-3.542802	-2.058750	-1.372659
6	-2.162268	-2.045493	-1.148153
7	-1.588052	-1.278159	-0.193163
1	-5.457294	-1.235143	-0.721148
1	-4.392456	0.194961	1.062431
1	-1.477926	-2.661318	-1.749585
6	-1.724854	0.354229	1.569717
6	0.034748	1.242401	2.550733
6	-2.114270	1.275072	2.521306
7	-0.986771	1.819043	3.122507
1	-3.132140	1.578337	2.793699
8	-0.338675	0.322892	1.600719
1	1.110159	1.366305	2.730280
1	-3.958731	-2.696334	-2.166000
6	2.963196	1.067523	-1.304986
6	3.144740	-0.251789	-1.052860
6	2.636851	-1.042909	0.106053
6	3.075956	-0.667142	1.507208
1	3.716695	-0.830520	-1.801250
1	4.143999	-0.951127	1.645720
1	3.009109	0.424939	1.670962
1	2.490366	-1.189429	2.288372
6	2.375339	-2.445685	-0.186034
1	2.724825	-2.814449	-1.172664
1	1.041094	-2.433976	-0.760976
8	2.467005	-3.350302	0.838137
1	2.310082	-4.248546	0.476318
1	3.421992	1.465483	-2.228795
6	2.110044	2.043007	-0.530910
6	0.633424	1.976604	-0.951470
6	0.172215	1.128533	-1.984339
6	-0.313842	2.780546	-0.275592
6	-1.191641	1.097913	-2.340502
1	0.888000	0.506554	-2.540832
6	-1.674913	2.731153	-0.613491
1	0.017498	3.441472	0.540986
6	-2.121022	1.890550	-1.652961

1	-1.523723	0.434807	-3.154755
1	-2.395960	3.352387	-0.059210
1	-3.188854	1.854696	-1.918472
1	2.134883	1.771985	0.546591
6	2.675845	3.472070	-0.660523
1	2.089295	4.198278	-0.063365
1	3.725116	3.507443	-0.301225
1	2.661315	3.806493	-1.719354

TSB5

49

TSB5_P

46	0.428048	0.072843	-0.892864
6	-2.515443	-0.643486	-0.600059
6	-3.696556	-1.407604	-0.507243
6	-3.614946	-2.796802	-0.651669
6	-2.361865	-3.388333	-0.889140
6	-1.233490	-2.564232	-0.975657
7	-1.301421	-1.222563	-0.832216
1	-4.523241	-3.413973	-0.579547
1	-4.658169	-0.908160	-0.321825
1	-0.231399	-2.981290	-1.157846
6	-2.527284	0.799262	-0.411086
6	-1.559627	2.778291	-0.278563
6	-3.438803	1.763091	-0.031706
7	-2.803799	2.996855	0.043295
1	-4.501165	1.632777	0.206510
8	-1.318612	1.461306	-0.592570
1	-0.710354	3.468884	-0.356655
1	-2.253431	-4.476097	-1.008068
6	3.351406	-0.755433	1.165344
6	3.634834	-0.246930	-0.052159
6	2.722272	-0.061012	-1.244112
6	3.360997	-0.456633	-2.582107
1	4.654725	0.137483	-0.236610
1	3.713408	-1.505984	-2.544267
1	2.646217	-0.353225	-3.419869
1	4.233481	0.199971	-2.781565
6	2.047938	1.269143	-1.217828
1	2.203625	1.917899	-0.333619
1	1.843858	-0.916846	-1.114599
8	1.926632	1.931962	-2.410642

1	1.598514	2.839796	-2.227134
1	4.174850	-0.739685	1.902566
6	2.044214	-1.346913	1.659191
6	0.970968	-0.300186	1.984021
6	-0.370562	-0.718617	2.156204
6	1.275255	1.064244	2.189260
6	-1.375630	0.198094	2.502037
1	-0.627310	-1.779545	2.006962
6	0.269152	1.985689	2.530496
1	2.313452	1.410113	2.067381
6	-1.060470	1.557912	2.686375
1	-2.413636	-0.149604	2.621216
1	0.528471	3.046812	2.674284
1	-1.850182	2.279723	2.945927
1	1.618569	-1.981657	0.848919
6	2.298438	-2.258152	2.877782
1	2.706362	-1.671265	3.727832
1	1.362324	-2.745673	3.216822
1	3.028412	-3.055806	2.626807

49

TSB5_R

46	0.539244	-0.809762	-0.596354
6	-2.432882	-0.818020	-0.220714
6	-3.703600	-1.222085	0.236514
6	-3.812408	-2.379467	1.012842
6	-2.650925	-3.110100	1.315966
6	-1.421168	-2.659942	0.826593
7	-1.308092	-1.538423	0.074445
1	-4.795783	-2.706687	1.382440
1	-4.587912	-0.619081	-0.013707
1	-0.487955	-3.199279	1.037742
6	-2.268176	0.398712	-0.995224
6	-1.078419	1.970081	-1.982926
6	-3.091254	1.372339	-1.522239
7	-2.316734	2.345134	-2.139670
1	-4.185690	1.426730	-1.483337
8	-0.967647	0.776076	-1.306929
1	-0.148736	2.449275	-2.312728
1	-2.687891	-4.024042	1.925845
6	3.077247	0.172600	1.581469
6	3.490037	-0.209017	0.352205
6	2.772687	-0.741657	-0.847748

6	3.398838	-1.986907	-1.459910
1	4.576793	-0.127791	0.149713
1	3.537367	-2.779050	-0.698773
1	2.793877	-2.393288	-2.291328
1	4.402704	-1.723059	-1.861134
6	2.130812	0.183911	-1.704706
1	2.022870	1.241740	-1.399386
1	1.276391	-2.043111	-0.109564
8	2.015742	-0.081301	-3.035594
1	1.530209	0.656112	-3.465515
1	3.870578	0.566759	2.242971
6	1.698474	0.111946	2.204042
6	0.740205	1.207064	1.728335
6	-0.640242	1.075066	2.005043
6	1.181637	2.367321	1.057747
6	-1.554036	2.066254	1.614039
1	-1.001811	0.175615	2.528883
6	0.268742	3.361756	0.663581
1	2.252513	2.486871	0.831162
6	-1.102599	3.215181	0.937202
1	-2.625964	1.938999	1.832408
1	0.632482	4.256636	0.133531
1	-1.818469	3.988158	0.617365
1	1.228502	-0.851533	1.898333
6	1.810104	0.118565	3.743037
1	2.255100	1.071143	4.101147
1	0.814250	0.007106	4.217332
1	2.451941	-0.715939	4.094770
49			
TSB5			
46	-0.485103	0.419034	-0.828580
6	2.476457	0.769851	-0.369492
6	3.698327	1.381247	-0.022245
6	3.697550	2.723576	0.369377
6	2.480426	3.425987	0.402215
6	1.305601	2.759979	0.037351
7	1.297216	1.460139	-0.339158
1	4.639994	3.219097	0.647282
1	4.628740	0.797053	-0.059592
1	0.330823	3.267527	0.047016
6	2.410532	-0.633868	-0.738147
6	1.338187	-2.476217	-1.306865

6	3.283977	-1.698150	-0.825563
7	2.583313	-2.841727	-1.186116
1	4.363942	-1.703836	-0.636128
8	1.156336	-1.132768	-1.067714
1	0.451251	-3.060276	-1.583235
1	2.433809	4.481953	0.704720
6	-3.210983	0.373294	1.426962
6	-3.536470	0.347492	0.116285
6	-2.716281	0.467235	-1.133915
6	-3.282001	1.422369	-2.179655
1	-4.605149	0.194717	-0.131328
1	-3.474545	2.421588	-1.742955
1	-2.605246	1.536623	-3.046442
1	-4.249755	1.015928	-2.547065
6	-2.089836	-0.739866	-1.591061
1	-2.122379	-1.646077	-0.957911
1	-1.492100	1.577813	-0.670966
8	-1.939910	-0.947119	-2.933139
1	-1.529781	-1.828873	-3.068976
1	-4.053542	0.206269	2.122623
6	-1.872134	0.625940	2.093434
6	-0.873544	-0.529195	1.976642
6	0.492998	-0.282919	2.248333
6	-1.268580	-1.850531	1.675683
6	1.438424	-1.319089	2.199317
1	0.817539	0.741315	2.492183
6	-0.323019	-2.889905	1.620181
1	-2.329062	-2.063768	1.469503
6	1.034325	-2.628996	1.877996
1	2.498141	-1.102208	2.405672
1	-0.650059	-3.912096	1.370872
1	1.776094	-3.440986	1.825699
1	-1.392273	1.494848	1.588005
6	-2.083541	1.006402	3.574330
1	-2.542247	0.164473	4.134959
1	-1.122082	1.259755	4.064665
1	-2.754999	1.885974	3.662854

R = H

63

TS_F1

Pd 0.21159380143638 0.36789827470202 -0.01628716554438

C	2.64911887625315	-1.08448255123743	1.28809558644351
C	3.48678969029974	-2.19593273600789	1.52752043262559
C	3.01016998414599	-3.48415400625678	1.26956884790498
C	1.69382868588406	-3.64210540793556	0.80417887799733
C	0.92129542929848	-2.49735030644927	0.58622711355845
N	1.38783420350900	-1.24674307799293	0.79981869305699
H	3.65189878699453	-4.35914829025967	1.44983969020920
H	4.49358484516269	-2.03644729209648	1.93742151651327
H	-0.11365815153380	-2.56835761655370	0.21963869775720
C	3.13380266228851	0.26434143192198	1.54997714494696
C	2.98248150255970	2.40321823248121	1.94224014126679
C	4.38983270410359	0.84368268555448	1.57872995565494
N	4.26560146373214	2.20023121948742	1.81948149397684
H	5.36399294247817	0.37140402104610	1.40603045265004
O	2.22310406495568	1.27243557542688	1.79579324923172
H	2.44586384805762	3.33434119469354	2.15972566458705
H	1.26707465015342	-4.63581867546973	0.61008019221360
C	-0.98492597853640	1.80613197783621	-0.89971607991358
C	-2.28802730224158	1.10472684451393	-1.06067374524617
C	-3.01723218137416	0.53528011782425	0.17355892300398
C	-3.64906662621308	1.65523130548792	-0.63040129385256
H	-2.33021981642591	0.45145258448396	-1.94730921313024
H	-4.47370805407512	1.30979116886989	-1.27800664471608
C	-2.43825313926544	0.87536734924786	1.53115014247680
H	-3.12097694352958	0.52032699157196	2.32790300028982
H	-2.28246005459344	1.96295023020961	1.66483044578782
O	-1.16887868157968	0.17467217452397	1.66927999508389
H	-0.78113067843748	0.35786429081543	2.54721833200302
H	-0.95192777476646	2.65611595227116	-0.19027992282635
C	-0.01025141915566	1.84627667964091	-1.96072999485212
C	1.41415554744763	0.35809681790111	-1.67741020679188
C	1.19242611332878	-0.76301317684617	-2.51396026036086
C	2.72542381299535	0.85498663574677	-1.51277932531073
C	2.27822350644065	-1.42123993766784	-3.10883552960542
H	0.16750822217862	-1.12754030084777	-2.69009342557970
C	3.80837204038831	0.19280392105856	-2.11281377588747
H	2.90780257924125	1.75557202378514	-0.90828213086252
C	3.58862243544636	-0.94867174928185	-2.90295467741404
H	2.10151714897825	-2.30124408069990	-3.74575613491421
H	4.82914314930715	0.57627947328515	-1.96372930282137
H	4.43833861808710	-1.45996635899944	-3.37953983736831
H	-0.31844443817026	1.35811252115069	-2.89985281019624

C	0.82528251735065	3.08943379580743	-2.14006306876343
H	1.66807620472628	2.93266751050179	-2.83737633114041
H	0.17324960466644	3.88516226488797	-2.56100027379984
H	1.22258808741375	3.46251062631337	-1.17569178086734
C	-3.86433429097336	3.03521781502852	-0.02912128516026
H	-4.30868143124001	3.70534521455057	-0.80091437398869
H	-2.90067935134006	3.50078077012454	0.26136026777909
O	-4.64256489749928	2.98732158522502	1.15879926062593
H	-5.55942993836066	2.76466198250050	0.91139873974763
C	-3.56257387336151	-0.86370372044688	0.07005095944981
C	-2.81593424698228	-1.88975992339348	-0.54176387825481
C	-4.81393185749910	-1.17449222761048	0.63886855866948
C	-3.30876008147176	-3.20396727346093	-0.58726026568823
H	-1.83862324854907	-1.64473195303755	-0.99029610808463
C	-5.30900376963724	-2.48705295513188	0.59619094464483
H	-5.40270497330167	-0.37368696177960	1.11471227109544
C	-4.55661558833810	-3.50474694135753	-0.01603443320402
H	-2.72078154133759	-3.99637411184741	-1.07533534256008
H	-6.28978754136496	-2.71788416804413	1.03860859905698
H	-4.94731322005471	-4.53265435026560	-0.05292194450303

63

TS_F2

Pd	-0.49045092636550	-0.05051668772716	-0.13703039518341
C	-3.39675637668832	-0.96892777023124	-0.76660545566805
C	-4.58500326417389	-1.03821786353091	-1.52780077348286
C	-4.58396016733390	-0.58782406715739	-2.85030797653248
C	-3.38928260295249	-0.09085529090721	-3.39758065689925
C	-2.25085470610430	-0.05577584193914	-2.58706597051524
N	-2.24636967867600	-0.47573158379876	-1.30302621826749
H	-5.50259936300760	-0.63467704434546	-3.45339017206286
H	-5.49199781256696	-1.46278585167090	-1.07590153210440
H	-1.29753008409909	0.34278571338026	-2.96567786881381
C	-3.40789243069674	-1.39046391362041	0.62840072484994
C	-2.57537401622797	-2.03137175288728	2.53591144432815
C	-4.39754412220609	-1.45430849496800	1.59425066377904
N	-3.84244329095779	-1.85589218824489	2.79588466717890
H	-5.46077310159235	-1.20326190731011	1.50152898123505
O	-2.23107462808931	-1.77130604935025	1.23770654250075
H	-1.77246361839911	-2.36769778216185	3.20218062525196
H	-3.33617133370361	0.26816000513863	-4.43466771421450
C	1.20249665013700	0.43978366162229	0.91217996420457
C	2.37253901316742	0.49765588830416	-0.02436694059354

C	3.48672127974602	-0.55646604136349	0.00469550423603
C	2.61245856998923	-0.44779991384743	-1.22299525398413
H	2.72889757962016	1.52341276139750	-0.21765260403630
H	3.11922299839980	0.05677543706929	-2.06373981476950
C	3.47809526478175	-1.75255628495964	0.95452169430463
H	3.83522839390299	-1.41003136237330	1.95143783972672
H	4.22197639925731	-2.48411678773664	0.56840088530835
O	2.19048269391860	-2.39027028642894	1.10012760951247
H	2.31436756417925	-3.23955991482470	1.56415237643274
H	1.24971967188594	-0.34225626492866	1.69315780714078
C	0.49010816725135	1.63836484097833	1.29107878453144
C	-1.38220569865042	1.71881346366403	0.41022010341175
C	-2.46671270767664	1.60439387730271	1.30889554274391
C	-1.43191108631177	2.68589294062222	-0.62143588238770
C	-3.61553141600276	2.38968011078623	1.12831461074900
H	-2.42365065295848	0.89067216860839	2.14507914216347
C	-2.58212057727194	3.47065480406196	-0.79558952382942
H	-0.57116214732556	2.81582109667759	-1.29694549613733
C	-3.67857598409369	3.31765456493080	0.07318934426246
H	-4.46480441390878	2.27782474003377	1.81943032836846
H	-2.62002495708855	4.21233934776137	-1.60800061139979
H	-4.57695596666438	3.93870058998889	-0.06041895825747
H	0.80274485404493	2.55489869591617	0.76289627232186
C	0.14284407634272	1.84166476402016	2.74753814815013
H	-0.28553998748216	0.92506579282063	3.19839800625196
H	1.08192966870972	2.07222774825394	3.29561732186217
H	-0.56193977380045	2.67896111190242	2.90005753396119
C	1.61131799598516	-1.46499023838284	-1.74345882101194
H	2.13965325121433	-2.39205418111795	-2.06010560341910
H	1.10830247673581	-1.05237015673884	-2.63997444165259
O	0.55935124312133	-1.83135107525912	-0.83358758723107
H	1.03901154871489	-2.20910127553593	-0.03412204093226
C	4.90589060389950	-0.04665009873720	-0.10696351771220
C	5.40472789040856	0.87588771809577	0.83481509039460
C	5.76340496455046	-0.52975966655236	-1.11556871192033
C	6.73546080833427	1.31699980095983	0.76295550229639
H	4.73940323957968	1.25989423348245	1.62484839178181
C	7.09450424969374	-0.08961835464323	-1.18879925982194
H	5.38280849858533	-1.25426499595278	-1.85333250239695
C	7.58218833797280	0.83485116020364	-0.24964264631106
H	7.11317358153283	2.04282846430554	1.49870402593760
H	7.75412812386797	-0.46851786538312	-1.98389796957885

H 8.62445326744577 1.18245338022817 -0.30860848965046

R = iPr (S configuration)

72

TS_F1

Pd	-0.48244580666285	-0.25432653833099	0.08370553232025
C	-3.36109498699145	0.11245924134474	1.24095580604694
C	-4.57691998143515	0.80777115287560	1.42213000481206
C	-4.62206995696254	2.18525313802099	1.19088319048688
C	-3.44391193854472	2.85030673141010	0.81126332248719
C	-2.27662390688260	2.09851369025242	0.64717075627457
N	-2.23441183813345	0.76015490922396	0.83332162199944
H	-5.56243251578998	2.73963459741488	1.32585299167621
H	-5.46640818964187	0.26191852712011	1.76591791604799
H	-1.32966434409018	2.57261139279534	0.34862670218718
C	-3.30000669966820	-1.32385433814703	1.47651559177248
C	-2.35497326027096	-3.24571290132148	1.88125357708397
C	-4.23442946541387	-2.34324786695431	1.43588104002553
N	-3.60862765798218	-3.55128829537223	1.68621481037788
H	-5.30493117024181	-2.27983654414777	1.20761064688208
O	-2.08502001633089	-1.90688671094085	1.77524070941252
H	-1.51247446861310	-3.90155783020545	2.13056928199196
H	-3.42439143542231	3.93577095235533	0.64220661999662
C	1.22112621075338	-1.08887452660524	-0.73420265970681
C	2.14865418071755	0.07110203204336	-0.82193101456093
C	2.54474815740863	0.85500671590214	0.44611577353608
C	3.59194150004140	0.07647394964271	-0.32435157326538
H	1.97319235341886	0.70539179181978	-1.70582412846602
H	4.26009717347509	0.71548287491820	-0.93001662804554
C	2.07736898047122	0.30311149338881	1.77622746872265
H	2.51944114030323	0.89666023783373	2.60061075017740
H	2.36264236703002	-0.75703211388514	1.91820866751142
O	0.62811069342820	0.43815061237286	1.83981240523318
H	0.30021358696800	0.09969695720756	2.69570545706043
H	1.49381050434513	-1.90657983174736	-0.03946381715797
C	0.39796169578944	-1.47507442578080	-1.85198674531530
C	-1.51493358234105	-0.68907890658408	-1.63653646494248
C	-1.72440896145302	0.44559506395475	-2.45816849534165
C	-2.52096696219794	-1.67563225198984	-1.54481112085854
C	-2.95341755241707	0.62541935255130	-3.10868339132181
H	-0.92764598073774	1.19719951791238	-2.57776636279426
C	-3.74813004184335	-1.49195445561138	-2.20228697623695

H	-2.35591371688155	-2.58715689455298	-0.95174350726883
C	-3.96997949725526	-0.33998264124595	-2.97618174487064
H	-3.11610982040267	1.51779015689134	-3.73198900259328
H	-4.53327460397516	-2.25787856328459	-2.11065811355821
H	-4.92955289891976	-0.20261951537162	-3.49665398950579
H	0.53762065912213	-0.87129793301039	-2.76366367866279
C	0.13875791695677	-2.94018139565693	-2.09777230799746
H	1.08112784902889	-3.39549497985773	-2.47208900334490
H	-0.13847287720564	-3.47159418645311	-1.16640338750232
H	-0.64856142942282	-3.10735193132540	-2.85486748606155
C	4.29714993538584	-1.12978372322288	0.27931792408519
H	3.54577816633701	-1.86442342778157	0.64220042118113
O	5.00217084230690	-0.73289601339814	1.45685697867093
H	5.75686675756902	-0.18060589487203	1.17298890774571
C	2.51619530964138	2.35735551352602	0.35963103988688
C	1.45379430214755	3.02723034815382	-0.27826471083821
C	3.53623194162618	3.11579346818574	0.96815922634595
C	1.40871442657435	4.43044257185546	-0.31008230541359
H	0.65987447754005	2.43120901344969	-0.75858869639501
C	3.49403071596928	4.51837082660195	0.93895889373136
H	4.37017111470658	2.59352644145384	1.46450559417610
C	2.42935549421880	5.17836828862328	0.30060221371138
H	0.57816968762712	4.94412838210159	-0.81822620086155
H	4.29841197498964	5.10110707220571	1.41265836711038
H	2.39927097702494	6.27789657098473	0.27417081567733
C	5.19383941441915	-1.86087139237258	-0.74360906596726
H	5.94444024211135	-1.11412877650393	-1.10033516153953
C	5.94204800800040	-3.02364544114499	-0.07863705565478
H	6.50090273842531	-2.68450761239188	0.81383598108576
H	5.22960646950091	-3.80881613017302	0.25317260406082
H	6.65590279065651	-3.49334007996259	-0.78383030360757
C	4.38450348514438	-2.33608680085270	-1.95972284729929
H	3.88355952546548	-1.50151343410224	-2.48954588185967
H	5.03859495132226	-2.85313060870348	-2.68935418984178
H	3.60187995506279	-3.06117657262947	-1.64756688053417

72

TS_F2

Pd	-0.86450361298026	-0.53901840112179	-0.00060681312116
C	-3.13219731379540	1.51590049246972	0.31335510111426
C	-4.14518392454300	2.23502644486366	0.98126557406379
C	-4.68278057725208	1.72526619841757	2.16860711007006
C	-4.21669207712679	0.49088671278895	2.64784417126176

C	-3.20795570516401	-0.16796187625059	1.93515007635731
N	-2.65722187778564	0.33694273819932	0.81077296195990
H	-5.47356702866608	2.27446502313825	2.70074184345478
H	-4.51985974754081	3.17095114266577	0.54376268079280
H	-2.80968406112797	-1.13899188546259	2.26750082489495
C	-2.56432985721190	2.03581938485297	-0.92813948617876
C	-1.59751934449934	1.92178873945935	-2.87661496652709
C	-2.28613393263482	3.30344918464504	-1.40391190790586
N	-1.66027912926050	3.20386145452109	-2.63700593580820
H	-2.46921912873110	4.26988873199858	-0.91972579642397
O	-2.12481608359679	1.14385184327119	-1.88540646368101
H	-1.20576909916343	1.39894838227891	-3.75697935594730
H	-4.62400710839134	0.03769740813546	3.56238297833150
C	0.85449091907485	-1.23416364214977	-0.85667263104905
C	2.03762966952423	-0.91328646971298	0.00749193542859
C	3.05485367595402	0.16741162895490	-0.38262961551199
C	2.20438190584558	0.36500540573744	0.85653975057712
H	2.48162798922569	-1.80066139246491	0.48936935128678
H	2.76652354269597	0.16715335944486	1.78360252398214
C	2.94486249831193	0.99291364218899	-1.66514566750782
H	3.31723553137902	0.37249133274671	-2.51037794243840
H	3.63457008462077	1.85815087904525	-1.55979381101357
O	1.61339962782786	1.46341798937340	-1.97424763567481
H	1.67495266801180	2.21643295364236	-2.59258774824706
H	0.82783664511560	-0.72672096604545	-1.84009727422248
C	0.22450213505772	-2.53810652116398	-0.83062706615642
C	-1.54623091925543	-2.47422130005480	0.16171900871090
C	-2.70909954158338	-2.75630622303299	-0.59322080284658
C	-1.39645620635866	-3.05582542190905	1.44624722560598
C	-3.72993368825519	-3.54779671411661	-0.04499360571240
H	-2.83049258917862	-2.32740447396408	-1.59919168439788
C	-2.41366756784469	-3.86194442273176	1.98225602229680
H	-0.47353318976620	-2.88252684148377	2.02272834828368
C	-3.58439566690281	-4.10325912989454	1.24000993311199
H	-4.64444905793773	-3.73722142916102	-0.62722038336903
H	-2.28657429936027	-4.31332661425654	2.97805659445788
H	-4.37924246433441	-4.73953008264544	1.65706028615445
H	0.64589715279299	-3.23741771288832	-0.08913650372134
C	-0.15217026893921	-3.18482538089621	-2.14508043850031
H	-0.67285525303664	-2.47467638036778	-2.81683504425972
H	0.78128070450759	-3.50477654797761	-2.65615318520566
H	-0.79176532166249	-4.07532448080926	-2.00461392216959

C 1.12128476994933 1.40869273910091 1.10315069879462
H 0.59204294833181 1.12327470254585 2.03872147686510
O 0.08720721916811 1.40547180805909 0.09116199745889
H 0.57158658240125 1.56567221885011 -0.77984954505062
C 4.51604606954987 -0.16499781441143 -0.17425270682330
C 5.05584299702301 -1.36789871587126 -0.67096427872340
C 5.37157625045910 0.75821913082756 0.46115379852326
C 6.42243923612865 -1.65270201564553 -0.51839718727312
H 4.39636174837125 -2.09058560362156 -1.17722105567115
C 6.73752174935503 0.47538011049938 0.61551479440490
H 4.96053931453559 1.70744852507585 0.83996562474980
C 7.26490387425034 -0.73234383249332 0.12715161975084
H 6.83151259458025 -2.59839913189298 -0.90485757600876
H 7.39378863346178 1.20036891351959 1.12004096728357
H 8.33510743902335 -0.95637232616025 0.24997486663501
C 1.66840922893237 2.84409690211694 1.27555652638602
H 2.21917444218669 3.09317892349147 0.33867282236305
C 0.51925370933362 3.84740376653485 1.44811345701527
H -0.06655986704359 3.61797136816891 2.36367499679858
H 0.91267741089610 4.87800355441191 1.55153270472861
H -0.17674306999044 3.82372018812800 0.58848802326880
C 2.64964756619900 2.92449084103111 2.45423485578328
H 2.14937553827208 2.63915931103167 3.40387854768155
H 3.52811153910776 2.26348519136765 2.32497054928906
H 3.02539277435899 3.95935540815763 2.57706717797009

R = iPr (S configuration)

72
TS_F1
Pd -0.53102357122370 -0.22825997881650 0.00910204315484
C -3.30543473667409 0.12705423513127 1.40517403714819
C -4.49280313131886 0.83104632179175 1.70444674012025
C -4.52673385958916 2.21930378625840 1.54846672355090
C -3.36422359155231 2.88486519054571 1.12385290057328
C -2.22562596789972 2.12393188945048 0.84281953361725
N -2.19578603377439 0.77737318563595 0.95722043609636
H -5.44514689681271 2.78055840952919 1.77512150791948
H -5.36815940266016 0.28213423183523 2.07837680306151
H -1.29239592803670 2.59808654671286 0.50391058012124
C -3.25763070421044 -1.32068085380929 1.55890769715718
C -2.32705871198506 -3.27852294064421 1.78619078634713
C -4.21439275485213 -2.31972172751446 1.53505116817173

N	-3.59815972747963	-3.55059383578581	1.67168052783465
H	-5.29738810176095	-2.22499770258005	1.39291857142125
O	-2.03655289011503	-1.94089252131637	1.73226410364301
H	-1.48257078490810	-3.96234761160339	1.93238763077089
H	-3.33571003132017	3.97721032669590	1.00895260141576
C	1.08022972870668	-1.05076753857446	-0.98627388640532
C	2.05074475678803	0.07440056465625	-1.03418442535300
C	2.60886109610704	0.68974460041482	0.26399655931042
C	3.51964055865749	-0.09240074536366	-0.67476959902968
H	1.84116595059783	0.81121835298730	-1.82606947549878
H	4.19640181643137	0.55521382693085	-1.26131940711826
C	2.16299700104661	0.06829192376643	1.57030155091673
H	2.68945147346038	0.55874352368606	2.41413037873659
H	2.36487863065187	-1.02110192528473	1.60999310258288
O	0.73632805436278	0.30286174960017	1.72446067602564
H	0.42942351465219	-0.05859216186272	2.57874008830229
H	1.36545333645077	-1.93815817746921	-0.39204571898033
C	0.17837901560968	-1.30857584915995	-2.07694951052649
C	-1.70867899666098	-0.53595675714480	-1.64105417593080
C	-1.97945273259580	0.64949755521289	-2.36692242087593
C	-2.70868501408627	-1.52677671273630	-1.53014826182664
C	-3.25686489836534	0.87013093596008	-2.90168743165266
H	-1.19116136313861	1.40749880429996	-2.50083507272454
C	-3.98499820356295	-1.30132220422970	-2.07046858118196
H	-2.49840778164717	-2.47471467728269	-1.01330296709466
C	-4.26392429467493	-0.10200148806568	-2.74817640825774
H	-3.46581605978211	1.80079102403385	-3.45082994219064
H	-4.76368764213985	-2.07188335247592	-1.96389835817096
H	-5.26226521497305	0.06818925479156	-3.17816376679988
H	0.26486427585144	-0.61563063241697	-2.92992948465373
C	-0.11915969512020	-2.73881467166989	-2.44687246277792
H	-0.34442682722301	-3.35662895172873	-1.55589158750897
H	-0.95172671273954	-2.82584002801223	-3.16805886989532
H	0.80145939428627	-3.15691034606151	-2.90853235561432
C	4.08311511031325	-1.49224301037736	-0.43890216359998
O	3.85145651592516	-2.29812724072274	-1.59585669412944
H	4.38768701076858	-1.92603214714797	-2.32344285006821
C	2.67506341964540	2.19654931774457	0.29840586721914
C	3.79070160728302	2.86663994328681	0.83824894172942
C	1.58239321628210	2.96027737099239	-0.16164701306983
C	3.81560060334685	4.26853301490985	0.91221482636154
H	4.65263299340688	2.28499828892037	1.20056246040065

C	1.60205214853671	4.36157724004435	-0.08711815307123
H	0.71070776275739	2.43630143974590	-0.58851349415692
C	2.72096079929792	5.01928449894776	0.45112048438092
H	4.69728362778205	4.77814576047906	1.32904861332166
H	0.74479960890575	4.94506474255867	-0.45639726477224
H	2.74283057399281	6.11791493022745	0.50637758538257
H	3.51468215497565	-2.00778765761227	0.36533117041942
C	5.56847911429333	-1.48000158356810	-0.01526230058786
H	6.12755408285610	-0.96280372741418	-0.83263459690093
C	5.77670054985484	-0.68324107870176	1.27996004857905
H	5.20809277994413	-1.13656489663530	2.12047398023802
H	6.84502956687933	-0.67922715758465	1.57299576372388
H	5.45564500679492	0.37217808494128	1.18056004575420
C	6.10983939445239	-2.90979086627505	0.11591558038914
H	5.60875972368584	-3.44311631898207	0.95185525691829
H	5.93146793384654	-3.49364251126557	-0.80654702689238
H	7.19774529239576	-2.90413742762916	0.32555307670014

72

TS_F2

Pd	-0.78062329755097	-0.37275569963165	-0.23333675328842
C	-3.10724960963070	1.48528386972575	0.33008417136288
C	-4.19188217970268	2.02978706542034	1.04219915033681
C	-4.87657374511471	1.22587894700005	1.96487869952796
C	-4.48075176537507	-0.11012404387005	2.12786972210644
C	-3.38742290981052	-0.58291206111965	1.38996415756024
N	-2.69967913883726	0.20002863848277	0.53619111263692
H	-5.72510982864881	1.63419177220086	2.53350413781922
H	-4.50020939708034	3.06641359382198	0.84696587548097
H	-3.02763796951272	-1.61964190113611	1.48473517102600
C	-2.35177283770140	2.28757341293902	-0.63867346201796
C	-1.24436934722397	2.63823743525783	-2.48187209482773
C	-1.74869473845437	3.52753745599989	-0.60620943943780
N	-1.03512439821425	3.72247429060105	-1.78208930410619
H	-1.75149745860977	4.26262929984238	0.20679559779932
O	-2.02908401963151	1.71645420018015	-1.85775680932429
H	-0.88584120539082	2.38442871232489	-3.48630869984337
H	-5.00360471024457	-0.78258847114886	2.82228924511270
C	1.01902923664422	-0.69549849642657	-1.15182372670277
C	2.17630542712837	-0.67535257174744	-0.20207757606104
C	3.09930640303005	0.54946777737340	-0.09714236216057
C	2.24663342534509	0.16195783436541	1.09199653642369
H	2.69443523633060	-1.64526428157830	-0.11330897271725

H	2.85011518508523	-0.32961679786127	1.87022178968831
C	2.93155132035065	1.81922882472021	-0.93651949690154
H	3.38681234767259	1.63867997323087	-1.93546814125142
H	3.52269709673936	2.62207366289695	-0.44338293477628
O	1.56448513992286	2.23962870180655	-1.10539359619803
H	1.50656691460214	3.14617142139651	-1.46920368516292
H	0.99563092965211	0.13993404241522	-1.87779142083006
C	0.43392032111686	-1.92714764112217	-1.63594253199113
C	-1.42719230469510	-2.22886813507972	-0.83629558799462
C	-2.51016578020706	-2.24907597761659	-1.74461721070176
C	-1.36219430132967	-3.20370269650581	0.19029659584627
C	-3.54250033475644	-3.18677550400052	-1.58583615983043
H	-2.56530197934167	-1.50769880002775	-2.55549651923188
C	-2.39231107984722	-4.14701990927071	0.33592865391744
H	-0.49807720278606	-3.22596078076172	0.87377256571384
C	-3.48635070513047	-4.13632020181562	-0.54879074478188
H	-4.39681405015978	-3.17784449862822	-2.27982225934491
H	-2.33311639241991	-4.90236324509781	1.13451053111741
H	-4.29028778374234	-4.87954935790300	-0.43951928496728
H	0.82172486458191	-2.84290103546151	-1.15817865005927
C	0.16612912105852	-2.06424014139675	-3.11818017054735
H	-0.44533570171079	-2.95429615835208	-3.35468190972316
H	-0.33836163298933	-1.16627175638880	-3.52530314346766
H	1.14221714352277	-2.16488176258916	-3.63975486437286
C	1.08611482181403	0.91540826525182	1.72455194028065
O	0.09730346941252	1.35633183383739	0.76653660583641
H	0.59622106999468	1.86548818448901	0.04728803843545
C	4.58119477895716	0.26456052765968	0.01442896465099
C	5.32658582256854	0.75547357819215	1.10485668321150
C	5.24446623624810	-0.44630899601920	-1.00616058700906
C	6.71064321529313	0.53160381401726	1.17900888954588
H	4.81565155116099	1.31433446318331	1.90547279054975
C	6.62809297978197	-0.67146214269149	-0.93356096298510
H	4.66603912018063	-0.83600627605393	-1.85919909381522
C	7.36321885943625	-0.18222303208913	0.15968649156070
H	7.28271167504183	0.91494083162297	2.03732249073377
H	7.13522347067231	-1.23377303643348	-1.73208084963322
H	8.44740555826806	-0.36013824584331	0.21875702217660
H	1.49743545737901	1.83517707497114	2.20531585346665
C	0.33411751987737	0.10384913020209	2.80304521833679
H	-0.14683592013857	-0.74552082679123	2.25562209010577
C	-0.77094239018231	0.96083823578310	3.43938217640089

H -1.44553734518723 1.39458568411526 2.67890040998818
H -1.38064463941638 0.35567018829262 4.13906339840834
H -0.32965964156661 1.79856705003795 4.01970625884671
C 1.26237958626572 -0.47167107113404 3.88141206366009
H 0.66325076294977 -0.95153709025706 4.68035178999417
H 1.95805618926851 -1.24067945450899 3.49202469271787
H 1.86644942378743 0.32707227790189 4.36185576208078

R = Me (R configuration)

66
TS_F1
Pd 0.41212214845522 0.38035664794697 -0.30643019977784
C 2.72895454533908 -0.71325161934206 1.52857271450576
C 3.46066256538245 -1.68653555466776 2.21173276622417
C 3.20838147611508 -3.02890984800946 1.94483234327206
C 2.22846166023531 -3.36135405872022 1.01228246022099
C 1.54039741497741 -2.33215746704162 0.38412127042262
N 1.78624607024566 -1.04220615757067 0.62919214159115
H 3.76395268350661 -3.80596788427859 2.47358660928724
H 4.19801050779408 -1.38238362289985 2.95591408455158
H 0.74991460351544 -2.54761008450223 -0.33848181173286
C 2.99127070801267 0.71181778842733 1.76554980339318
C 2.50520045457990 2.79450622583823 2.01387863519626
C 4.12763119696473 1.44751146942834 1.88263129269149
N 3.78990271906175 2.77597329474048 2.03452133877096
H 5.16019123295534 1.10301766515258 1.84466398244872
O 1.94048210590091 1.57842684386932 1.85362418658403
H 1.83168774362968 3.64433513234690 2.11965063343642
H 1.98017816472318 -4.39651199366932 0.77029716272530
C -1.09500616513841 1.44001450800822 -1.17965650190394
C -2.21343851566619 0.44629546377186 -1.12029620727681
C -2.69378178517524 -0.11299529446024 0.22161697529336
C -3.60586091684700 0.74581317518863 -0.61060194806786
H -2.18063523633409 -0.28419141211516 -1.93574111166719
H -4.38298142935838 0.17622142533281 -1.13385516954882
C -2.10022402115580 0.50542988611871 1.47199731206663
H -2.62648849702799 0.13163392580115 2.36031940402613
H -2.18115973663218 1.60088054600296 1.47869123799682
O -0.71892007609514 0.12209795685050 1.54799031491523
H -0.27905274041395 0.58137208502321 2.27474076145711
H -1.22990690311036 2.37263173540167 -0.61426875796055

C	-0.23008290935934	1.53524389840884	-2.31203552485646
C	1.60382728607814	0.60201733674326	-1.95237887225637
C	1.78541586906309	-0.51017803977093	-2.78614416790596
C	2.68175986209629	1.46252319951460	-1.70636770914444
C	3.04513200246888	-0.79267098542864	-3.31176025310298
H	0.94391485726859	-1.16925149265118	-3.02198389512883
C	3.94080507263970	1.17582130752948	-2.23321322129677
H	2.54764817701363	2.35108865251644	-1.08080876425552
C	4.12451147282215	0.04508497021796	-3.03053616749010
H	3.18699347070187	-1.66719305219692	-3.95046956405864
H	4.77918203927926	1.84266697105882	-2.01846171104003
H	5.10792110570231	-0.18627725305367	-3.44775789361037
H	-0.44351410427941	0.83036911728854	-3.12194023586412
C	0.24066154578142	2.89420264546575	-2.77730164415979
H	-0.61655626608771	3.40706335292452	-3.24230487933073
H	0.59026691751238	3.51701434698747	-1.94225313388976
H	1.04067770909788	2.82690989521397	-3.52405798397492
C	-4.11147616986807	2.07518892026550	-0.07925589097389
H	-3.26937208925691	2.76433289884808	0.09782215556332
O	-4.69047659462296	1.87953448256936	1.19454168641621
H	-5.53190834246858	1.42317839707050	1.08222566414390
C	-3.02877782477411	-1.57547972623867	0.36344398018537
C	-2.40796887647500	-2.58013460576896	-0.38059856276582
C	-4.00488139817477	-1.93887410226922	1.30149556415324
C	-2.74871020747042	-3.92059059163636	-0.18265578395665
H	-1.65279330927433	-2.31312900927564	-1.12625533249303
C	-4.33838841774881	-3.27278998590339	1.51167877042781
H	-4.51039716202715	-1.15383274128374	1.87082035036392
C	-3.70423404273733	-4.27017347664281	0.77003487208748
H	-2.26479556657737	-4.69735962985069	-0.77915861790832
H	-5.09842119997817	-3.54012200333773	2.24891508805579
H	-3.97041921718052	-5.31748293280368	0.93592957856585
C	-5.07231454438123	2.73528316676603	-1.05130269127532
H	-5.91669041720550	2.06567244126327	-1.28026890288976
H	-5.47754405384086	3.66314798621654	-0.62328213512752
H	-4.56331005777679	2.96975056237062	-1.99924569194812

66

TS_F2

Pd	0.83016318655513	0.25240196908526	-0.13401075667593
C	3.24521320047366	-1.62211494620892	0.52288707466562
C	4.30935282585431	-2.15075939872586	1.25945744669322
C	4.74838472940524	-1.48503379616472	2.39561563563102

C	4.14193491293971	-0.27959877627053	2.73767823896809
C	3.10663054268564	0.19426953848040	1.94018672814988
N	2.64815686542080	-0.46472154444791	0.86849868880319
H	5.56527664846527	-1.89505319756742	2.99360696179347
H	4.79349610589947	-3.06767746151066	0.91968980719727
H	2.61413026057714	1.14461972466755	2.16623382537245
C	2.79013976731328	-2.34379159682109	-0.66888120667857
C	1.94345315214947	-2.54140919827381	-2.63211572697313
C	2.82447320347596	-3.64775639691410	-1.06523384986086
N	2.27178185556710	-3.74783600647983	-2.32394372382229
H	3.19777026041425	-4.51622037747192	-0.52436524437483
O	2.22063643676045	-1.63622218540292	-1.67857095580946
H	1.50108172387570	-2.15746421195818	-3.55100555789007
H	4.46997466270945	0.29588290844720	3.60400552339124
C	-0.89108018600295	0.78467707376675	-1.08869280510175
C	-2.00228261133539	0.74484181678086	-0.08095274999536
C	-3.14105413645909	-0.26314936338297	-0.13030930229391
C	-2.15668169171385	-0.31756537083057	1.00595881333706
H	-2.31170190415688	1.73985590043401	0.25846108487282
H	-2.57042977760807	0.09233231147897	1.93288551337400
C	-3.27656188183188	-1.34030083796170	-1.19611795869966
H	-3.70968327561851	-0.87694104577681	-2.10025603889512
H	-4.00650436920571	-2.07525882956029	-0.81413879609494
O	-2.05390197507163	-1.99343707524371	-1.52487908356954
H	-2.19594500761696	-2.60657496279014	-2.25339747126526
H	-0.92965529274721	0.04010140258164	-1.89481665264497
C	-0.22986999688951	2.00615972958458	-1.40855967297505
C	1.42824157774984	2.20943256428231	-0.18217032796830
C	2.64179364682478	2.52517219544993	-0.80476093831898
C	1.02844239004324	2.93754262018439	0.94928733844604
C	3.44801470208638	3.53815776828468	-0.28381848599111
H	2.97022456683633	1.96570720143893	-1.68586950067395
C	1.83264552722213	3.95269819360998	1.46431004131730
H	0.06467869707085	2.71860372937882	1.42157202778994
C	3.04807304048191	4.25133237935079	0.84802712075905
H	4.39678767806385	3.78064737386331	-0.76792642635820
H	1.50101593395203	4.51843995686488	2.33865424540648
H	3.68313776099067	5.04431230146523	1.24773864187887
H	-0.62022471809322	2.89889172314698	-0.91497303215365
C	0.28256691551645	2.25951615553708	-2.80614717305638
H	0.69541578865267	1.34851983916809	-3.26499535508169
H	-0.56163658878899	2.60608751377445	-3.42277898838088

H	1.04720084830786	3.04643077782706	-2.83515209844496
C	-1.16124631761685	-1.42300847121649	1.33231012591869
H	-0.54743043413482	-1.05916773395273	2.17171277075596
O	-0.21284632354673	-1.65910752411573	0.28493075570834
H	-0.73217067540682	-1.97668230898857	-0.48476739762634
C	-4.50966396769345	0.27456489064161	0.21069812382860
C	-5.21793685340667	-0.25777983640143	1.29091514276784
C	-5.08444178118915	1.30141509542311	-0.54456128656215
C	-6.46809688397404	0.24919135142008	1.63551021340165
H	-4.77128854356336	-1.06711065232078	1.87728961496765
C	-6.33447719678706	1.81333373492952	-0.20084286021987
H	-4.53527857287163	1.72274938277317	-1.39228877321017
C	-7.02003081457764	1.29681971157481	0.89893725403158
H	-7.01169793080100	-0.17304129860133	2.48371234507596
H	-6.77142572166416	2.62376732099395	-0.78841750793132
H	-7.98499320334109	1.71802254718557	1.19159715189529
C	-1.85182706767844	-2.71973167689370	1.74914505153599
H	-1.12270720460093	-3.43815707900173	2.14664512233142
H	-2.59766056204113	-2.52625716805449	2.53248760717452
H	-2.37698752880618	-3.18222502436502	0.89968186795800

R = Me (S configuration)

66

TS_F1

Pd	-0.73320627594447	-0.48126745964916	-0.09838304405446
C	-3.09881188237194	1.44105832246674	0.54503974707493
C	-4.21409847651878	1.94216276421171	1.21797412916042
C	-4.85700376588793	1.14598970448831	2.16011039148207
C	-4.38943946482343	-0.14716741546979	2.37206221216340
C	-3.29345187059985	-0.58429274280635	1.63811655292155
N	-2.64333625309292	0.19191647939102	0.76376773981363
H	-5.71627518735653	1.52919350265983	2.71516762216978
H	-4.56430313428999	2.94951981441802	0.98632189206980
H	-2.90368623803737	-1.59855146352098	1.76377292756299
C	-2.35338371331725	2.31171380960700	-0.37940815407161
C	-1.31985693871608	4.12185487945633	-0.89080448343018
C	-1.70563213207069	2.15662403335063	-1.56721034318104
N	-1.03456474627217	3.32614799343834	-1.86712991101642
H	-1.66070632695174	1.27976852229877	-2.20987957808746
O	-2.12271550914390	3.58588767450589	0.03689708896947
H	-0.98370827658456	5.14514068087717	-0.72611805691194
H	-4.86197524306568	-0.81931160375876	3.08931613318352

C	1.05042703546752	-0.88975638893412	-0.97724158189855
C	2.16981347352703	-0.74376469936554	0.01636904190055
C	3.16081325751275	0.41026373474578	-0.03311223369260
C	2.25041469529421	0.27745171285096	1.15490873410184
H	2.62092824609662	-1.70289598135768	0.29445006418181
H	2.76083382560785	-0.13478234704804	2.03189872402657
C	3.07339345352195	1.54766169239944	-1.04879683552109
H	3.54809406275545	1.19670612429464	-1.98218801449815
H	3.68190537468171	2.38035624392433	-0.65305282798798
O	1.75042743454973	1.98962439647498	-1.31583109661519
H	1.72612254297926	2.71018123568675	-1.95581242902449
H	1.08244054603573	-0.18495545021636	-1.81923541078329
C	0.46947013770243	-2.16435530143988	-1.28005659256670
C	-1.47041621363400	-2.33499480873036	-0.63761332952451
C	-2.48290855199686	-2.26853359673704	-1.60926616704608
C	-1.53158895472790	-3.34921195545894	0.33160215599627
C	-3.55810442328782	-3.15427921660843	-1.57529182722384
H	-2.44087666158569	-1.51102168258321	-2.39714984091705
C	-2.59876363030486	-4.24987699090068	0.35169137443166
H	-0.73076437329914	-3.45790126390837	1.07066756559420
C	-3.61769869003174	-4.14841809485661	-0.59505102086801
H	-4.34990940956949	-3.07157932375044	-2.32395595278919
H	-2.62511292422900	-5.04368280677341	1.10228355520007
H	-4.45108500516362	-4.85472733438308	-0.57809356524169
H	0.79785026505139	-2.98410982609786	-0.63319311663684
C	0.28842320827200	-2.56918558368545	-2.72646238152412
H	-0.11492138783661	-1.74565592664487	-3.33267429720630
H	1.27982385685420	-2.82952019563050	-3.13194095321921
H	-0.36394256964879	-3.44363157156778	-2.84004239793838
C	1.16972200435465	1.26861528413672	1.56127177040024
O	0.13770678717933	1.37903271766148	0.58388869121928
H	0.56559782014628	1.77517047905244	-0.21247052621024
C	4.61741763649585	0.09469942808155	0.20565377456145
C	5.29760927933552	0.70687662179409	1.26402475929603
C	5.31670538945755	-0.76297783536356	-0.64842914230329
C	6.64560647201141	0.44059787303365	1.48862427165768
H	4.75733085115860	1.38859921383491	1.92760294952593
C	6.66446020604214	-1.03727669850071	-0.42302130019489
H	4.79486648959089	-1.23559171834878	-1.48625949964710
C	7.32615755558086	-0.44413849088720	0.65253530545365
H	7.16670691825049	0.92309019611928	2.31876316439752
H	7.20123367307510	-1.71722432376419	-1.08823609073825

H	8.37387863092596	-0.68430220387245	0.85107691240654
H	1.63532390641973	2.26730903427397	1.64235045533424
C	0.52291410354173	0.93750273090862	2.89444548818750
H	0.13776317429174	-0.09501488554114	2.90993173273102
H	1.24834987481853	1.05716977838690	3.71127851976006
H	-0.31478991512557	1.62381257863148	3.07972593983438

66

TS_F2

Pd	-0.36565050259444	-0.35222032389272	-0.18643600252434
C	-2.93801320534854	0.79301628743564	1.28183848291779
C	-3.96342569021387	1.71595035764300	1.50811644290576
C	-3.88324250424117	2.97491602993201	0.92406021476363
C	-2.76481053946804	3.28776856754652	0.15666887225282
C	-1.79036590425551	2.31609542236485	-0.01660335315563
N	-1.87726940840856	1.09109389801598	0.51348320850049
H	-4.67730229381446	3.70843285047456	1.08113570520741
H	-4.80193389963542	1.44104127576273	2.14938839798357
H	-0.90370843127353	2.52104291172958	-0.62089369269754
C	-3.03683376624703	-0.54824943709270	1.86687391490410
C	-2.36497364484215	-2.35372243894075	2.82897589599283
C	-4.08628272870438	-1.38624612556124	2.08260014031288
N	-3.63232260866392	-2.53366833430280	2.69783540610456
H	-5.12928487141737	-1.24226543227775	1.80698245020270
O	-1.91842959367073	-1.17463427298722	2.34449724607178
H	-1.62629654503186	-3.01556567288367	3.28009066391234
H	-2.63467107315752	4.26667074502750	-0.30794572159553
C	1.18286684017008	-1.45655001977145	-0.93649864699101
C	2.28548041718439	-0.44879742467503	-0.93278710941764
C	2.80836003734784	0.08335931157795	0.40237238481617
C	3.67758334666173	-0.78984142781132	-0.47526457727090
H	2.22107008881754	0.28250701500043	-1.74486886629971
H	4.44791357685076	-0.21438868666811	-1.00168127076733
C	2.14951996459352	-0.48321079742020	1.64467466415551
H	2.65590347266189	-0.10580020776277	2.54481518643640
H	2.18217994112424	-1.58320923400426	1.67019755242935
O	0.78714816126877	-0.03616581059171	1.65532955492327
H	0.30485723508792	-0.35326697958210	2.42941988179087
H	1.31134424583841	-2.32730372939526	-0.28084639508800
C	0.34861116757848	-1.67481372265940	-2.06501087950889
C	-1.48317927868030	-0.62327832125812	-1.86507366653271
C	-1.54628884108191	0.44114169833612	-2.77302409994626
C	-2.62578819736373	-1.39314485340158	-1.61784493472181

C	-2.76156310975356	0.78634937401834	-3.36156506485701
H	-0.64824602489624	1.02051552259081	-3.01272535897553
C	-3.84215235706754	-1.03773002834327	-2.20028641054931
H	-2.57982808153400	-2.24899643181123	-0.93673879520820
C	-3.91368781814364	0.05807314515935	-3.06326270379897
H	-2.81072351508160	1.62795051379171	-4.05667741797630
H	-4.73669808486855	-1.62075959035323	-1.96738802048085
H	-4.86445713511485	0.34376668733749	-3.52060317627925
H	0.57441904102271	-1.06845342324437	-2.94659966414105
C	-0.13476923310945	-3.07161227585766	-2.36520444801676
H	-0.47446081093373	-3.59160169304091	-1.45746484732863
H	-0.94089520298237	-3.09136057975203	-3.10771415790314
H	0.72274890623895	-3.63171490435810	-2.77010942347504
C	4.12178325355794	-2.21769008385077	-0.20384351272620
O	3.97904984615058	-2.99357887418564	-1.37440191994845
H	4.66083953676694	-2.73161206400284	-2.00300666052025
C	3.17481861877424	1.54061524351549	0.53364740642447
C	4.17832413976925	1.89719145494911	1.44439526169633
C	2.53769744921051	2.54980944303178	-0.19143826089600
C	4.53245320388179	3.22835652687493	1.63943081171638
H	4.69305647909919	1.11308672888905	2.00786301936145
C	2.90333468759940	3.88601661041420	-0.01320344798832
H	1.75132735640126	2.28891995781424	-0.90629863961574
C	3.89312860265608	4.22861421249517	0.90669214580126
H	5.31318615249190	3.49067053842161	2.35694265925900
H	2.41061437745576	4.66583996076739	-0.59785603601489
H	4.17949155179661	5.27335253760078	1.05509311272296
H	3.45229967585694	-2.69925237474992	0.52740844614097
C	5.53999783371905	-2.23860394796094	0.35326543560783
H	5.88935427298670	-3.26925808614250	0.50933516556835
H	6.23596459695451	-1.73788066734368	-0.33770513925271
H	5.59740815542403	-1.69833304628099	1.30986623008673

Ring Opening step

53

TS_cis (R = H)

Pd	-0.58033610654764	-0.82411175432299	-0.16478459984549
C	-2.20894968438791	1.80232185982405	-0.71313498984742
C	-3.37715420030703	2.48923613771292	-1.11765969447442
C	-4.27629815567789	1.87165644710914	-1.98928302930501
C	-3.98102286135491	0.58138372371202	-2.46164841994046
C	-2.80747577730442	-0.03742336212907	-2.02349898253344

N	-1.94002559602516	0.54329746908467	-1.15911288391815
H	-5.18843406491335	2.39557755123900	-2.31078183977662
H	-3.55282806107481	3.51293889259876	-0.75956569524915
H	-2.53505603752735	-1.04981029950089	-2.35771962504773
C	-1.29316026698218	2.45112296063688	0.21702894724302
C	0.57558658919074	2.82336522106600	1.27183157764296
C	-1.45216028865061	3.46073797098687	1.15094575458880
N	-0.25533035230549	3.67159617925659	1.81099004775152
H	-2.35751370985407	4.02569792251969	1.40233179265732
O	0.02136008983600	2.04284831232631	0.29240069308366
H	1.64156751911168	2.67494318598693	1.48223342829968
H	-4.64774495679818	0.05669696476160	-3.16007112762181
C	0.72899359121103	-1.93358065203987	0.96187027765838
C	1.65075320825280	-0.92463740800757	0.47651702445569
C	2.81138360013631	-1.09973876266100	-0.46183408474909
C	1.62918788640232	-0.70155095335350	-1.30485965780147
H	1.64562940133445	0.03028441152227	1.02573337156616
H	1.59928482800845	0.36008186371653	-1.60077919757133
C	3.46732863539014	-2.46940192167623	-0.64849405140590
H	2.72134269881927	-3.28112474093439	-0.53773174200303
H	4.21002974649849	-2.61673522637820	0.17006756433699
O	4.03241743702145	-2.62097295026030	-1.93559711589396
H	4.85973091050006	-2.10591204906898	-1.97519083494562
H	0.84599781934788	-2.97046279469043	0.59610449289983
C	0.03701424364366	-1.76293722497007	2.32140972112663
C	-1.32394981603842	-1.14599581658121	1.95306720301025
C	-1.78269707131385	0.07403276540693	2.52902969822120
C	-2.22004231461525	-1.87561071208031	1.09549316562881
C	-3.07241873559577	0.53801638381637	2.27429322581877
H	-1.11112668141420	0.63961384781258	3.19341977136774
C	-3.53193966861791	-1.38486122522347	0.85557324104543
H	-1.97372693975822	-2.90492798574467	0.78917653295893
C	-3.94986898201264	-0.18578325313808	1.43076673424242
H	-3.41401058551826	1.47213738303781	2.74411607987435
H	-4.21654008384773	-1.96726297236421	0.22160521137830
H	-4.96763724235348	0.18875063091475	1.24816823873132
H	0.59082681718238	-1.00959369587823	2.92019232002886
C	-0.07631372671251	-3.06124292150908	3.12442094285347
H	-0.62287336546534	-3.83980125673787	2.55437298405293
H	0.92834835210164	-3.46085854939924	3.36933201149991
H	-0.62233015819299	-2.88844291138723	4.07269560588193
C	1.13665536411662	-1.69731820622584	-2.33915809749473

H	1.85051592580425	-1.66488846850600	-3.19493739985144
O	-0.20597492876329	-1.48761213028499	-2.76142929310464
H	-0.25039827373708	-0.61728269088264	-3.20342347699174
H	1.17576631758486	-2.73122372300368	-1.95068699740865
H	3.55917713217347	-0.29422476610844	-0.34629246312400

53

TS_trans (R = H)

Pd	0.44244666517615	0.06567925885765	0.43608716071616
C	-2.33402508192271	1.05718945970945	-0.62894762788793
C	-3.64139669720843	1.56033163841167	-0.43581547851394
C	-3.93638400380644	2.29821803055414	0.71223657884472
C	-2.91044493766313	2.54007802343716	1.64179952558109
C	-1.64114565761529	2.01437235096114	1.38708363132636
N	-1.34980835650395	1.27757906456239	0.28747329637380
H	-4.94943458143502	2.69521169330365	0.87330637665630
H	-4.40593007920768	1.38492402665032	-1.20507752196791
H	-0.80622409821811	2.16737257529802	2.08743377943645
C	-2.04665407097240	0.27196465934211	-1.82283240396901
C	-0.83495411924428	-0.66696672113140	-3.36990846808787
C	-2.84016337278068	-0.46870384197672	-2.68217361193548
N	-2.04689008694410	-1.06000917571292	-3.64846147886470
H	-3.92414571617119	-0.62976097308664	-2.64675992607766
O	-0.74951616290611	0.14746868763861	-2.27217189498552
H	0.09878403324876	-0.88205819135930	-3.90326845278225
H	-3.08595005228633	3.12718733426686	2.55398733492171
C	2.02808637284407	-1.23741680010273	0.38960598532426
C	2.26307604692808	-0.55635325208547	-0.86840864598679
C	2.29812406260591	1.23574278807302	-0.74171532877132
C	3.40573895158236	0.34582730320995	-1.23930320924207
H	1.66551268458807	-0.91297027021936	-1.72229158580351
H	1.70409926365049	1.72978962435930	-1.52816267741009
H	3.55037866955753	0.39606612238707	-2.33369659458543
C	2.56146883198363	2.05421513710365	0.50922214788991
H	3.21396654926124	2.90817553313931	0.21284399277476
H	3.13887280421102	1.47701604199746	1.25438509542978
O	1.38537904091968	2.49427292843160	1.17877374020363
H	0.89562910404684	3.08293787335472	0.57174186198396
H	2.74495061617227	-1.08071602277103	1.21648187488436
C	1.22625236500633	-2.54569130816290	0.42625541916628
C	-0.19542007210165	-2.08481241395498	0.79263423173915
C	-0.41404744250073	-1.38639827108034	2.03144119540198
C	-1.32880353999926	-2.43332711201018	0.00283808961013

C	-1.73612167876146	-1.07320982936903	2.44744400222052
H	0.41076327579539	-1.27419500000706	2.75336961256362
C	-2.61637491515353	-2.11178184649122	0.43015369045311
H	-1.17735885292918	-2.97661830348708	-0.94271594825501
C	-2.82478776096257	-1.42337081414291	1.65006552840937
H	-1.89047356171019	-0.56348371153481	3.40968225294333
H	-3.47993443809642	-2.40615164054019	-0.18417312946742
H	-3.84767588580473	-1.18559257259518	1.97675248304503
H	1.18054730443594	-2.97191762119451	-0.59787354926313
C	1.79680951446010	-3.59388050892743	1.38474284924353
H	1.86431388334946	-3.19865599968705	2.41865620968863
H	1.15324544761236	-4.49536355947150	1.40777609580196
H	2.81453353452060	-3.90167071717936	1.07084615620839
C	4.73618030492703	0.27087301776004	-0.48765203578572
H	5.34353974046130	-0.55098966976023	-0.93374461634057
O	5.41560539909835	1.51056809600122	-0.47510978980105
H	5.80306908791589	1.66233958051246	-1.35724672858853
H	4.57442900734673	-0.00692443768143	0.57274809973061
